# Enantioselective Assembly of Fully Substituted α‐Amino Allenoates Through a Mannich Addition and Stepwise [3,3]‐σ Rearrangement Sequence

**DOI:** 10.1002/advs.202409334

**Published:** 2024-11-20

**Authors:** Haoxuan Yuan, Yi Zhou, Xiongda Xie, Ming Bao, Kewei Chen, Kemiao Hong, Zhixiang Yu, Xinfang Xu

**Affiliations:** ^1^ School of Pharmaceutical Sciences Sun Yat‐sen University Guangzhou 510006 China; ^2^ Beijing National Laboratory for Molecular Sciences (BNLMS) Key Laboratory of Bioorganic Chemistry and Molecular Engineering of Ministry of Education College of Chemistry Peking University Beijing 100871 China; ^3^ School of Chemistry and Chemical Engineering Zhejiang Sci‐Tech University Hangzhou 310018 China; ^4^ School of Chemistry and Chemical Engineering Henan Normal University Xinxiang 453007 China

**Keywords:** alkyne functionalization, asymmetric catalysis, cooperative catalysis, gold catalysis, α‐amino allenoate

## Abstract

Chiral fully‐substituted allenes are synthetically significant and pivotal building blocks that can engage in diverse transformations toward a variety of bioactive molecules. The enantioselective assembly of these skeletons using readily available reactants offers significant advantages but remains challenging. Herein, an asymmetric formal Michael‐type addition of alkynyl imines with the key alkylgold intermediates derived in situ from *N*‐propargylamides is accomplished under gold‐complex and chiral quinine‐derived squaramide (QN‐SQA) synergetic catalysis. Control experiments and the density functional theory (DFT) calculations indicated that this cascade reaction involves a Mannich‐type addition and stepwise [3,3]‐σ rearrangement sequence, leading to the fully substituted α‐amino allenoates, which are elusive and take multi‐step to prepare with other methods, in high yields and excellent enantioselectivity.

## Graphical abstract

An asymmetric formal Michael‐type addition of alkynyl imines with the key alkylgold intermediates derived in situ from N‐propargylamides is accomplished. This cascade reaction involves a Mannich‐type addition and stepwise [3,3]‐σ rearrangement sequence under gold‐complex and chiral quinine‐derived squaramide (QN‐SQA) synergetic catalysis, leading to the fully substituted α‐amino allenoates in high yields and excellent enantioselectivity.

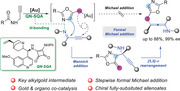

## 1 Introduction

**Scheme 1:**
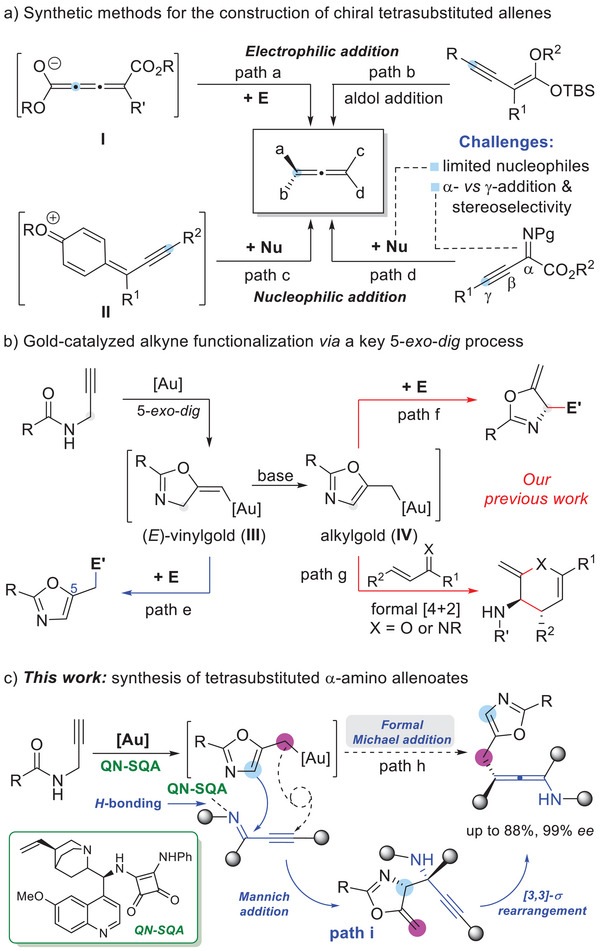
Catalytic alkyne functionalization and construction of chiral tetrasubstituted allenoates.

Axially chiral allene is one of the pivotal structural moieties in synthetic chemistry (For reviews see)^[^ ^1^ ^]^ and medicinal chemistry (For reviews see).^[^ ^2^ ^]^ Especially, axially chiral tetrasubstituted allenes are versatile reagents, which can be used as chiral building blocks for the synthesis of natural products and bioactive molecules.^[^ ^3^ ^]^ During the past decade, a variety of catalytic approaches have been reported for construction of enantioenriched tetrasubstituted allenes, including electrophilic addition using in situ formed cumulenolates **I** (**Scheme** **1**a, path a)^[^ ^4^ ^]^ or presynthesized alkynyl silyl enol ethers (Scheme 1a, path b)^[^ ^5^ ^]^ as donors, and nucleophilic addition with in situ generated *para*‐quinone methide intermediates **II** (Scheme 1a, path c)^[^ ^6^ ^]^ or readily available alkynyl ketimines serving as acceptors (Scheme 1a, path d).^[^ ^7^, ^8^, ^9^, ^10^, ^11^ ^]^ Nevertheless, most of these methods are invalid for the synthesis of chiral *α*‐amino allenoates. In this context, Wang and co‐workers first reported an enantioselective γ‐addition of 1‐alkynyl ketimines using 2,3‐disubstituted indoles as nucleophiles, which provides a practical method for the direct construction of chiral tetrasubstituted *α*‐amino allenoates with excellent enantioselectivities (Scheme 1a, path d, Nu = 2,3‐disubstituted indoles).^[^ ^7^ ^]^ However, only few catalytic asymmetric examples have been disclosed after this work by using isoxazolones,^[^ ^8^ ^]^ pyrroloisoquinolines,^[^ ^9^ ^]^ 2‐naphthols,^[^ ^10^ ^]^ or cyanoacetates^[^ ^11^ ^]^ as corresponding nucleophiles, which seriously limited the synthetic applications of this reactions. Moreover, the control of regioselectivity (α‐ vs γ‐addition) and stereoselectivity is the other main challenge in this area.^[^ ^12^ ^]^ Therefore, the development of effective and novel synthetic strategies for the assembly of fully substituted α‐amino allenoate frameworks with readily accessable materails under mild conditions would certainly be in demand.

Catalytic alkyne functionalzition via homogeneous gold catalysis has emerged as one of the most powerful tools for the synthesis of complex natural products and bioactive molecules.^[^ ^13^ ^]^ In this context, the key (*E*)‐vinylgold intermediates (**III**),^[^ ^14^ ^]^ which is derived from *N*‐propargylamides via a gold catalyzed 5‐*exo*‐*dig* cyclization process, has been widely used for the synthesis of oxazoles with diverse functional groups on the 5‐position (Scheme 1b, path e).^[^ ^15^, ^16^ ^]^ Recently, our group has realized an asymmetric interception reaction through the alkylgold intermediate (**IV**), which is derived from vinylgold species **III** through an aromatization driven *H*‐shift process with the assistance of an organocatalyst chiral quinine‐derived squaramide (QN‐SQA), including asymmetic Mannich or Aldol reaction on the original propargyl site (Scheme 1b, path f),^[^ ^17^ ^]^ and stepwise [4+2] annulation with α,β‐unsaturated imines/ketones (Scheme 1b, path g).^[^ ^18^ ^]^ Inspired by these achievements, and as a continuation of our research program on catalytic alkyne transformations (For reviews see)^[^ ^19^ ^]^ via interception of reactive in situ formed intermediates,^[^ ^20^ ^]^ we envisioned that a Michael‐type addition of alkynyl imines with the alkylgold species (**IV**) might be enabled for the construction of tetrasubstituted amino allenoates (Scheme 1c, path h). Herein, we report a regio‐ and stereoselective stepwise cascade reaction of *N*‐propargylamides with alkynyl imines under synergetic catalysis by the combination of a gold‐complex and a chiral quinine‐derived squaramide (QN‐SQA), leading to the chiral fully substituted α‐amino allenoates in good to high yields and excellent enantioselectivity (Scheme 1c, path i). Mechanistic studies and density functional theory (DFT) calculations indicate that this formal Michael‐type addition reaction involves a Mannich‐type addition of the in situ formed alkylgold intermediate (**IV**) with alkynyl imine, followed by a stepwise[3,3]‐σ rearrangement. This stepwise method provides an alternative protocol for the synthesis of α‐amino allenoate derivatives through direct γ‐addition process, which features mild conditions, high functional group compatibility, and excellent selectivity control.

## 2 Results and Discussion

Initially, the readily available *N*‐propargylamide **1a** and β, γ‐alkynyl‐α‐imino ester **2a** were chosen as model substrates, and reactions were conducted in 1,2‐dichloroethane (DCE) at 30 °C (**Table** **1**). Various bifunctional quinine‐derived squaramides **3a**‐**3e** were investigated in the presence of the gold complex Me_3_(OMe)*t*BuXPhosAuNTf_2_ in order to control stereoselectivity. All these organocatalysts promoted the transformation smoothly, producing the formal Michael‐type addition adduct **4a** in moderate to high yields with good enantioselectivity (entries 1–5). The phenyl substituted catalyst **3a** gave better results in terms of yield and stereoselectivity (entry 1: 70% yield and 95% *ee*). Further evaluation by using chiral phosphoric acids (**3f** and **3** **g**) instead of quinine‐derived squaramide catalysts gave no desired product (entries 6–7). Control experiments in the absence of either the gold catalyst or the organocatalyst resulted in no reaction or no desired product, respectively (entries 8 and 9). These results confirmed the importance of cooperative catalysis in enabling this unprecedented transformation. With the identified optimal organocatalyst **3a**, different gold complexes and solvents were screened, and no better results was obtained in terms of yield and selectivity (entries 10–16). The yield was improved to 77% when two equivalents of *N*‐propargylamide **1a** was used to form product **4a** in 95% *ee* (entry 17).

**Table 1:** Optimization of the Reaction Conditions.

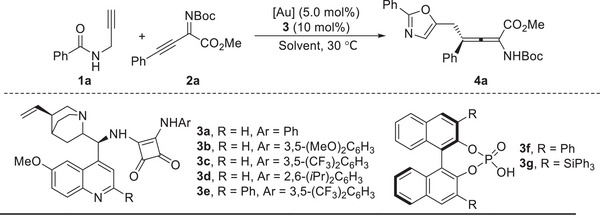				
Entry^a)^	[Au]/3	Solvent	Yields [%]^b)^	*ee* [%]^c)^
1	Me_3_(OMe)*t*BuXPhosAuNTf_2_/**3a**	DCE	70	95
2	Me_3_(OMe)*t*BuXPhosAuNTf_2_/**3b**	DCE	58	92
3	Me_3_(OMe)*t*BuXPhosAuNTf_2_/**3c**	DCE	62	96
4	Me_3_(OMe)*t*BuXPhosAuNTf_2_/**3d**	DCE	65	85
5	Me_3_(OMe)*t*BuXPhosAuNTf_2_/**3e**	DCE	48	89
6	Me_3_(OMe)*t*BuXPhosAuNTf_2_/**3f**	DCE	ND	–
7	Me_3_(OMe)*t*BuXPhosAuNTf_2_/**3g**	DCE	ND	–
8	‐/**3a**	DCE	NR	–
9	Me_3_(OMe)*t*BuXPhosAuNTf_2_/‐	DCE	ND	–
10	JohnPhos(MeCN)AuSbF_6_/**3a**	DCE	ND	–
11	PPh_3_AuNTf_2_/**3a**	DCE	26	35
12	IPrAuNTf_2_/**3a**	DCE	ND	–
13	Me_4_ *t*BuXPhosAuCl + AgNTf_2_/**3a**	DCE	16	10
14	Me_3_(OMe)*t*BuXPhosAuNTf_2_/**3a**	Toluene	ND	–
15	Me_3_(OMe)*t*BuXPhosAuNTf_2_/**3a**	TBME	NR	–
16	Me_3_(OMe)*t*BuXPhosAuNTf_2_/**3a**	THF	NR	–
17^d)^	Me_3_(OMe)*t*BuXPhosAuNTf_2_/**3a**	DCE	77	95

^a)^ The reaction was carried out on a 0.10 mmol scale: to the solution of **2a** (29.0 mg, 0.10 mmol, 1.0 equiv.), gold catalyst (5.0 mol%), and **3** (10 mol%) in solvent (1.0 mL), was added **1a** (19.1 mg, 0.12 mmol,1.2 equiv.) in the same solvent (1.0 mL) under argon atmosphere at 30 °C in 15 min, and the reaction mixture was stirring overnight under these conditions; TBME = *tert*‐butyl methyl ether; THF = tetrahydrofuran;

^b)^ Determined by ^1^H NMR of the crude reaction mixture with 1,3,5‐trimethoxybenzene as internal reference;

^c)^ The *ee* values were determined by chiral HPLC analysis;

^d)^ **1a** (31.8 mg, 0.20 mmol, 2.0 equiv.) was used. TBME = methyl *tert*‐butyl ether. THF = tetrahydrofuran. NR = no reaction. ND = not detected.

With the optimal reaction conditions established (Table 1, entry 17), we next turned our attention to explore the substrate scope for the synthesis of highly enantioenriched chiral tetrasubstituted α‐amino allenoates (Table 2). The impact of the ester part of alkynyl imines was first explored, revealing that the reaction could be applied to methyl, ethyl, and *tert*‐butyl esters in good yields with excellent enantioselectivities (**4a**‐**4c**, >73% yields with >92% *ee*); while in the case with *L*‐menthyl ester, the addition product **4d** was generated in 45% yield with >10:1 *dr* (note b: contaminated with the Mannich addition product **4d’** in 34% yield with >10:1 *dr*). The electronic and steric properties of the substituents on the aryl ring had little effect on the reaction outcomes, generating **4e**‐**4l** in 68–88% yields with 90–95% *ee*. Instead of aryl substitution, the *tert*‐butyl and cyclohexyl substituted alkynyl imines were well tolerated in this reaction, affording the corresponding products **4m** and **4n** smoothly in high yields with 96% and 95% *ee*, respectively. Terminal alkynyl imine was not compatible under current conditions, which might be due to the interaction between the terminal alkyne imine and the gold catalyst that hampers the reactivity. Moreover, both electron‐rich and election‐deficient substitutions on the different positions of aryl rings tethered to the propargylamides were found to be tolerated in this reaction, producing the allene products **6a**‐**6k** in generally good yields with 90–96% *ee*. The styryl and *tert*‐butyl group substituted *N*‐propargylamides also underwent the reaction smoothly under optimal conditions, furnishing corresponding products **6l** and **6m** in 56% and 65% yields with 90% and 99% *ee*, respectively. The absolute stereochemistry of the major enantiomer of **6f** was determined as (*S*) by X‐ray crystallography,^[^ ^21^ ^]^ and other products were assigned by analogy.

**Table 2:** Substrate scope for the synthesis of allenes^a)^ (see table footnotes).

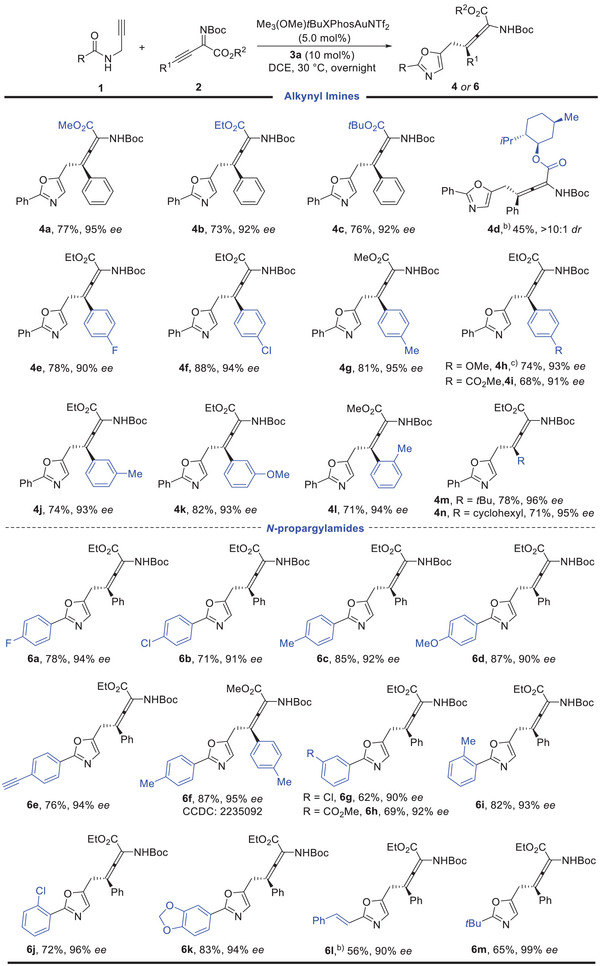

^a)^ Reaction conditions: *N*‐propargylamides **1** (0.20 mmol, 2.0 equiv.) in DCE (1.0 mL) was added to the solution of alkynyl imines **2** (0.10 mmol, 1.0 equiv.), Me_3_(OMe)*t*BuXPhosAuNTf_2_ (5.0 mg, 5.0 mol%), and **3a** (5.0 mg, 10 mol%) in DCE (1.0 mL) under an argon atmosphere at 30 °C in 15 min, and the reaction mixture was stirring overnight under these conditions;

^b)^ The Mannich addition product **4d'** was isolated in 34% yield with 10:1 *dr*;

^c)^ The loading of **3a** was increased to 15 mol%.

**Scheme 2:**
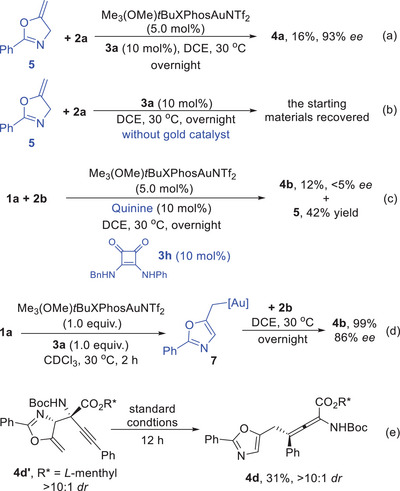
Control experiments.

To gain insight into the reaction mechanism of this formal Michael‐type addition process, several control experiments were performed (**Scheme** **2**). To explore whether this transformation went through a gold and organo cooperative catalysis^[^ ^22^ ^]^ or a relay^[^ ^23^ ^]^ catalysis, the reaction of alkylideneoxazoline **5** with **2a** was conducted under the standard conditions, forming **4a** in only 16% yield with 93% *ee* (Scheme 2a). The majority of the reactants remained intact when the reaction was performed in the absence of the gold catalyst (Scheme 2b). These results excluded the possibility of a stepwise catalytic relay via alkylideneoxazoline **5**. The desired product **4b** was formed in very low yield and compound **5** was the major product in 42% yield when a combination of organocatalyst **3** **h** and quinine were used as cocatalysts instead of quinine‐derived squaramide catalyst **3a** (Scheme 2c), which implied the critical factor of this bifunctional organocatalyst for the success of this interception process.^[^ ^17^, ^18^ ^]^ On the other hand, when equal amounts of *N*‐propargylamide **1a**, gold catalyst Me_3_(OMe)*t*BuXPhosAuNTf_2_, and organocatalyst **3a** were dissolved in CDCl_3_ at 30 °C, the gold‐complex **7** was formed after 2 h in quantitative yield, which is detected by the proton NMR spectrum of the reaction mixture. Then, alkynyl imine **2b** was added into the above solution and the allene product **4b** was isolated in 99% yield with 86% *ee* (Scheme 2d). These results suggested that the alkylgold species **7** might be the key intermediate in this reaction, and the gold complex and organocatalyst collaborate synergistically. In addtion, the Mannich addition product **4d’** could be converted to the allene product **4d** slowly under standard conditions (Scheme 2e), which implied a sequential Mannich type addtion and rearrangement relay in this reaction, instead of direct Michael type addition process.

**Figure 1:**
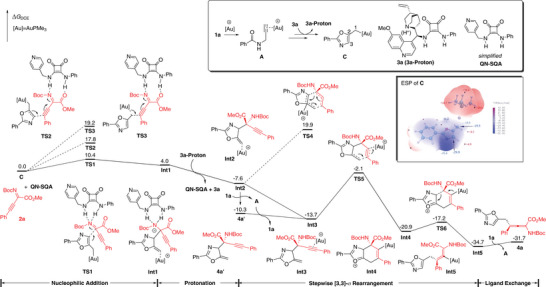
DFT calculations on the gold‐catalyzed formal Michael addition reaction.

We further carried out DFT calculations to elucidate how the present reaction takes place and which factors influence the selectivity of the key alkylgold species under this gold and quinine‐derived squaramide (QN‐SQA) synergetic catalyzed conditions. To save the computational cost without sacrificing the understandings of the mechanisms, we used ligand (PMe_3_) and simplified **QN‐SQA** instead of **3a** as co‐catalyst (as shown in **Figure** **1**) to study the reaction in racemic version. According to our previous study,^[^ ^17^, ^18^ ^]^ the key alkylgold species **C** was formed smoothly with the assistance of alkalic co‐catalyst quinine‐derived squaramide (Figure 1, in box). Then, the key alkylgold species **C** reacted with the substrate **2a** in the presence of co‐catalyst **QN‐SQA**. Considering the atomic connection of the product **4a**, it seems that the reaction site is the terminal position of alkylgold species **C** (C1 in **C**), however, DFT calculations revealed that this reaction proceeds through a nucleophilic Mannich‐type addition/protonation/[3,3]‐σ rearrangement sequence. First, **2a** is activated by **QN‐SQA** through hydrogen bonding, then C3 position in **C** undergoes nucleophilic attack at the electrophilic iminyl carbon via **TS1** to form **Int1,** requiring an activation free energy of 10.4 kcal mol^−1^. The nucleophilic attack of C3 at the less electrophilic alkynyl carbon of **2a** via **TS2** is disfavored over **TS1** by 7.4 kcal mol^−1^ (the NPA charges for iminyl carbon and alkynyl carbon are +0.162 e and +0.076 e, respectively). On the other hand, the direct nucleophilic attack of C1 at alkynyl carbon of **2a** via **TS3** is disfavored over **TS1** by 8.8 kcal mol^−1^, which could be understood by the electrostatic potential (ESP) of **C** (the electrostatic potential around C3 is more negative than C1). Subsequently, **Int1** undergoes protonation to give the intermediate **Int2**, which can further proceed to afford the Mannich addition product **4a’** through catalyst transfer with **1a**. Subsequently, a stepwise [3,3]‐σ rearrangement easily takes place with the assistance of gold catalyst.^[^ ^24^ ^]^ It should be noted that the direct [3,3]‐σ rearrangement from **Int2** via **TS4** requires an activation free energy of 27.5 kcal mol^−1^, which could not happen at room temperature and could be ruled out. On the contrary, the alkyne motif in **4a’** can be activated by gold catalyst to generate **Int3**, then intramolecular cyclization through **TS5** takes place smoothly with an activation free energy of 11.6 kcal mol^−1^. Subsequent C‐C cleavage via **TS6**, with an activation free energy of 3.7 kcal mol^−1^, affords the corresponding gold‐coordinated allene product **Int5**, which then undergoes ligand exchange with **1a** to release **4a**. These results are consistent with our experiments, indicating the reaction could occur around room temperature.

**Figure 2:**
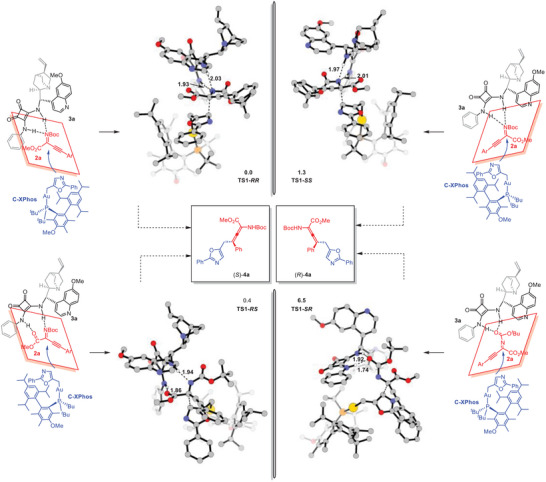
DFT calculations for understanding the enantioselectivity differentiation in real catalytic system.

The origin of stereoselectivity was further investigated. According to the free energy surface above (Figure 1), the first nucleophilic attack step via **TS1** is stereoselectivity‐determining and the subsequent [3,3]‐σ rearrangement is stereospecific. Thus, we calculated the key transition states using the real catalyst Me_3_(OMe)*t*BuXPhosAuNTf_2_ and QN‐SQA **3a** (**Figure** **2**, and see Supporting Information for details). **TS1‐*RR*** and **TS1‐*RS*** were found as the most favored transition states to generate (*S*)−**4a**, while **TS1‐*SS*** and **TS1‐*SR*** lead to (*R*)−**4a**. Both **TS1‐*RR*** and **TS1‐*RS*** are favored over **TS1‐*SS*** and **TS1‐*SR***. Based on Boltzmann distribution, the relative activation free energies of the four transition states predict a 86% *ee* for the present reaction, in agreement with the experimental results. Our calculation supported that the QN‐SQA **3a** was used as hydrogen‐bond donor with substrate **2a** (rather than the gold complex) to provide a chiral environment. Then, the *Re‐* and *Si*‐face of **2a**, which is activated by the chiral Lewis base catalyst QN‐SQA **3a**, could be recognized owing to the steric factors arising from the nucleophilic attack of the bulky gold species **C** on **2a**.

**Scheme 3:**
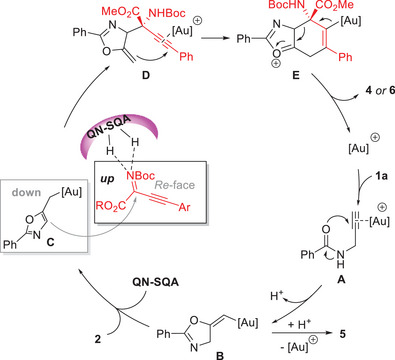
Proposed reaction mechanism.

Based on the above results and previously reported work,^[^ ^14^, ^15^, ^16^, ^17^, ^18^ ^]^ a proposed mechanistic pathway is illustrated in **Scheme** **3**. The reaction is initiated via the formation of a π‐complex **A** with the gold catalyst and *N*‐propargylamide **1a**. Then, a 5‐*exo‐dig* cyclization of activated alkyne via *anti*‐oxyauration process delivers the key (*E*)‐vinylgold intermediate **B**.^[^ ^25^ ^]^ Direct protodeauration of **B** would lead to the byproduct **5**. Whereas, with the assistance of a basic organocatalyst, the key alkylgold species **C** is generated through a *H*‐shift process driven by aromatization.^[^ ^17^, ^18^ ^]^ Then, a stereoselective Mannich‐type addition on the *Re*‐face of alkynyl imines **2** occurs to afford intermediate **D** with the assistance of QN‐SQA **3a** via *H*‐bonding. Finally, a stepwise stereospecific [3,3]‐σ rearrangement process gives the (*S*)‐products **4** and **6** via intermediate **E**.

**Scheme 4:**
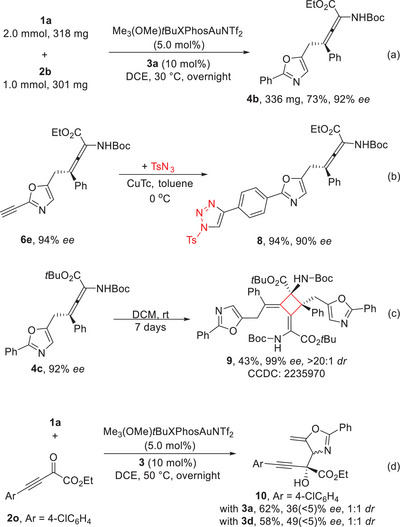
Scale‐up and synthetic transformations.

To demonstrate the synthetic utility of current method, chiral α‐amino allenoate **4a** was prepared on 1.0 mmol scale in 73% yield with 92% *ee* (**Scheme** **4**a). Further synthetic transformations with these generated products were conducted (Scheme 4b,c). The click reaction of **6e** with TsN_3_ occurred smoothly, delivering the triazole product **8** in 94% yield with 90% *ee*. Moreover, the allene **4c** could be converted to the multi‐substituted cyclobutene compound **9** via a [2+2] dimerization process. After crystallization, this product was obtained in 43% yield with >20:1 *dr* and 99% *ee*. The structure of **9** was confirmed by X‐ray diffraction analysis.^[^ ^21^ ^]^ In addition, ynone **2o**, instead of imine, only provided the Aldol addition product **10** in good yield with moderate enantioselectivity under briefly optimized conditions (Scheme 4d).

## 3 Conclusion

In conclusion, we have developed a regio‐ and enantioselective formal Michael‐type addition of *N*‐propargylamides with alkynyl imines, leading to the chiral fully substituted α‐amino allenoates in good yields and excellent enantioselectivity under gold complex and chiral quinine‐derived squaramide synergetic catalysis. Mechanistic studies and DFT calculations indicate that this cascade reaction involves a Mannich‐type addition and stepwise [3,3]‐σ rearrangement sequence through the key alkylgold intermediates derived from *N*‐propargylamides. In comparison with these reported advances for the synthesis of axially chiral allenes, this novel cascade progress provides a general protocol for the construction of tetrassubstituted α‐amino allenoates under mild conditions with broad functional group compatibility.

## 4 Experimental Section

### General Procedure for the Synthesis of α‐Amino Allenoates **4** and **6**

To a 10‐mL oven‐dried vial containing a magnetic stirring bar, alkynyl imines **2** (0.10 mmol), **3a** (5.0 mg, 10 mol%), Me_3_(OMe)*t*BuXPhosAuNTf_2_ (5.0 mg, 5.0 mol%) in DCE (1.0 mL), was added propargyl amides **1** (0.20 mmol, 2.0 equiv.) in DCE (1.0 mL) at 30 °C under argon atmosphere. The resulting reaction mixture was stirred overnight under these conditions. When the reaction completed (monitored by TLC), the solvent was evaporated *in vacuo* and the residue was purified by flash column chromatography on silica gel (hexanes/ethyl acetate = 10:1 to 5:1) to afford the pure products **4** and **6** in good to high yields.

## Conflict of Interest

The authors declare no conflict of interest.

## Author Contributions

H.Y. designed the reaction, optimized the conditions, and performed data curation, investigation, and formal analysis. X.D.X., M.B., K.C., and K.H. performed data curation, investigation, formal analysis. Y.Z. and Z.Y. performed the DFT calculation studies. X.F.X. conceived the platform, gathered funding, led the project, and wrote the original draft. All authors discussed the results and commented on the article.

## Supporting information



Supporting Information

## Data Availability

The data that support the findings of this study are available from the corresponding author upon reasonable request.
