# Supplementary material for: Enantioselective Assembly of Fully Substituted α‐Amino Allenoates Through a Mannich Addition and Stepwise [3,3]‐σ Rearrangement Sequence
Source: Adv Sci (Weinh). 2024 Nov 20;12(2):2409334. doi: 10.1002/advs.202409334 (PMC11727130; doi:10.1002/advs.202409334)
Supplement: Supplementary file 1 — Supporting Information [file ADVS-12-2409334-s001.pdf]

ADVANCED SCIENCE
Open Access

Supporting Information

for *Adv. Sci.*, DOI 10.1002/advs.202409334

Enantioselective Assembly of Fully Substituted α-Amino Allenoates Through a Mannich Addition and Stepwise [3,3]-σ Rearrangement Sequence

Haoxuan Yuan, Yi Zhou, Xiongda Xie, Ming Bao, Kewei Chen, Kemiao Hong, Zhixiang Yu* and Xinfang Xu**

SUPPORTING INFORMATION

Enantioselective Assembly of Fully Substituted α-Amino Allenoates through A Mannich Addition and Stepwise [3,3]-σ Rearrangement Sequence

Haoxuan Yuan,[1,+] Yi Zhou,[2,+] Xiongda Xie,[1] Ming Bao,[3,*] Kewei Chen,[1] Kemiao Hong,[3] Zhixiang Yu,[2,*] and Xinfang Xu[3,4,*]

[1]School of Pharmaceutical Sciences, Sun Yat-sen University, Guangzhou 510006, China.

[2]Beijing National Laboratory for Molecular Sciences (BNLMS), Key Laboratory of Bioorganic Chemistry and Molecular Engineering of Ministry of Education, College of Chemistry, Peking University, Beijing 100871, China.

[3]School of Chemistry and Chemical Engineering, Zhejiang Sci-Tech University, Hangzhou 310018, China.

[4]School of Chemistry and Chemical Engineering, Henan Normal University, Xinxiang 453007, China.

[+] These authors contributed equally: Haoxuan Yuan, Yi Zhou

Emails: bm2309@126.com; yuzx@pku.edu.cn; xuxinfang@zstu.edu.cn

Table of Contents

1. General Information **S2**

2. General Procedure for the Preparation of Alkynyl Imines 2 **S3-S4**

3. General Procedure for the Synthesis of α-Amino Allenoates 4 and 6 **S4-S21**

4. Control Experiments **S21-S24**

5. General Procedure for Scale Up **S24**

6. Synthetic Transformations **S25-S27**

7. NMR Spectra for 4, 6, 8, 9 and 10 **S28-S60**

8. HPLC Analysis Figures for 4, 6, 8, 9 and 10 **S61-S90**

9. Single-Crystal X-ray Diffraction of 6f, and 9 **S91-S92**

10. Computational Studies **S93-S147**

11. References **S147-S148**

General Information:

All reactions were carried out in oven-dried glassware. Solvents were dried following the standard methods. Flash column chromatography was performed using silica gel (300-400 mesh). Analytical thin-layer chromatography was performed using glass plates pre-coated with 200-300 mesh silica gel impregnated with a fluorescent indicator (254 nm). ^{1}H NMR and ^{13}C NMR spectra were recorded in $CDCl_3$ on a 400 MHz and 500 MHz spectrometer; chemical shifts were reported in ppm with the solvent signal as reference, and coupling constants (*J*) were given in Hertz. The peak information was described as: br = broad, s = singlet, d = doublet, t = triplet, q = quartet, m = multiplet, comp = composite. Enantioselectivity was determined on HPLC using Chiralpak IA, IA-3, IC, AD-H, and OD-H column. High-resolution mass spectra (HRMS) were recorded on a commercial apparatus (ESI or CI Source). Gold complexes were purchased from Laajoo, and used directly without additional treatment. Starting materials **1**[1] and **2**[2] were synthesized according to previously published procedure and had physical and spectral properties identical to those earlier reported.

General Procedure for the Preparation of Alkynyl Imines 2

Propargyl amides **2** were prepared were prepared according to literature procedures.[2] The compounds **2a-2c**, **2e-2h**, **2j** and **2l** are known compounds and had physical and spectral properties identical to those earlier reported.[2]

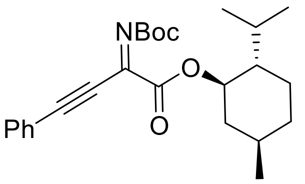

(1*S*,2*R*,5*S*)-2-Isopropyl-5-methylcyclohexyl (*Z*)-2-((*tert*-butoxycarbonyl)imino)-4-phenylbut-3-ynoate (2d). Yellow oil; ^{1}H NMR (500 MHz, $CDCl_3$) (δ, ppm) 7.54 – 7.50 (m, 2H), 7.47 – 7.44 (m, 1H), 7.41 – 7.37 (m, 2H), 4.95 – 4.86 (m, 1H), 2.11 (d, *J* = 11.9 Hz, 1H), 2.03 – 1.97 (m, 1H), 1.76 – 1.68 (m, 3H), 1.58 (s, 9H), 1.19 – 1.06 (m, 3H), 0.95 – 0.91 (m, 7H), 0.80 (d, *J* = 6.9 Hz, 3H); ^{13}C NMR (125 MHz, DMSO) (δ, ppm) 160.8, 160.3, 145.4, 132.9, 131.1, 128.8, 120.2, 101.1, 84.2, 81.3, 78.0, 48.1, 40.4, 34.2, 31.6, 28.2, 26.3, 23.5, 22.1, 20.9, 16.4.; HRMS (TOF MS ESI$^+$) calculated for $C_{25}H_{33}NO_4Na$ $[M+Na]^+$: 434.2302, found 434.2305.

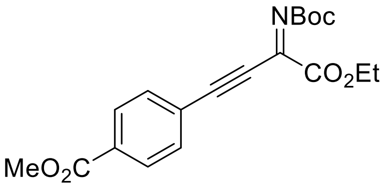

Methyl (*E*)-4-(3-((*tert*-butoxycarbonyl)imino)-4-ethoxy-4-oxobut-1-yn-1-yl) benzoate (2i) Yellow oil; ^{1}H NMR (500 MHz, $CDCl_3$) (δ, ppm) 8.05 (d, *J* = 8.3 Hz, 2H), 7.60 (d, *J* = 8.2 Hz, 2H), 4.42 (q, *J* = 7.1 Hz, 2H), 3.93 (s, 3H), 1.56 (s, 9H), 1.40 (t, *J* = 7.1 Hz, 3H); ^{13}C NMR (125 MHz, $CDCl_3$) (δ, ppm) 166.1, 161.1, 159.8, 144.7, 132.8, 132.1, 129.8, 124.4, 99.6, 84.5, 82.7, 63.6, 52.6, 28.2, 14.2; HRMS (TOF MS ESI$^+$) calculated for $C_{19}H_{21}NO_6Na$ $[M+Na]^+$: 382.1261, found 382.1258.

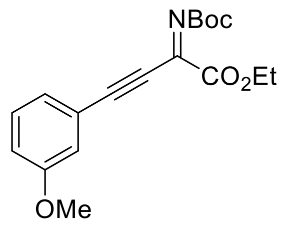

Ethyl (*E*)-2-((*tert*-butoxycarbonyl)imino)-4-(3-methoxyphenyl)but-3-ynoate (2k). Yellow oil; ^{1}H NMR (500 MHz, $CDCl_3$) (δ, ppm) 7.29 (t, *J* = 8.0 Hz, 1H), 7.14 (d, *J* = 7.6 Hz, 1H), 7.06 – 6.98 (m, 2H), 4.41 (q, *J* = 7.1 Hz, 2H), 3.80 (s, 3H), 1.58 (s, 9H), 1.40 (t, *J* = 7.1 Hz, 3H); ^{13}C NMR (125 MHz, $CDCl_3$) (δ, ppm) 161.3, 160.0, 159.5, 144.9, 129.9, 125.5, 121.0, 117.9, 117.4, 101.3, 84.3, 80.8, 63.5, 55.5, 28.2, 14.2; HRMS (TOF MS ESI$^+$) calculated for $C_{18}H_{21}NO_5Na$ [M+Na]$^+$: 354.1312, found 354.1315.

General Procedure for the Synthesis of α-Amino Allenoates 4 and 6

To a 10-mL oven-dried vial containing a magnetic stirring bar, alkynyl imines **2** (0.10 mmol), **3a** (5.0 mg, 10 mol%), Me_3(OMe)*t*BuXPhosAuNTf_2 (5.0 mg, 5.0 mol%) in DCE (1.0 mL), was added propargyl amides **1** (0.20 mmol, 2.0 equiv.) in DCE (1.0 mL) at 30 °C under argon atmosphere. The resulting reaction mixture was stirred overnight under these conditions. When the reaction was completed (monitored by TLC), the solvent was evaporated *in vacuo* and the residue was purified by flash column chromatography on silica gel (hexanes/ethyl acetate = 10:1 to 5:1) to afford the pure products **4** and **6** in good to high yields.

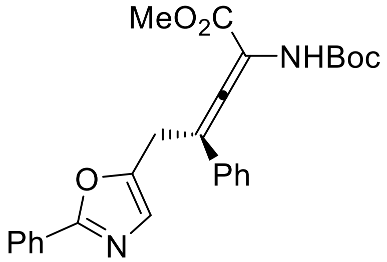

Methyl (*S*)-2-((*tert*-butoxycarbonyl)amino)-4-phenyl-5-(2-phenyloxazol-5-yl) penta-2,3-dienoate (4a) Yellow oil, 34.3 mg, 77% yield, 95% *ee*, $[\alpha]_D^{20}$ = 24.24° (c = 0.066, DCM); ^{1}H NMR (400 MHz, $CDCl_3$) (δ, ppm) 8.00 – 7.96 (m, 2H), 7.51 – 7.48 (m, 2H), 7.43 – 7.41 (comp, 3H), 7.38 – 7.34 (m, 2H), 7.30 – 7.26 (m, 1H), 7.00 (s,

1H), 6.21 (s, 1H), 4.09 (s, 2H), 3.72 (s, 3H), 1.43 (s, 9H); ^{13}C NMR (100 MHz, $CDCl_3$) (δ, ppm) 203.1, 165.2, 161.2, 149.4, 134.8, 130.2, 128.83, 128.77, 128.3, 127.8, 127.0, 126.2, 125.9, 112.9, 104.2, 80.9, 53.3, 29.8, 28.3.; HRMS (TOF MS ESI$^+$) calculated for $C_{26}H_{27}N_2O_5$ [M+H]$^+$: 447.1914, found 447.1916; HPLC conditions for determination of enantiomeric excess: Chiral OD-H, λ = 254 nm, hexane : 2-propanol = 95:05, flow rate = 1.0 mL/min, t_{minor} = 20.5 min, t_{major} = 30.6 min.

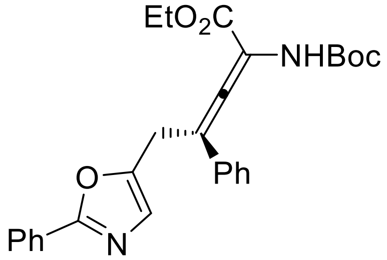

Ethyl (*S*)-2-((*tert*-butoxycarbonyl)amino)-4-phenyl-5-(2-phenyloxazol-5-yl)penta-2,3-dienoate (4b) Yellow oil, 33.6 mg, 73% yield, 92% *ee*; ^{1}H NMR (400 MHz, $CDCl_3$) (δ, ppm) 8.00 – 7.96 (m, 2H), 7.51 – 7.48 (m, 2H), 7.41 (comp, 3H), 7.35 (t, J = 7.5 Hz, 2H), 7.30 – 7.27 (m, 1H), 6.99 (s, 1H), 6.21 (s, 1H), 4.19 (q, J = 7.1 Hz, 2H), 4.09 (s, 2H), 1.43 (s, 9H), 1.19 (t, J = 7.1 Hz, 3H); ^{13}C NMR (100 MHz, $CDCl_3$) (δ, ppm) 203.1, 176.4, 164.7, 161.1, 151.8, 149.5, 130.1, 128.8, 128.7, 128.2, 127.8, 127.0, 126.2, 125.9, 112.8, 104.3, 80.8, 62.4, 29.0, 28.3, 14.3; HRMS (TOF MS ESI$^+$) calculated for $C_{27}H_{29}N_2O_5$ [M+H]$^+$: 461.2071, found 461.2076; HPLC conditions for determination of enantiomeric excess: Chiral OD-H, λ = 254 nm, hexane : 2-propanol = 95:05, flow rate = 1.0 mL/min, t_{minor} = 15.1 min, t_{major} = 20.6 min.

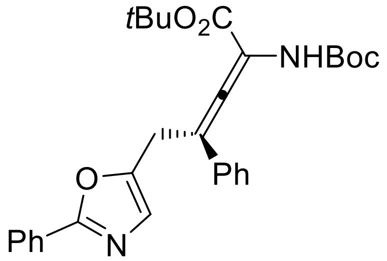

***tert*-Butyl (*S*)-2-((*tert*-butoxycarbonyl)amino)-4-phenyl-5-(2-phenyloxazol-5-yl)**

penta-2,3-dienoate (4c) Yellow oil, 37.1 mg, 76% yield, 92% *ee*; ^{1}H NMR (400 MHz, $CDCl_3$) (δ, ppm) 8.00 – 7.95 (m, 2H), 7.51 – 7.47 (m, 2H), 7.43 – 7.39 (comp, 3H), 7.36 (t, J = 7.4 Hz, 2H), 7.30 – 7.26 (m, 1H), 6.98 (s, 1H), 6.20 (s, 1H), 4.08 (s, 2H), 1.42 (s, 9H), 1.38 (s, 9H); ^{13}C NMR (100 MHz, $CDCl_3$) (δ, ppm) 202.9, 163.6, 161.0, 149.6, 135.2, 130.1, 128.8, 128.7, 128.0, 127.84, 127.80, 126.9, 126.2, 125.8, 112.3, 105.0, 83.0, 80.6, 29.0, 28.3, 28.1.; HRMS (TOF MS ESI$^+$) calculated for $C_{29}H_{32}N_2O_5Na$ [M+Na]$^+$: 511.2203, found 511.2200; HPLC conditions for determination of enantiomeric excess: Chiral OD-H, λ = 254 nm, hexane : 2-propanol = 95:05, flow rate = 1.0 mL/min, t_{minor} = 32.7 min, t_{major} = 38.4 min.

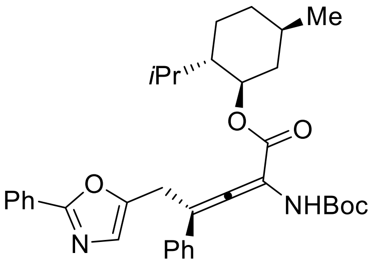

(1*S*,2*R*,5*S*)-2-Isopropyl-5-methylcyclohexyl (*S*)-2-((*tert*-butoxycarbonyl)amino)-4-phenyl-5-(2-phenyloxazol-5-yl)penta-2,3-dienoate (4d) Yellow oil, 25.7 mg, 45% yield, >10:1 *dr*; ^{1}H NMR (500 MHz, $CDCl_3$) (δ, ppm) 8.00 – 7.97 (m, 2H), 7.49 – 7.46 (m, 2H), 7.43 – 7.39 (comp, 3H), 7.34 (t, J = 7.5 Hz, 2H), 7.28 – 7.26 (m, 1H), 6.97 (s, 1H), 6.24 (s, 1H), 4.65 – 4.58 (m, 1H), 4.09 (s, 2H), 1.90 (d, J = 11.7 Hz, 1H), 1.63 – 1.60 (m, 1H), 1.43 (s, 9H), 1.19 – 1.12 (m, 2H), 0.97 – 0.91 (m, 2H), 0.90 – 0.85 (m, 2H), 0.78 (d, J = 6.4 Hz, 3H), 0.67 (d, J = 10.6 Hz, 1H), 0.61 – 0.56 (comp, 6H); ^{13}C NMR (125 MHz, $CDCl_3$) (δ, ppm) 203.0, 164.3, 161.0, 149.6, 135.0, 130.1, 128.8, 128.64, 128.59, 128.13, 128.07, 127.8, 126.8, 126.2, 125.9, 104.4, 100.1, 46.9, 40.4, 34.0, 31.4, 29.8, 28.6, 28.3, 26.2, 23.4, 22.0, 20.5, 16.3; HRMS (TOF MS ESI$^+$) calculated for $C_{35}H_{43}N_2O_5$ [M+H]$^+$: 571.3166, found 571.3169.

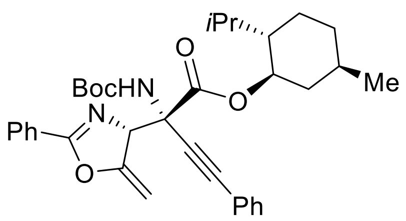

(1*R*,2*S*,5*R*)-2-Isopropyl-5-methylcyclohexyl (*R*)-2-((*tert*-butoxycarbonyl)amino)-2-((*R*)-5-methylene-2-phenyl-4,5-dihydrooxazol-4-yl)-4-phenylbut-3-ynoate (4d’) Colorless oil, 34% yield, 29.1 mg, >20:1 *dr*; ^{1}H NMR (400 MHz, $CDCl_3$) (δ, ppm) 8.03 – 7.92 (m, 2H), 7.56 – 7.50 (m, 1H), 7.49 – 7.40 (comp, 4H), 7.39 – 7.32 (comp, 3H), 5.27 – 5.21 (m, 1H), 5.08 (s, 1H), 4.98 (s, 1H), 4.95 – 4.89 (m, 1H), 3.76 (s, 1H), 2.25 (d, *J* = 12.2 Hz, 1H), 2.19 – 2.13 (m, 1H), 1.72 (d, *J* = 11.8 Hz, 2H), 1.59 – 1.49 (m, 2H), 1.26 (s, 9H), 1.22 – 1.09 (m, 1H), 0.99 – 0.96 (comp, 3H), 0.95 – 0.92 (m, 2H), 0.89 (d, *J* = 6.9 Hz, 3H), 0.81 (d, *J* = 6.9 Hz, 3H); ^{13}C NMR (100 MHz, $CDCl_3$) (δ, ppm) 169.9, 164.8, 157.2, 132.3, 131.9, 129.2, 128.7, 128.6, 128.5, 126.5, 121.9, 88.1, 86.6, 85.1, 78.3, 74.0, 73.1, 47.4, 47.2, 40.4, 34.3, 31.6, 29.9, 26.3, 23.5, 22.2, 20.9, 16.4; HRMS (TOF MS ESI$^+$) calculated for $C_{35}H_{43}N_2O_5$ $[M+H]^+$: 571.3166, found 571.3176.

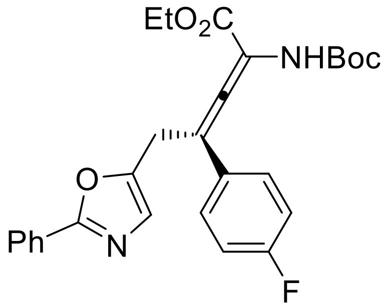

Ethyl (*S*)-2-((*tert*-butoxycarbonyl)amino)-4-(4-fluorophenyl)-5-(2-phenyloxazol-5-yl)penta-2,3-dienoate (4e) Yellow oil, 37.3 mg, 78% yield, 90% *ee*; ^{1}H NMR (400 MHz, $CDCl_3$) (δ, ppm) 8.00 – 7.94 (m, 2H), 7.49 – 7.45 (m, 2H), 7.44 – 7.40 (comp, 3H), 7.04 (t, *J* = 8.7 Hz, 2H), 6.98 (s, 1H), 6.23 (s, 1H), 4.19 (q, *J* = 7.1 Hz, 2H), 4.06 (s, 2H), 1.43 (s, 9H), 1.20 (t, *J* = 7.1 Hz, 3H); ^{13}C NMR (100 MHz, $CDCl_3$) (δ, ppm) 202.8, 164.5, 162.7 (d, *J* = 248.0 Hz), 161.2, 151.8, 149.3, 130.9 (d, *J* = 3.0 Hz), 130.2, 128.8, 128.7, 127.7, 126.2, 126.0, 115.7 (d, *J* = 21.8 Hz), 111.9, 104.3, 80.9,

62.5, 29.2, 28.3, 14.3; ^{19}F NMR (471 MHz, $CDCl_3$) (δ, ppm) -113.7; HRMS (TOF MS ESI$^+$) calculated for $C_{27}H_{27}N_2O_5NaF$ $[M+Na]^+$: 501.1769, found 501.1789; HPLC conditions for determination of enantiomeric excess: Chiral OD-H, λ = 254 nm, hexane : 2-propanol = 95:05, flow rate = 1.0 mL/min, t_{minor} = 19.8 min, t_{major} = 27.0 min.

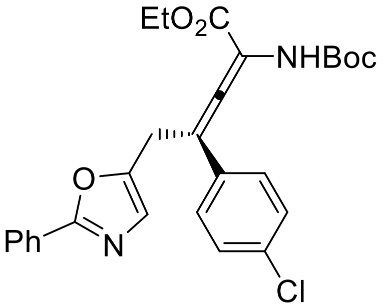

Ethyl (*S*)-2-((*tert*-butoxycarbonyl)amino)-4-(4-chlorophenyl)-5-(2-phenyloxazol-5-yl)penta-2,3-dienoate (4f) Yellow oil, 43.5 mg, 88% yield, 94% *ee*, $[\alpha]_D^{20}$ = 9.09^o (c = 0.033, DCM); ^{1}H NMR (500 MHz, $CDCl_3$) (δ, ppm) 7.99 – 7.96 (m, 2H), 7.44 – 7.41 (comp, 5H), 7.32 (d, *J* = 8.4 Hz, 2H), 6.98 (s, 1H), 6.22 (s, 1H), 4.19 (d, *J* = 7.1 Hz, 2H), 4.06 (s, 2H), 1.43 (s, 9H), 1.20 (t, *J* = 7.1 Hz, 3H); ^{13}C NMR (125 MHz, $CDCl_3$) (δ, ppm) 203.2, 164.4, 161.2, 151.8, 149.2, 134.1, 133.4, 130.2, 128.9, 128.8, 128.3, 127.7, 126.2, 126.0, 111.9, 104.5, 81.0, 62.6, 29.0, 28.3, 14.3; HRMS (TOF MS ESI$^+$) calculated for $C_{27}H_{27}ClN_2O_5Na$ $[M+Na]^+$: 517.1501, found 517.1495; HPLC conditions for determination of enantiomeric excess: Chiral OD-H, λ = 254 nm, hexane : 2-propanol = 95:05, flow rate = 1.0 mL/min, t_{minor} = 21.4 min, t_{major} = 27.2 min.

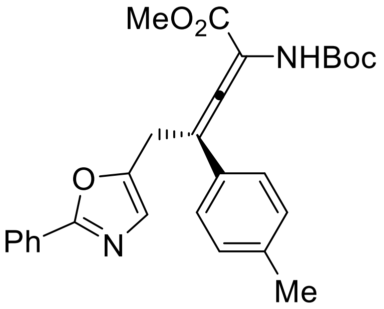

Methyl (*S*)-2-((*tert*-butoxycarbonyl)amino)-5-(2-phenyloxazol-5-yl)-4-(*p*-tolyl)

penta-2,3-dienoate (4g) Yellow oil, 37.3 mg, 81% yield, 95% *ee*, $[\alpha]_D^{20}$ = 30.30° (c = 0.066, DCM); ^{1}H NMR (500 MHz, $CDCl_3$) (δ, ppm) 8.00 – 7.96 (m, 2H), 7.45 – 7.37 (comp, 6H), 7.16 (d, *J* = 7.0 Hz, 2H), 7.00 (s, 1H), 6.22 (s, 1H), 4.07 (s, 2H), 3.71 (s, 3H), 2.34 (s, 3H), 1.43 (s, 9H); ^{13}C NMR (125 MHz, $CDCl_3$) (δ, ppm) 202.8, 165.2, 161.1, 149.5, 138.2, 131.9, 131.7, 130.1, 129.5, 128.8, 127.8, 127.0, 126.2, 125.9, 112.8, 104.0, 80.8, 53.3, 28.9, 28.3, 21.3; HRMS (TOF MS ESI$^+$) calculated for $C_{27}H_{29}N_2O_5$ $[M+H]^+$: 461.2071, found 461.2060; HPLC conditions for determination of enantiomeric excess: Chiral OD-H, λ = 254 nm, hexane : 2-propanol = 95:05, flow rate = 1.0 mL/min, t_{minor} = 16.2 min, t_{major} = 25.8 min.

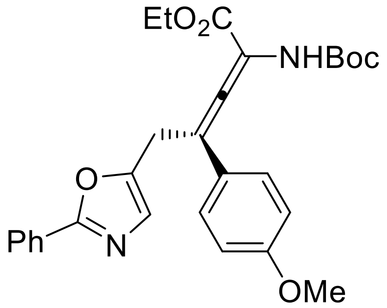

Ethyl (*S*)-2-((*tert*-butoxycarbonyl)amino)-4-(4-methoxyphenyl)-5-(2-phenyloxazol-5-yl)penta-2,3-dienoate (4h) Yellow oil, 36.3 mg, 74% yield, 93% *ee*; ^{1}H NMR (400 MHz, $CDCl_3$) (δ, ppm) 7.99 – 7.97 (m, 2H), 7.43 – 7.42 (m, 2H), 7.42 – 7.40 (comp, 3H), 6.98 (s, 1H), 6.90 – 6.87 (m, 2H), 6.21 (s, 1H), 4.20 (d, *J* = 7.1 Hz, 1H), 4.17 (d, *J* = 7.1 Hz, 1H), 4.06 (s, 2H), 3.81 (s, 3H), 1.42 (s, 9H), 1.19 (t, *J* = 7.1 Hz, 3H); ^{13}C NMR (100 MHz, $CDCl_3$) (δ, ppm) 202.4, 164.7, 161.1, 159.7, 149.7, 146.5, 130.1, 128.8, 128.3, 127.8, 127.1, 126.2, 125.9, 114.2, 112.3, 104.0, 80.7, 62.4, 55.5, 29.1, 28.4, 14.3; HRMS (TOF MS ESI$^+$) calculated for $C_{28}H_{30}N_2O_6Na$ $[M+Na]^+$: 513.1996, found 513.1987; HPLC conditions for determination of enantiomeric excess: Chiral AD-H, λ = 254 nm, hexane : 2-propanol = 95:05, flow rate = 1.0 mL/min, t_{minor} = 31.4 min, t_{major} = 48.2 min.

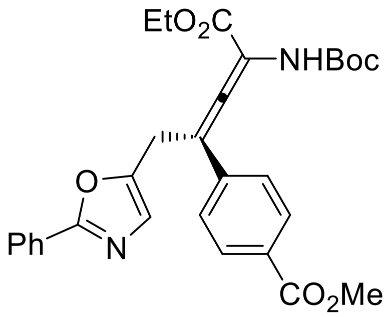

Methyl (*S*)-4-(4-((*tert*-butoxycarbonyl)amino)-5-ethoxy-5-oxo-1-(2-phenyloxazol-5-yl)penta-2,3-dien-2-yl)benzoate (4i) Yellow oil, 35.2 mg, 68% yield, 91% *ee*; ^{1}H NMR (500 MHz, $CDCl_3$) (δ, ppm) 8.04 – 8.00 (m, 2H), 8.00 – 7.95 (m, 2H), 7.56 (d, *J* = 8.3 Hz, 2H), 7.44 – 7.40 (comp, 3H), 6.99 (s, 1H), 6.25 (s, 1H), 4.20 (q, *J* = 7.0 Hz, 2H), 4.11 (s, 2H), 3.91 (s, 3H), 1.43 (s, 9H), 1.19 (t, *J* = 7.1 Hz, 3H); ^{13}C NMR (125 MHz, $CDCl_3$) (δ, ppm) 204.4, 166.9, 164.3, 161.2, 149.1, 139.5, 130.2, 130.0, 129.6, 128.83, 128.75, 127.7, 126.9, 126.2, 126.0, 112.2, 104.8, 81.1, 62.6, 52.3, 29.8, 28.3, 14.2; HRMS (TOF MS ESI^{+}) calculated for $C_{29}H_{30}N_2O_7Na$ $[M+Na]^{+}$: 541.1945, found 541.1949; HPLC conditions for determination of enantiomeric excess: Chiral IC, λ = 254 nm, hexane : 2-propanol = 80:20, flow rate = 1.0 mL/min, t_{minor} = 11.7 min, t_{major} = 13.6 min.

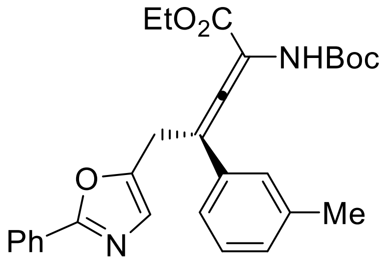

Ethyl (*S*)-2-((*tert*-butoxycarbonyl)amino)-5-(2-phenyloxazol-5-yl)-4-(*m*-tolyl) penta-2,3-dienoate (4j) Yellow oil, 35.1mg, 74% yield, 93% *ee*; ^{1}H NMR (500 MHz, $CDCl_3$) (δ, ppm) 8.01 – 7.95 (m, 2H), 7.44 – 7.40 (comp, 3H), 7.32 – 7.28 (m, 2H), 7.26 – 7.21 (m, 1H), 7.12 – 7.08 (m, 1H), 6.99 (s, 1H), 6.23 (s, 1H), 4.19 (q, *J* = 7.1 Hz, 2H), 4.08 (s, 2H), 2.35 (s, 3H), 1.43 (s, 9H), 1.20 (t, *J* = 7.1 Hz, 3H); ^{13}C NMR (125 MHz, $CDCl_3$) 203.0, 164.7, 161.1, 149.6, 138.3, 134.8, 130.1, 129.2, 129.1, 129.0, 128.8, 128.6, 127.9, 127.8, 126.2, 125.9, 124.1, 112.8, 80.8, 62.4, 29.0, 28.3, 21.7, 14.3; HRMS (TOF MS ESI+) calculated for $C_{28}H_{30}N_2O_5Na$ $[M+Na]^{+}$: 497.2047,

found 497.2044; HPLC conditions for determination of enantiomeric excess: Chiral OD-H, λ = 254 nm, hexane : 2-propanol = 95:05, flow rate = 1.0 mL/min, t_{minor} = 12.0 min, t_{major} =16.9 min.

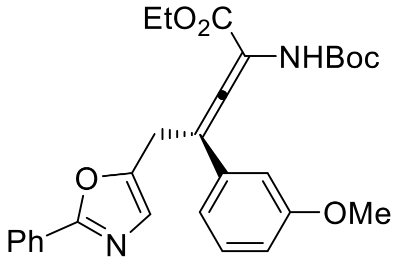

Ethyl (*S*)-2-((*tert*-butoxycarbonyl)amino)-4-(3-methoxyphenyl)-5-(2-phenyloxazol -5-yl)penta-2,3-dienoate (4k) Yellow oil, 40.2 mg, 82% yield, 93% *ee*; ^{1}H NMR (500 MHz, $CDCl_3$) (δ, ppm) 8.00 – 7.97 (m, 2H), 7.44 – 7.41 (comp, 3H), 7.30 – 7.27 (m, 1H), 7.10 – 7.08 (m, 2H), 7.00 (s, 1H), 6.85 – 6.82 (m, 1H), 6.22 (s, 1H), 4.19 (q, *J* = 7.1 Hz, 2H), 4.07 (s, 2H), 3.80 (s, 3H), 1.43 (s, 9H), 1.19 (t, *J* = 7.1 Hz, 3H); ^{13}C NMR (125 MHz, $CDCl_3$) (δ, ppm) 203.2, 164.6, 161.1, 159.8, 149.5, 136.4, 130.1, 129.7, 128.81, 128.78, 127.8, 126.2, 125.9, 120.2, 119.4, 113.7, 113.1, 112.7, 80.8, 62.5, 55.4, 29.1, 28.3, 14.3; HRMS (TOF MS ESI$^+$) calculated for $C_{28}H_{30}N_2O_6Na$ $[M+Na]^+$: 513.1996, found 513.1989; HPLC conditions for determination of enantiomeric excess: Chiral AD-H, λ = 254 nm, hexane : 2-propanol = 90:10, flow rate = 1.0 mL/min, t_{minor} = 14.9 min, t_{major} = 25.4 min.

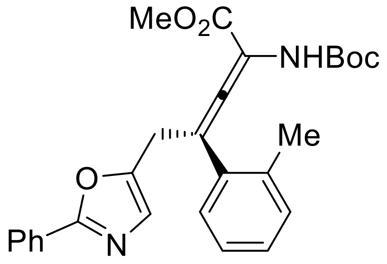

Methyl (*S*)-2-((*tert*-butoxycarbonyl)amino)-5-(2-phenyloxazol-5-yl)-4-(*o*-tolyl) penta-2,3-dienoate (4l) Yellow oil, 32.7 mg, 71% yield, 94% *ee*; ^{1}H NMR (500 MHz, $CDCl_3$) (δ, ppm) 8.02 – 7.97 (m, 2H), 7.45 – 7.40 (comp, 3H), 7.37 – 7.33 (m, 1H), 7.20 (s, 3H), 7.00 (s, 1H), 6.10 (s, 1H), 4.03 (d, *J* = 16.4 Hz, 1H), 3.90 (d, *J* = 16.4 Hz,

1H), 3.71 (s, 3H), 2.41 (s, 3H), 1.45 (s, 9H); ^{13}C NMR (125 MHz, $CDCl_3$) 198.0, 165.5, 161.2, 149.1, 136.4, 135.7, 130.8, 130.1, 128.8, 128.4, 128.1, 127.8, 126.2, 126.1, 125.9, 112.4, 102.4, 80.6, 53.1, 32.1, 28.4, 20.2; HRMS (TOF MS ESI$^+$) calculated for $C_{27}H_{29}N_2O_5$ $[M+H]^+$: 461.2071, found 461.2073; HPLC conditions for determination of enantiomeric excess: Chiral OD-H, λ = 254 nm, hexane : 2-propanol = 95:05, flow rate = 1.0 mL/min, t_{minor} = 10.9 min, t_{major} =22.9 min.

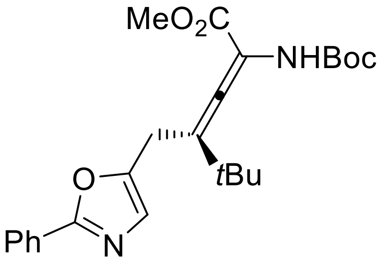

Methyl (*S*)-2-((*tert*-butoxycarbonyl)amino)-5,5-dimethyl-4-((2-phenyloxazol-5-yl) methyl)hexa-2,3-dienoate (4m) Colorless oil, 33.2 mg, 78% yield, 96% *ee*; ^{1}H NMR (500 MHz, $CDCl_3$) (δ, ppm) 8.00 – 7.90 (m, 2H), 7.49 – 7.35 (m, 3H), 6.97 (s, 1H), 6.02 (s, 1H), 3.69 (d, J = 16.7 Hz, 1H), 3.66 (s, 3H), 3.58 (d, J = 16.7 Hz, 1H), 1.43 (s, 9H), 1.19 (s, 9H); ^{13}C NMR (125 MHz, $CDCl_3$) 198.6, 166.0, 160.9, 150.4, 130.0, 128.8, 127.9, 126.1, 125.4, 121.1, 103.3, 80.2, 52.9, 36.0, 28.8, 28.4, 26.1; HRMS (TOF MS ESI$^+$) calculated for $C_{24}H_{31}N_2O_5$ $[M+H]^+$: 427.2227, found 427.2232; HPLC conditions for determination of enantiomeric excess: Chiral OD-H, λ = 254 nm, hexane : 2-propanol = 90:10, flow rate = 1.0 mL/min, t_{minor} = 6.0 min, t_{major} =10.2 min.

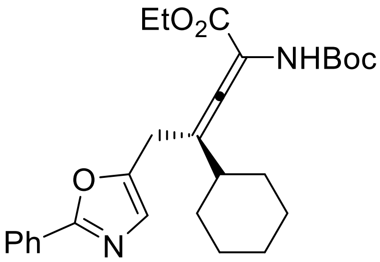

Ethyl

(*S*)-2-((*tert*-butoxycarbonyl)amino)-4-cyclohexyl-5-(2-phenyloxazol-5-yl)penta-2,

3-dienoate (4n) Colorless oil, 33.1 mg, 71% yield, 95% *ee*; ^{1}H NMR (500 MHz, $CDCl_3$) (δ, ppm) 8.03 – 7.95 (m, 2H), 7.47 – 7.35 (comp, 3H), 7.00 (s, 1H), 6.11 (s, 1H), 4.27 – 4.17 (m, 1H), 4.15 – 4.06 (m, 1H), 3.62 (q, *J* = 7.1 Hz, 2H), 2.07 (t, *J* = 10.6 Hz, 1H), 1.91 (dd, *J* = 25.2, 11.8 Hz, 2H), 1.79 – 1.70 (m, 2H), 1.69 – 1.63 (m, 1H), 1.43 (s, 9H), 1.32 – 1.24 (comp, 5H), 1.20 (t, *J* = 7.1 Hz, 3H); ^{13}C NMR (125 MHz, $CDCl_3$) 198.8, 165.5, 161.0, 150.1, 130.2, 129.2, 128.8, 127.8, 126.2, 125.2, 117.1, 103.3, 62.0, 42.0, 31.7, 29.0, 28.4, 26.30, 26.26, 26.2, 14.3; HRMS (TOF MS ESI$^+$) calculated for $C_{27}H_{35}N_2O_5$ [M+H]$^+$: 467.2540, found 467.2533; HPLC conditions for determination of enantiomeric excess: Chiral OD-H, λ = 254 nm, hexane : 2-propanol = 90:10, flow rate = 1.0 mL/min, t_{minor} = 5.3 min, t_{major} =7.1 min.

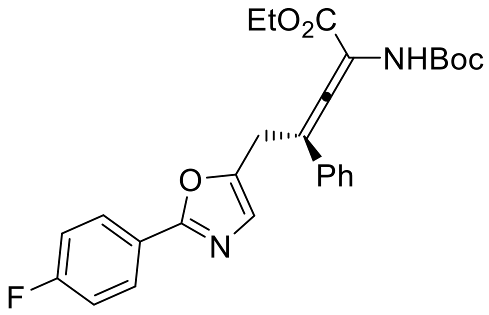

Ethyl (*S*)-2-((*tert*-butoxycarbonyl)amino)-5-(2-(4-fluorophenyl)oxazol-5-yl)-4-phenylpenta-2,3-dienoate (6a) Yellow oil, 37.3 mg, 78% yield, 94% *ee*; ^{1}H NMR (400 MHz, $CDCl_3$) (δ, ppm) 8.01 – 7.94 (m, 2H), 7.53 – 7.44 (m, 2H), 7.39 – 7.33 (m, 2H), 7.30 – 7.27 (m, 1H), 7.14 – 7.09 (m, 2H), 7.01 – 6.93 (m, 1H), 6.22 (s, 1H), 4.19 (q, *J* = 6.1 Hz, 2H), 4.08 (s, 2H), 1.43 (t, 9H), 1.21 (t, *J* = 6.1 Hz, 3H); ^{13}C NMR (100 MHz, $CDCl_3$) (δ, ppm) 203.1, 164.8, 164.0 (d, *J* = 250.0 Hz), 160.3, 149.6, 134.8, 130.2, 128.8, 128.3, 128.2, 127.0, 125.9, 124.2 (d, *J* = 3.0 Hz), 116.6 (d, *J* = 22.5 Hz), 116.0 (d, *J* = 22.1 Hz), 112.7, 62.5, 29.8, 29.0, 28.3, 14.3; ^{19}F NMR (376 MHz, $CDCl_3$) (δ, ppm) -105.7; HRMS (TOF MS ESI$^+$) calculated for $C_{27}H_{27}FN_2O_5Na$ [M+Na]$^+$: 501.1796, found 501.1784; HPLC conditions for determination of enantiomeric excess: Chiral OD-H, λ = 254 nm, hexane : 2-propanol = 95:05, flow rate = 1.0 mL/min, t_{minor} = 12.0 min, t_{major} = 16.1 min.

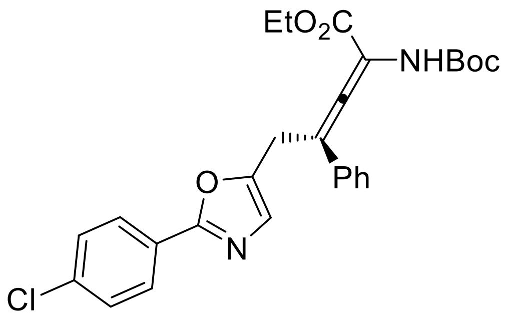

Ethyl (*S*)-2-((*tert*-butoxycarbonyl)amino)-5-(2-(4-chlorophenyl)oxazol-5-yl)-4-phenylpenta-2,3-dienoate (6b) Yellow oil, 35.1 mg, 71% yield, 91% *ee*, $[\alpha]_D^{20}$ = 18.18° (c = 0.066, DCM); ^{1}H NMR (500 MHz, $CDCl_3$) (δ, ppm) 7.93 – 7.90 (m, 2H), 7.50 – 7.48 (m, 2H), 7.41 – 7.38 (m, 2H), 7.37 – 7.34 (m, 2H), 7.31 – 7.28 (m, 1H), 7.00 (s, 1H), 6.21 (s, 1H), 4.08 (s, 2H), 3.72 (s, 3H), 1.43 (s, 9H); ^{13}C NMR (125 MHz, $CDCl_3$) (δ, ppm) 203.1, 165.1, 160.2, 149.8, 136.2, 134.7, 129.2, 128.8, 128.3, 127.5, 127.0, 126.3, 126.1, 120.1, 112.9, 104.1, 80.9, 53.3, 31.6, 28.3; HRMS (TOF MS ESI$^+$) calculated for $C_{27}H_{27}ClN_2O_5Na$ $[M+Na]^+$: 517.1501, found 517.1497; HPLC conditions for determination of enantiomeric excess: Chiral OD-H, λ = 254 nm, hexane : 2-propanol = 95:05, flow rate = 1.0 mL/min, t_{minor} = 14.2 min, t_{major} = 50.0 min.

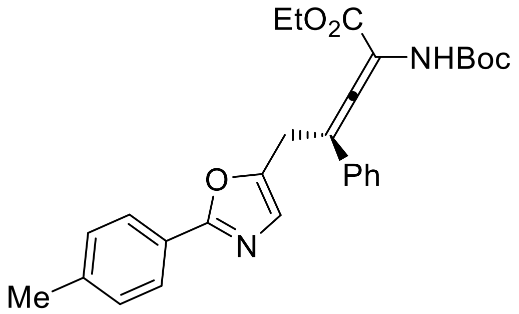

Ethyl (*S*)-2-((*tert*-butoxycarbonyl)amino)-4-phenyl-5-(2-(*p*-tolyl)oxazol-5-yl) penta-2,3-dienoate (6c) Yellow oil, 40.3 mg, 85% yield, 92% *ee*; ^{1}H NMR (500 MHz, $CDCl_3$) (δ, ppm) 7.89 – 7.85 (m, 2H), 7.52 – 7.47 (m, 2H), 7.37 – 7.33 (m, 2H), 7.31 – 7.27 (m, 1H), 7.24 – 7.21 (m, 2H), 6.97 (s, 1H), 6.22 (s, 1H), 4.19 (q, *J* = 7.1 Hz, 2H), 4.08 (s, 2H), 2.39 (s, 3H), 1.43 (s, 9H), 1.20 (t, *J* = 7.1 Hz, 3H); ^{13}C NMR (125 MHz, $CDCl_3$) (δ, ppm) 203.1, 164.7, 161.3, 156.7, 149.1, 140.4, 134.9, 129.5, 128.7, 128.2, 127.0, 126.1, 125.7, 125.1, 112.8, 106.8, 62.5, 29.8, 29.0, 28.3, 21.6, 14.3;

HRMS (TOF MS ESI+) calculated for $C_{28}H_{30}N_2O_5Na$ $[M+Na]^+$: 497.2047, found 497.2044; HPLC conditions for determination of enantiomeric excess: Chiral OD-H, λ = 254 nm, hexane : 2-propanol = 95:05, flow rate = 1.0 mL/min, t_{minor} = 14.9 min, t_{major} = 43.5 min.

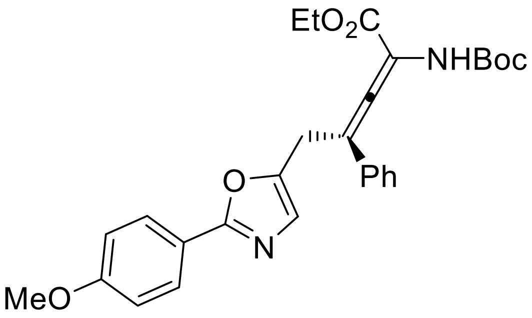

Ethyl (*S*)-2-((*tert*-butoxycarbonyl)amino)-5-(2-(4-methoxyphenyl)oxazol-5-yl)-4-phenylpenta-2,3-dienoate (6d) Yellow oil, 42.6 mg, 87% yield, 90% *ee*; ^{1}H NMR (500 MHz, $CDCl_3$) (δ, ppm) 7.93 – 7.88 (m, 2H), 7.53 – 7.47 (m, 2H), 7.38 – 7.33 (m, 2H), 7.30 – 7.27 (m, 1H), 6.96 – 6.91 (comp, 3H), 6.23 (s, 1H), 4.19 (q, *J* = 7.1 Hz, 2H), 4.07 (s, 2H), 3.84 (s, 3H), 1.43 (s, 9H), 1.19 (t, *J* = 7.1 Hz, 3H); ^{13}C NMR (125 MHz, $CDCl_3$) (δ, ppm) 203.1, 164.7, 161.2, 148.9, 134.9, 128.7, 128.2, 127.8, 127.0, 125.6, 120.7, 114.2, 112.8, 104.2, 80.8, 62.4, 55.5, 29.0, 28.3, 14.3; HRMS (TOF MS ESI+) calculated for $C_{28}H_{30}N_2O_6Na$ $[M+Na]^+$: 513.1996, found 513.2000; HPLC conditions for determination of enantiomeric excess: Chiral AD-H, λ = 254 nm, hexane : 2-propanol = 90:10, flow rate = 1.0 mL/min, t_{minor} = 27.4 min, t_{major} = 34.7 min.

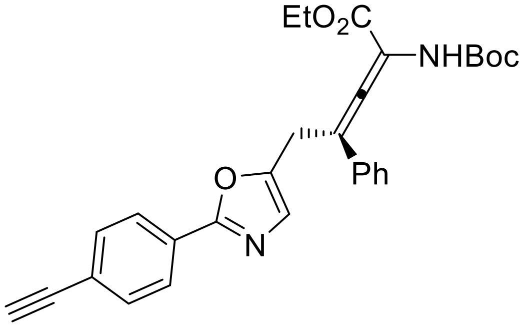

Ethyl (*S*)-2-((*tert*-butoxycarbonyl)amino)-5-(2-(4-ethynylphenyl)oxazol-5-yl)-4-phenylpenta-2,3-dienoate (6e) Yellow oil, 36.8 mg, 76% yield, 94% *ee*; ^{1}H NMR

(400 MHz, $CDCl_3$) (δ, ppm) 7.97 – 7.91 (m, 2H), 7.56 – 7.52 (m, 2H), 7.51 – 7.47 (m, 2H), 7.38 – 7.34 (m, 2H), 7.31 – 7.27 (m, 1H), 7.02 (s, 1H), 6.23 (s, 1H), 4.20 (q, J = 7.1 Hz, 2H), 4.09 (s, 2H), 3.19 (s, 1H), 1.43 (s, 9H), 1.20 (t, J = 7.1 Hz, 3H); ^{13}C NMR (100 MHz, $CDCl_3$) (δ, ppm) 203.1, 164.6, 160.4, 151.8, 150.0, 134.8, 132.6, 128.8, 128.3, 127.8, 127.0, 126.3, 126.0, 123.8, 112.7, 104.3, 83.3, 80.8, 79.2, 62.5, 29.0, 28.3, 14.3; HRMS (TOF MS ESI$^+$) calculated for $C_{29}H_{28}N_2O_5Na$ $[M+Na]^+$: 507.1890, found 507.1887; HPLC conditions for determination of enantiomeric excess: Chiral AD-H, λ = 254 nm, hexane : 2-propanol = 90:10, flow rate = 1.0 mL/min, t_{minor} = 31.2 min, t_{major} = 36.3 min.

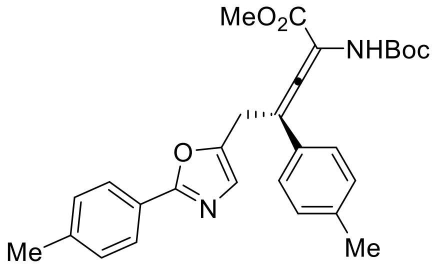

Methyl (*S*)-2-((*tert*-butoxycarbonyl)amino)-4-(*p*-tolyl)-5-(2-(*p*-tolyl)oxazol-5-yl) penta-2,3-dienoate (6f) Yellow solid, 41.2 mg, 87% yield, 95% *ee*; ^1H NMR (500 MHz, $CDCl_3$) (δ, ppm) 7.87 (d, J = 7.9 Hz, 2H), 7.38 (d, J = 7.9 Hz, 2H), 7.22 (d, J = 7.8 Hz, 2H), 7.16 (d, J = 7.8 Hz, 2H), 6.97 (s, 1H), 6.21 (s, 1H), 4.05 (s, 2H), 3.70 (s, 3H), 2.38 (s, 3H), 2.34 (s, 3H), 1.43 (s, 9H); ^{13}C NMR (125 MHz, $CDCl_3$) (δ, ppm) 202.8, 165.3, 161.3, 149.1, 140.3, 138.2, 131.8, 129.51, 129.45, 126.9, 126.1, 125.7, 125.1, 112.8, 103.9, 80.8, 53.2, 28.9, 28.2, 21.6, 21.3; HRMS (TOF MS ESI$^+$) calculated for $C_{28}H_{30}N_2O_5Na$ $[M+Na]^+$: 497.2047, found 497.2044; HPLC conditions for determination of enantiomeric excess: Chiral IC, λ = 254 nm, hexane : 2-propanol = 90:10, flow rate = 1.0 mL/min, t_{minor} = 30.3 min, t_{major} = 52.4 min.

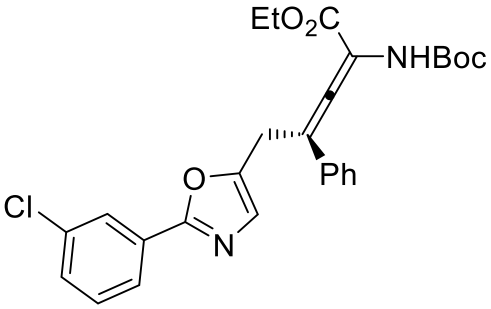

Ethyl (*S*)-2-((*tert*-butoxycarbonyl)amino)-5-(2-(3-chlorophenyl)oxazol-5-yl)-4-phenylpenta-2,3-dienoate (6g) Yellow oil, 30.6 mg, 62% yield, 90% *ee*; ^{1}H NMR (400 MHz, $CDCl_3$) (δ, ppm) 7.96 (s, 1H), 7.89 – 7.84 (m, 1H), 7.50 – 7.47 (m, 2H), 7.38 – 7.34 (comp, 4H), 7.31 – 7.28 (m, 1H), 7.01 (s, 1H), 6.22 (s, 1H), 4.21 (q, J = 7.1 Hz, 2H), 4.09 (s, 2H), 1.42 (s, 9H), 1.21 (t, J = 7.1 Hz, 3H); ^{13}C NMR (100 MHz, $CDCl_3$) (δ, ppm) 203.0, 164.6, 159.8, 150.1, 134.9, 134.8, 132.1, 130.2, 130.1, 129.4, 128.8, 128.5, 128.3, 127.0, 126.20, 126.18, 124.3, 112.7, 80.9, 62.5, 29.0, 28.3, 14.3; HRMS (TOF MS ESI$^+$) calculated for $C_{27}H_{27}ClN_2O_5Na$ [M+Na]$^+$: 517.1501, found 517.1511; HPLC conditions for determination of enantiomeric excess: Chiral OD-H, λ = 254 nm, hexane : 2-propanol = 95:05, flow rate = 1.0 mL/min, t_{minor} = 10.8 min, t_{major} = 14.5 min.

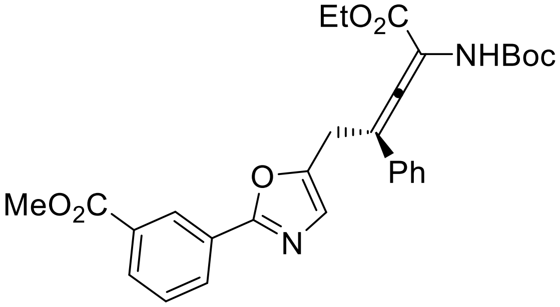

Methyl (*S*)-3-(5-(4-((*tert*-butoxycarbonyl)amino)-5-ethoxy-5-oxo-2-phenylpenta-2,3-dien-1-yl)oxazol-2-yl)benzoate (6h) Yellow oil, 35.7 mg, 69% yield, 92% *ee*; ^{1}H NMR (400 MHz, $CDCl_3$) (δ, ppm) 8.63 (s, 1H), 8.18 (d, J = 7.7 Hz, 1H), 8.09 (d, J = 7.7 Hz, 1H), 7.53 – 7.49 (comp, 3H), 7.38 – 7.34 (m, 2H), 7.30 – 7.27 (m, 1H), 7.03 (s, 1H), 6.23 (s, 1H), 4.20 (q, J = 6.8 Hz, 2H), 4.11 (s, 2H), 3.95 (s, 3H), 1.42 (s, 9H), 1.20 (d, J = 6.8 Hz, 3H); ^{13}C NMR (100 MHz, $CDCl_3$) (δ, ppm) 203.1, 166.5, 164.6, 160.1, 150.1, 134.8, 131.04, 130.98, 130.3, 129.0, 128.8, 128.2, 128.1, 127.2, 127.0,

126.2, 112.6, 104.3, 80.8, 62.5, 52.4, 29.0, 28.3, 14.3; HRMS (TOF MS ESI$^+$) calculated for $C_{29}H_{30}N_2O_7Na$ [M+Na]$^+$: 541.1945, found 541.1951; HPLC conditions for determination of enantiomeric excess: Chiral OD-H, λ = 254 nm, hexane : 2-propanol = 90:10, flow rate = 1.0 mL/min, t_{minor} = 11.0 min, t_{major} = 13.0 min.

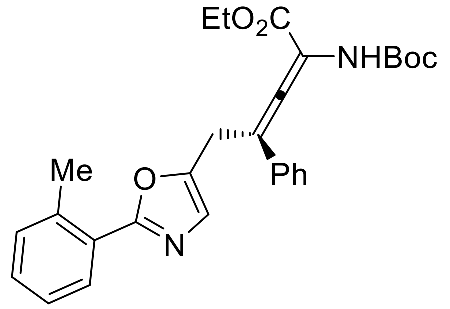

Ethyl (*S*)-2-((*tert*-butoxycarbonyl)amino)-4-phenyl-5-(2-(o-tolyl)oxazol-5-yl) penta-2,3-dienoate (6i) Yellow oil, 38.9 mg, 82% yield, 93% *ee*; ^{1}H NMR (400 MHz, $CDCl_3$) (δ, ppm) 7.92 – 7.89 (m, 1H), 7.52 – 7.49 (m, 2H), 7.37 – 7.34 (m, 2H), 7.30 – 7.28 (m, 2H), 7.26 – 7.22 (m, 2H), 7.02 (s, 1H), 6.22 (s, 1H), 4.18 (q, *J* = 7.1 Hz, 2H), 4.10 (s, 2H), 2.63 (s, 3H), 1.42 (s, 9H), 1.18 (t, *J* = 7.1 Hz, 3H); ^{13}C NMR (100 MHz, $CDCl_3$) (δ, ppm) 203.1, 164.7, 161.4, 149.0, 137.3, 134.9, 131.6, 129.8, 128.74, 128.73, 128.2, 127.1, 127.0, 126.8, 126.0, 125.6, 112.9, 104.2, 62.4, 29.8, 29.0, 28.3, 22.0, 14.2; HRMS (TOF MS ESI$^+$) calculated for $C_{28}H_{30}N_2O_5Na$ [M+Na]$^+$: 497.2047, found 497.2049; HPLC conditions for determination of enantiomeric excess: Chiral OD-H, λ = 254 nm, hexane : 2-propanol = 95:05, flow rate = 1.0 mL/min, t_{minor} = 8.6 min, t_{major} = 12.0 min.

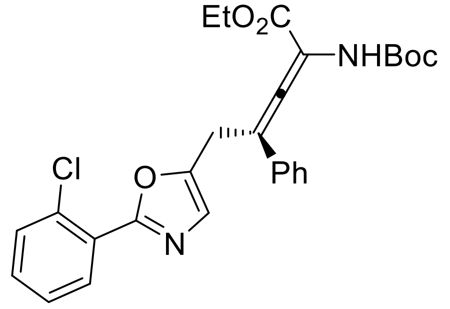

Ethyl (*S*)-2-((*tert*-butoxycarbonyl)amino)-5-(2-(2-chlorophenyl)oxazol-5-yl)-4-phenylpenta-2,3-dienoate (6j) Yellow oil, 35.6 mg, 72% yield, 96% *ee*; ^{1}H NMR

(400 MHz, $CDCl_3$) (δ, ppm) 7.94 – 7.90 (m, 1H), 7.52 – 7.48 (comp, 3H), 7.38 – 7.35 (m, 2H), 7.35 – 7.32 (m, 2H), 7.32 – 7.30 (m, 1H), 7.09 (s, 1H), 6.23 (s, 1H), 4.19 (q, J = 7.1 Hz, 2H), 4.11 (s, 2H), 1.42 (s, 9H), 1.18 (t, J = 7.1 Hz, 3H); ^{13}C NMR (100 MHz, $CDCl_3$) (δ, ppm) 203.1, 164.7, 158.8, 150.0, 134.8, 132.4 132.1, 131.3, 130.9, 130.8, 128.7, 128.2, 127.0, 126.9, 126.6, 126.0, 80.8, 62.5, 29.0, 28.3, 14.3; HRMS (TOF MS ESI$^+$) calculated for $C_{27}H_{27}ClN_2O_5Na$ [M+Na]$^+$: 517.1501, found 517.1505; HPLC conditions for determination of enantiomeric excess: Chiral OD-H, λ = 254 nm, hexane : 2-propanol = 95:05, flow rate = 1.0 mL/min, t_{minor} = 15.1 min, t_{major} = 21.2 min.

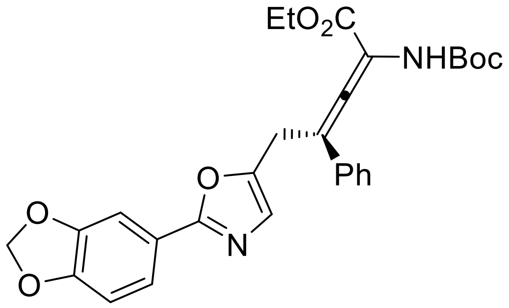

Ethyl (*S*)-5-(2-(benzo[*d*][1,3]dioxol-5-yl)oxazol-5-yl)-2-((*tert*-butoxycarbonyl) amino)-4-phenylpenta-2,3-dienoate (6k) Yellow oil, 44.8 mg, 83% yield, 94% *ee*; ^1H NMR (500 MHz, $CDCl_3$) (δ, ppm) 7.54 – 7.51 (m, 1H), 7.50 – 7.47 (m, 2H), 7.43 (s, 1H), 7.38 – 7.33 (m, 2H), 7.28 – 7.25 (m, 1H), 6.94 (s, 1H), 6.84 (d, J = 8.1 Hz, 1H), 6.23 (s, 1H), 6.01 (s, 2H), 4.20 (q, J = 6.9 Hz, 2H), 4.06 (s, 2H), 1.43 (s, 9H), 1.21 (t, J = 7.0 Hz, 3H); ^{13}C NMR (125 MHz, $CDCl_3$) (δ, ppm) 203.1, 164.7, 160.9, 149.3, 149.0, 148.1, 134.8, 128.7, 128.2, 127.0, 125.7, 122.0, 120.8, 112.7, 108.6, 106.6, 104.2, 101.6, 80.1, 62.4, 29.8, 28.9, 28.3, 14.3; HRMS (TOF MS ESI$^+$) calculated for $C_{28}H_{28}N_2O_7Na$ [M+Na]$^+$: 527.1789, found 527.1786; HPLC conditions for determination of enantiomeric excess: Chiral OD-H, λ = 254 nm, hexane : 2-propanol = 95:05, flow rate = 1.0 mL/min, t_{minor} = 23.1 min, t_{major} = 42.6min.

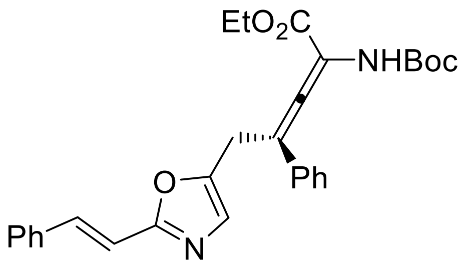

Ethyl (*S*,*E*)-2-((*tert*-butoxycarbonyl)amino)-4-phenyl-5-(2-styryloxazol-5-yl) penta-2,3-dienoate (6l) Yellow oil, 27.2 mg, 56% yield, 90% *ee*, $[\alpha]_D^{20}$ = -15.15° (c = 0.066, DCM); ^{1}H NMR (500 MHz, $CDCl_3$) (δ, ppm) 7.52 – 7.47 (comp, 5H), 7.43 – 7.41 (m, 1H), 7.39 – 7.36 (comp, 4H), 7.28 – 7.25 (m, 1H), 6.97 – 6.94 (m, 1H), 6.91 – 6.85 (m, 1H), 6.24 (s, 1H), 4.23 (q, *J* = 7.0 Hz, 2H), 4.05 (s, 2H), 1.43 (s, 9H), 1.26 (t, *J* = 7.0 Hz, 3H); ^{13}C NMR (125 MHz, $CDCl_3$) (δ, ppm) 203.2, 164.7, 161.0, 149.3, 135.8, 135.3, 134.8, 132.0, 129.2, 129.1, 129.04, 128.97, 128.7, 128.5, 128.2, 127.2, 127.0, 126.1, 114.2, 112.6, 80.9, 62.5, 29.0, 28.3, 14.3; HRMS (TOF MS ESI$^+$) calculated for $C_{29}H_{30}N_2O_5Na$ $[M+Na]^+$: 509.2047, found 509.2039; HPLC conditions for determination of enantiomeric excess: Chiral AD-H, λ = 254 nm, hexane : 2-propanol = 90:10, flow rate = 1.0 mL/min, t_{minor} = 25.8 min, t_{major} = 30.4 min.

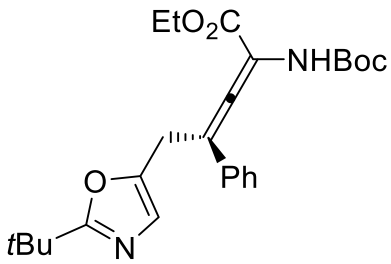

Ethyl (*S*)-2-((*tert*-butoxycarbonyl)amino)-5-(2-(*tert*-butyl)oxazol-5-yl)-4-phenylpenta-2,3-dienoate (6m) Yellow oil, 28.6 mg, 65% yield, 99% *ee*; ^{1}H NMR (400 MHz, $CDCl_3$) (δ, ppm) 7.47 – 7.44 (m, 2H), 7.32 (s, 3H), 5.85 (s, 1H), 5.11 (s, 1H), 4.91 (s, 1H), 4.79 (s, 1H), 4.22 (q, *J* = 5.8 Hz, 2H), 1.47 (s, 9H), 1.29 (t, *J* = 5.8 Hz, 3H), 1.22 (s, 9H); ^{13}C NMR (100 MHz, $CDCl_3$) (δ, ppm) 200.3, 175.1, 167.4, 157.4, 154.4, 132.2, 132.0, 128.71, 128.67, 128.5, 128.4, 122.5, 88.7, 71.9, 62.6, 33.5, 28.4, 27.5, 14.1; HRMS (TOF MS ESI$^+$) calculated for $C_{25}H_{33}N_2O_5$ $[M+H]^+$: 441.2384, found 441.2381; HPLC conditions for determination of enantiomeric

excess: Chiral OD-H, λ = 254 nm, hexane : 2-propanol = 95:05, flow rate = 1.0 mL/min, t_{minor} =3.9 min, t_{major} = 4.2 min.

Preparation of the Racemic Products 4 and 6

To a 10-mL oven-dried vial containing a magnetic stirring bar, alkynyl imines **2** (0.10 mmol), mixed organocatalyst (quinine 1.6 mg, 50% + quinidine 1.6 mg, 50%), Me_3(OMe)*t*BuXPhosAuNTf$_2$ (5.0 mg, 5.0 mol%) in DCE (1.0 mL), was added propargyl amides **1** (0.20 mmol, 2.0 equiv.) in DCE (1.0 mL) at 30 °C under argon atmosphere. The resulting reaction mixture was stirred overnight under these conditions. When the reaction was completed (monitored by TLC), the solvent was evaporated *in vacuo* and the residue was purified by flash column chromatography on silica gel (hexanes/ethyl acetate = 10:1 to 5:1) to afford the racemic pure products **4** and **6** in good to high yields.

Control Experiments

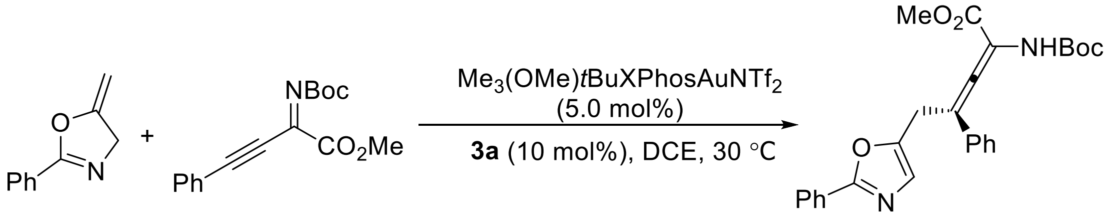

5 **2a** **4a**, 16%, 93% *ee*

To a 10-mL oven-dried vial containing a magnetic stirring bar, the solution of **2a** (30.1 mg, 0.10 mmol), **3a** (5.0 mg, 10 mol%), Me_3(OMe)*t*BuXPhosAuNTf$_2$ (5.0 mg, 5.0 mol%) in DCE (3.0 mL), was added compound **5** (31.8 mg, 0.20 mmol, 2.0 equiv.) in DCE (1.0 mL) at 30 °C under argon atmosphere, and the resulting solution was stirred overnight at 30 °C. Then the reaction mixture was subjected to proton NMR analysis in $CDCl_3$ after the solvent was evaporated and 16% of **4a** was observed (determined by proton NMR using mesitylene as internal standard. See Figure S1, the second spectrum).

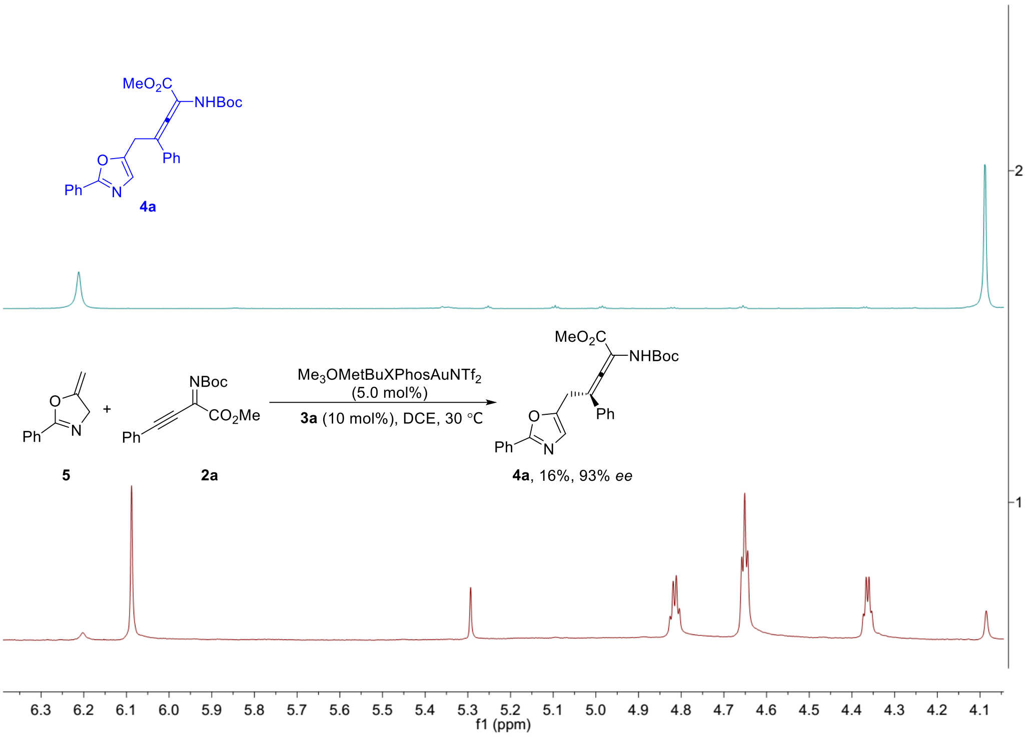

Figure S1. Proton NMR spectrum of the crude reaction mixture of **5** with **2a** under standard conditions.

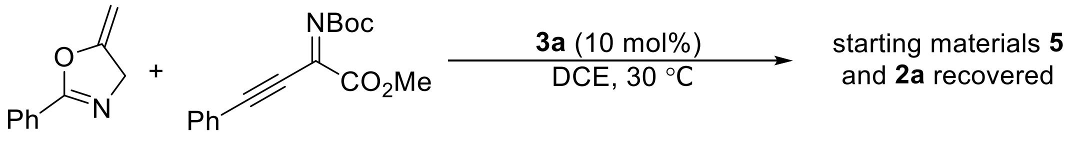

5 **2a**

To a 10-mL oven-dried vial containing a magnetic stirring bar, the solution of **2a** (30.1 mg, 0.10 mmol), **3a** (5.0 mg, 10 mol%) in DCE (1.0 mL), was added compound **5** (31.8 mg, 0.20 mmol, 2.0 equiv.) in DCE (1 mL) at 30 °C under argon atmosphere, and the resulting solution was stirred for 7 days at 30 °C. Then the reaction mixture was subjected to proton NMR analysis in $CDCl_3$ after the solvent was evaporated and no product **4a** was observed.

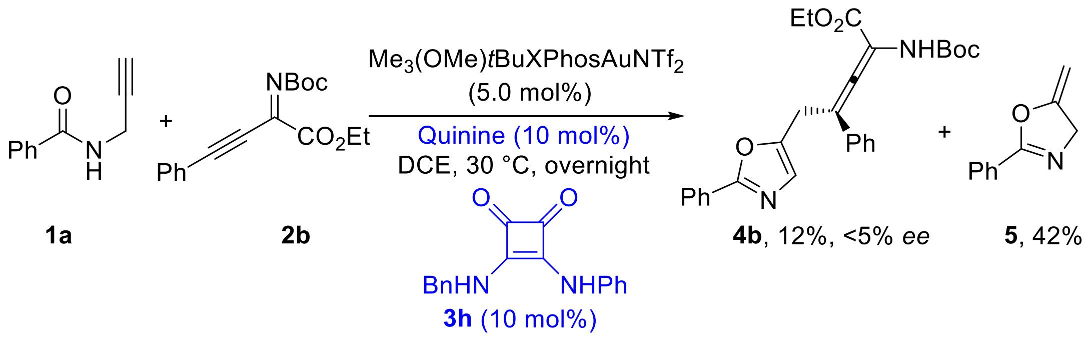

To a 10-mL oven-dried vial containing a magnetic stirring bar, the solution of **2b** (30.1 mg, 0.10 mmol), **3h** (5.0 mg, 10 mol%), quinine (3.2 mg, 10 mol%), $Me_3(OMe)$*t*BuXPhosAuNTf_2 (5.0 mg, 5.0 mol%) in DCE (1.0 mL), was added compound **1a** (31.8 mg, 0.20 mmol, 2.0 equiv.) in DCE (1.0 mL) at 30 °C under argon atmosphere. The resulting reaction mixture was stirred overnight under these conditions. Then the reaction mixture was subjected to proton NMR analysis in $CDCl_3$ after the solvent was evaporated and 12% of **4b** with <5% *ee* was observed contaminated with **5** in 42% yield (the yields were determined by proton NMR using mesitylene as internal standard).

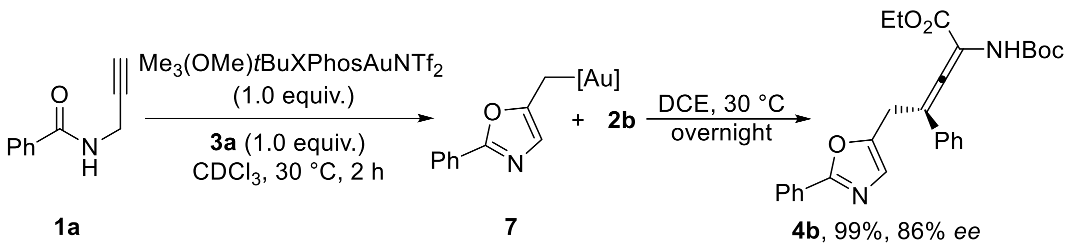

To a 10-mL oven-dried vial, the solution of **1a** (8.0 mg, 0.050 mmol), $Me_3(OMe)$*t*BuXPhosAuNTf_2 (50.0 mg, 1.0 equiv.) and **3a** (25.0 mg, 1.0 equiv.) in $CDCl_3$ (2.0 mL) was stirred for 2 h under an argon atmosphere. Then, the reaction mixture was subjected to proton NMR analysis in $CDCl_3$ after the solvent was evaporated, and alkylgold complex **7** was formed mainly. Subsequently, a solution of **2b** (15.1 mg, 1.0 equiv.) in DCE (1.0 mL) was added to the above obtained **7** in DCE (1.0 mL) at 30 °C under argon atmosphere. The resulting reaction mixture was stirred overnight under these conditions. When the reaction was completed (monitored by TLC), the crude reaction mixture was purified by flash column chromatography on silica gel (Hexanes : EtOAc = 10:1 to 5:1) to give the pure product **4b** in 99% yield

with 86% *ee*.

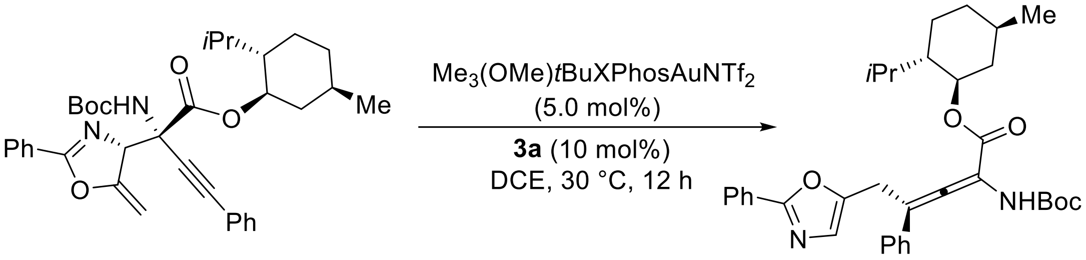

4d', >10:1 *dr* **4d**, 31%, >10:1 *dr*

To a 10-mL oven-dried vial containing a magnetic stirring bar, the solution of **3a** (1.3 mg, 10 mol%), Me_3(OMe)*t*BuXPhosAuNTf_2 (1.3 mg, 5.0 mol%) in DCE (0.50 mL), was added compound **4d'** (14.3 mg, 0.025 mmol) in DCE (0.5 mL) at 30 °C under argon atmosphere, and the resulting solution was stirred overnight at 30 °C. When the reaction was completed (monitored by TLC), the solvent was evaporated *in vacuo* and the residue was purified by flash column chromatography on silica gel (hexanes/ethyl acetate = 10:1 to 5:1) to afford 4.4 mg pure product **4d** in 31% yields with >10:1 *dr*.

General Procedure for Scale Up

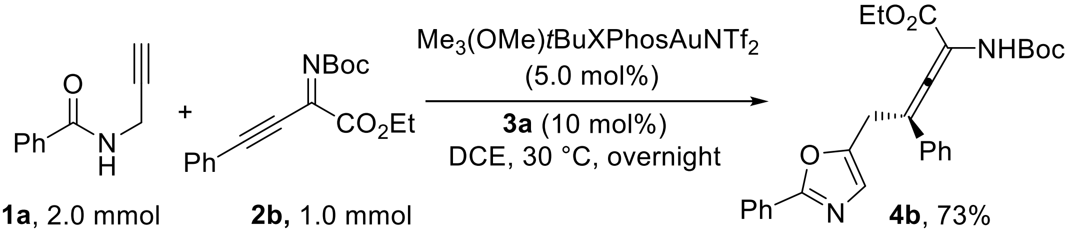

1a, 2.0 mmol 318 mg **2b,** 1.0 mmol 301 mg **4b**, 73% 336 mg, 92% *ee*

To a 50-mL oven-dried vial containing a magnetic stirring bar, alkynyl imine **2b** (301 mg, 1.0 mmol), **3a** (50 mg, 10 mol%), Me_3(OMe)*t*BuXPhosAuNTf_2 (50 mg, 5.0 mol%) in DCE (10 mL), was added propargyl amide **1a** (318 mg, 2.0 mmol) in DCE (10 mL) at 30 °C under argon atmosphere. The resulting reaction mixture was stirred overnight under these conditions. When the reaction was completed (monitored by TLC), the solvent was evaporated *in vacuo* and the residue was purified by flash column chromatography on silica gel without additional treatment (hexanes/ethyl acetate = 10:1 to 5:1) to afford 336 mg pure product **4b** in 73% yield with 92% *ee*.

Synthetic Transformations

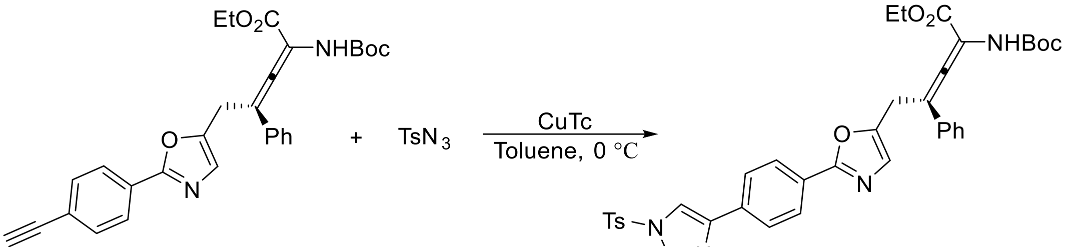

6e, 94% *ee* **8**, 94%, 90% *ee*

Synthesis of 8: To a 10-mL oven-dried vial with a magnetic stirring bar, **6e** (48.4 mg, 0.1 mmol) and CuTc (1.9 mg, 10 mol%) in toluene (1.0 mL), was added TsN_3 (30.0 mg, 0.15 mmol) at 0 °C, and the resulting reaction mixture was stirred for 1 h under these conditions. When the reaction was completed (monitored by TLC), the solvent was removed under reduced pressure, and the residue was purified by column chromatography on silica gel (Hexanes : EtOAc = 5:1 to 3:1) to afford 64.0 mg pure product **8** in 94% yield. White solid, 90% *ee*; ^{1}H NMR (400 MHz, $CDCl_3$) (δ, ppm) 8.37 (s, 1H), 8.09 – 8.00 (comp, 4H), 7.93 – 7.85 (m, 2H), 7.53 – 7.46 (m, 2H), 7.44 – 7.33 (comp, 4H), 7.32 – 7.28 (m, 1H), 7.02 (s, 1H), 6.23 (s, 1H), 4.19 (q, J = 6.7 Hz, 2H), 4.10 (s, 2H), 2.45 (s, 3H), 1.42 (s, 9H), 1.20 (t, J = 6.8 Hz, 3H); ^{13}C NMR (100 MHz, $CDCl_3$) (δ, ppm) 203.1, 164.6, 160.5, 149.9, 147.6, 146.8, 134.8, 133.1, 130.7, 130.4, 128.9, 128.8, 128.3, 128.2, 127.0, 126.8, 126.4, 126.2, 119.5, 112.7, 100.1, 80.9, 62.5, 29.0, 28.3, 22.0, 14.3; HRMS (TOF MS ESI$^+$) calculated for $C_{36}H_{35}N_5O_7Na$ $[M+Na]^+$: 704.2149, found 704.2130; HPLC conditions for determination of enantiomeric excess: Chiral IA, λ = 254 nm, hexane : 2-propanol = 70:30, flow rate = 1.0 mL/min, t_{minor} = 39.0 min, t_{major} = 64.4 min.

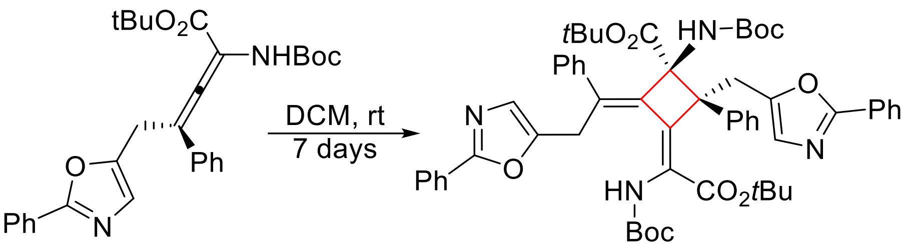

4c, 92% *ee* **9**, 43%, 99% *ee*, >20:1 *dr*

Synthesis of 9: To a 10-mL oven-dried vial with a magnetic stirring bar, the solution of **4c** (48.8 mg, 0.10 mmol) in DCM was stirred at room temperature for 7 days. Then, the solvent was removed under reduced pressure, and the residue was purified by recrystallization in mixed solvent (PE/DCM) to afford 41.5 mg pure product **9** in 43% yield as white solid. 99% *ee*; ^{1}H NMR (400 MHz, $CDCl_3$) (δ, ppm) 8.16 – 7.99 (m, 2H), 7.99 – 7.89 (m, 2H), 7.54 – 7.34 (comp, 6H), 7.21 – 7.08 (comp, 9H), 6.82 (s, 1H), 6.72 (s, 1H), 6.59 (s, 1H), 5.51 (s, 1H), 4.02 (q, J = 16.7 Hz, 2H), 3.72 (d, J = 15.4 Hz, 1H), 3.60 (d, J = 15.4 Hz, 1H), 1.57 (s, 9H), 1.38 (s, 9H), 1.12 (s, 9H), 1.00 (s, 9H); ^{13}C NMR (100 MHz, $CDCl_3$) (δ, ppm) 164.0, 161.3, 161.0, 157.2, 152.9, 151.7, 149.7, 149.1, 134.5, 130.2, 130.13, 130.06, 128.9, 128.8, 128.3, 128.2, 127.9, 127.84, 127.76, 127.0, 126.5, 126.2, 125.7, 125.6, 124.2, 84.1, 82.2, 80.9, 78.7, 70.5, 65.4, 32.6, 31.5, 28.34, 28.30, 28.2, 27.9; HRMS (TOF MS ESI^{+}) calculated for $C_{58}H_{64}N_4O_{10}Na$ [M+Na]$^{+}$: 999.4514, found 999.4505; HPLC conditions for determination of enantiomeric excess: Chiral IC, λ = 254 nm, hexane : 2-propanol = 95:05, flow rate = 1.0 mL/min, t_{minor} = 16.1 min, t_{major} = 19.7 min.

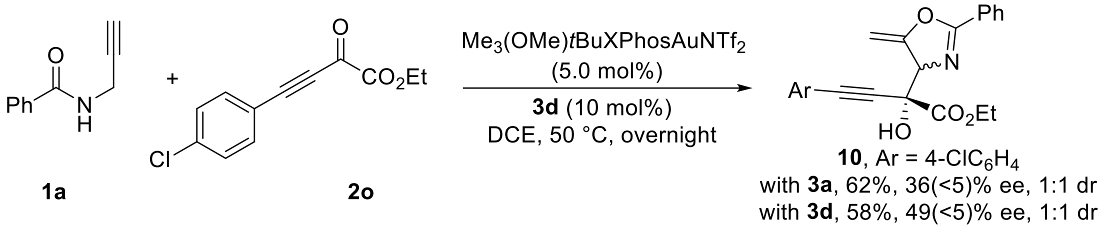

***Synthesis of 10*:** To a 10-mL oven-dried vial containing a magnetic stirring bar, ynone **2o** (23.6 mg, 0.10 mmol), **3d** (5.8 mg, 10 mol%), $Me_3(OMe)$*t*$BuXPhosAuNTf_2$ (5 mg, 5.0 mol%) in DCE (1.0 mL), was added propargyl amide **1a** (15.9 mg, 2.0 mmol) in DCE (1 mL) at 50 °C under argon atmosphere. The resulting reaction mixture was stirred overnight under these conditions. When the reaction was completed (monitored by TLC), the solvent was evaporated *in vacuo* and the residue was purified by flash column chromatography on silica gel without additional treatment (hexanes/ethyl acetate = 10:1 to 5:1) to afford 22.9 mg pure product **10** in 58% yield as a colorless oil. 49(<5)% *ee*, 1:1 *dr* (when **3a** was used instead of **3d**: 24.1 mg pure product **10** was afford in 62% yield with 36(<5)% *ee* and 1:1 *dr*). ^1H NMR (500 MHz, $CDCl_3$) (δ, ppm) 7.99 (d, J = 7.7 Hz, 2H), 7.53 (t, J = 7.3 Hz, 1H), 7.46 – 7.38 (comp, 4H), 7.32 (d, J = 8.3 Hz, 2H), 5.24 (s, 1H), 5.08 (s, 1H), 4.93 (s, 1H), 4.48 (q, J = 7.1 Hz, 2H), 1.43 (t, J = 7.1 Hz, 3H); ^{13}C NMR (125 MHz, $CDCl_3$) (δ, ppm) 170.1, 165.1, 157.0, 135.4, 133.3, 132.4, 128.9, 128.7, 128.6, 126.2, 120.2, 88.4, 85.7, 74.00, 73.2, 63.9, 29.8, 14.2; HRMS (TOF MS ESI$^+$) calculated for $C_{22}H_{19}ClNO_4$ $[M+H]^+$: 396.0997, found 396.0988; HPLC conditions for determination of enantiomeric excess: Chiral OD-H, λ = 254 nm, hexane : 2-propanol = 90:10, flow rate = 1.0 mL/min, t_{major} = 10.2 min, t_{minor} = 11.9 min. NMR signals of **10’** ^1H NMR (500 MHz, $CDCl_3$) (δ, ppm) 7.97 (d, J = 7.2 Hz, 2H), 7.46 (d, J = 6.8 Hz, 1H), 7.40 – 7.33 (m, 2H), 7.35 – 7.31 (m, 2H), 7.26 – 7.18 (m, 2H), 5.15 (s, 1H), 4.97 (s, 1H), 4.56 (s, 1H), 4.23 (q, J = 7.1 Hz, 2H), 1.37 (t, J = 7.1 Hz, 3H); ^{13}C NMR (125 MHz, $CDCl_3$) (δ, ppm) 169.4, 165.1, 156.9, 135.3, 133.4, 132.6, 128.8, 128.71, 128.69, 126.0, 120.3, 88.7, 85.8, 74.6, 74.4, 63.5, 29.8, 14.2; HPLC conditions for determination of enantiomeric excess: Chiral IC, λ = 254 nm, hexane : 2-propanol = 90:10, flow rate = 1.0 mL/min, t_{major} = 8.5 min, t_{minor} = 10.8 min.

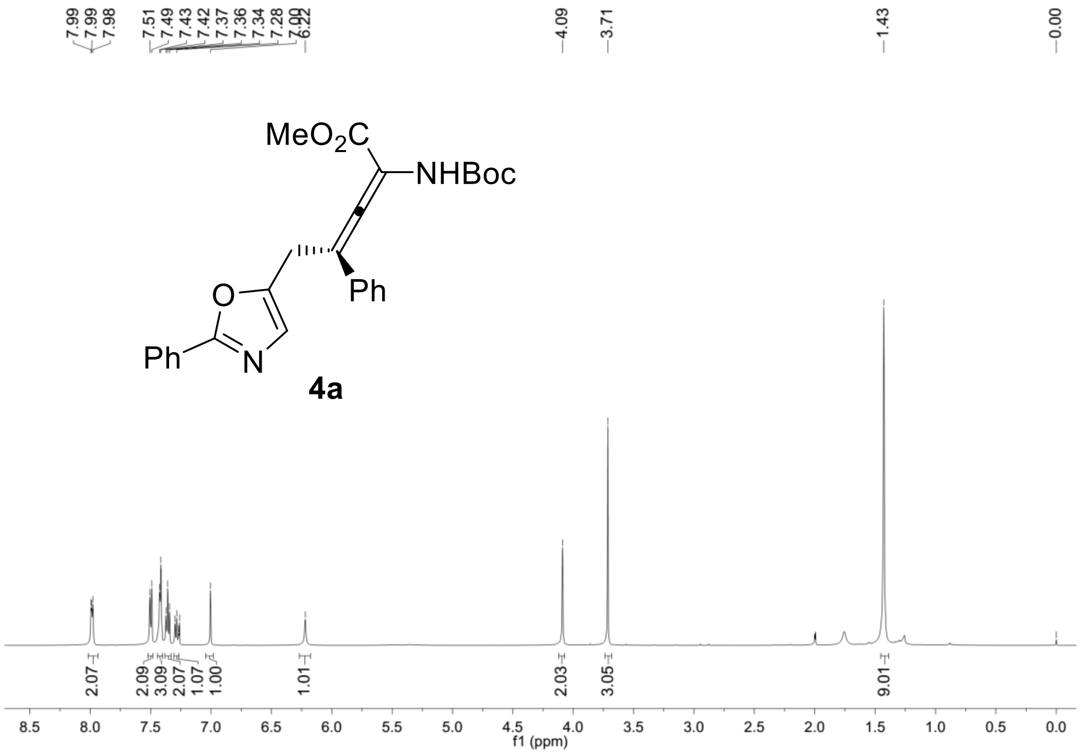
7.99
7.99
7.98
7.51
7.49
7.43
7.42
7.37
7.36
7.34
7.28
7.00
6.22
4.09
3.71
1.43
0.00
MeO2C
NHBoc
Ph
O
N
Ph
4a
2.07
2.09
3.09
2.07
1.07
1.00
1.01
2.03
3.05
9.01
f1 (ppm)

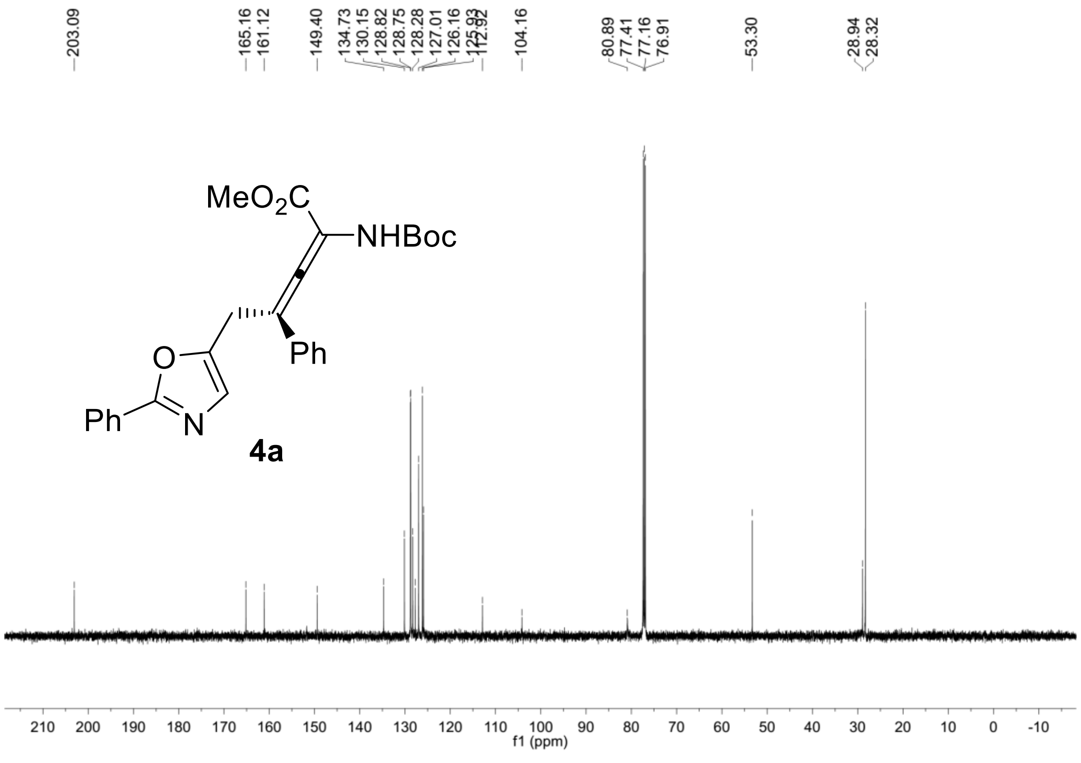
203.09
165.16
161.12
149.40
134.73
130.15
128.82
128.75
128.28
127.01
126.16
125.93
112.92
104.16
80.89
77.41
77.16
76.91
53.30
28.94
28.32
MeO2C
NHBoc
Ph
O
N
Ph
4a
f1 (ppm)

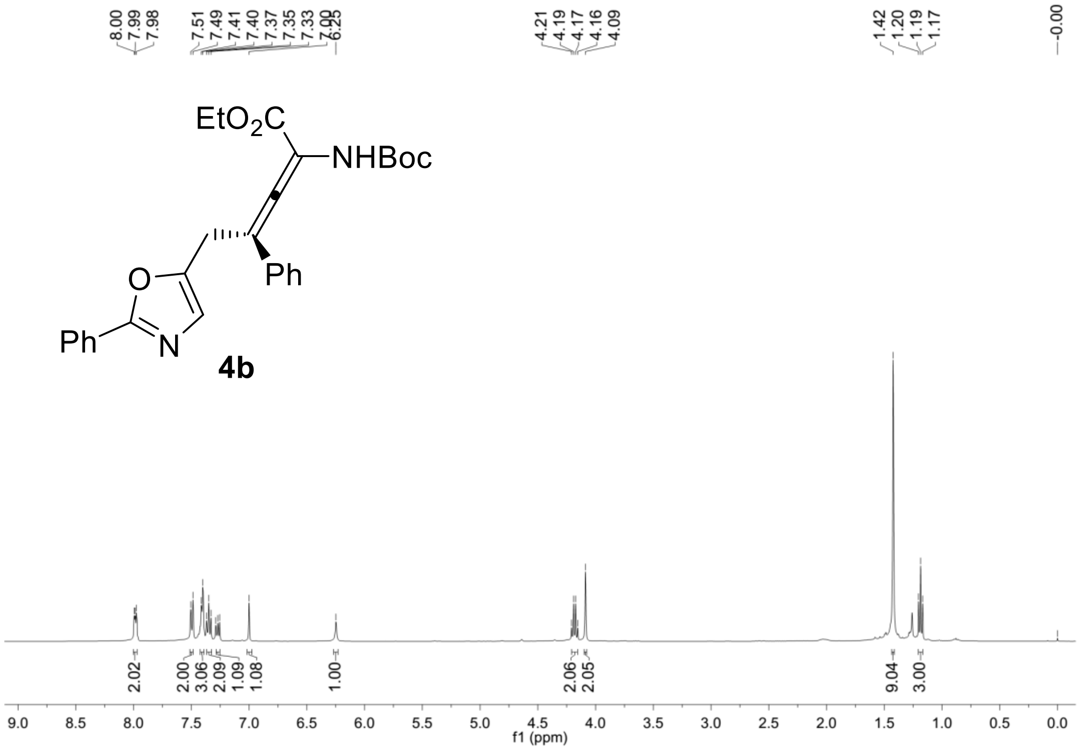
8.00
7.99
7.98
7.51
7.49
7.41
7.40
7.37
7.35
7.33
7.00
6.25
4.21
4.19
4.17
4.16
4.09
1.42
1.20
1.19
1.17
-0.00
EtO2C
NHBoc
Ph
O
N
Ph
4b
2.02
2.00
3.06
2.09
1.09
1.08
1.00
2.06
2.05
9.04
3.00
9.0 8.5 8.0 7.5 7.0 6.5 6.0 5.5 5.0 4.5 4.0 3.5 3.0 2.5 2.0 1.5 1.0 0.5 0.0
f1 (ppm)

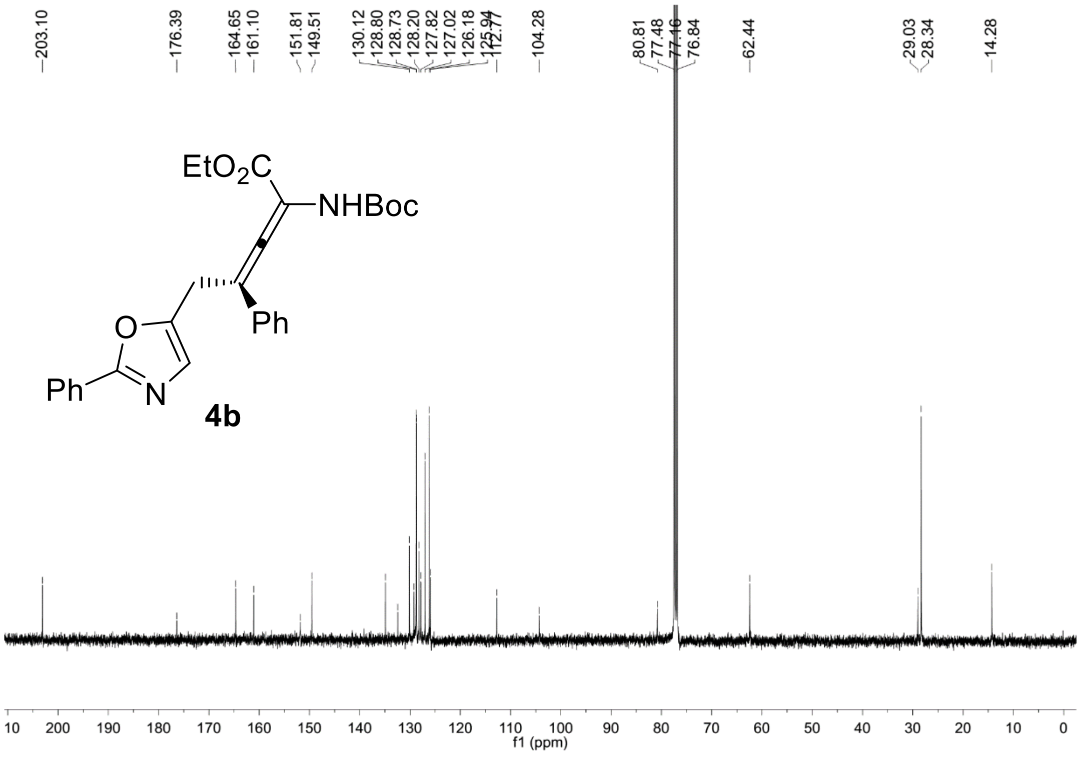
203.10
176.39
164.65
161.10
151.81
149.51
130.12
128.80
128.73
128.20
127.82
127.02
126.18
125.94
112.77
104.28
80.81
77.48
77.16
76.84
62.44
29.03
28.34
14.28
EtO2C
NHBoc
Ph
O
N
Ph
4b
10 200 190 180 170 160 150 140 130 120 110 100 90 80 70 60 50 40 30 20 10 0
f1 (ppm)

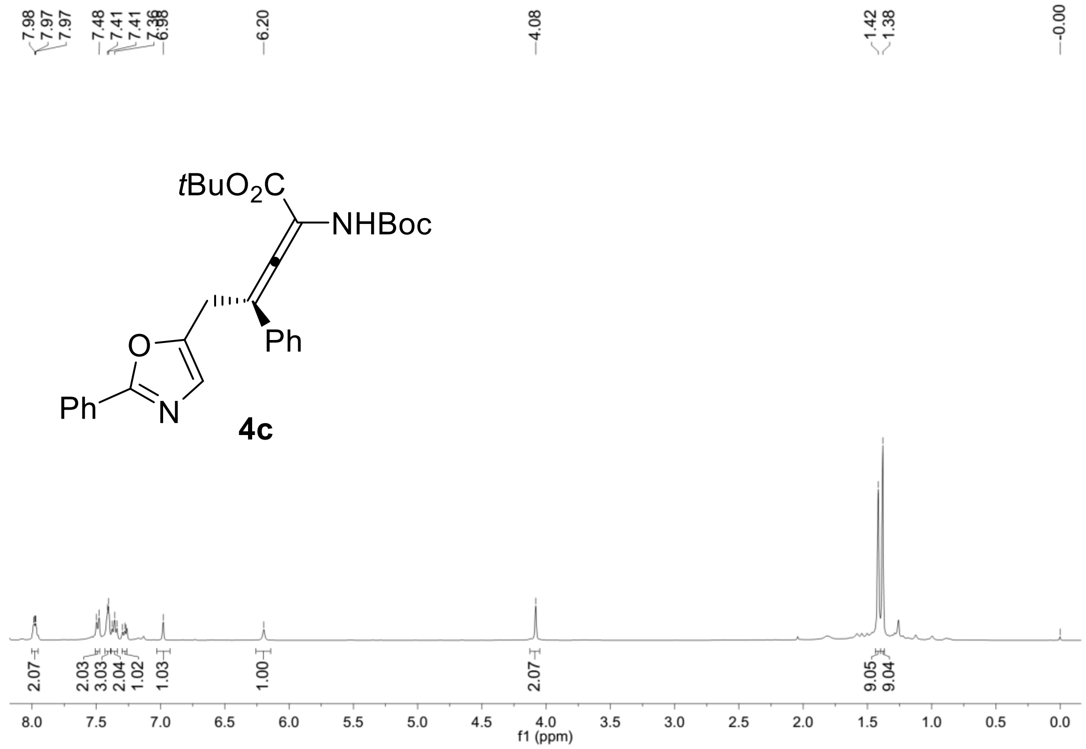
7.98
7.97
7.97
7.48
7.41
7.41
7.36
6.98
6.20
4.08
1.42
1.38
-0.00
tBuO2C
NHBoc
Ph
O
Ph
N
4c
2.07
2.03
3.03
2.04
1.02
1.03
1.00
2.07
9.05
9.04
f1 (ppm)

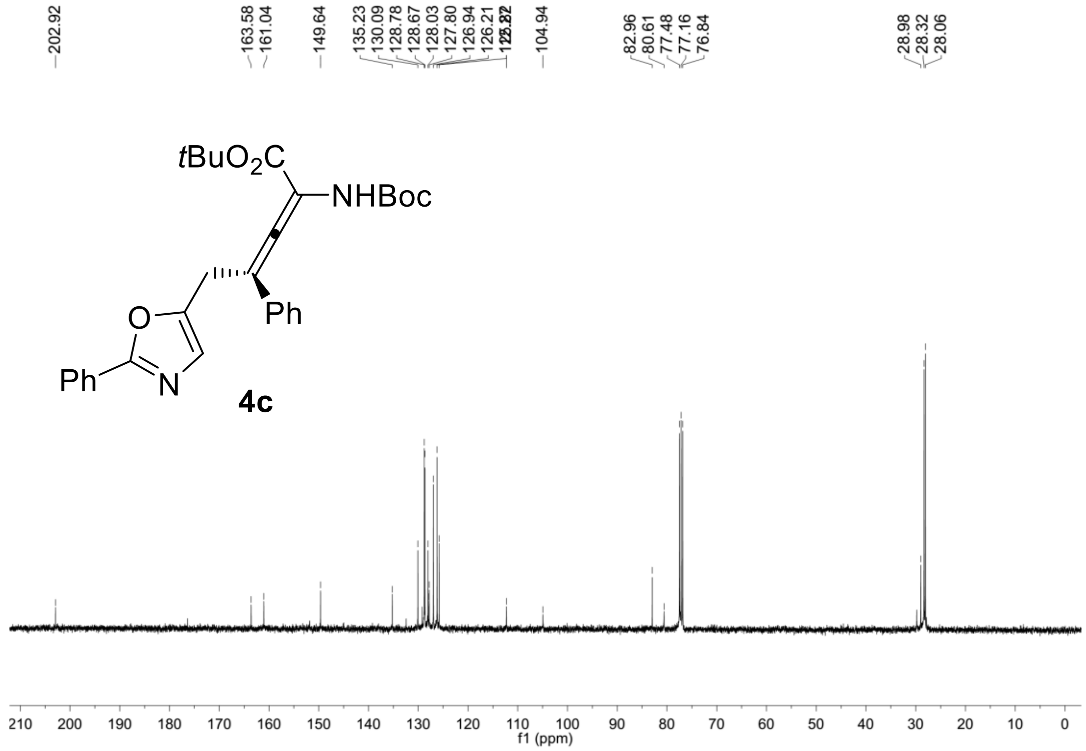
202.92
163.58
161.04
149.64
135.23
130.09
128.78
128.67
128.03
127.80
126.94
126.21
104.94
82.96
80.61
77.48
77.16
76.84
28.98
28.32
28.06
tBuO2C
NHBoc
Ph
O
Ph
N
4c
f1 (ppm)

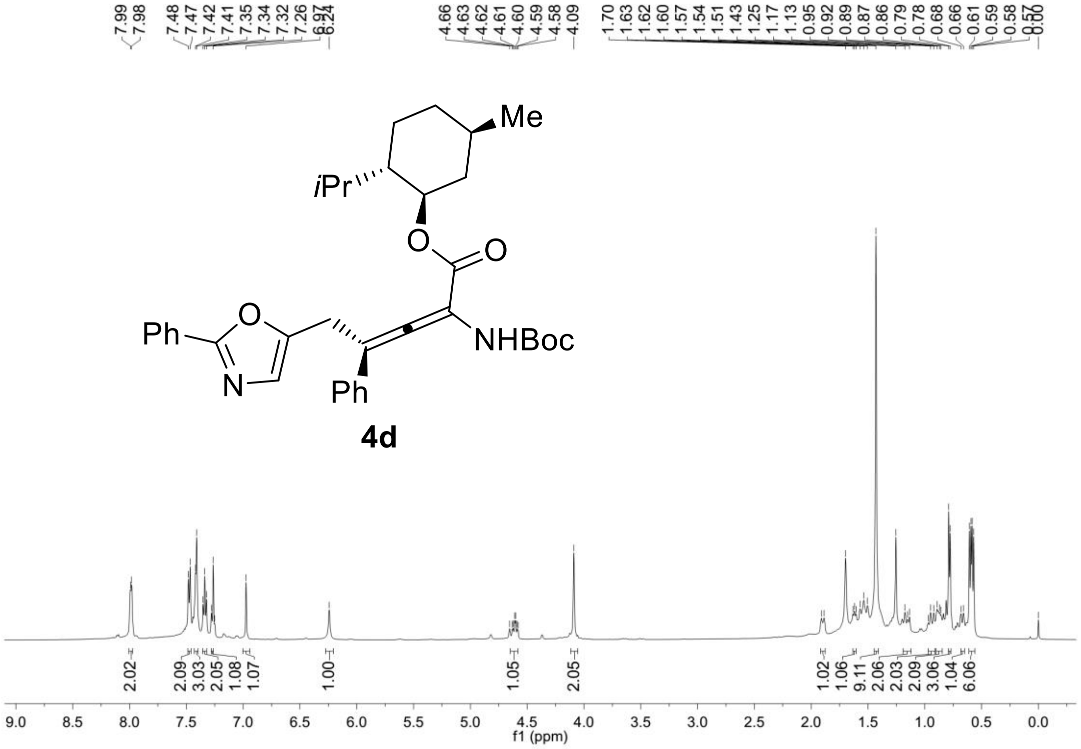
7.99 7.98 7.48 7.47 7.42 7.41 7.35 7.34 7.32 7.26 6.97 6.24
4.66 4.63 4.62 4.61 4.60 4.59 4.58 4.09
1.70 1.63 1.62 1.60 1.57 1.54 1.51 1.43 1.25 1.17 1.13 0.95 0.92 0.89 0.87 0.86 0.79 0.78 0.68 0.66 0.61 0.59 0.58 0.57 0.00
Me
iPr
O
O
Ph
O
N
NHBoc
Ph
4d
2.02 2.09 3.03 2.05 1.08 1.07 1.00 1.05 2.05 1.02 1.06 9.11 2.06 2.03 2.09 3.06 1.04 6.06
f1 (ppm)

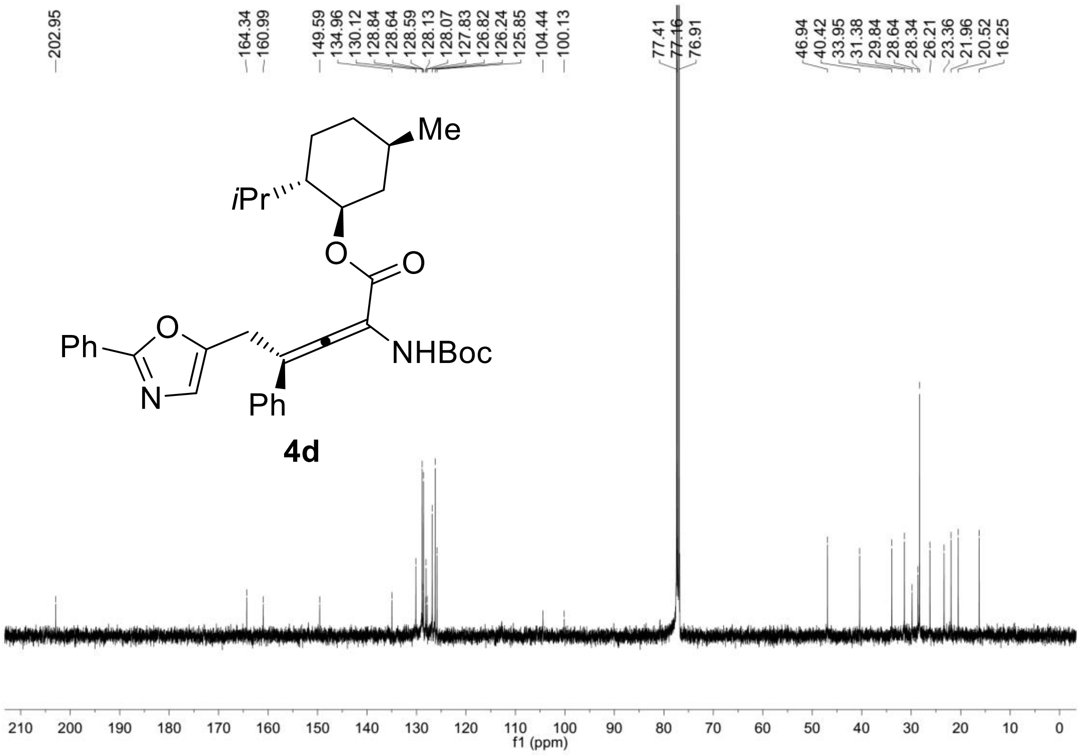
202.95
164.34 160.99
149.59 134.96 130.12 128.84 128.64 128.59 128.13 128.07 127.83 126.82 126.24 125.85 104.44 100.13
77.41 77.16 76.91
46.94 40.42 33.95 31.38 29.84 28.64 28.34 26.21 23.36 21.96 20.52 16.25
Me
iPr
O
O
Ph
O
N
NHBoc
Ph
4d
f1 (ppm)

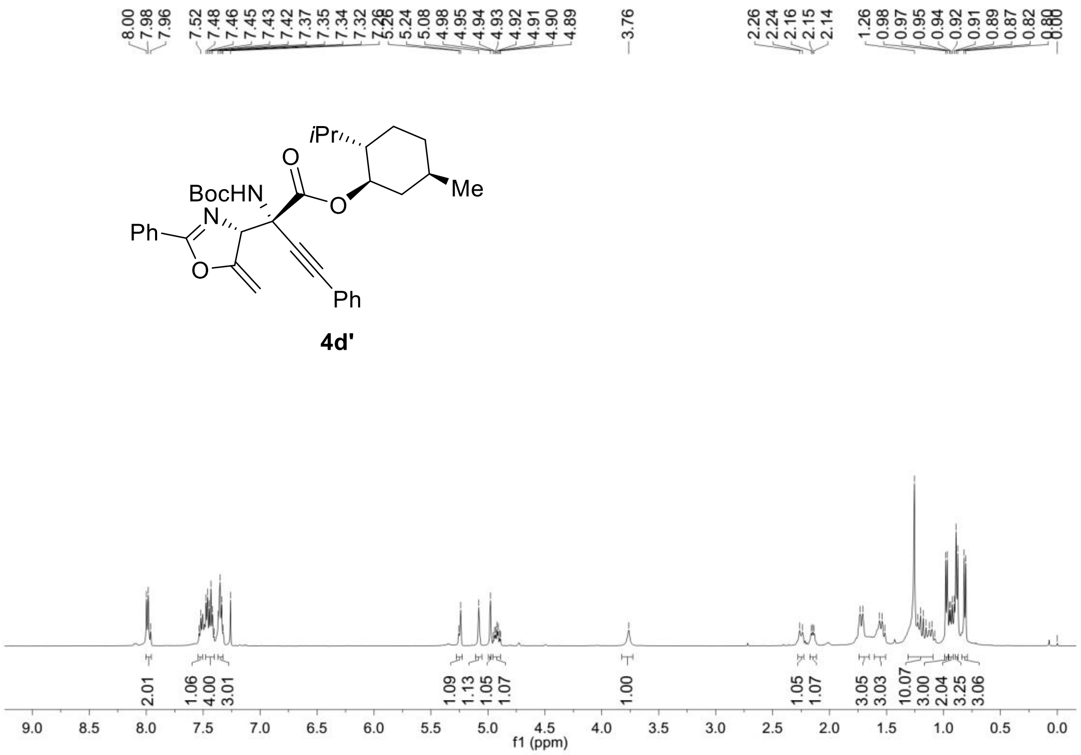
8.00 7.98 7.96 7.52 7.48 7.46 7.45 7.43 7.42 7.37 7.35 7.34 7.32 5.24 5.08 4.98 4.95 4.94 4.93 4.92 4.91 4.90 4.89 3.76 2.26 2.24 2.16 2.15 2.14 1.26 0.98 0.97 0.95 0.94 0.92 0.91 0.89 0.87 0.82
iPr
O
BocHN
N
O
Ph
Me
Ph
2.01 1.06 4.00 3.01 1.09 1.13 1.05 1.07 1.00 1.05 1.07 3.05 3.03 10.07 3.00 2.04 3.25 3.06
9.0 8.5 8.0 7.5 7.0 6.5 6.0 5.5 5.0 4.5 4.0 3.5 3.0 2.5 2.0 1.5 1.0 0.5 0.0
f1 (ppm)

4d'

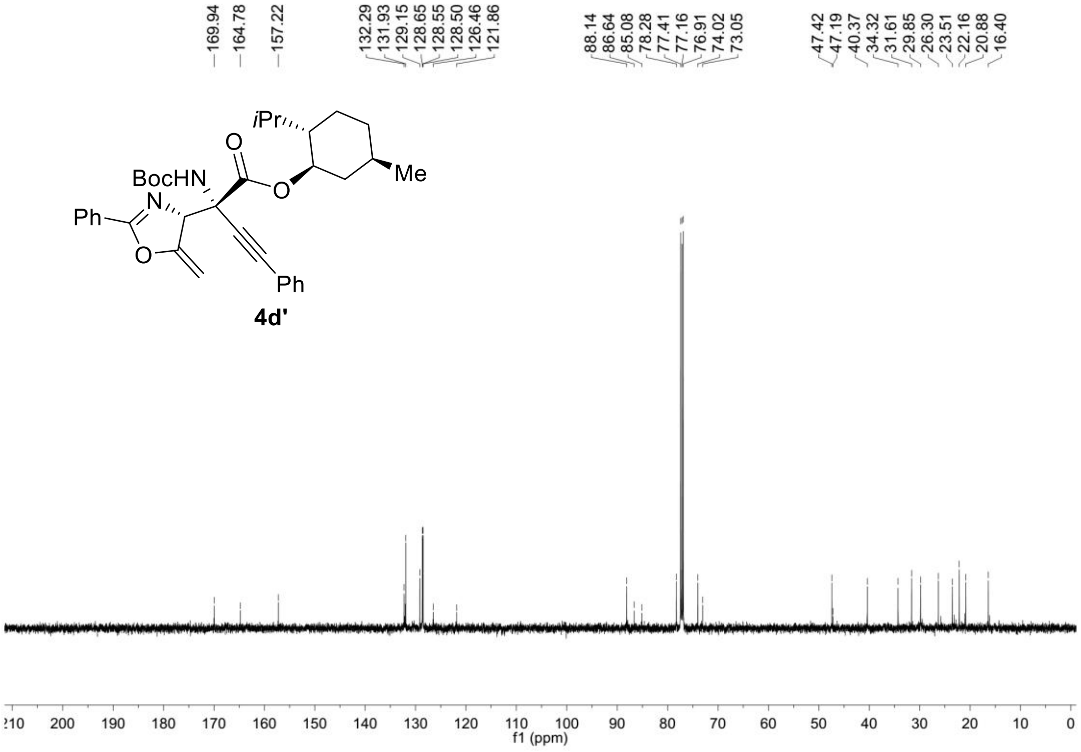
169.94 164.78 157.22 132.29 131.93 129.15 128.65 128.55 128.50 126.46 121.86 88.14 86.64 85.08 78.28 77.41 77.16 76.91 74.02 73.05 47.42 47.19 40.37 34.32 31.61 29.85 26.30 23.51 22.16 20.88 16.40
iPr
O
BocHN
N
O
Ph
Me
Ph
200 190 180 170 160 150 140 130 120 110 100 90 80 70 60 50 40 30 20 10 0
f1 (ppm)

4d'

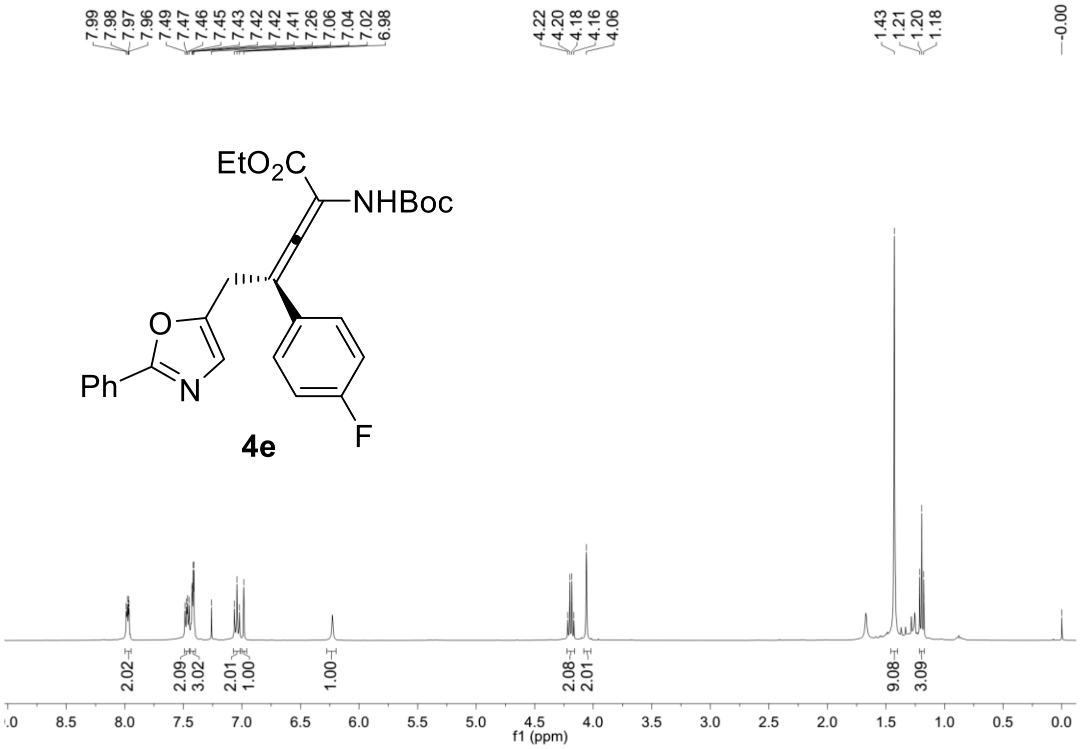
EtO2C
NHBoc
O
N
Ph
F
4e
f1 (ppm)

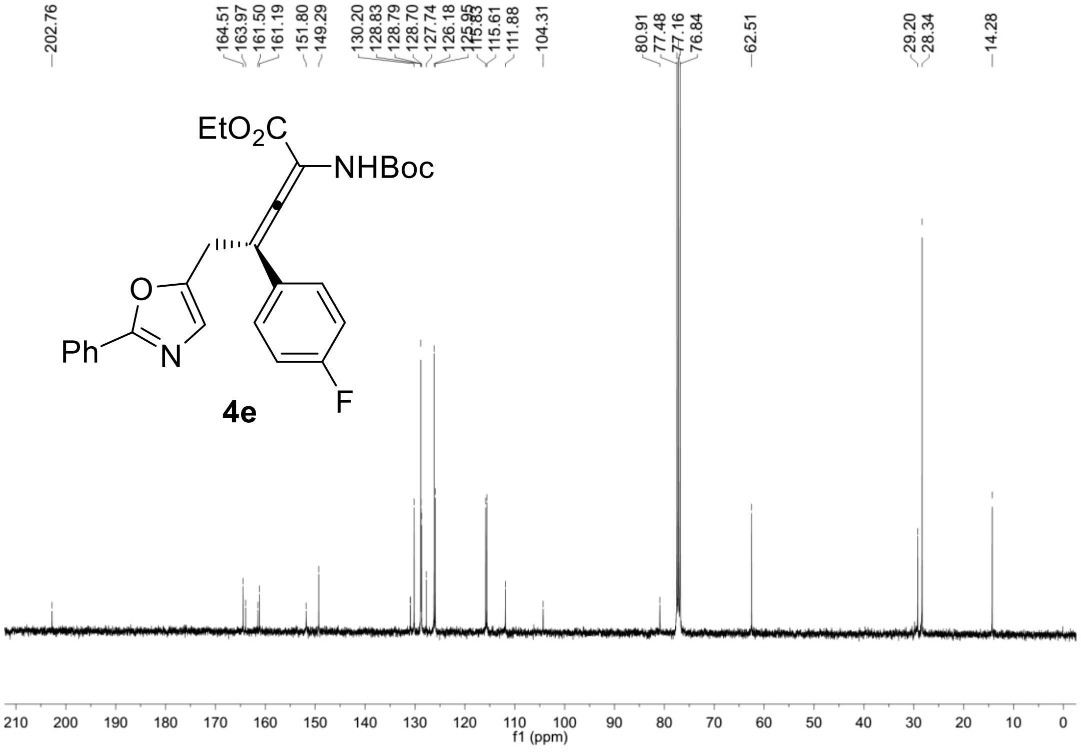
EtO2C
NHBoc
O
N
Ph
F
4e
f1 (ppm)

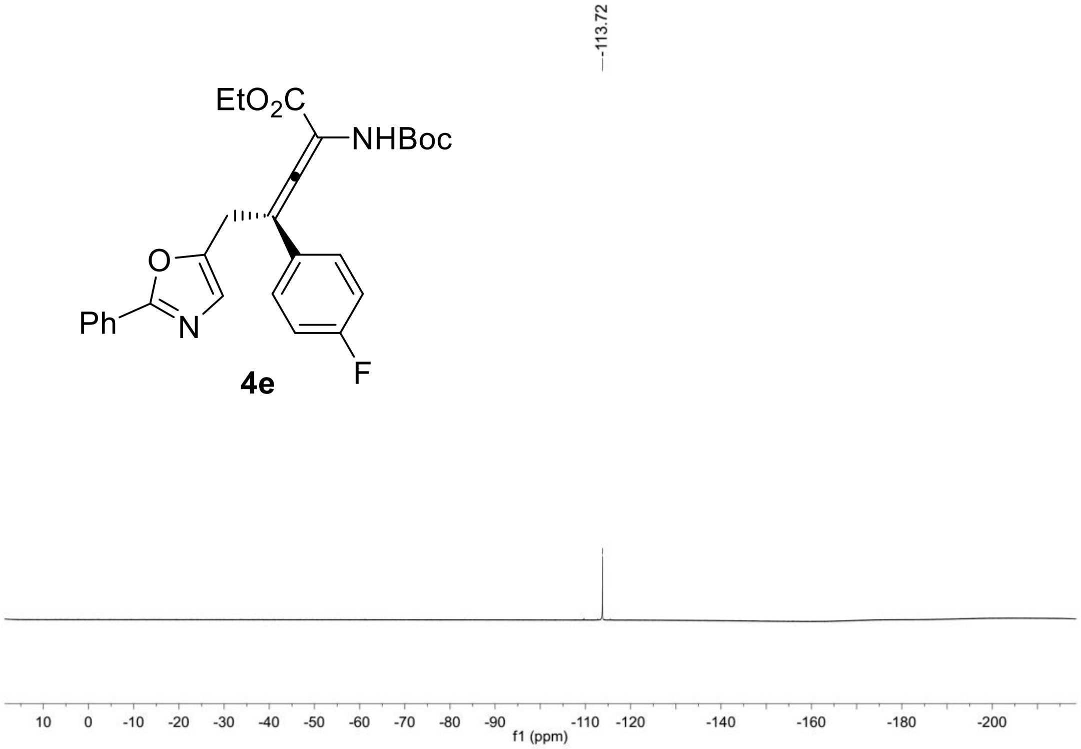

-113.72
EtO2C
NHBoc
O
Ph
N
4e
F
f1 (ppm)

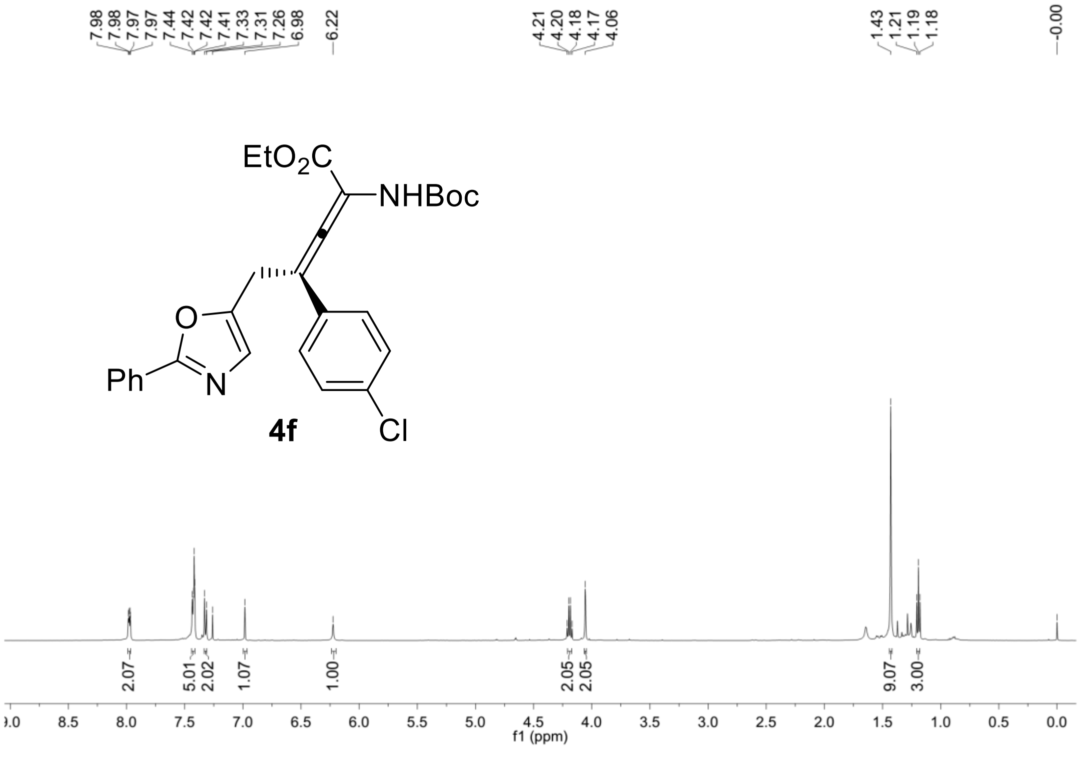

7.98 7.98 7.97 7.97 7.44 7.42 7.42 7.41 7.33 7.31 7.26 6.98
6.22
4.21 4.20 4.18 4.17 4.06
1.43 1.21 1.19 1.18
-0.00
EtO2C
NHBoc
O
Ph
N
4f
Cl
2.07 5.01 2.02 1.07 1.00 2.05 2.05 9.07 3.00
f1 (ppm)

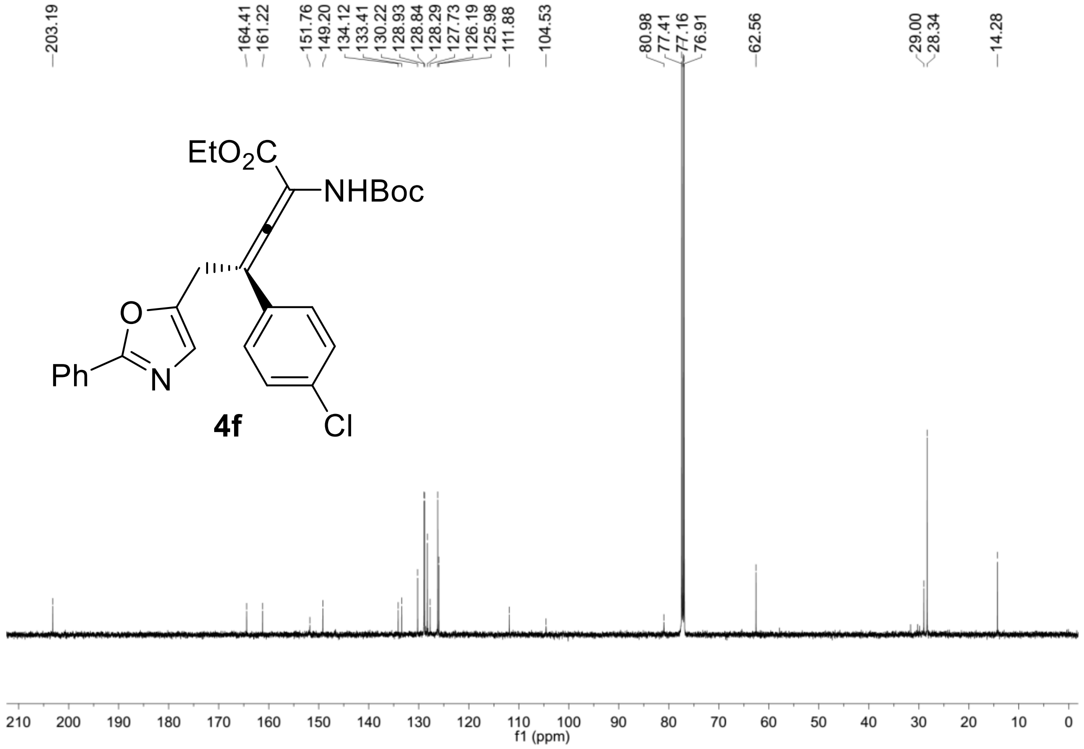
203.19
164.41
161.22
151.76
149.20
134.12
133.41
130.22
128.93
128.84
128.29
127.73
126.19
125.98
111.88
104.53
80.98
77.41
77.16
76.91
62.56
29.00
28.34
14.28
EtO2C
NHBoc
O
N
Ph
4f
Cl
f1 (ppm)

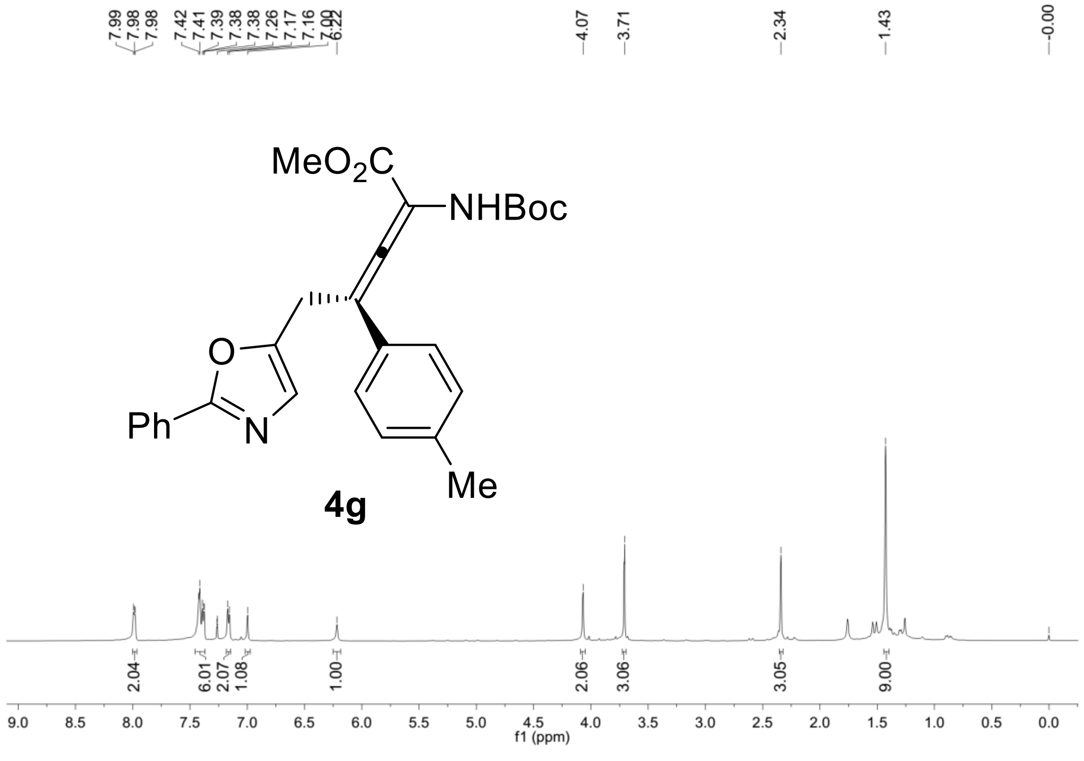
7.99
7.98
7.98
7.42
7.41
7.39
7.38
7.38
7.26
7.17
7.16
6.22
4.07
3.71
2.34
1.43
-0.00
MeO2C
NHBoc
O
N
Ph
4g
Me
2.04
6.01
2.07
1.08
1.00
2.06
3.06
3.05
9.00
f1 (ppm)

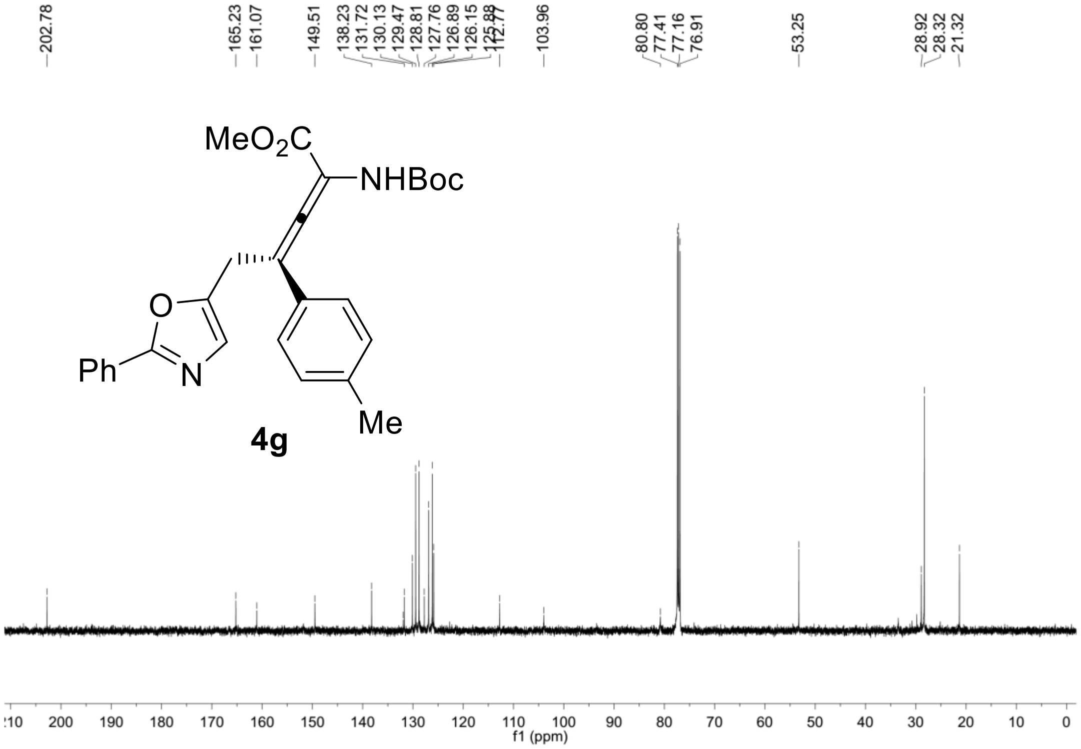
202.78
165.23
161.07
149.51
138.23
131.72
130.13
129.47
128.81
127.76
126.89
126.15
112.77
103.96
80.80
77.41
77.16
76.91
53.25
28.92
28.32
21.32
MeO2C
NHBoc
O
Ph
N
Me
4g
f1 (ppm)

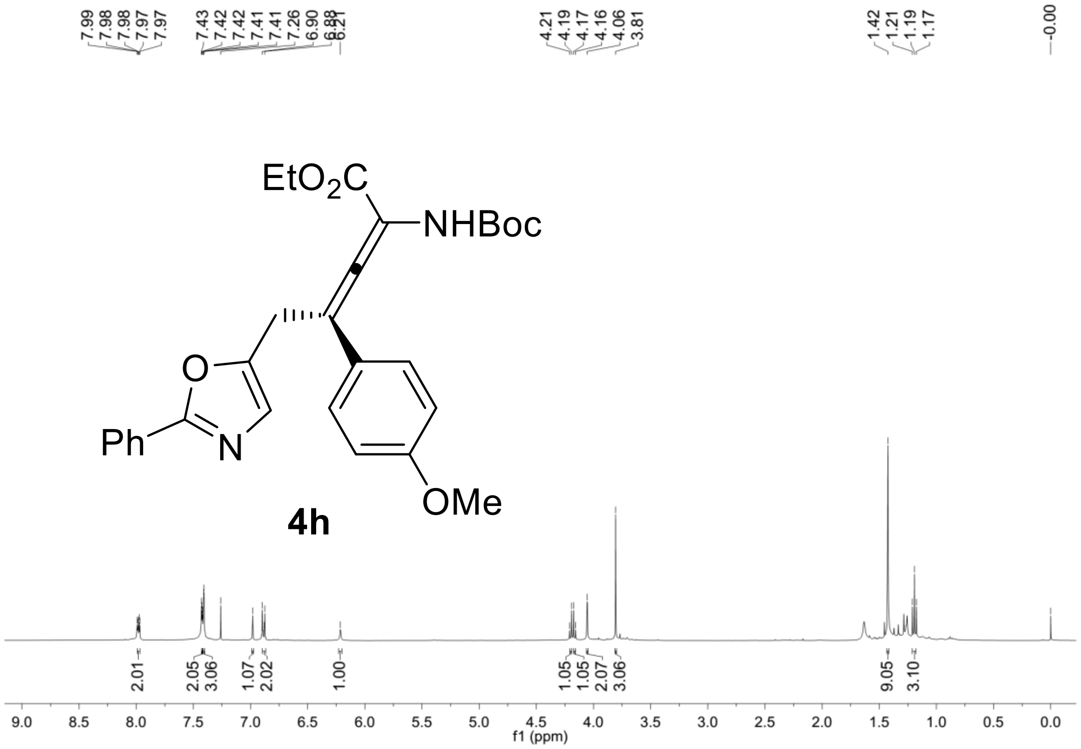
7.99
7.98
7.98
7.97
7.97
7.43
7.42
7.42
7.41
7.41
7.26
6.90
6.88
6.21
4.21
4.19
4.17
4.16
4.06
3.81
1.42
1.21
1.19
1.17
-0.00
EtO2C
NHBoc
O
Ph
N
OMe
4h
2.01
2.05
3.06
1.07
2.02
1.00
1.05
1.05
2.07
3.06
9.05
3.10
f1 (ppm)

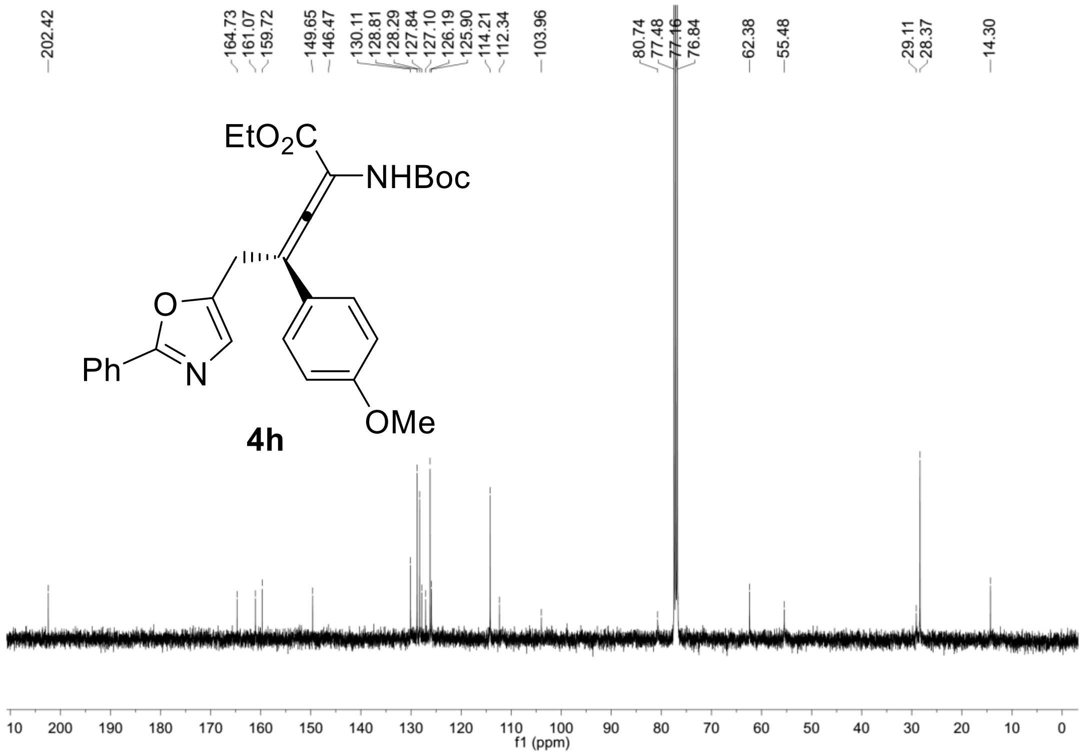

4h

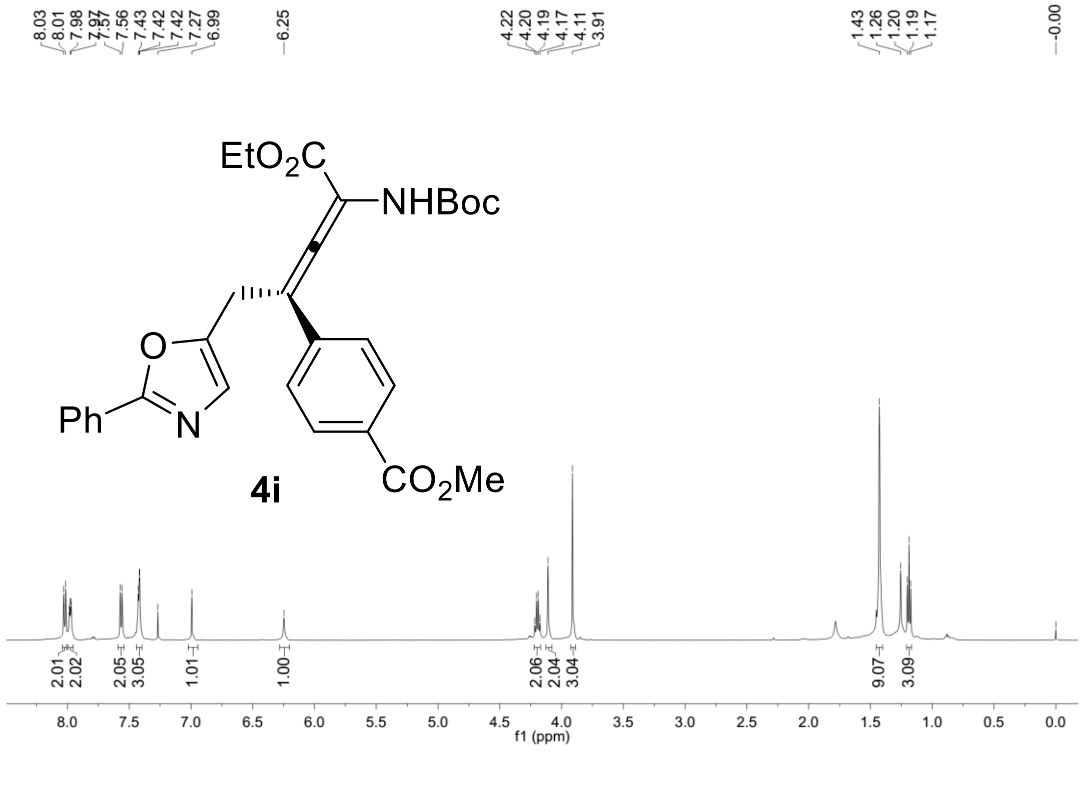

4i

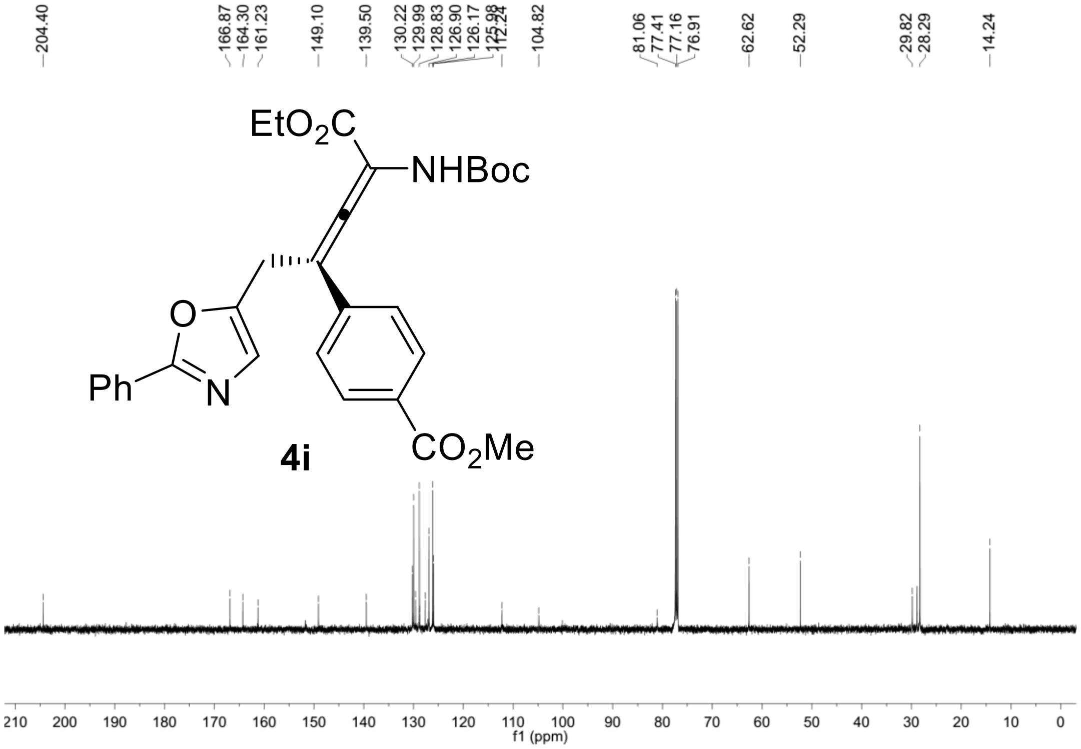
204.40
166.87
164.30
161.23
149.10
139.50
130.22
129.99
128.83
126.90
126.17
125.98
112.24
104.82
81.06
77.41
77.16
76.91
62.62
52.29
29.82
28.29
14.24
EtO2C
NHBoc
O
Ph
N
CO2Me
4i
f1 (ppm)

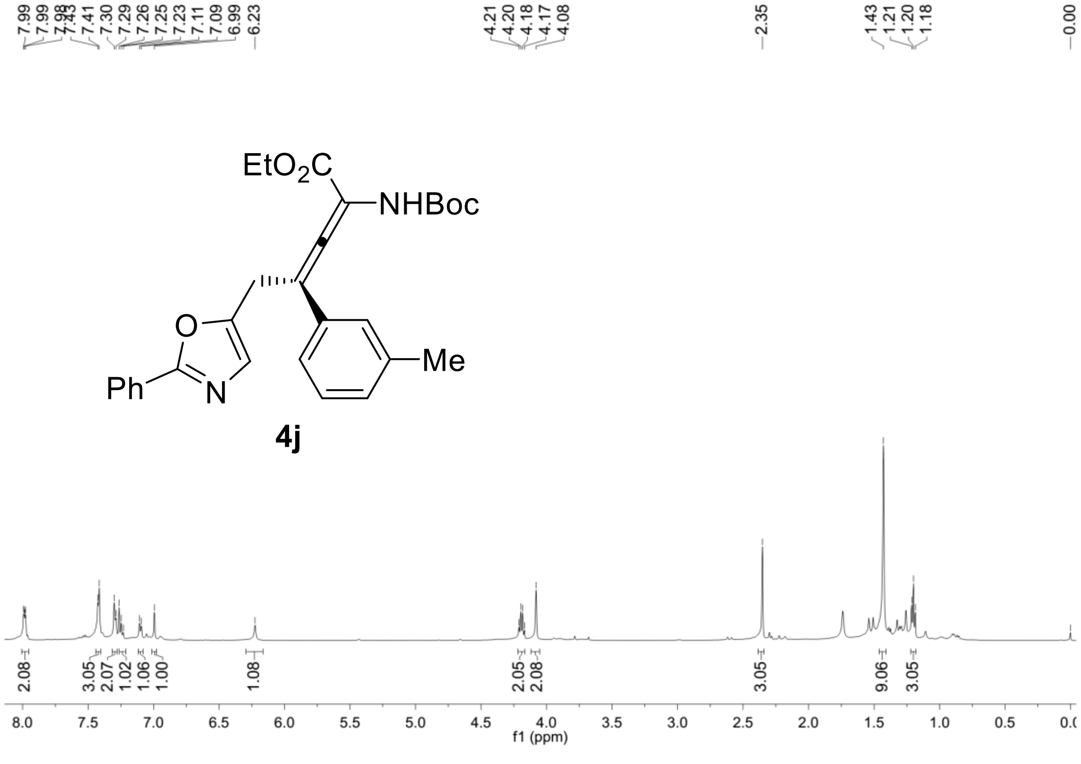
7.99
7.99
7.98
7.43
7.41
7.30
7.29
7.26
7.25
7.23
7.11
7.09
6.99
6.23
4.21
4.20
4.18
4.17
4.08
2.35
1.43
1.21
1.20
1.18
0.00
EtO2C
NHBoc
O
Ph
N
Me
4j
2.08
3.05
2.07
1.02
1.06
1.00
1.08
2.05
2.08
3.05
9.06
3.05
f1 (ppm)

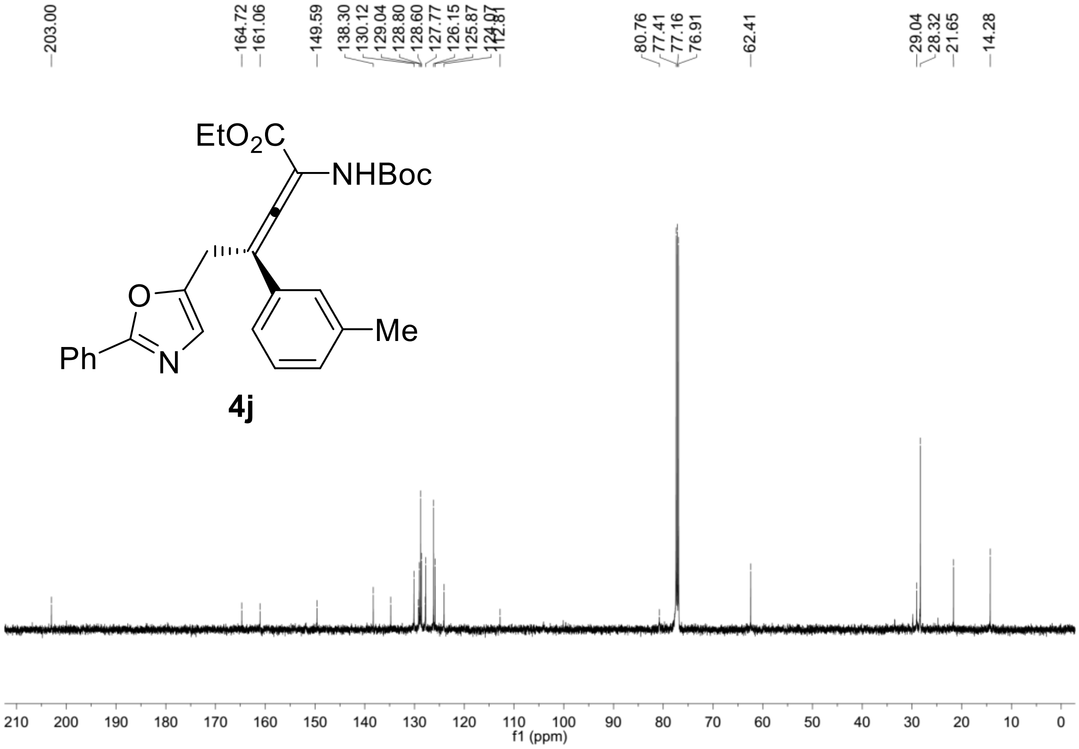
203.00
164.72
161.06
149.59
138.30
130.12
129.04
128.80
128.60
127.77
126.15
125.87
112.81
80.76
77.41
77.16
76.91
62.41
29.04
28.32
21.65
14.28
EtO2C
NHBoc
O
N
Ph
Me
f1 (ppm)

4j

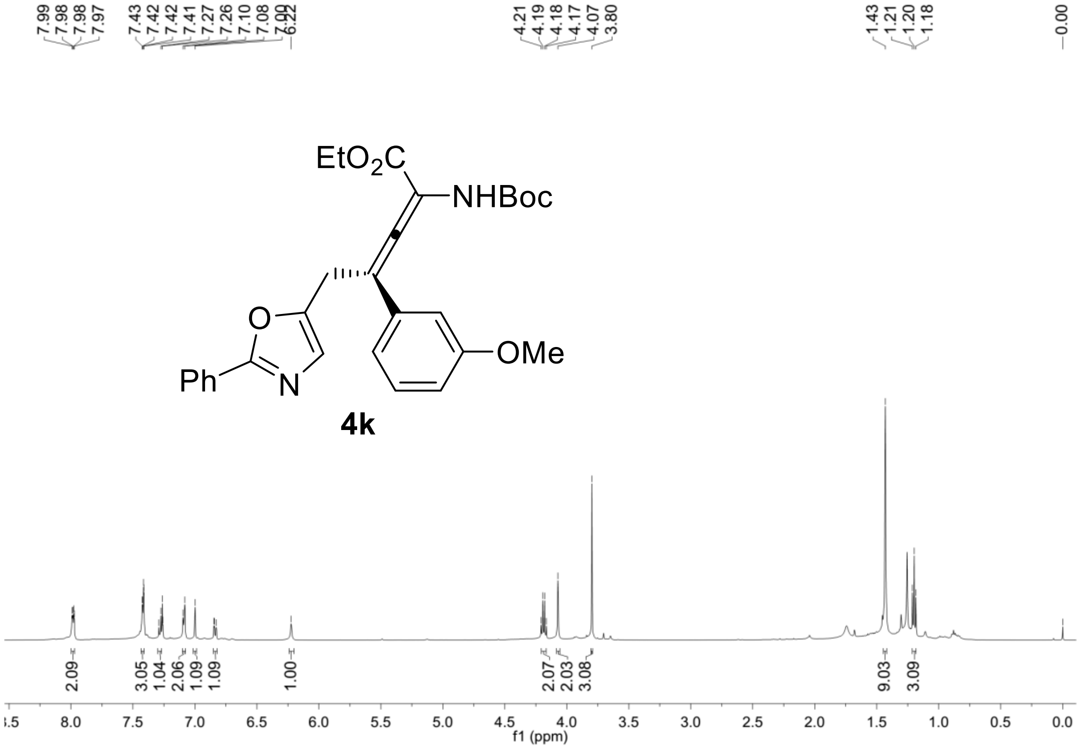
7.99
7.98
7.98
7.97
7.43
7.42
7.42
7.41
7.27
7.26
7.10
7.08
4.21
4.19
4.18
4.17
4.07
3.80
1.43
1.21
1.20
1.18
0.00
EtO2C
NHBoc
O
N
Ph
OMe
2.09
3.05
1.04
2.06
1.09
1.09
1.00
2.07
2.03
3.08
9.03
3.09
f1 (ppm)

4k

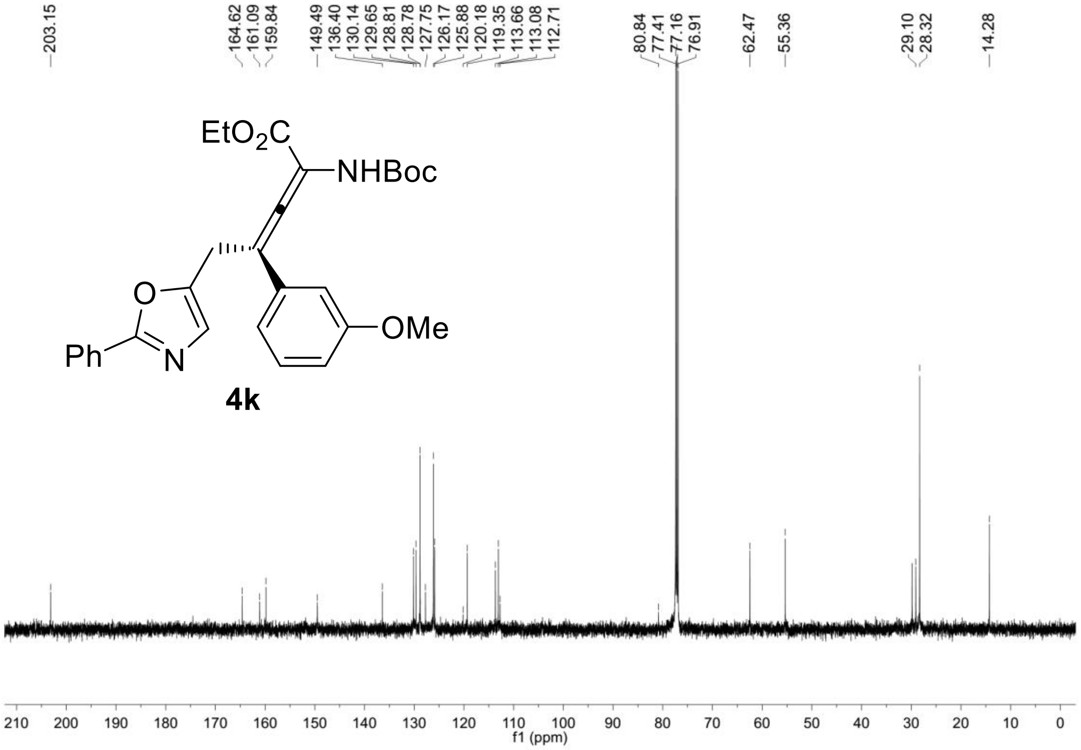
203.15
164.62
161.09
159.84
149.49
136.40
130.14
129.65
128.81
128.78
127.75
126.17
125.88
120.18
119.35
113.66
113.08
112.71
80.84
77.41
77.16
76.91
62.47
55.36
29.10
28.32
14.28
EtO2C
NHBoc
OMe
Ph
O
N
4k
f1 (ppm)

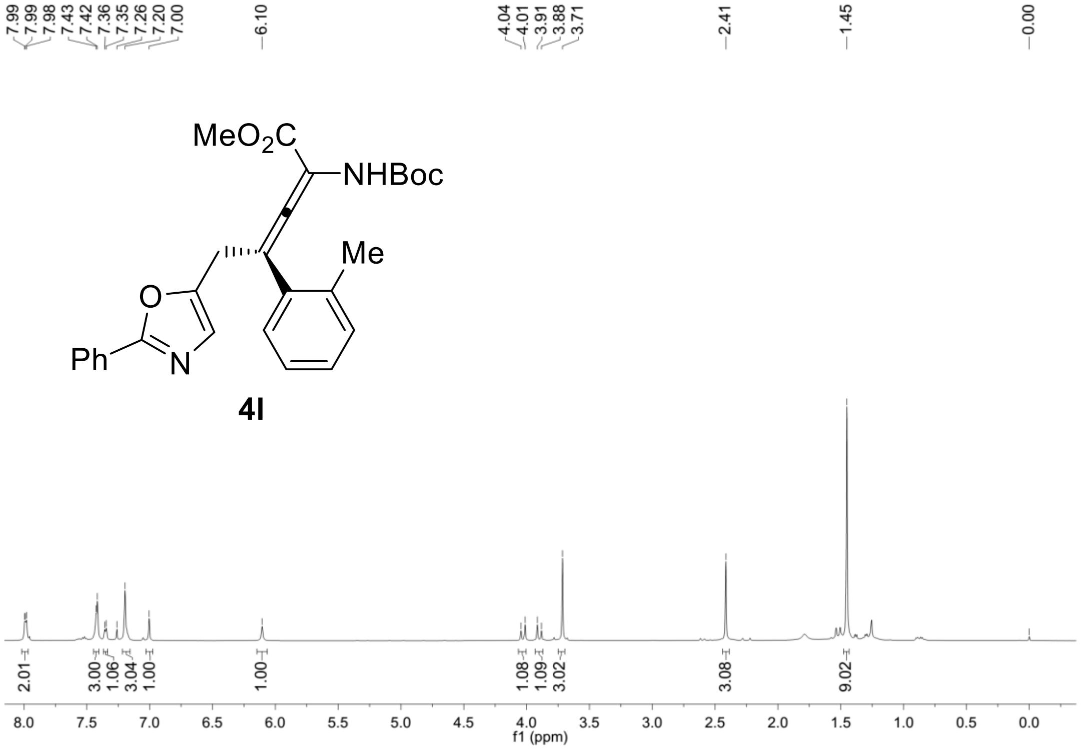
7.99
7.99
7.98
7.43
7.42
7.36
7.35
7.26
7.20
7.00
6.10
4.04
4.01
3.91
3.88
3.71
2.41
1.45
0.00
MeO2C
NHBoc
Me
Ph
O
N
4l
2.01
3.00
1.06
3.04
1.00
1.00
1.08
1.09
3.02
3.08
9.02
f1 (ppm)

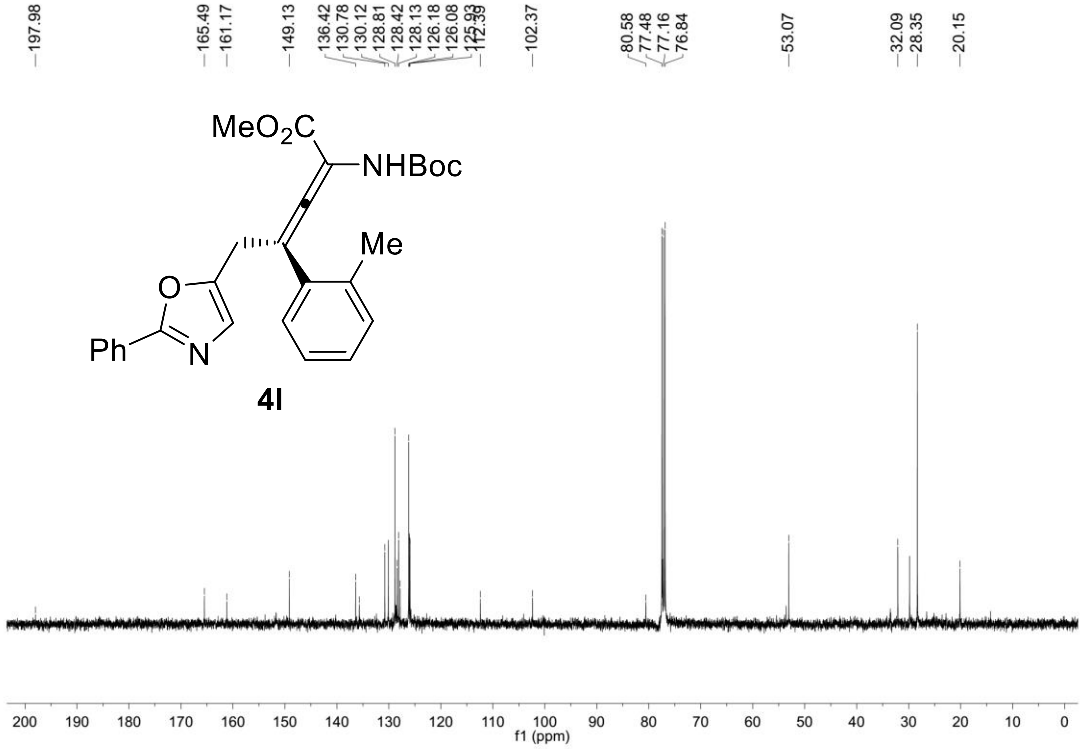
197.98
165.49
161.17
149.13
136.42
130.78
130.12
128.81
128.42
128.13
126.18
126.08
125.93
112.39
102.37
80.58
77.48
77.16
76.84
53.07
32.09
28.35
20.15
MeO2C
NHBoc
Me
O
Ph
N
4l
f1 (ppm)

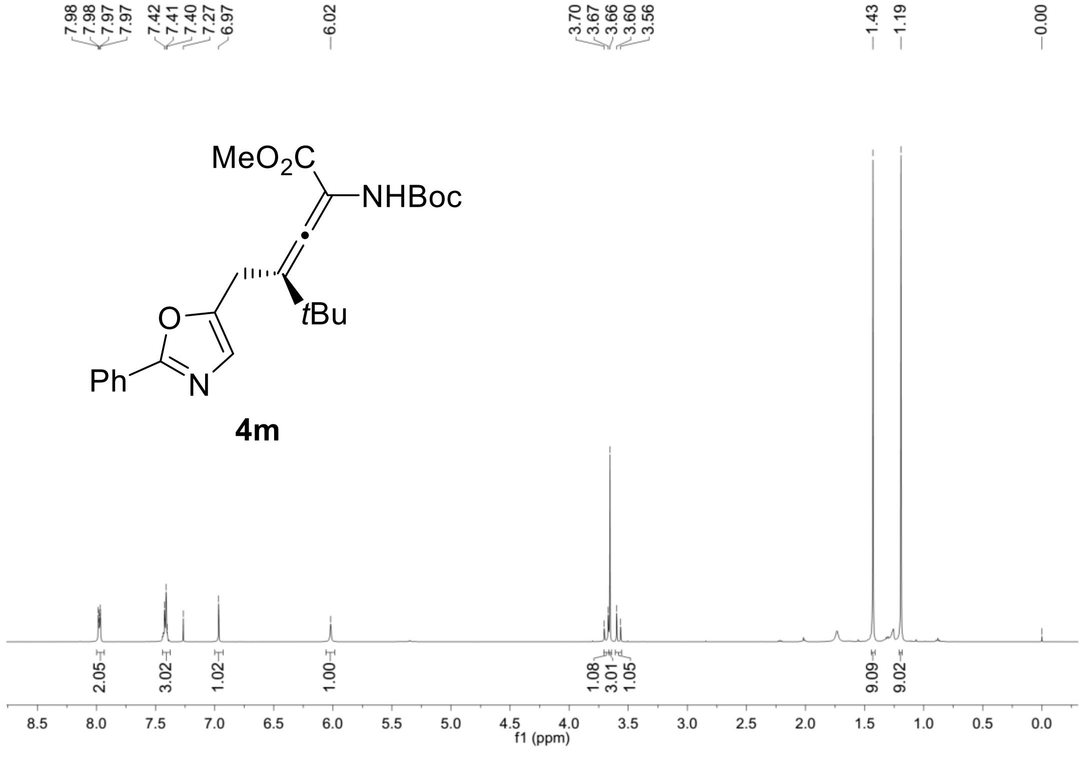
7.98
7.98
7.97
7.97
7.42
7.41
7.40
7.27
6.97
6.02
3.70
3.67
3.66
3.60
3.56
1.43
1.19
0.00
MeO2C
NHBoc
tBu
O
Ph
N
4m
2.05
3.02
1.02
1.00
1.08
3.01
1.05
9.09
9.02
f1 (ppm)

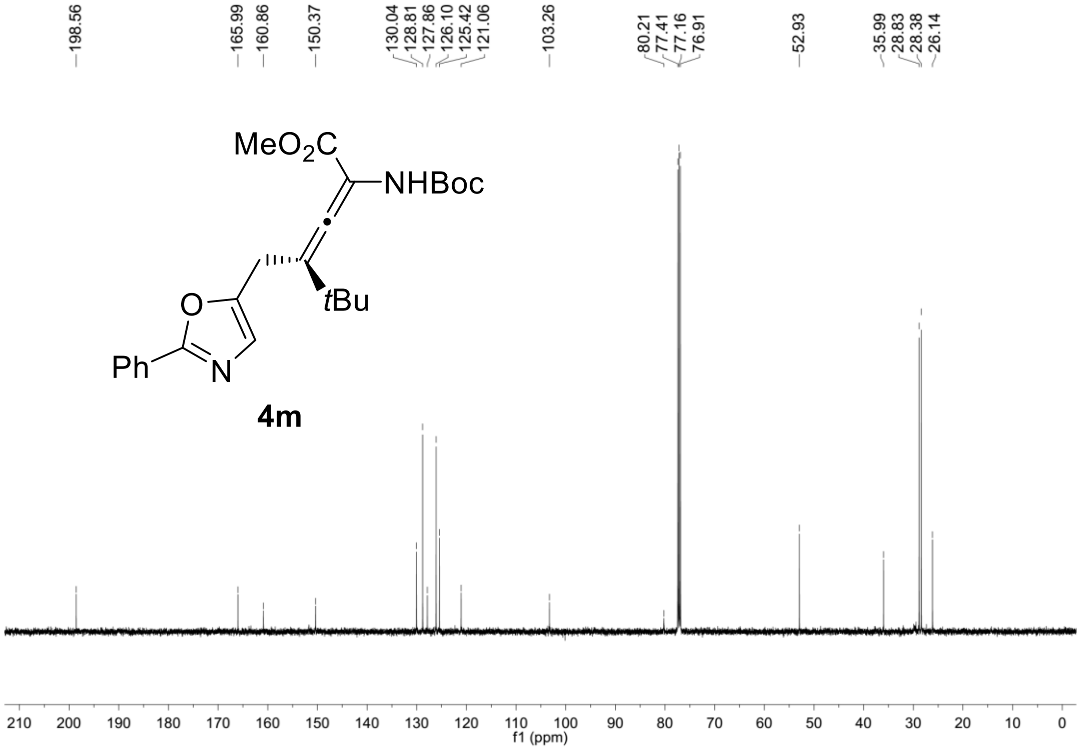
198.56
165.99
160.86
150.37
130.04
128.81
127.86
126.10
125.42
121.06
103.26
80.21
77.41
77.16
76.91
52.93
35.99
28.83
28.38
26.14
MeO2C
NHBoc
tBu
O
N
Ph
4m
210 200 190 180 170 160 150 140 130 120 110 100 90 80 70 60 50 40 30 20 10 0
f1 (ppm)

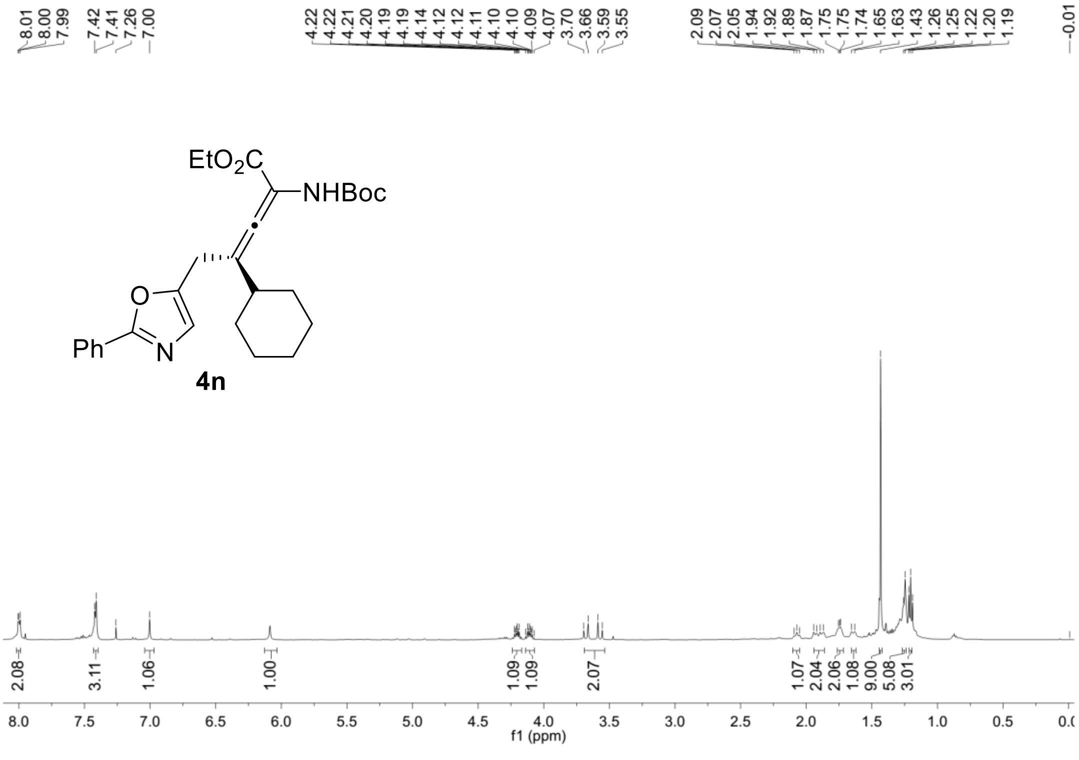
8.01
8.00
7.99
7.42
7.41
7.26
7.00
4.22
4.22
4.21
4.20
4.19
4.19
4.14
4.12
4.12
4.11
4.10
4.10
4.09
4.07
3.70
3.66
3.59
3.55
2.09
2.07
2.05
1.94
1.92
1.89
1.87
1.75
1.75
1.74
1.65
1.63
1.43
1.26
1.25
1.22
1.20
1.19
-0.01
EtO2C
NHBoc
O
N
Ph
4n
2.08
3.11
1.06
1.00
1.09
1.09
2.07
1.07
2.04
2.06
1.08
9.00
5.08
3.01
8.0 7.5 7.0 6.5 6.0 5.5 5.0 4.5 4.0 3.5 3.0 2.5 2.0 1.5 1.0 0.5 0.0
f1 (ppm)

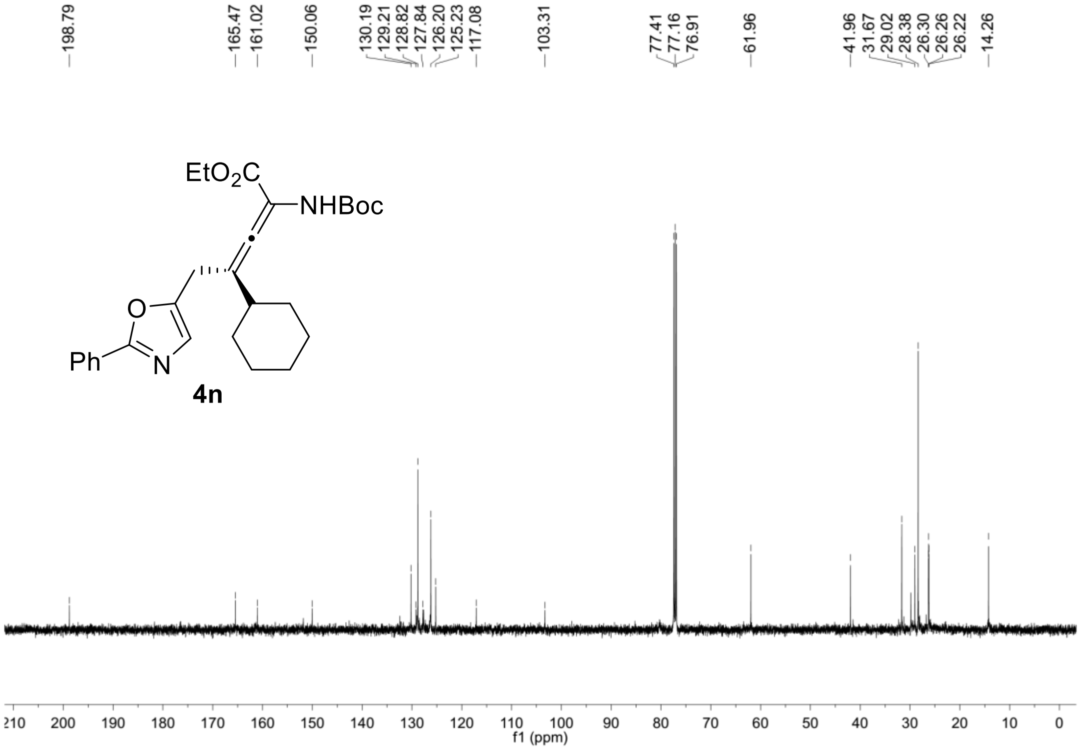
198.79
165.47
161.02
150.06
130.19
129.21
128.82
127.84
126.20
125.23
117.08
103.31
77.41
77.16
76.91
61.96
41.96
31.67
29.02
28.38
26.30
26.26
26.22
14.26
EtO2C
NHBoc
O
N
Ph
4n
f1 (ppm)

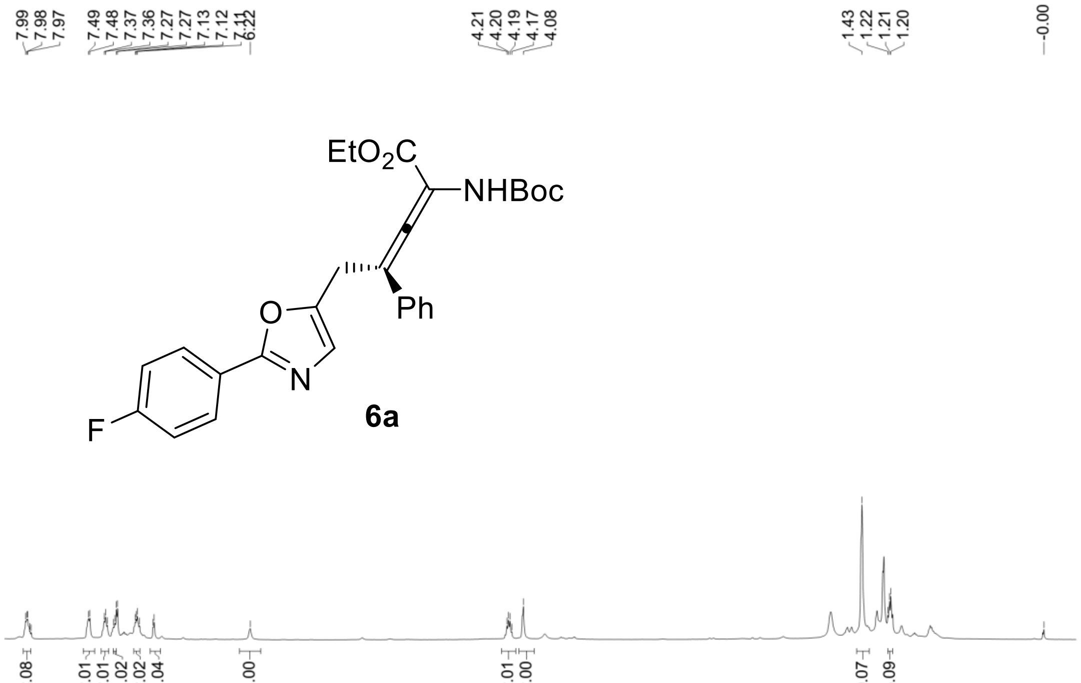
7.99
7.98
7.97
7.49
7.48
7.37
7.36
7.27
7.27
7.13
7.12
7.11
6.22
4.21
4.20
4.19
4.17
4.08
1.43
1.22
1.21
1.20
-0.00
EtO2C
NHBoc
Ph
O
N
F
6a
2.08
2.01
2.01
1.02
2.02
1.04
1.00
2.01
2.00
9.07
3.09
f1 (ppm)

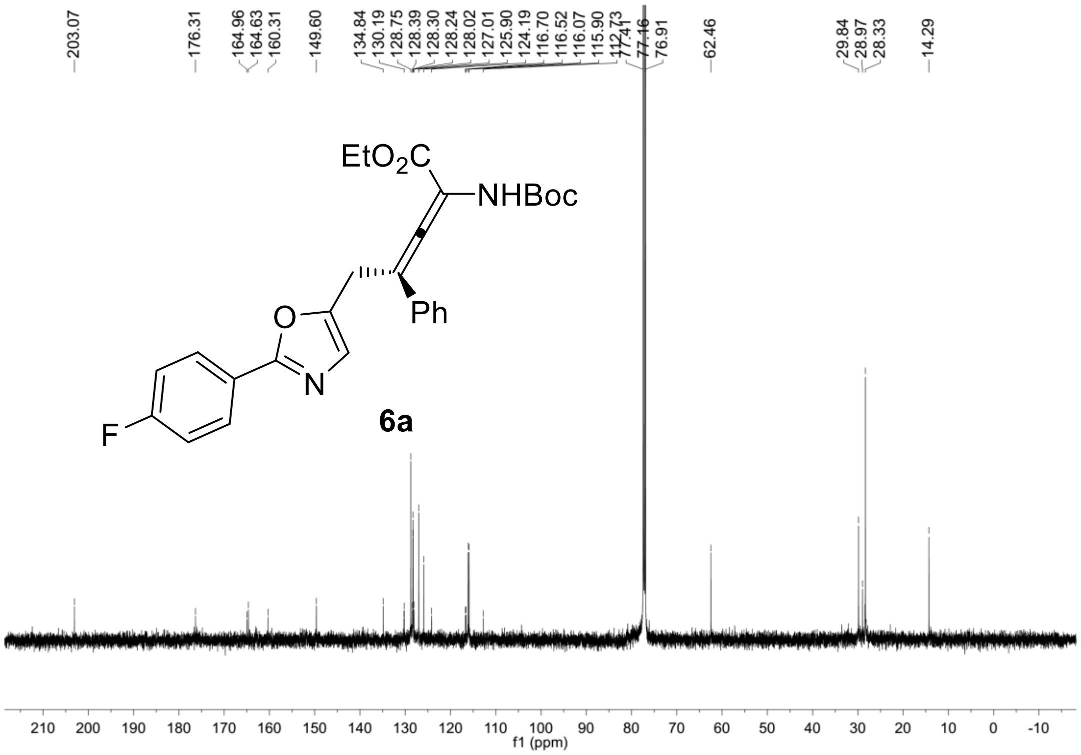
203.07
176.31
164.96
164.63
160.31
149.60
134.84
130.19
128.75
128.39
128.30
128.24
128.02
127.01
125.90
124.19
116.70
116.52
116.07
115.90
112.73
77.41
77.16
76.91
62.46
29.84
28.97
28.33
14.29
EtO2C
NHBoc
Ph
O
N
F
6a
f1 (ppm)

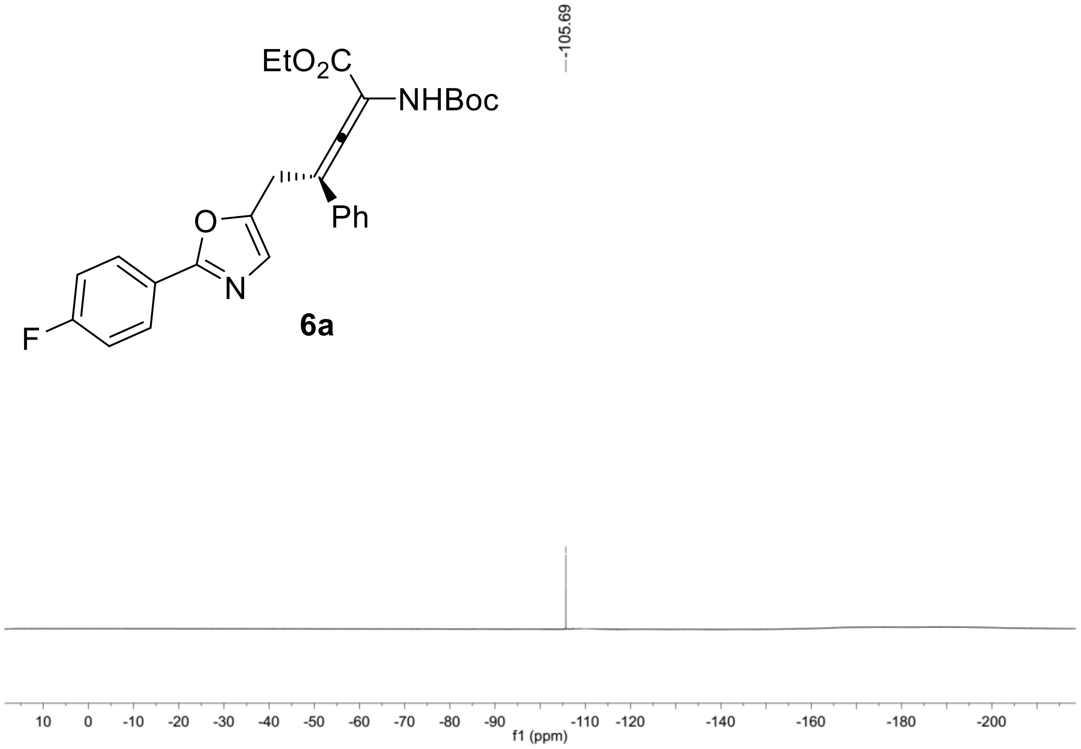
-105.69
EtO2C
NHBoc
Ph
O
N
F
6a
f1 (ppm)

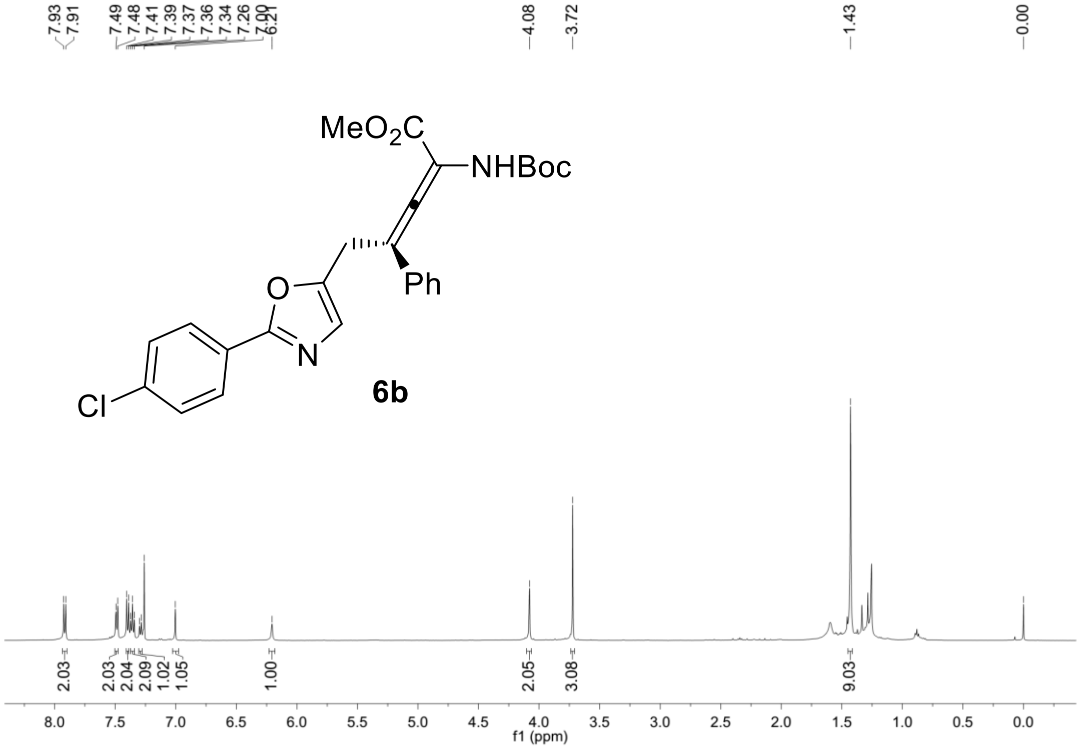
7.93 7.91 7.49 7.48 7.41 7.39 7.37 7.36 7.34 7.26 7.00 6.21
4.08 3.72 1.43 0.00
MeO2C
NHBoc
Ph
O
N
Cl
6b
2.03 2.03 2.04 2.09 1.02 1.05 1.00 2.05 3.08 9.03
f1 (ppm)

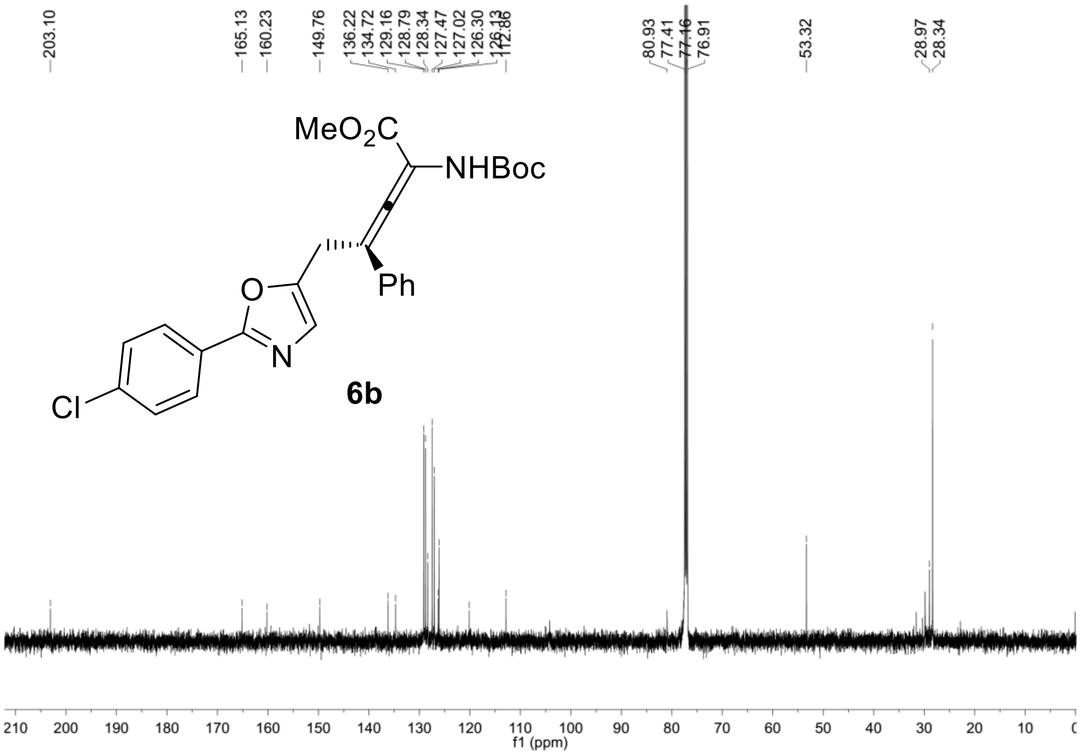
203.10 165.13 160.23 149.76 136.22 134.72 129.16 128.79 128.34 127.47 127.02 126.30 126.13 112.86
80.93 77.41 77.16 76.91 53.32 28.97 28.34
MeO2C
NHBoc
Ph
O
N
Cl
6b
f1 (ppm)

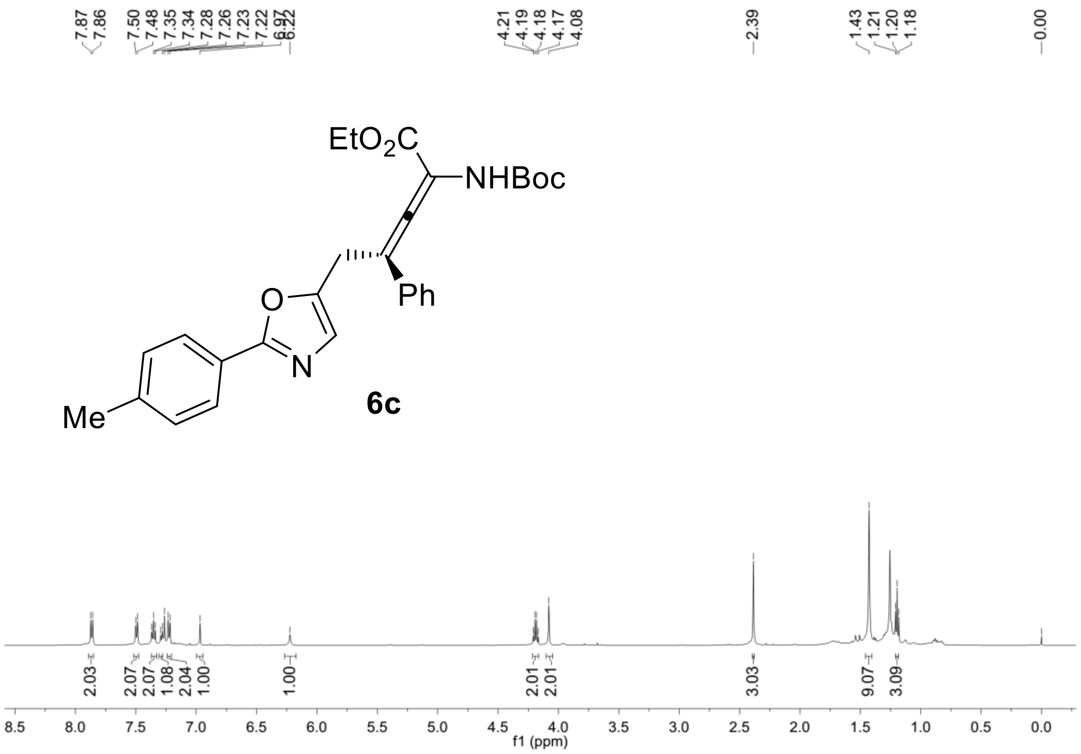
EtO2C
NHBoc
Ph
O
N
Me
6c
f1 (ppm)

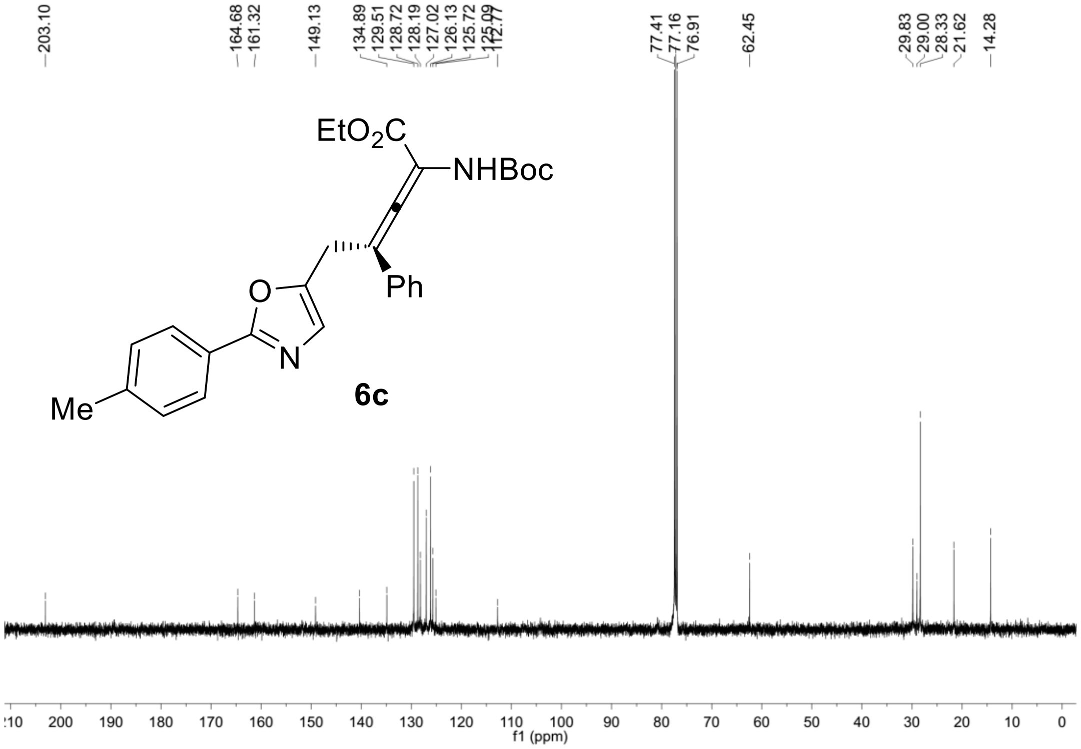
EtO2C
NHBoc
Ph
O
N
Me
6c
f1 (ppm)

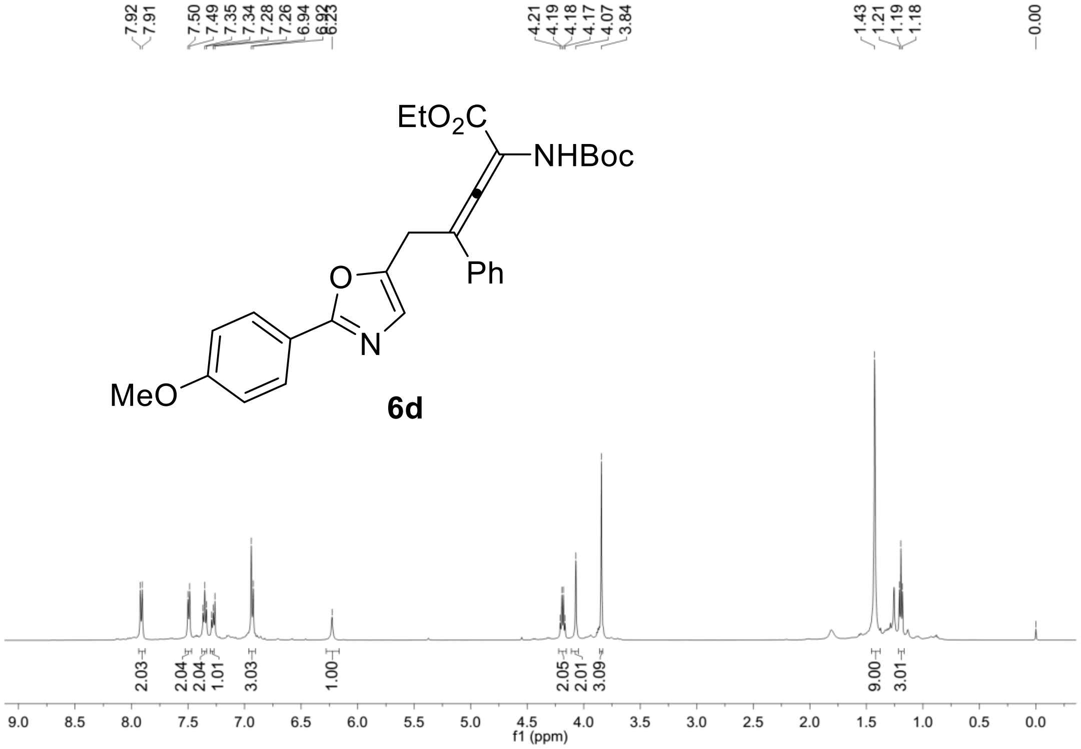
7.92
7.91
7.50
7.49
7.35
7.34
7.28
7.26
6.94
6.92
6.23
4.21
4.19
4.18
4.17
4.07
3.84
1.43
1.21
1.19
1.18
0.00
EtO2C
NHBoc
Ph
O
N
MeO
6d
2.03
2.04
2.04
1.01
3.03
1.00
2.05
2.01
3.09
9.00
3.01
f1 (ppm)

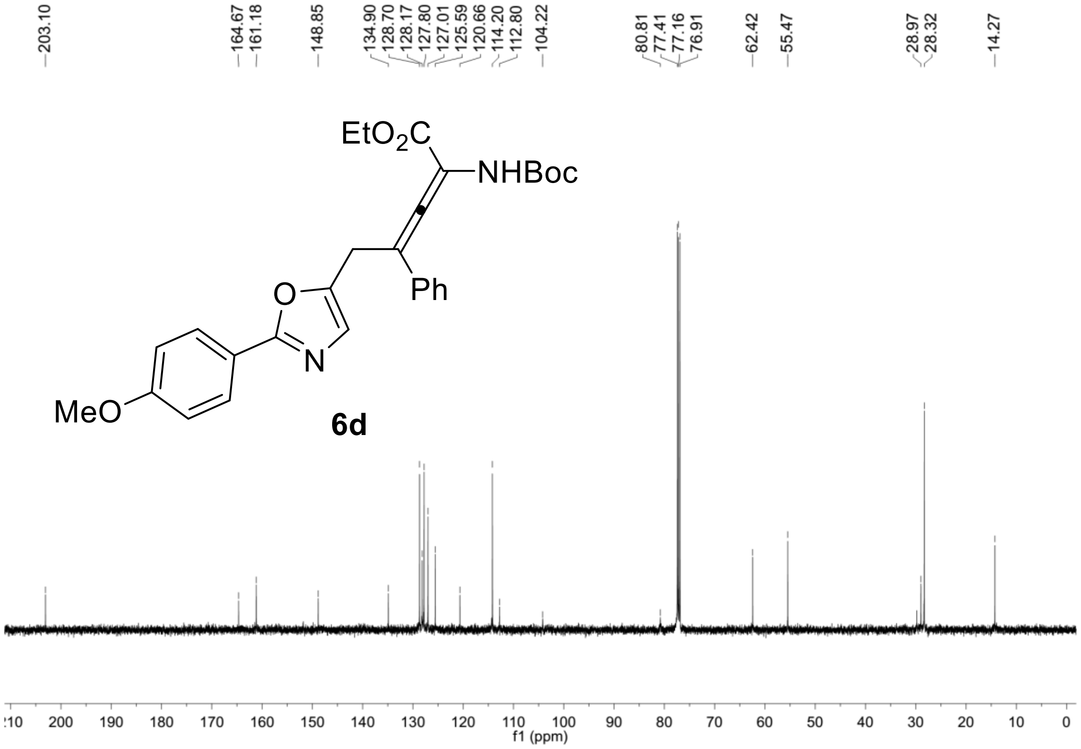
203.10
164.67
161.18
148.85
134.90
128.70
128.17
127.80
127.01
125.59
120.66
114.20
112.80
104.22
80.81
77.41
77.16
76.91
62.42
55.47
28.97
28.32
14.27
EtO2C
NHBoc
Ph
O
N
MeO
6d
f1 (ppm)

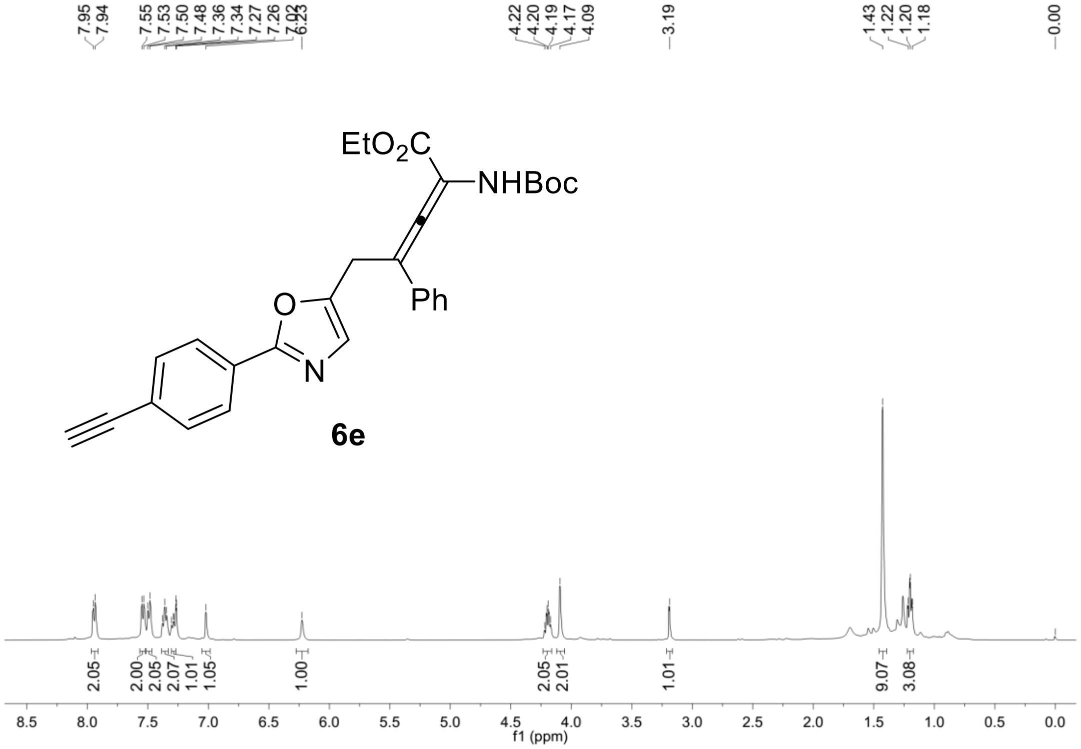
7.95
7.94
7.55
7.53
7.50
7.48
7.36
7.34
7.27
7.26
7.02
6.23
4.22
4.20
4.19
4.17
4.09
3.19
1.43
1.22
1.20
1.18
0.00
EtO2C
NHBoc
Ph
O
N
6e
2.05
2.00
2.05
2.07
1.01
1.05
1.00
2.05
2.01
1.01
9.07
3.08
f1 (ppm)

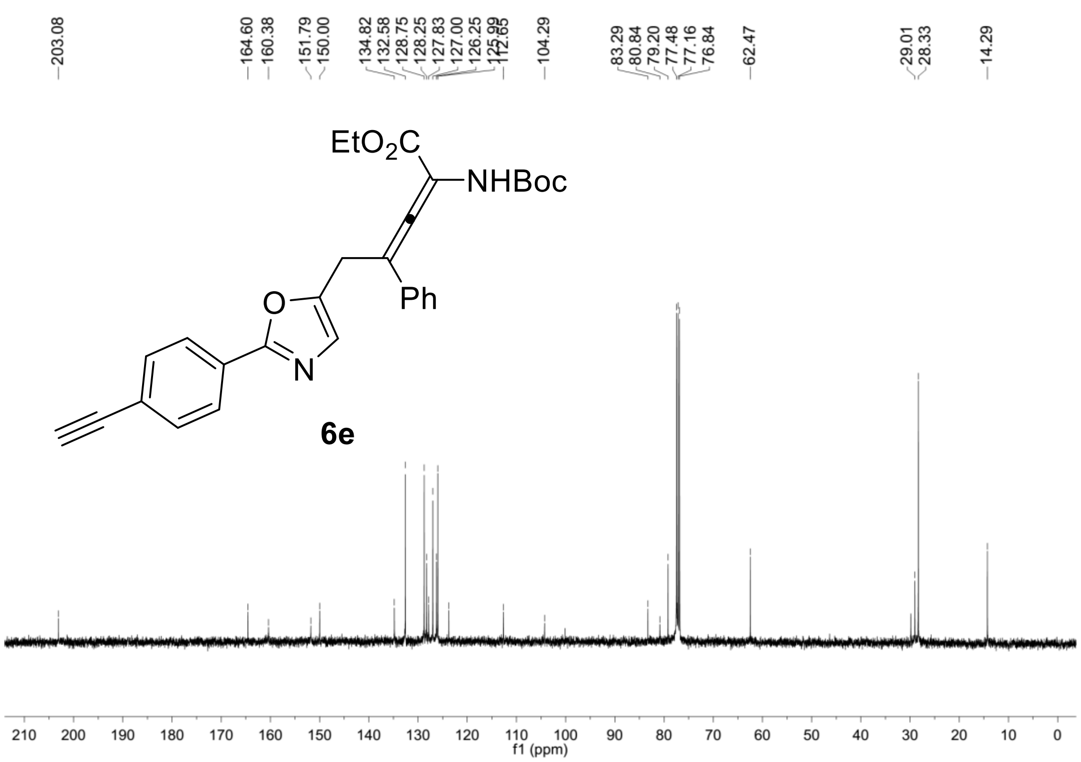
203.08
164.60
160.38
151.79
150.00
134.82
132.58
128.75
128.25
127.83
127.00
126.25
125.99
112.65
104.29
83.29
80.84
79.20
77.48
77.16
76.84
62.47
29.01
28.33
14.29
EtO2C
NHBoc
Ph
O
N
6e
f1 (ppm)

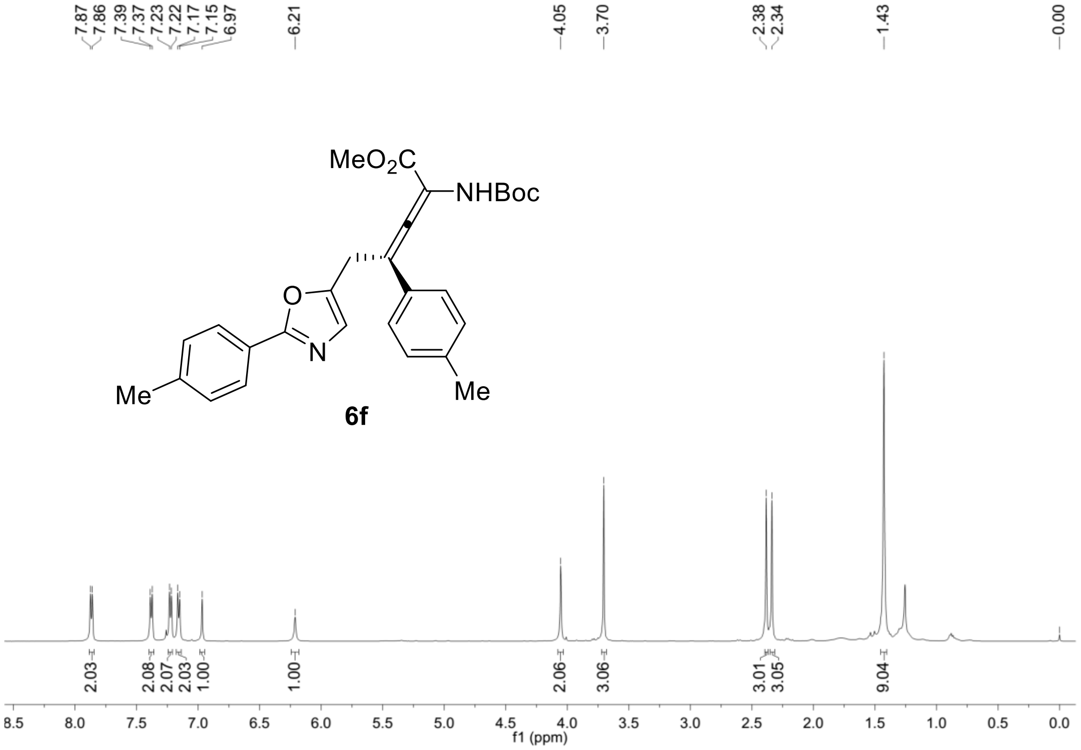
7.87
7.86
7.39
7.37
7.23
7.22
7.17
7.15
6.97
6.21
4.05
3.70
2.38
2.34
1.43
0.00
MeO2C
NHBoc
O
N
Me
Me
6f
2.03
2.08
2.07
2.03
1.00
1.00
2.06
3.06
3.01
3.05
9.04
8.5 8.0 7.5 7.0 6.5 6.0 5.5 5.0 4.5 4.0 3.5 3.0 2.5 2.0 1.5 1.0 0.5 0.0
f1 (ppm)

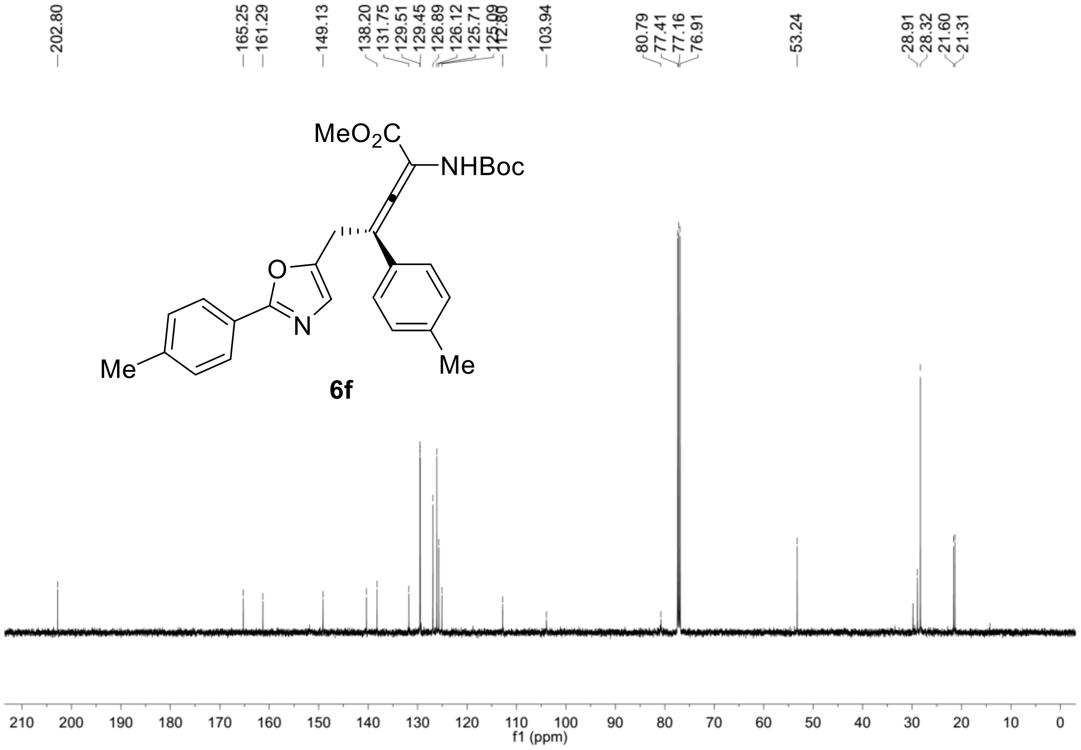
202.80
165.25
161.29
149.13
138.20
131.75
129.51
129.45
126.89
126.12
125.71
125.09
112.80
103.94
80.79
77.41
77.16
76.91
53.24
28.91
28.32
21.60
21.31
MeO2C
NHBoc
O
N
Me
Me
6f
210 200 190 180 170 160 150 140 130 120 110 100 90 80 70 60 50 40 30 20 10 0
f1 (ppm)

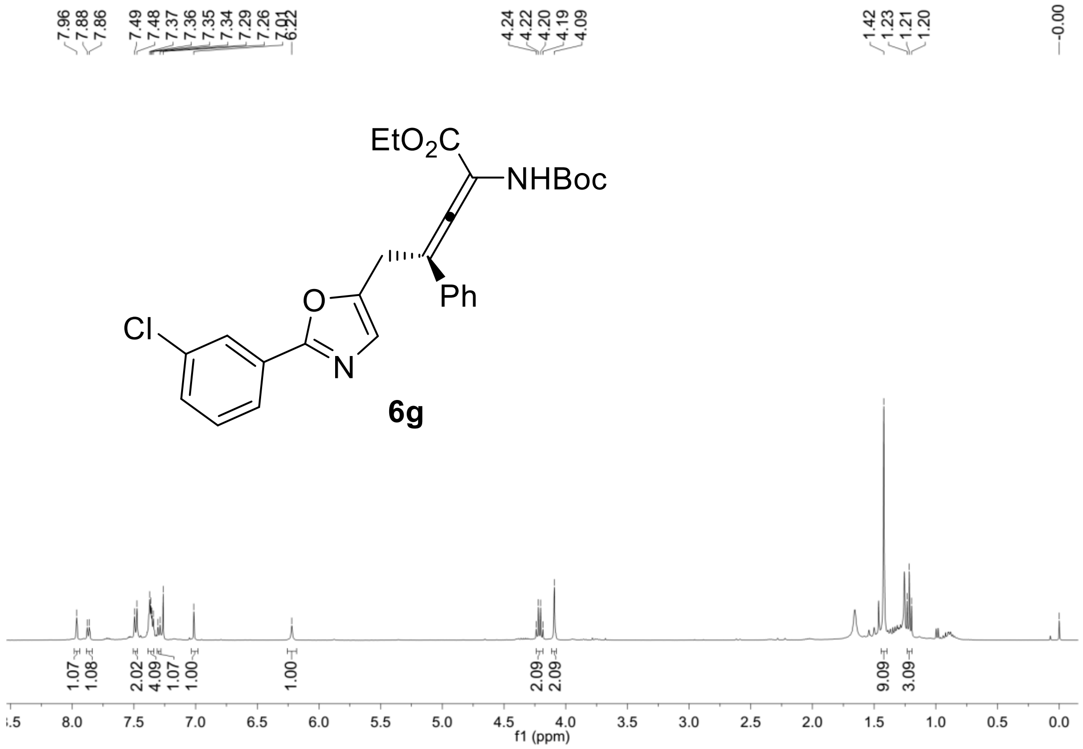
7.96 7.88 7.86 7.49 7.48 7.37 7.36 7.35 7.34 7.29 7.26 7.01 6.22
4.24 4.22 4.20 4.19 4.09
1.42 1.23 1.21 1.20
-0.00
EtO2C
NHBoc
Ph
O
N
Cl
6g
1.07 1.08 2.02 4.09 1.07 1.00 1.00 2.09 2.09 9.09 3.09
f1 (ppm)

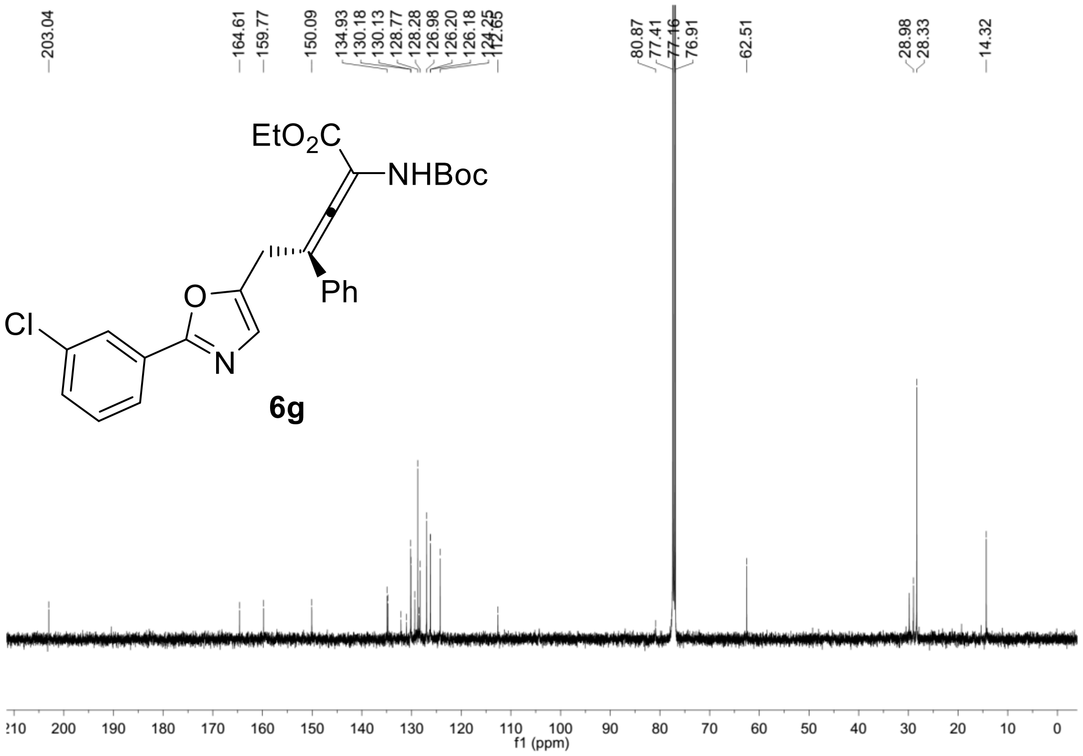
203.04
164.61 159.77 150.09 134.93 130.18 130.13 128.77 128.28 126.98 126.20 126.18 124.25 112.65
80.87 77.41 77.16 76.91 62.51
28.98 28.33 14.32
EtO2C
NHBoc
Ph
O
N
Cl
6g
f1 (ppm)

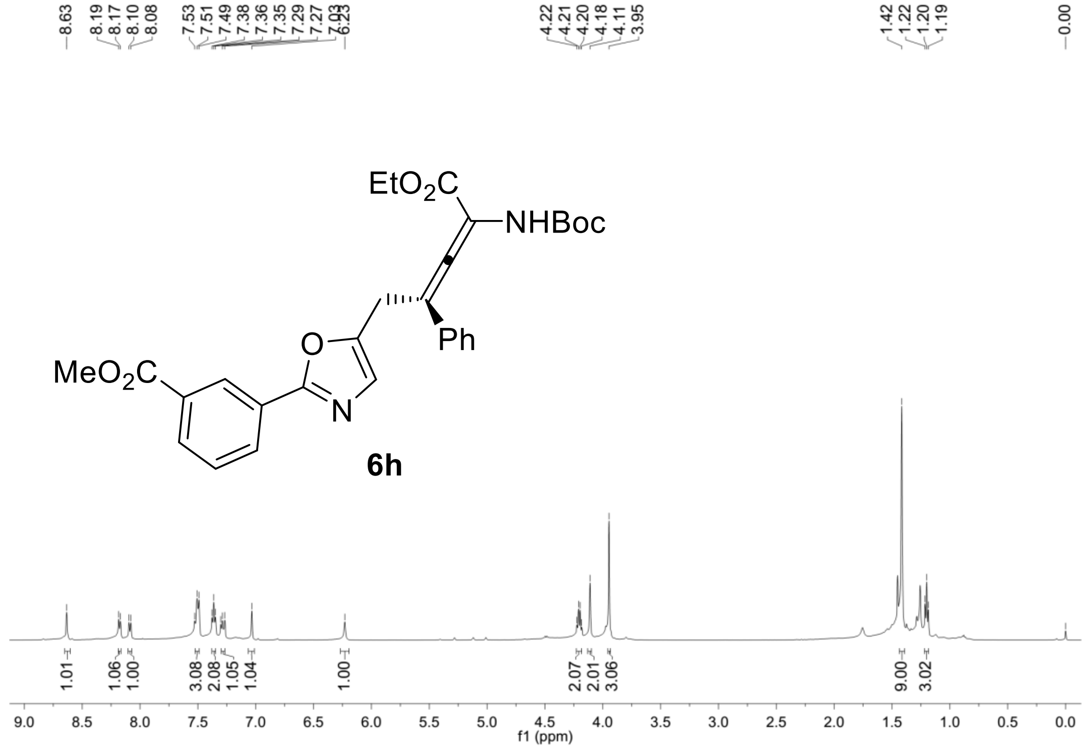
8.63 8.19 8.17 8.10 8.08 7.53 7.51 7.49 7.38 7.36 7.35 7.29 7.27 7.03 6.23
4.22 4.21 4.20 4.18 4.11 3.95
1.42 1.22 1.20 1.19
0.00
EtO2C
NHBoc
Ph
MeO2C
O
N
6h
1.01 1.06 1.00 3.08 2.08 1.05 1.04 1.00 2.07 2.01 3.06 9.00 3.02
f1 (ppm)

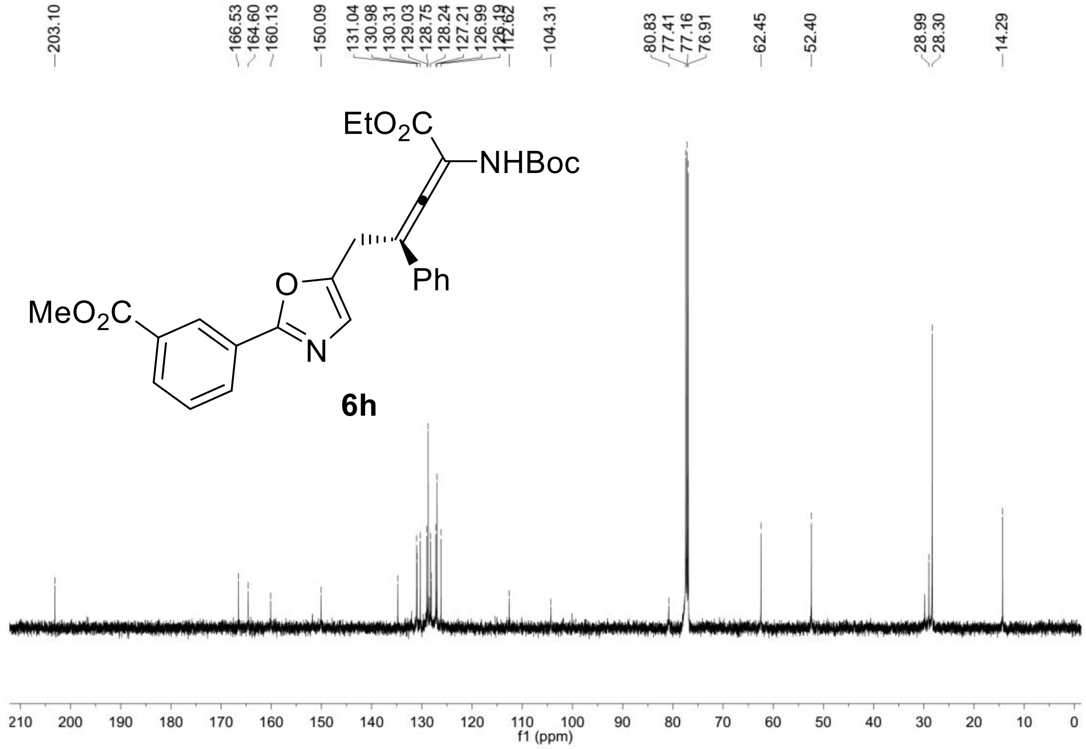
203.10
166.53 164.60 160.13 150.09
131.04 130.98 130.31 129.03 128.75 128.24 127.21 126.99 126.19 112.62
104.31
80.83 77.41 77.16 76.91
62.45 52.40 28.99 28.30 14.29
EtO2C
NHBoc
Ph
MeO2C
O
N
6h
f1 (ppm)

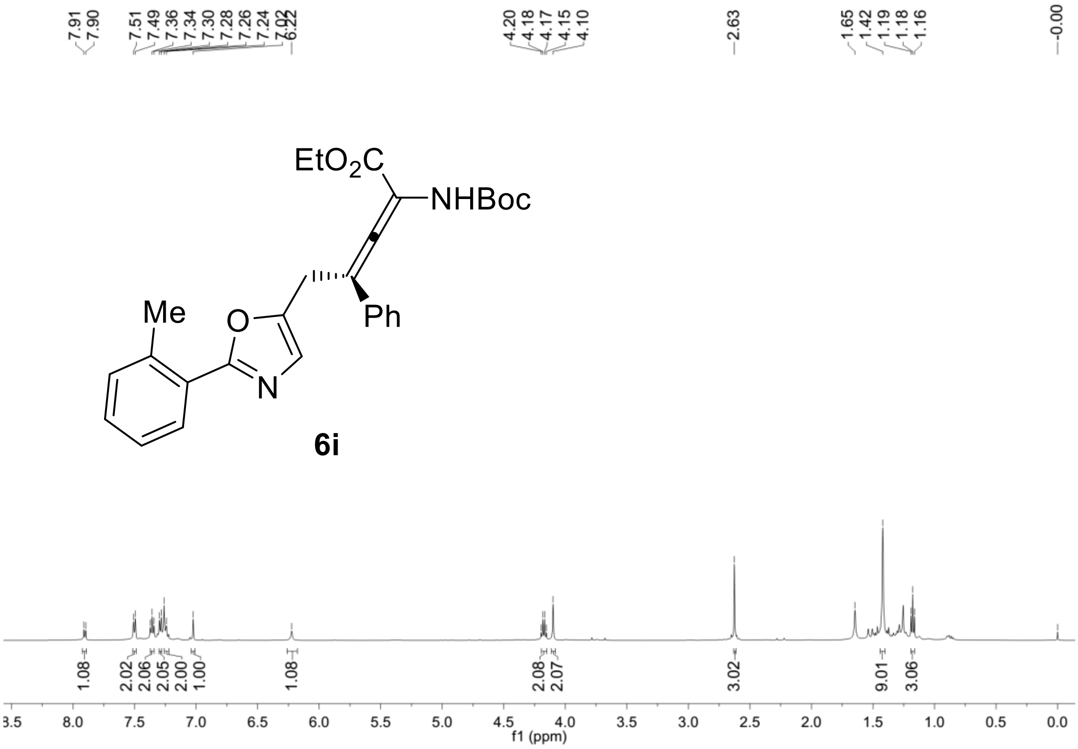
7.91
7.90
7.51
7.49
7.36
7.34
7.30
7.28
7.26
7.24
7.02
6.22
4.20
4.18
4.17
4.15
4.10
2.63
1.65
1.42
1.19
1.18
1.16
-0.00
EtO2C
NHBoc
Me
O
N
Ph
6i
1.08
2.02
2.06
2.05
2.00
1.00
1.08
2.08
2.07
3.02
9.01
3.06
f1 (ppm)

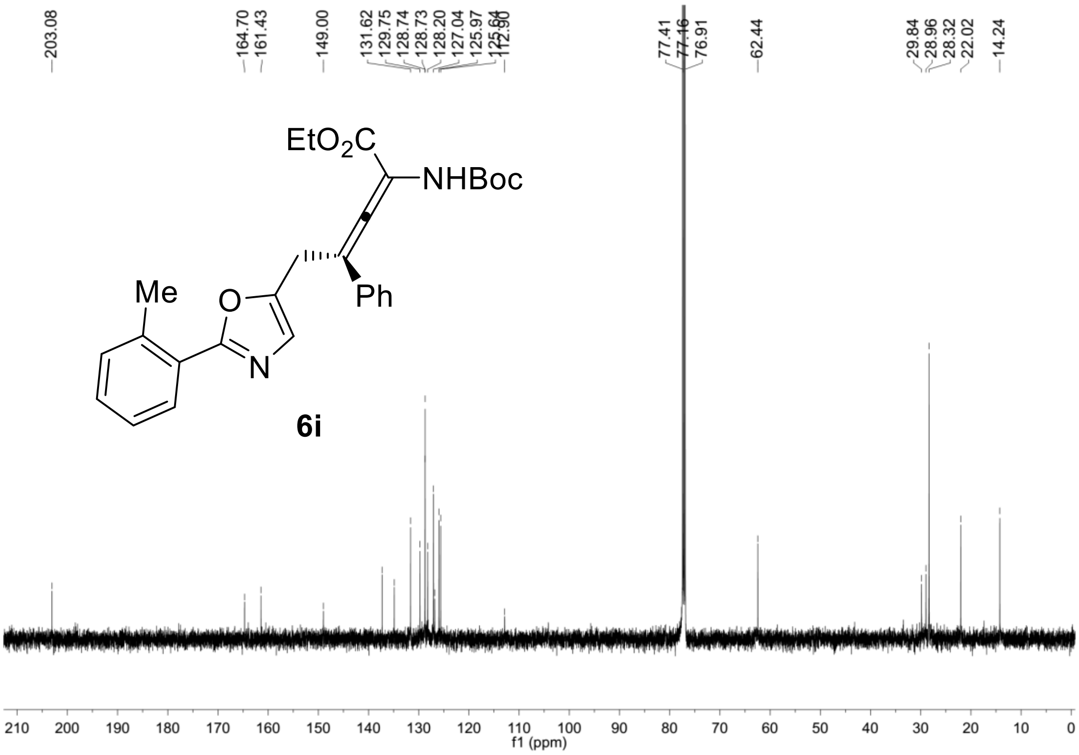
203.08
164.70
161.43
149.00
131.62
129.75
128.74
128.73
128.20
127.04
125.97
125.64
112.90
77.41
77.16
76.91
62.44
29.84
28.96
28.32
22.02
14.24
EtO2C
NHBoc
Me
O
N
Ph
6i
f1 (ppm)

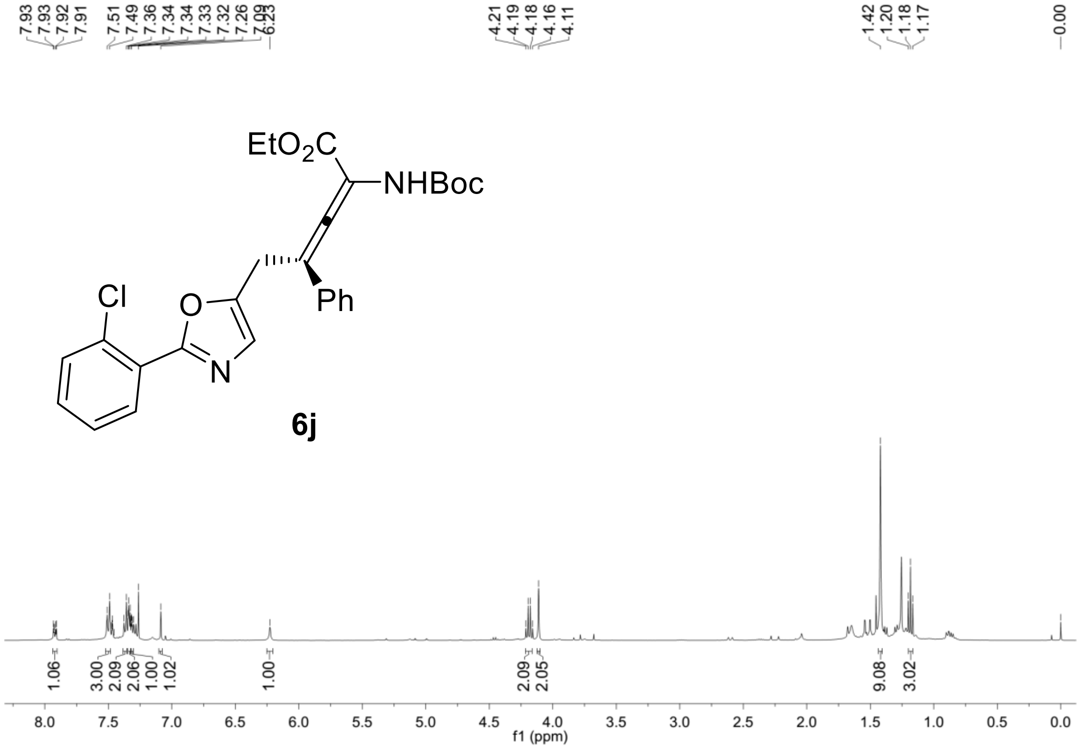
7.93
7.93
7.92
7.91
7.51
7.49
7.36
7.34
7.34
7.33
7.32
7.26
7.09
6.23
4.21
4.19
4.18
4.16
4.11
1.42
1.20
1.18
1.17
0.00
EtO2C
NHBoc
Ph
Cl
O
N
6j
1.06
3.00
2.09
2.06
1.00
1.02
1.00
2.09
2.05
9.08
3.02
f1 (ppm)

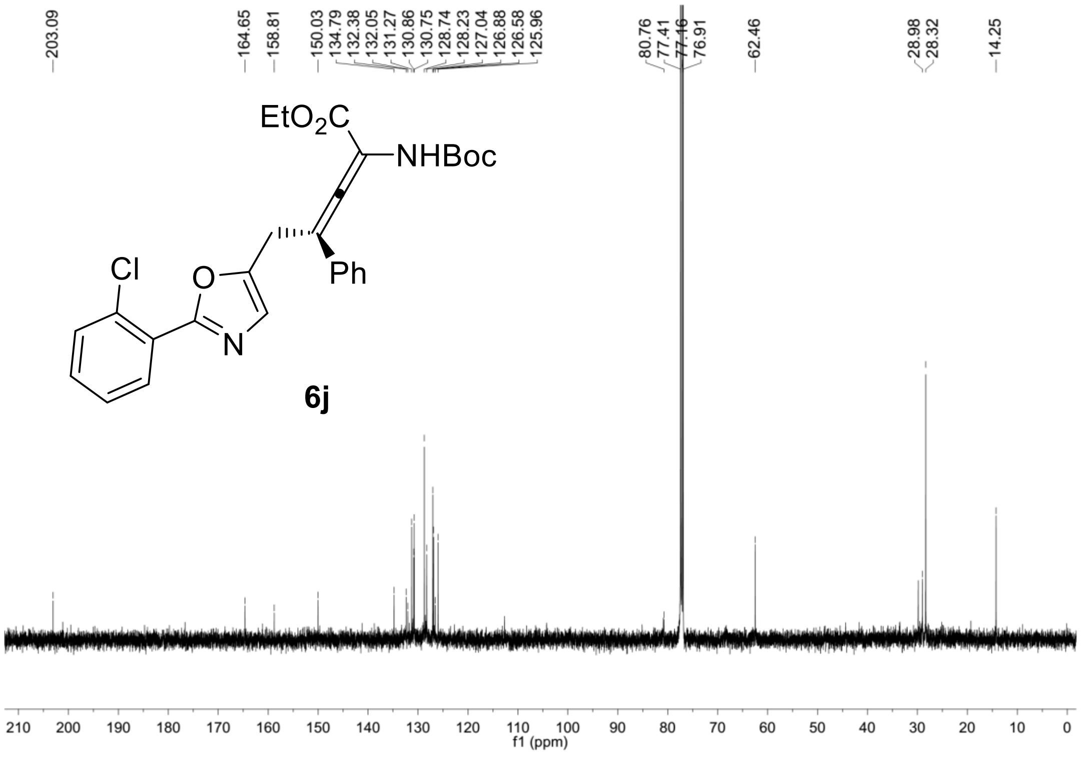
203.09
164.65
158.81
150.03
134.79
132.38
132.05
131.27
130.86
130.75
128.74
128.23
127.04
126.88
126.58
125.96
80.76
77.41
77.16
76.91
62.46
28.98
28.32
14.25
EtO2C
NHBoc
Ph
Cl
O
N
6j
f1 (ppm)

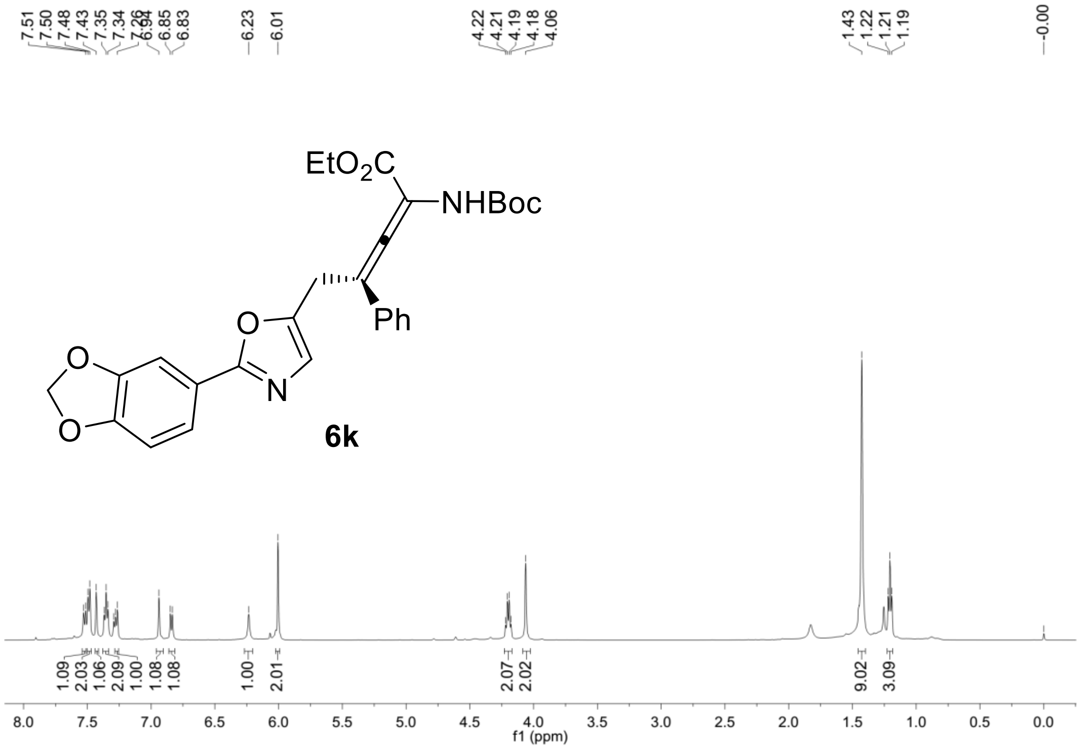

7.51
7.50
7.48
7.43
7.35
7.34
7.26
6.94
6.85
6.83
6.23
6.01
4.22
4.21
4.19
4.18
4.06
1.43
1.22
1.21
1.19
-0.00
EtO2C
NHBoc
Ph
O
N
6k
1.09
2.03
1.06
2.09
1.00
1.08
1.08
1.00
2.01
2.07
2.02
9.02
3.09
f1 (ppm)

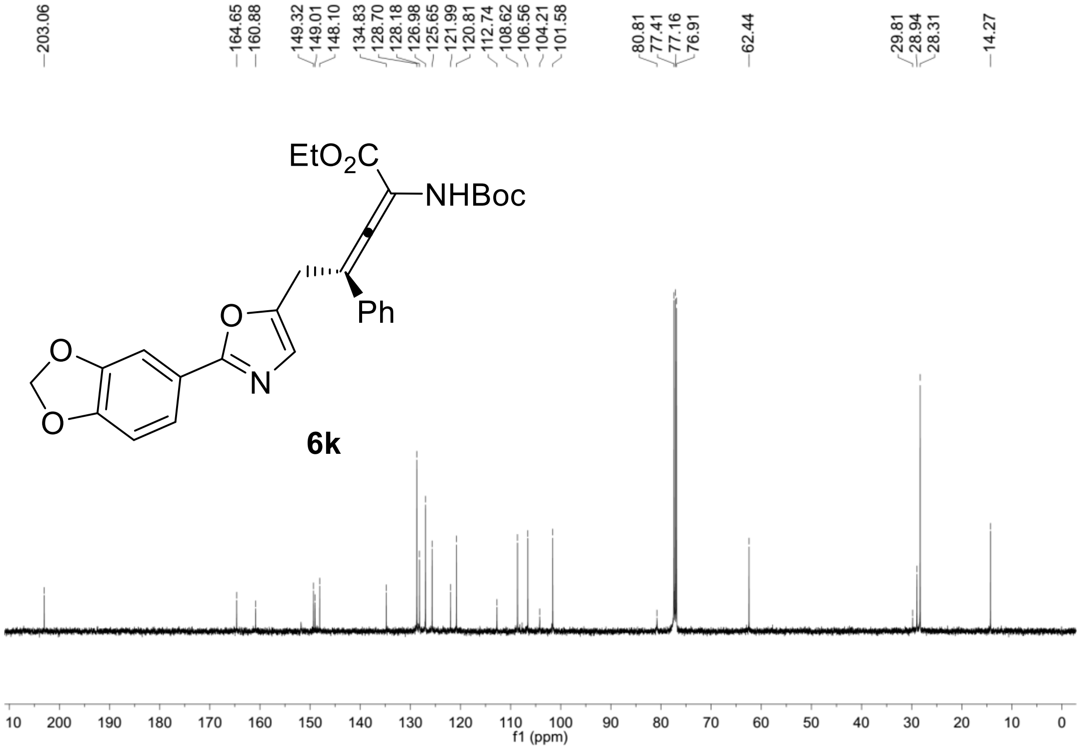

203.06
164.65
160.88
149.32
149.01
148.10
134.83
128.70
128.18
126.98
125.65
121.99
120.81
112.74
108.62
106.56
104.21
101.58
80.81
77.41
77.16
76.91
62.44
29.81
28.94
28.31
14.27
EtO2C
NHBoc
Ph
O
N
6k
f1 (ppm)

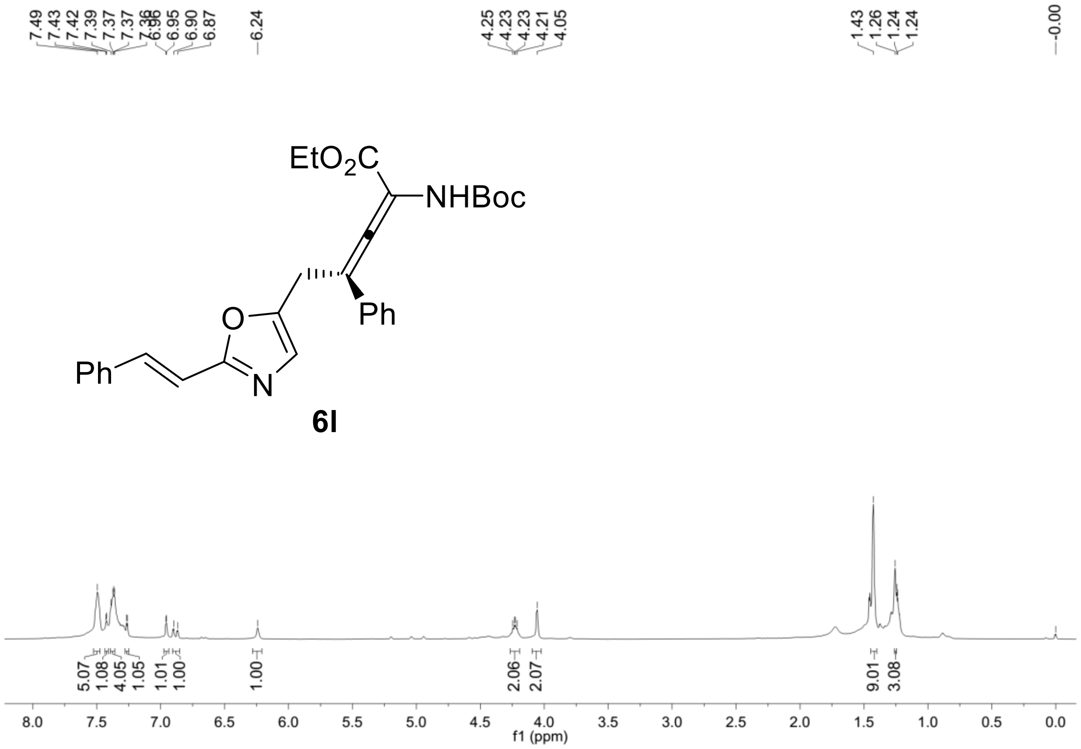
7.49 7.43 7.42 7.39 7.37 7.37 7.36 6.95 6.90 6.87 6.24 4.25 4.23 4.23 4.21 4.05 1.43 1.26 1.24 1.24 0.00
EtO2C
NHBoc
Ph
O
N
Ph
6l
5.07 1.08 4.05 1.05 1.01 1.00 1.00 2.06 2.07 9.01 3.08
8.0 7.5 7.0 6.5 6.0 5.5 5.0 4.5 4.0 3.5 3.0 2.5 2.0 1.5 1.0 0.5 0.0
f1 (ppm)

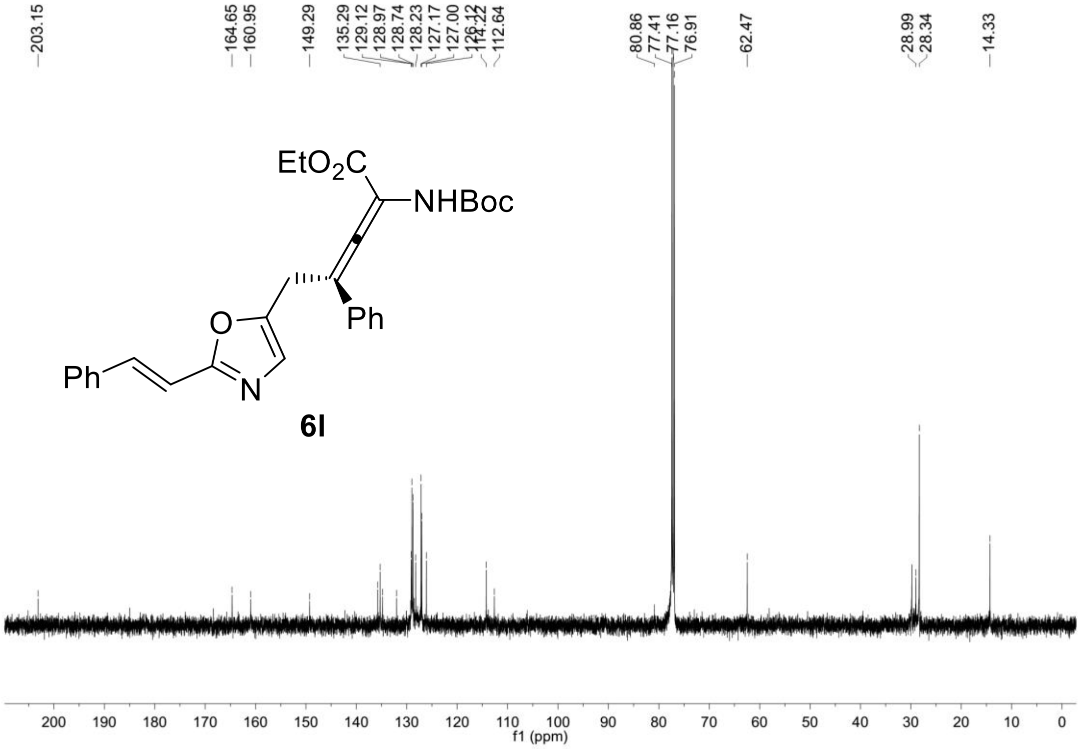
203.15 164.65 160.95 149.29 135.29 129.12 128.97 128.74 128.23 127.17 127.00 126.12 114.22 112.64 80.86 77.41 77.16 76.91 62.47 28.99 28.34 14.33
EtO2C
NHBoc
Ph
O
N
Ph
6l
200 190 180 170 160 150 140 130 120 110 100 90 80 70 60 50 40 30 20 10 0
f1 (ppm)

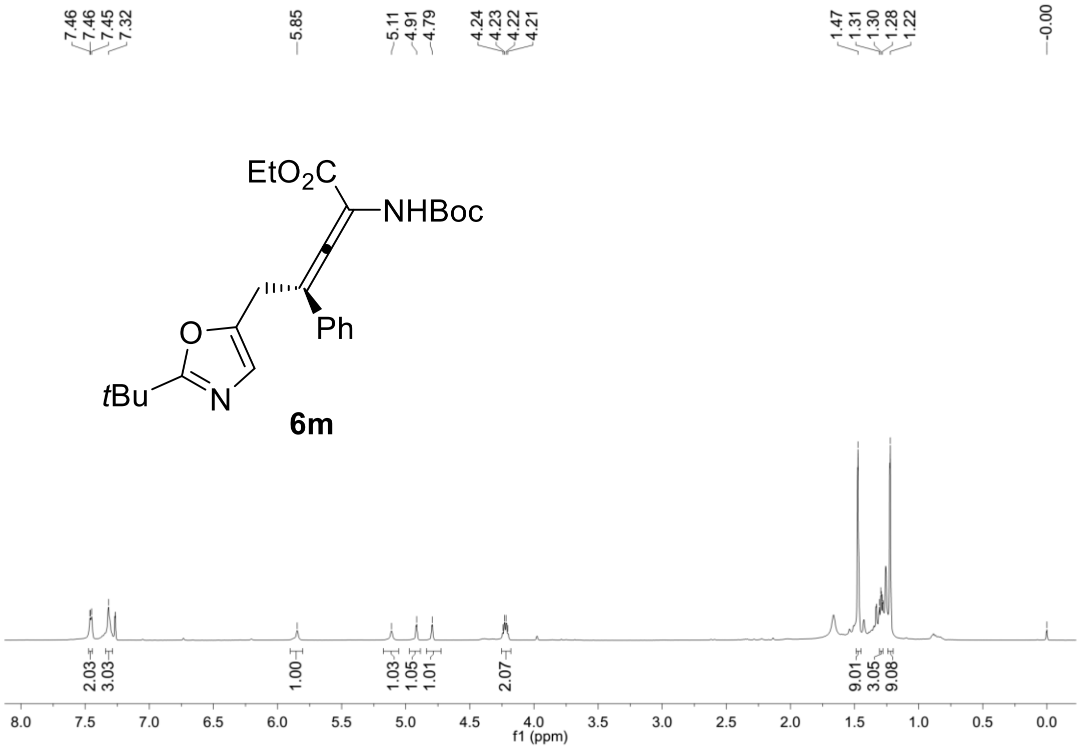
7.46
7.46
7.45
7.32
5.85
5.11
4.91
4.79
4.24
4.23
4.22
4.21
1.47
1.31
1.30
1.28
1.22
0.00
EtO2C
NHBoc
Ph
O
N
tBu
6m
2.03
3.03
1.00
1.03
1.05
1.01
2.07
9.01
3.05
9.08
f1 (ppm)

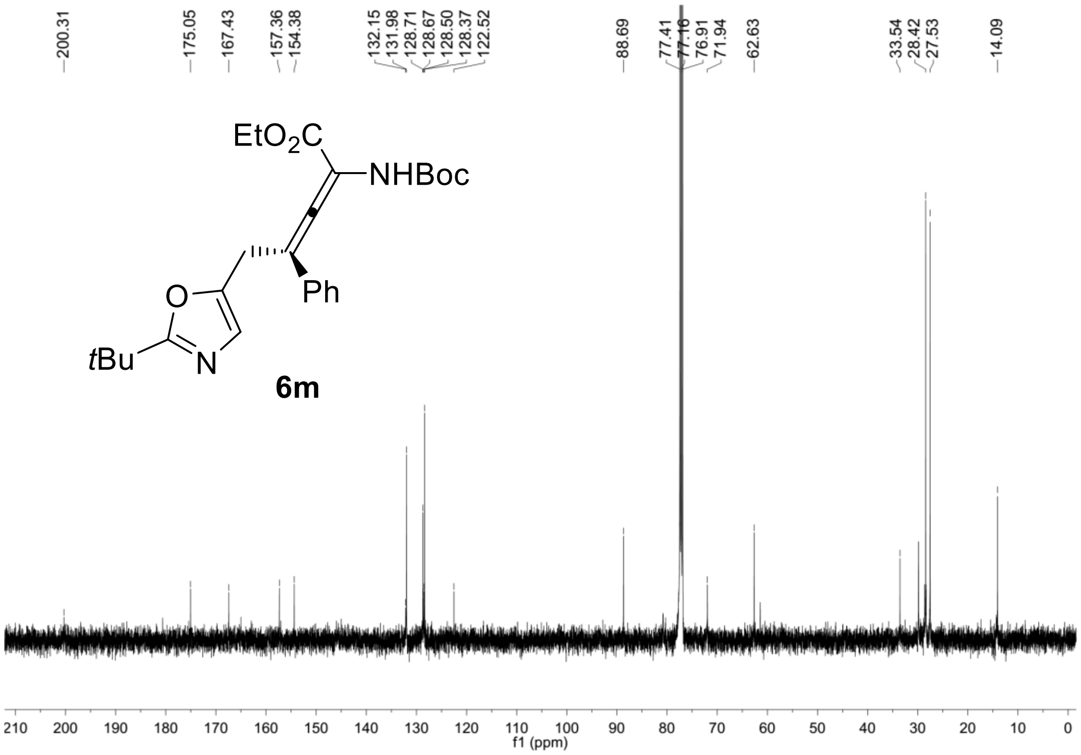
200.31
175.05
167.43
157.36
154.38
132.15
131.98
128.71
128.67
128.50
128.37
122.52
88.69
77.41
77.16
76.91
71.94
62.63
33.54
28.42
27.53
14.09
EtO2C
NHBoc
Ph
O
N
tBu
6m
f1 (ppm)

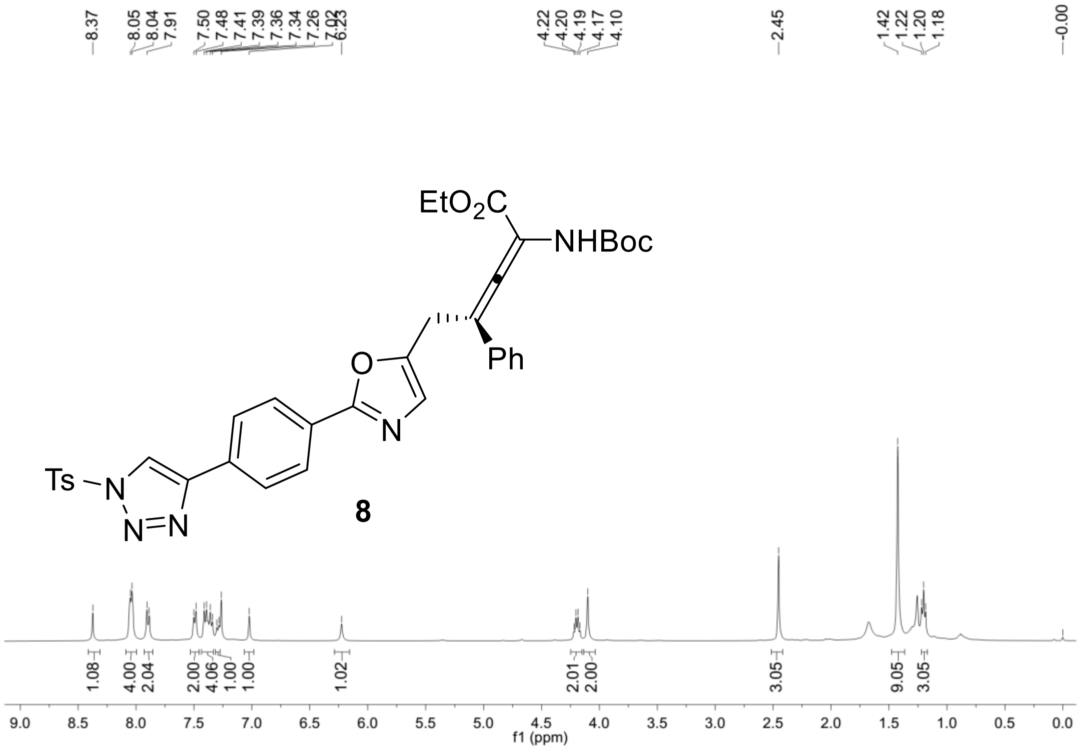
8.37 8.05 8.04 7.91 7.50 7.48 7.41 7.39 7.36 7.34 7.26 7.02 6.23
4.22 4.20 4.19 4.17 4.10
2.45
1.42 1.22 1.20 1.18
0.00
EtO2C
NHBoc
Ph
Ts
8
1.08 4.00 2.04 2.00 4.06 1.00 1.00 1.02 2.01 2.00 3.05 9.05 3.05
f1 (ppm)

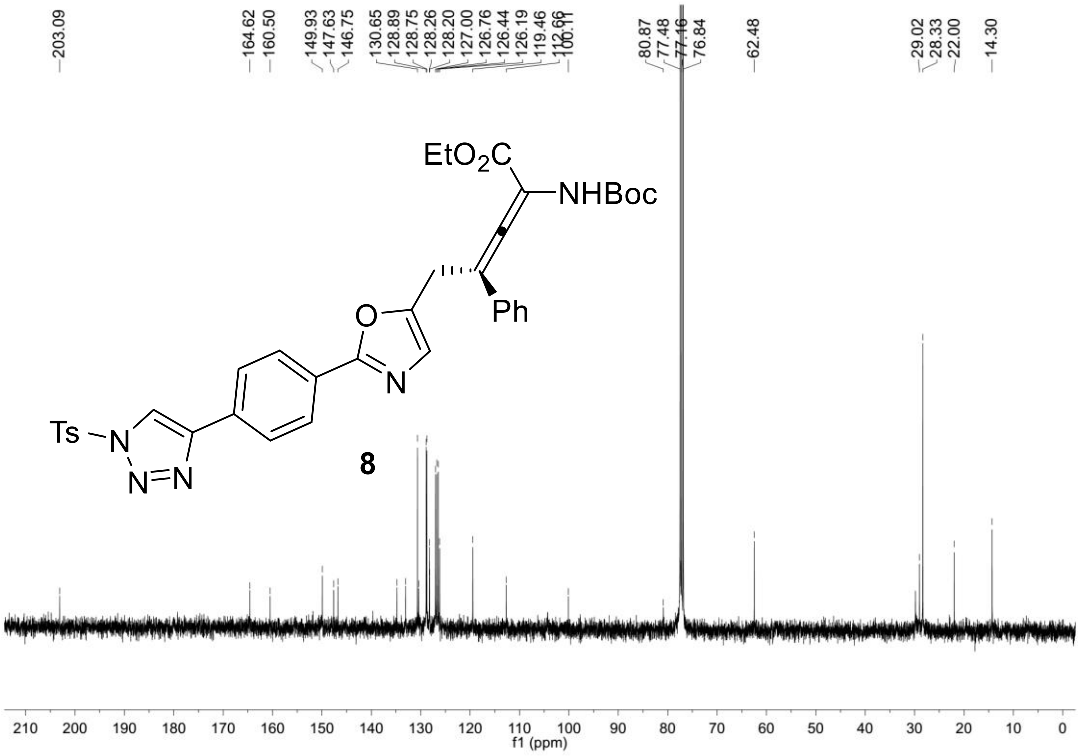
203.09
164.62 160.50
149.93 147.63 146.75
130.65 128.89 128.75 128.26 128.20 127.00 126.76 126.44 126.19 119.46 112.66 100.11
80.87 77.48 77.16 76.84
62.48
29.02 28.33 22.00
14.30
EtO2C
NHBoc
Ph
Ts
8
f1 (ppm)

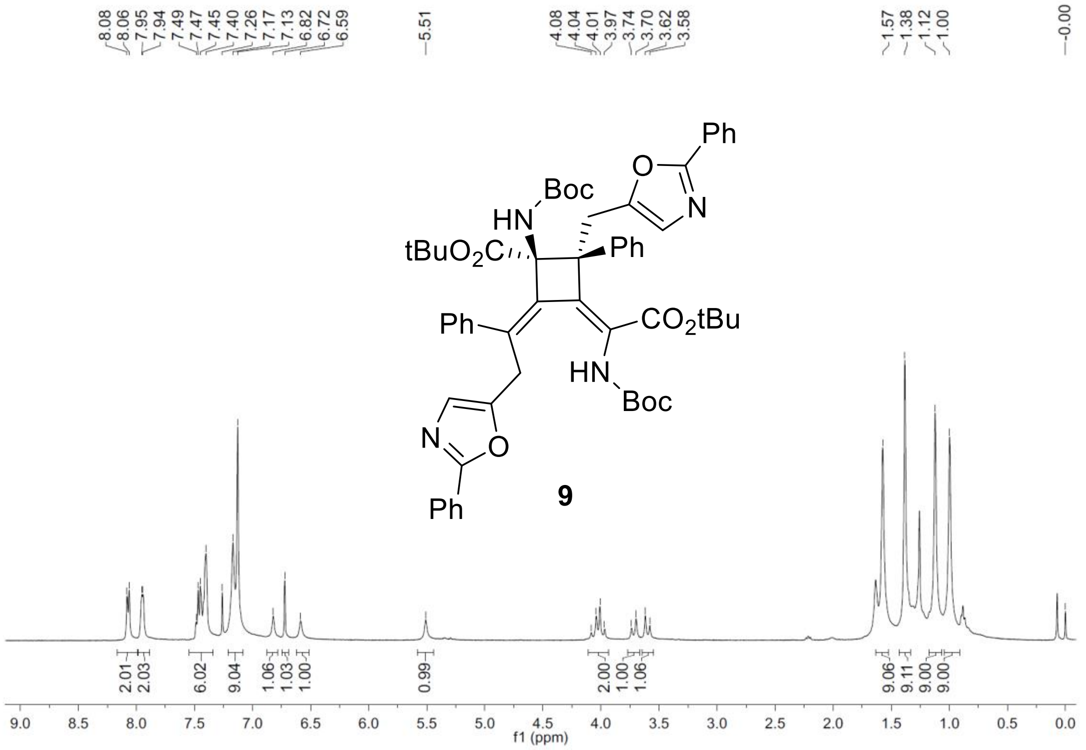
8.08 8.06 7.95 7.94 7.49 7.47 7.45 7.40 7.26 7.17 7.13 6.82 6.72 6.59
5.51
4.08 4.04 4.01 3.97 3.74 3.70 3.62 3.58
1.57 1.38 1.12 1.00
0.00
Ph
O
N
Boc
HN
tBuO2C
Ph
Ph
CO2tBu
HN
Boc
N
O
Ph
9
2.01 2.03 6.02 9.04 1.06 1.03 1.00 0.99 2.00 1.00 1.06 9.06 9.11 9.00 9.00
9.0 8.5 8.0 7.5 7.0 6.5 6.0 5.5 5.0 4.5 4.0 3.5 3.0 2.5 2.0 1.5 1.0 0.5 0.0
f1 (ppm)

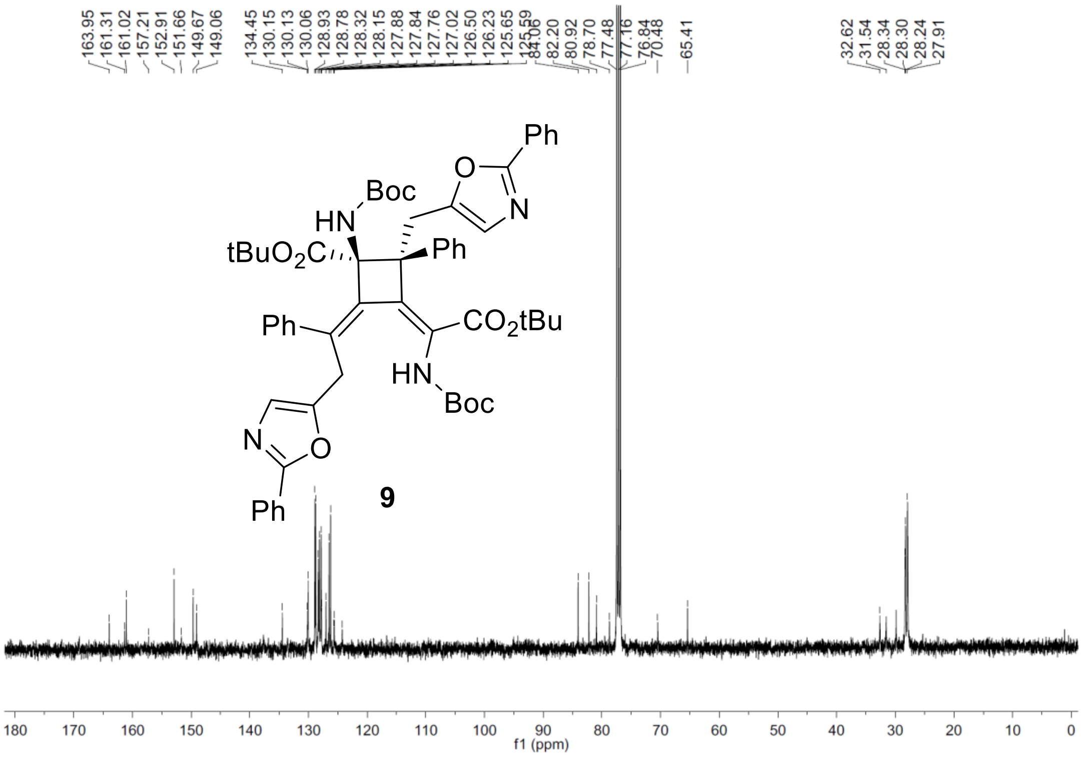
163.95 161.31 161.02 157.21 152.91 151.66 149.67 149.06
134.45 130.15 130.13 130.06 128.93 128.78 128.32 128.15 127.88 127.84 127.76 127.02 126.50 126.23 125.65 125.59
84.06 82.20 80.92 78.70 77.48 77.16 76.84 70.48
65.41
32.62 31.54 28.34 28.30 28.24 27.91
Ph
O
N
Boc
HN
tBuO2C
Ph
Ph
CO2tBu
HN
Boc
N
O
Ph
9
180 170 160 150 140 130 120 110 100 90 80 70 60 50 40 30 20 10 0
f1 (ppm)

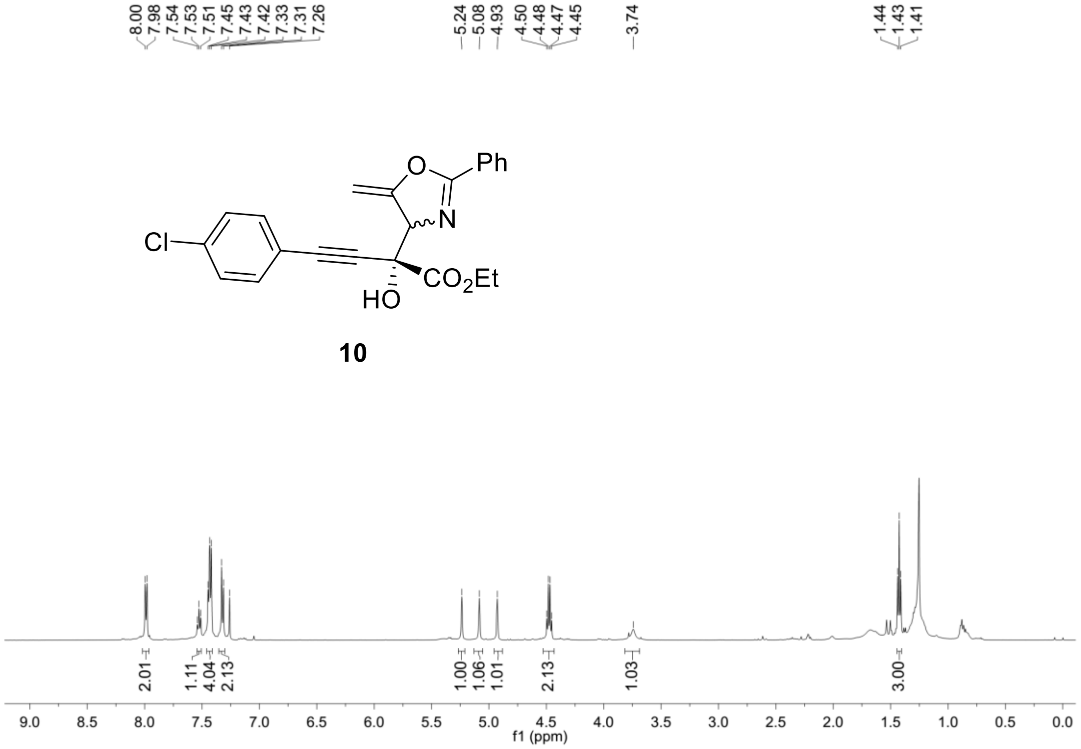
8.00
7.98
7.54
7.53
7.51
7.45
7.43
7.42
7.33
7.31
7.26
5.24
5.08
4.93
4.50
4.48
4.47
4.45
3.74
1.44
1.43
1.41
O
Ph
N
Cl
CO_2Et
HO
10
2.01
1.11
4.04
2.13
1.00
1.06
1.01
2.13
1.03
3.00
9.0 8.5 8.0 7.5 7.0 6.5 6.0 5.5 5.0 4.5 4.0 3.5 3.0 2.5 2.0 1.5 1.0 0.5 0.0
f1 (ppm)

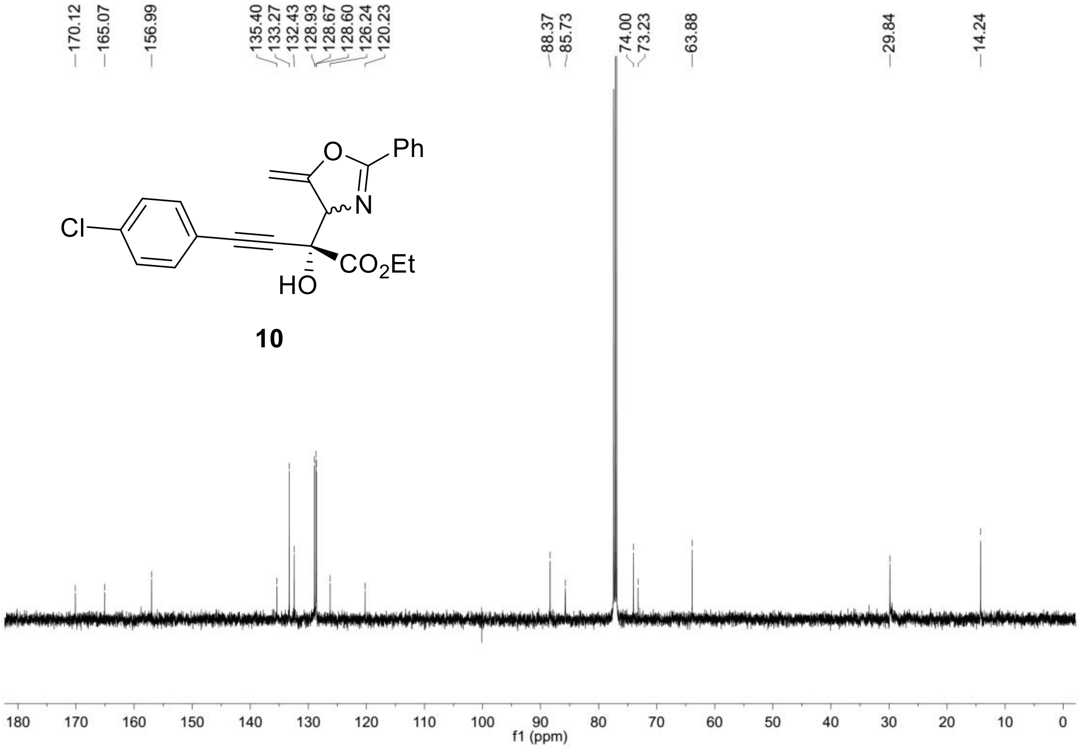
170.12
165.07
156.99
135.40
133.27
132.43
128.93
128.67
128.60
126.24
120.23
88.37
85.73
74.00
73.23
63.88
29.84
14.24
O
Ph
N
Cl
CO_2Et
HO
10
180 170 160 150 140 130 120 110 100 90 80 70 60 50 40 30 20 10 0
f1 (ppm)

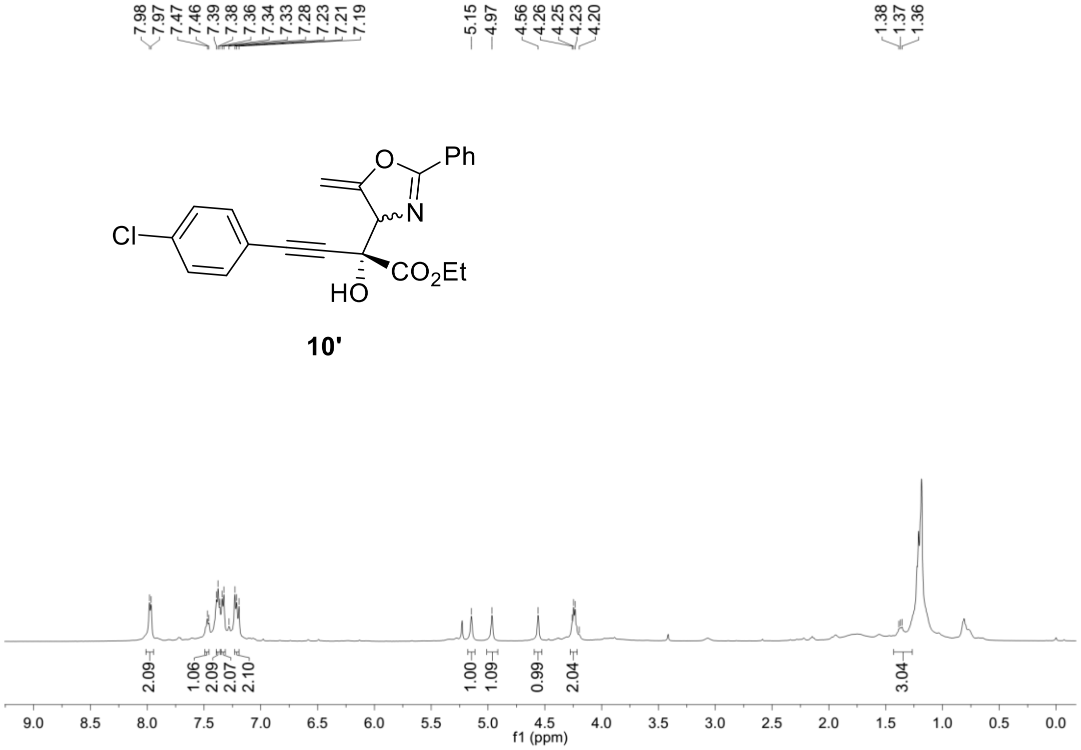
7.98
7.97
7.47
7.46
7.39
7.38
7.36
7.34
7.33
7.28
7.23
7.21
7.19
5.15
4.97
4.56
4.26
4.25
4.23
4.20
1.38
1.37
1.36
O
Ph
N
Cl
CO2Et
HO
10'
2.09
1.06
2.09
2.07
2.10
1.00
1.09
0.99
2.04
3.04
f1 (ppm)

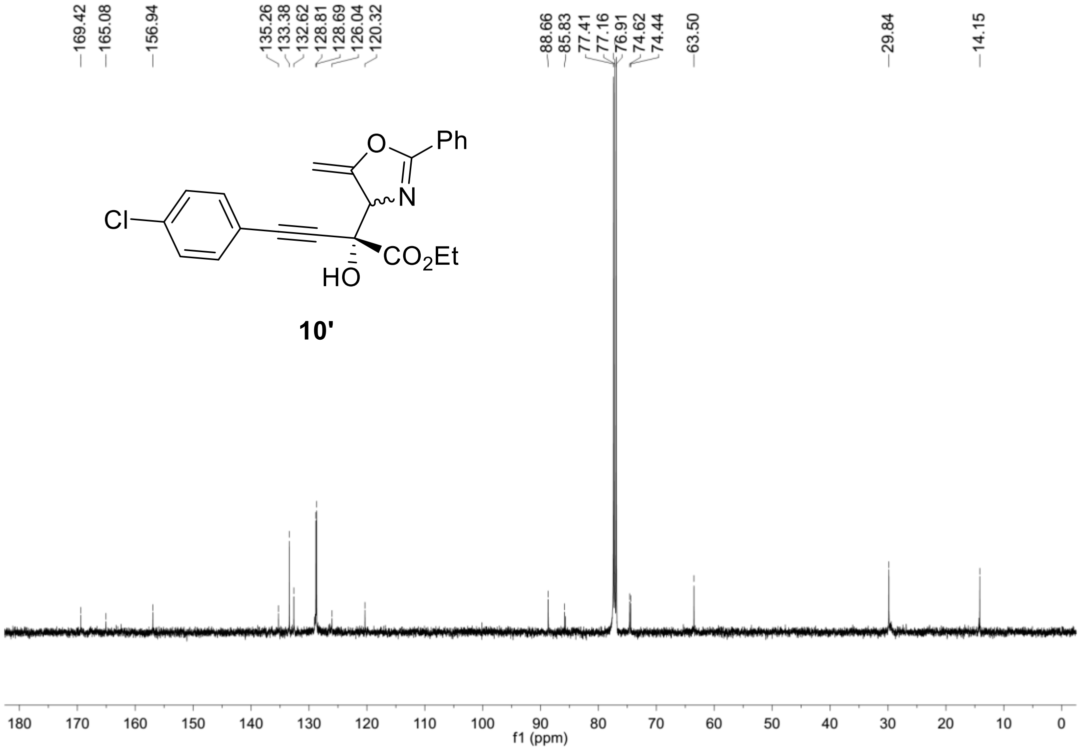
169.42
165.08
156.94
135.26
133.38
132.62
128.81
128.69
126.04
120.32
88.66
85.83
77.41
77.16
76.91
74.62
74.44
63.50
29.84
14.15
O
Ph
N
Cl
CO2Et
HO
10'
f1 (ppm)

Condition: hexane: 2-propanol = 95:05

Flow rate = 1.0 mL/min, λ = 254 nm, Chiral OD-H.

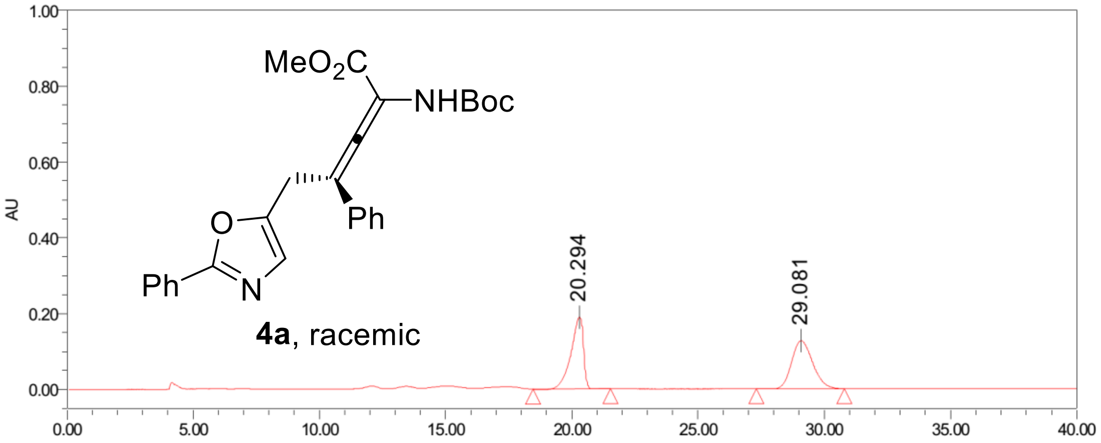

Entry	Retention Time/min	Area	Height	Area(%)
1	20.294	6441668	188930	46.92
2	29.081	7287921	126853	53.08

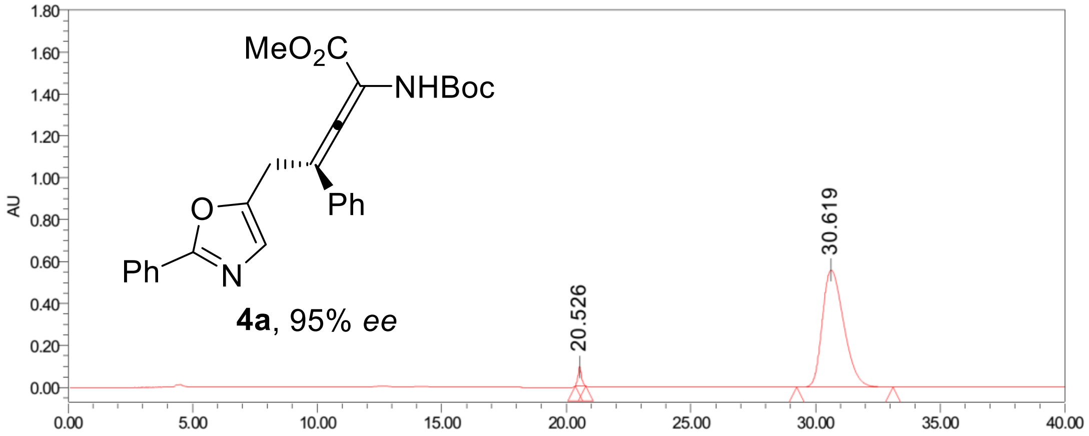

Entry	Retention Time/min	Area	Height	Area(%)
1	20.526	931923	92755	2.71
2	30.619	33419807	558738	97.29

Condition: hexane: 2-propanol = 95:05

Flow rate = 1.0 mL/min, λ = 254 nm, Chiral OD-H.

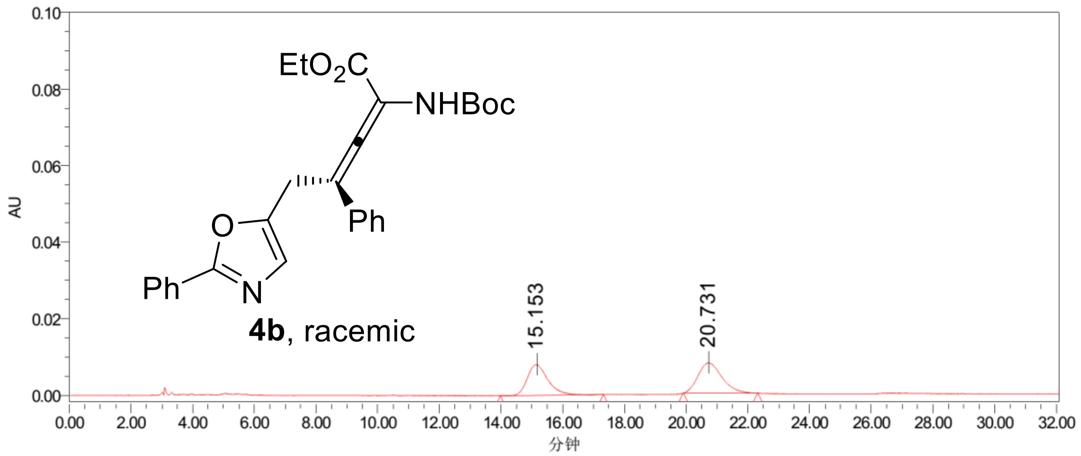

Entry	Retention Time/min	Area	Height	Area(%)
1	15.153	368847	8007	46.47
2	20.731	424899	7925	53.53

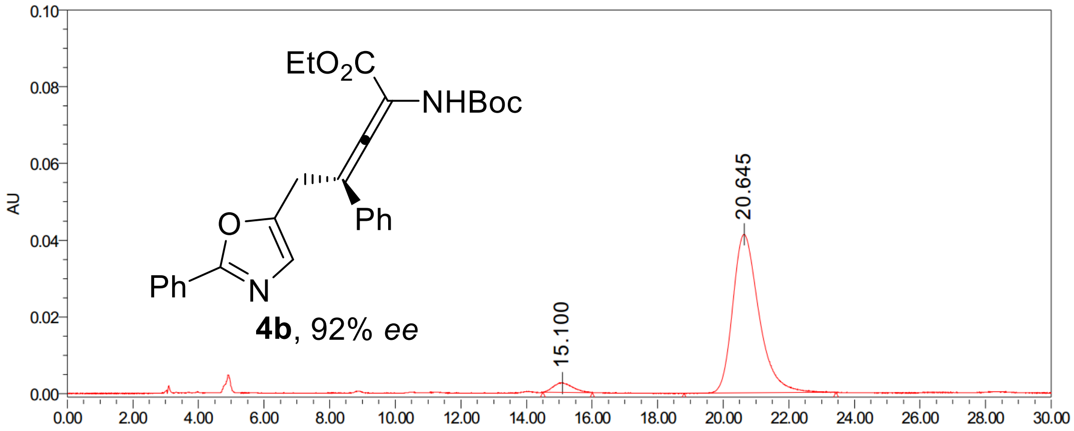

Entry	Retention Time/min	Area	Height	Area(%)
1	15.100	100649	2468	4.19
2	20.645	2303100	41329	95.81

Condition: hexane: 2-propanol = 95:05

Flow rate = 1.0 mL/min, λ = 254 nm, Chiral OD-H.

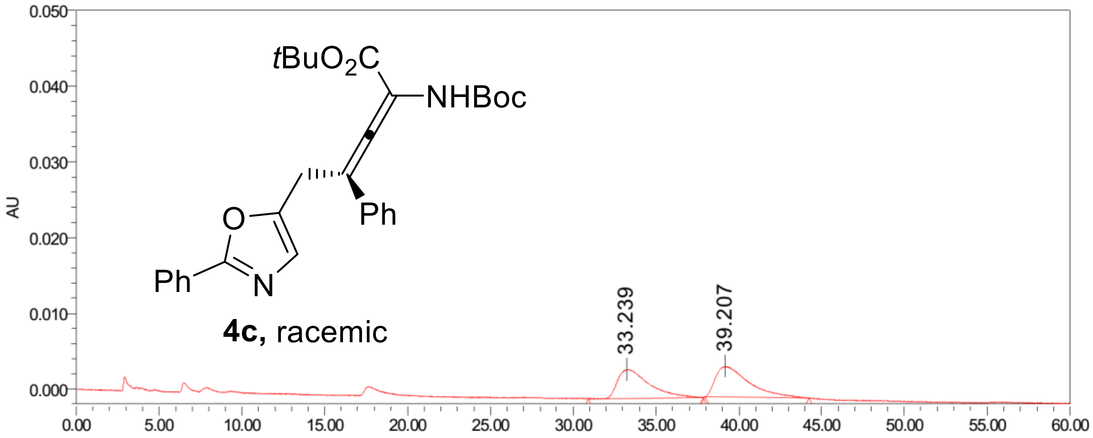

Entry	Retention Time/min	Area	Height	Area(%)
1	33.239	506185	3815	47.38
2	39.207	562192	3994	52.62

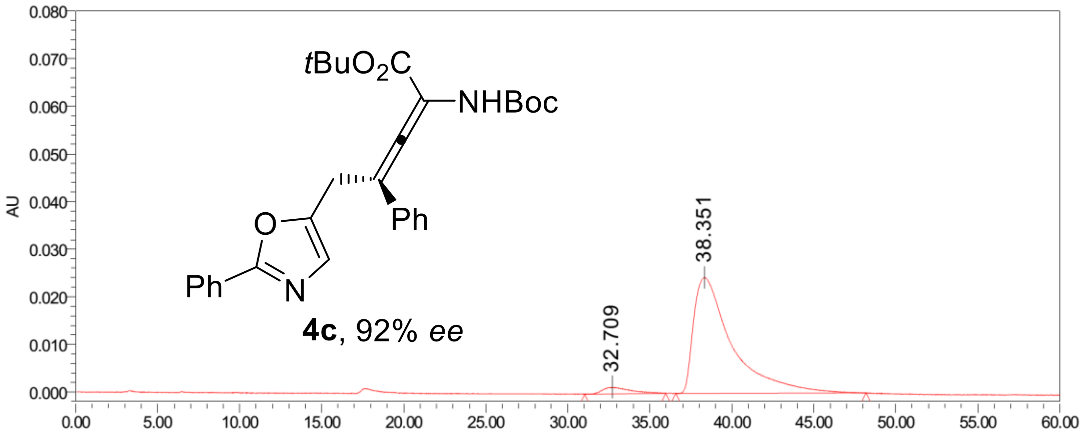

Entry	Retention Time/min	Area	Height	Area(%)
1	32.709	157679	1368	3.82
2	38.351	3973144	24345	96.18

Condition: hexane: 2-propanol = 95:05

Flow rate = 1.0 mL/min, λ = 254 nm, Chiral OD-H.

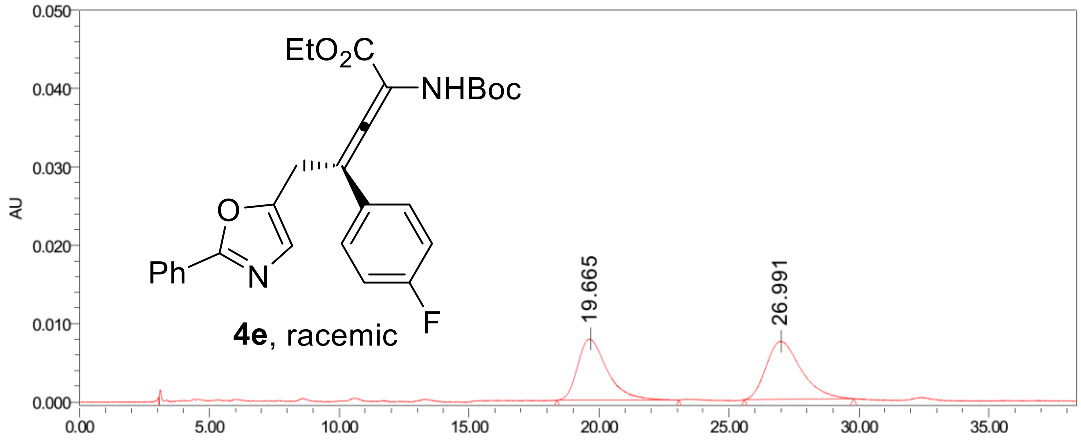

Entry	Retention Time/min	Area	Height	Area(%)
1	19.665	616889	7838	47.20
2	26.991	690195	7430	52.80

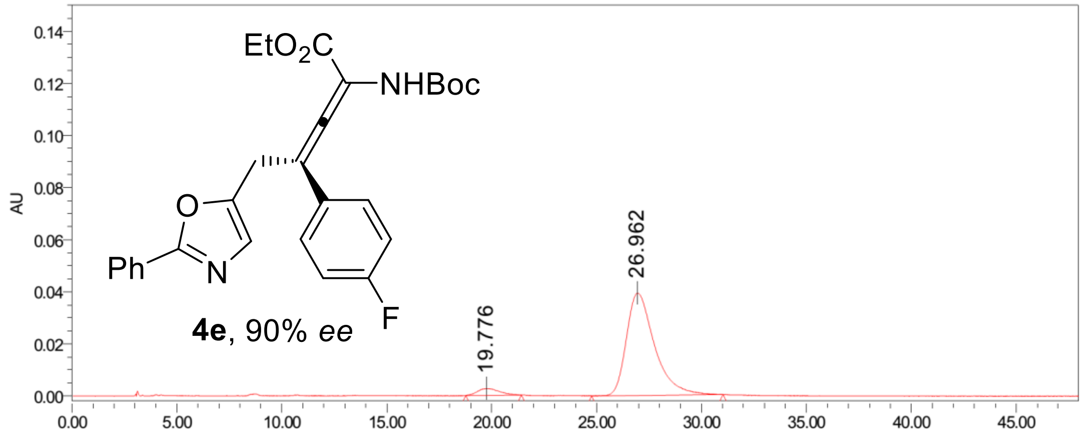

Entry	Retention Time/min	Area	Height	Area(%)
1	19.776	191001	2684	4.95
2	26.962	3668777	39327	95.05

Condition: hexane: 2-propanol = 95:05

Flow rate = 1.0 mL/min, λ = 254 nm, Chiral OD-H.

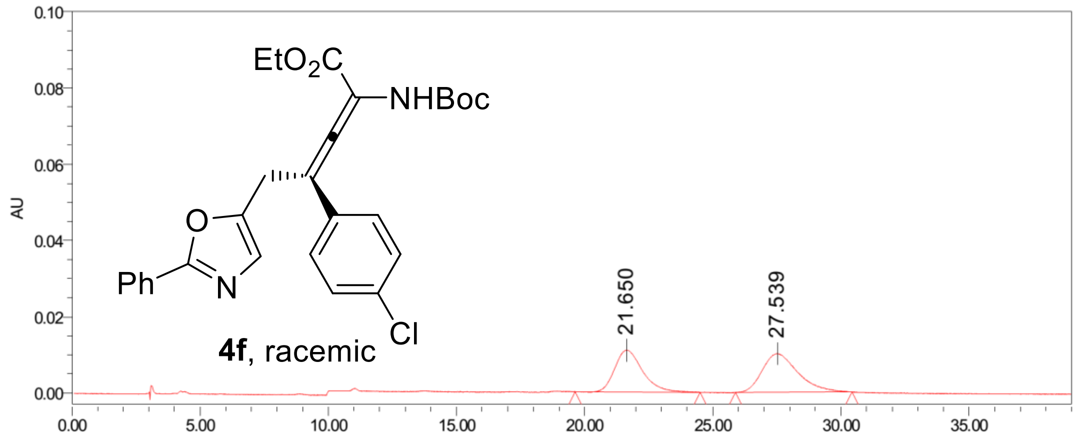

Entry	Retention Time/min	Area	Height	Area(%)
1	21.650	826839	10959	45.67
2	27.539	983800	10159	54.33

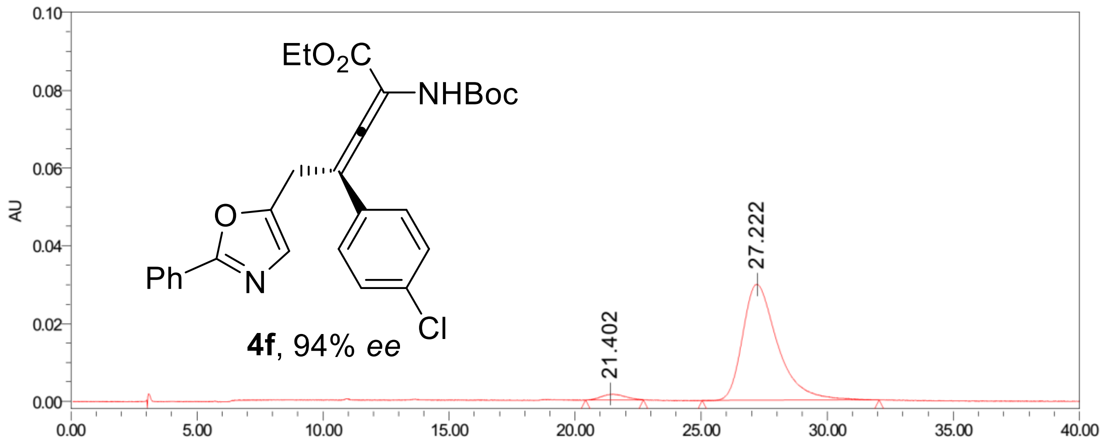

Entry	Retention Time/min	Area	Height	Area(%)
1	21.402	94684	1521	3.17
2	27.222	2895048	29852	96.83

Condition: hexane: 2-propanol = 95:05

Flow rate = 1.0 mL/min, λ = 254 nm, Chiral OD-H.

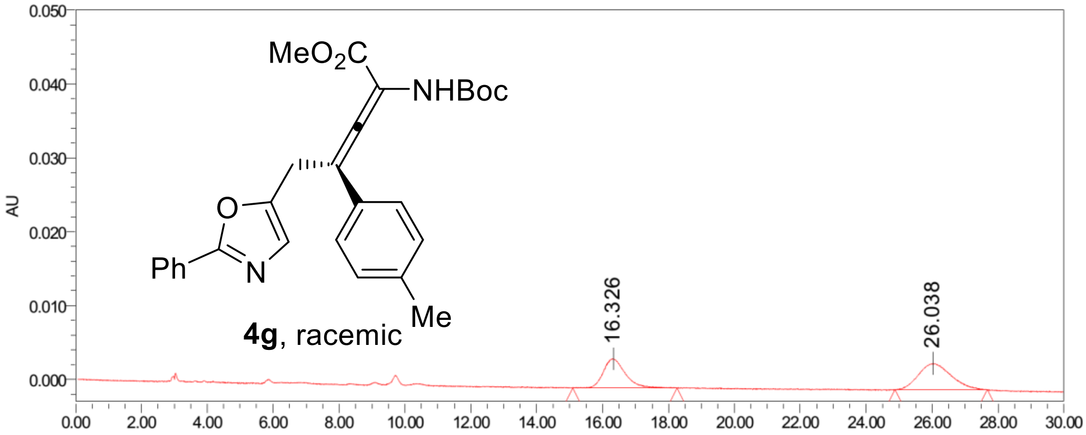

Entry	Retention Time/min	Area	Height	Area(%)
1	16.326	183681	3966	43.32
2	26.038	240329	3542	56.68

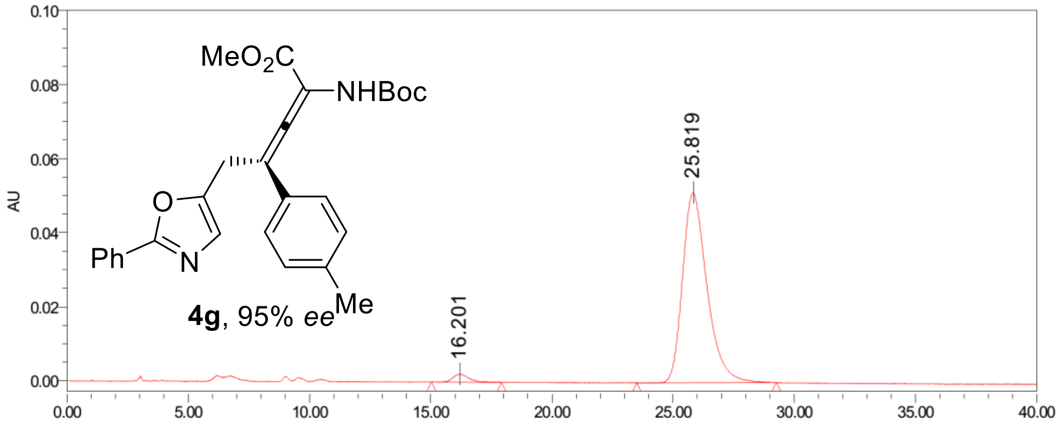

Entry	Retention Time/min	Area	Height	Area(%)
1	16.201	95192	2138	2.58
2	25.819	3599626	51332	97.42

Condition: hexane: 2-propanol = 95:05

Flow rate = 1.0 mL/min, λ = 254 nm, Chiral AD-H.

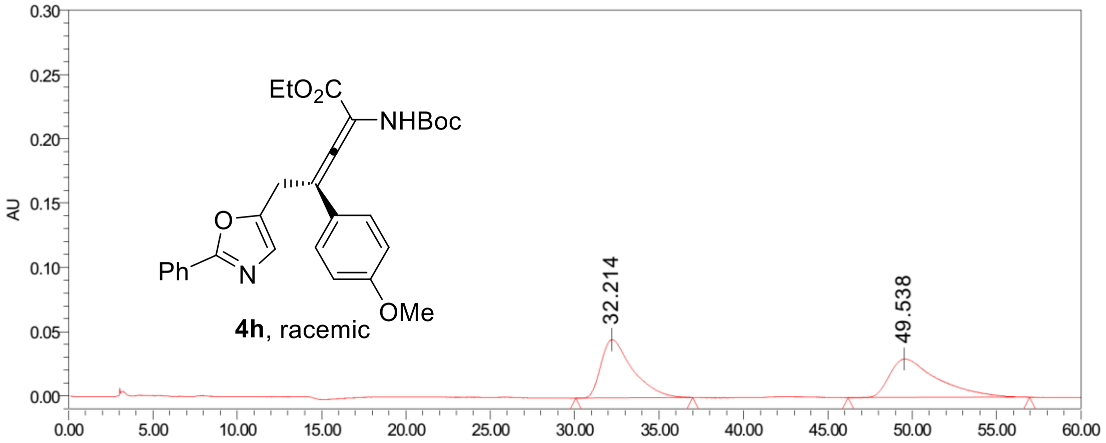

Entry	Retention Time/min	Area	Height	Area(%)
1	32.214	5915490	45374	49.86
2	49.538	5948873	30064	50.14

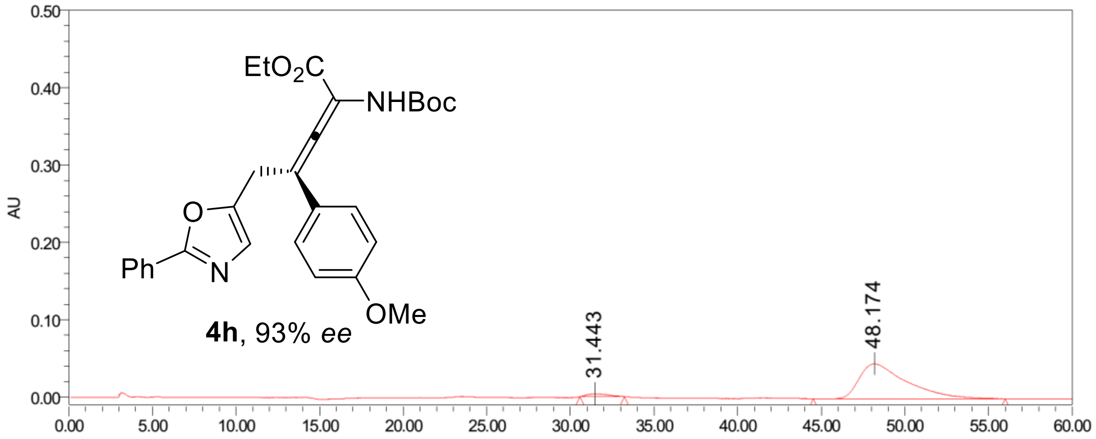

Entry	Retention Time/min	Area	Height	Area(%)
1	31.443	342001	4007	3.73
2	48.174	8815098	45023	96.27

Condition: hexane: 2-propanol =80:20

Flow rate = 1.0 mL/min, λ = 254 nm, Chiral OD-H.

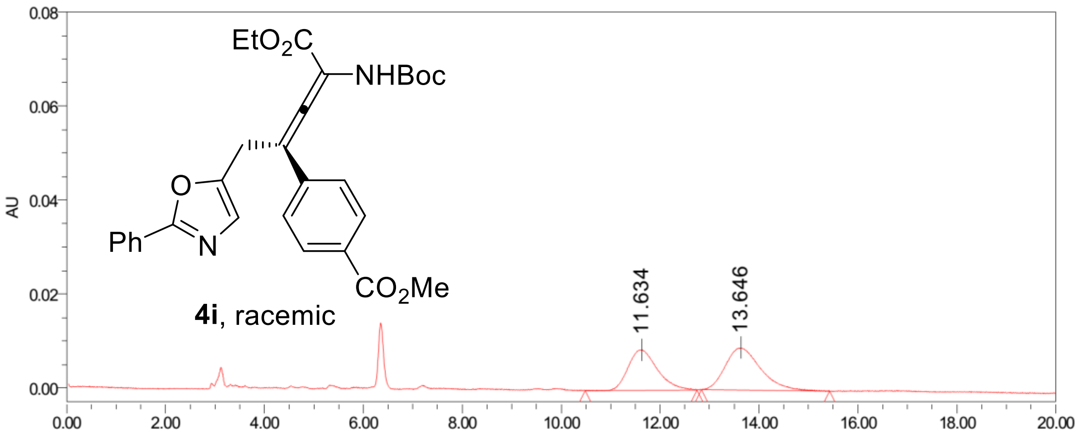

Entry	Retention Time/min	Area	Height	Area(%)
1	11.634	353257	8647	43.96
2	13.646	450249	8977	56.04

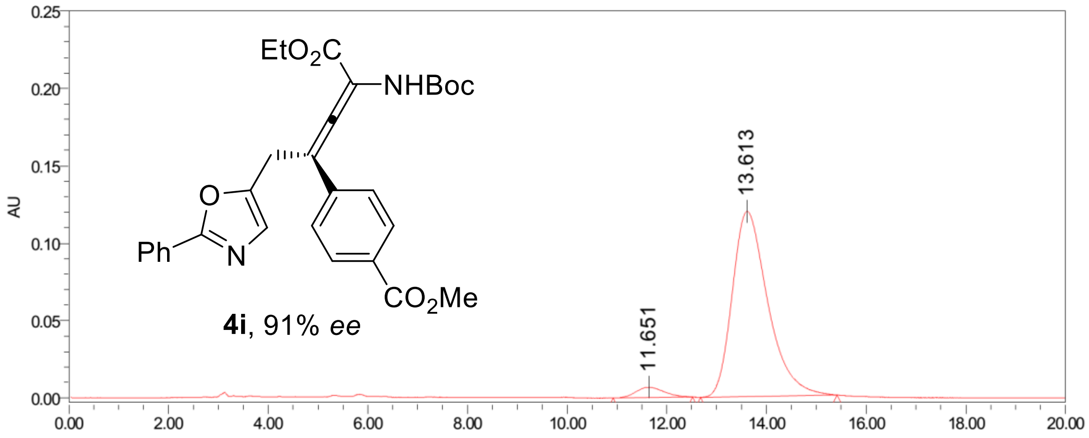

Entry	Retention Time/min	Area	Height	Area(%)
1	11.651	263302	6704	4.24
2	13.613	5952863	119663	95.76

Condition: hexane: 2-propanol = 95:05

Flow rate = 1.0 mL/min, λ = 254 nm, Chiral OD-H.

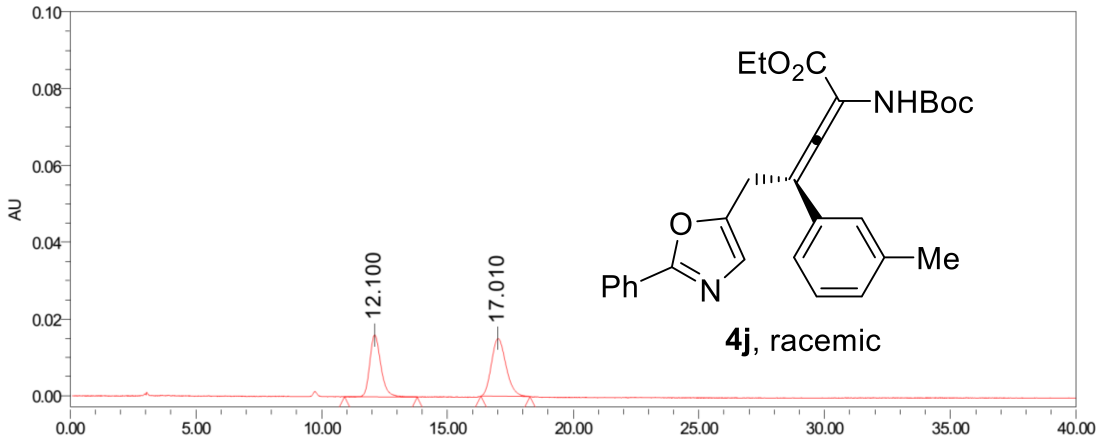

Entry	Retention Time/min	Area	Height	Area(%)
1	12.100	496745	16109	44.84
2	17.010	611098	15123	55.16

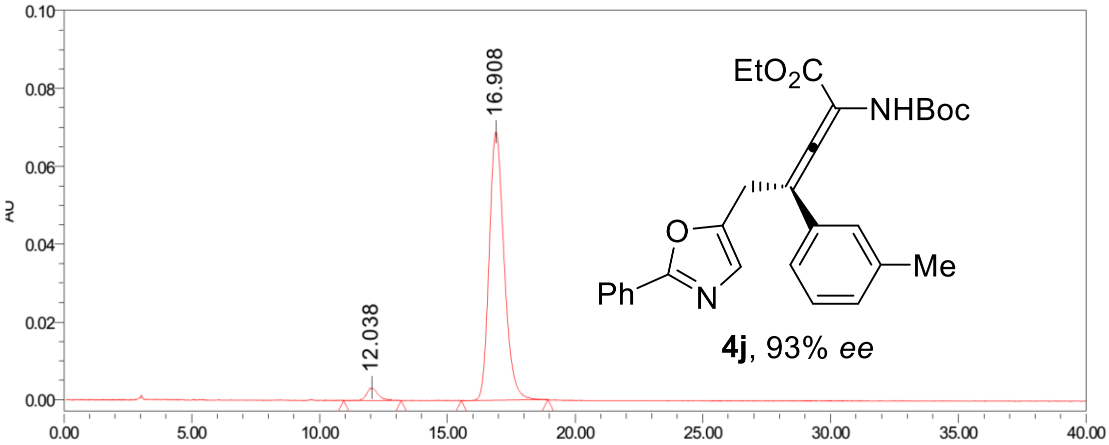

Entry	Retention Time/min	Area	Height	Area(%)
1	12.038	99386	3239	3.42
2	16.908	2804325	68977	96.58

Condition: hexane: 2-propanol = 90:10

Flow rate = 1.0 mL/min, λ = 254 nm, Chiral AD-H.

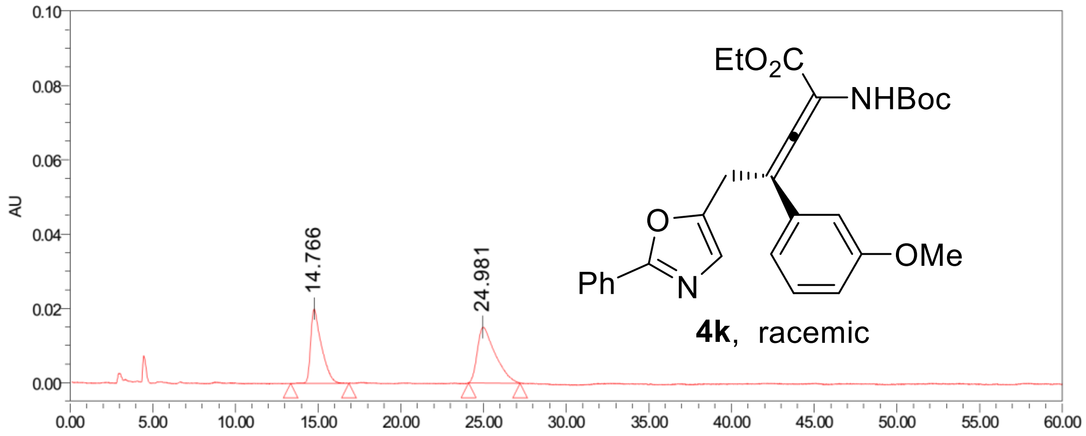

Entry	Retention Time/min	Area	Height	Area(%)
1	14.766	898506	20027	44.35
2	24.981	1127506	15017	55.65

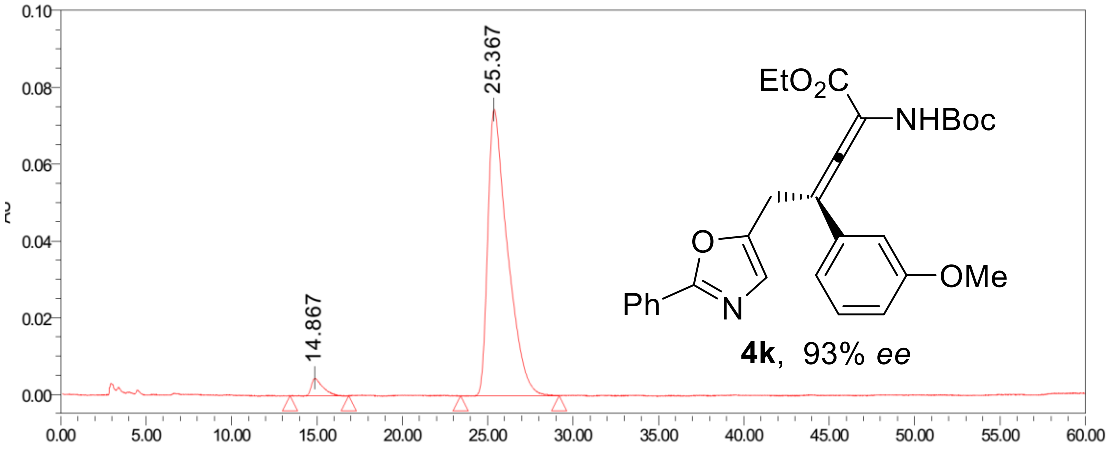

Entry	Retention Time/min	Area	Height	Area(%)
1	14.867	209739	4562	3.38
2	25.367	6000781	74568	96.62

Condition: hexane: 2-propanol = 95:05

Flow rate = 1.0 mL/min, λ = 254 nm, Chiral OD-H.

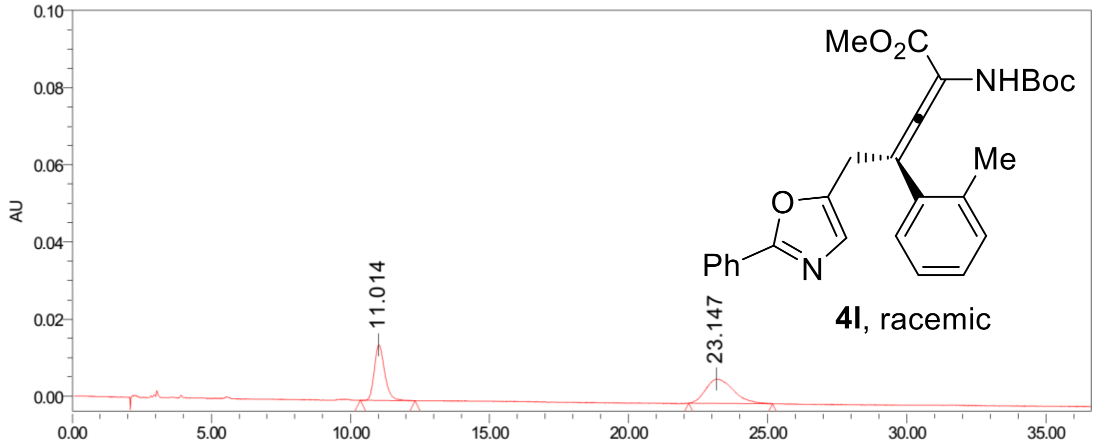

Entry	Retention Time/min	Area	Height	Area(%)
1	11.014	370800	14412	46.04
2	23.147	434590	6316	53.96

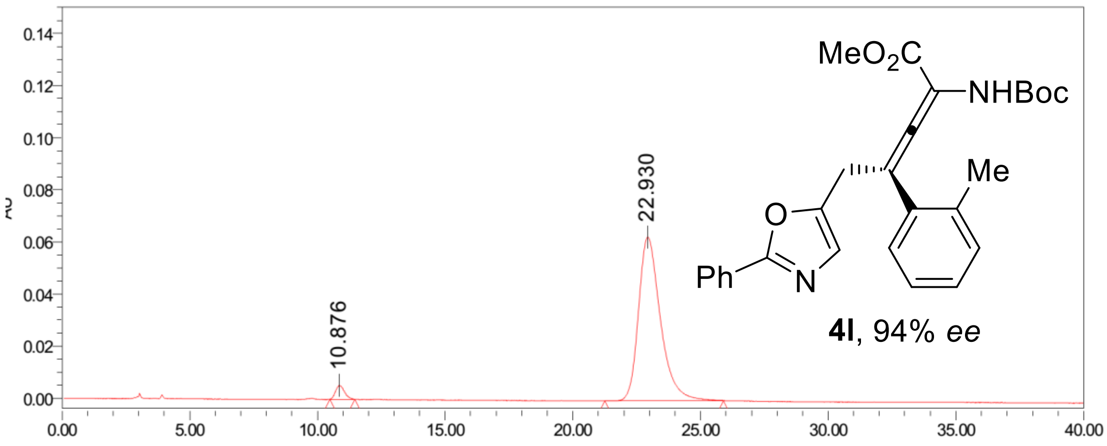

Entry	Retention Time/min	Area	Height	Area(%)
1	10.876	126340	5277	3.26
2	22.930	3753360	62779	96.74

Condition: hexane: 2-propanol = 90:10

Flow rate = 1.0 mL/min, λ = 254 nm, Chiral OD-H.

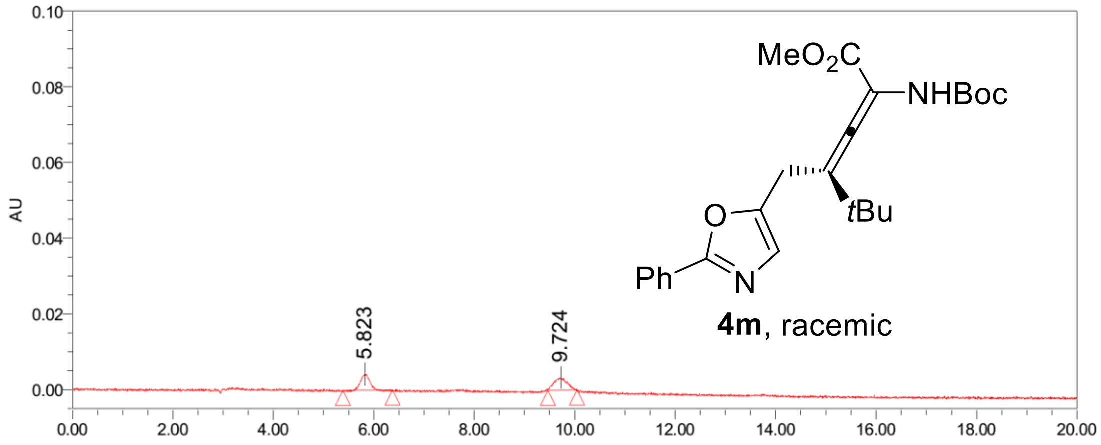

Entry	Retention Time/min	Area	Height	Area(%)
1	5.823	57584	4169	49.17
2	9.724	59521	3354	50.83

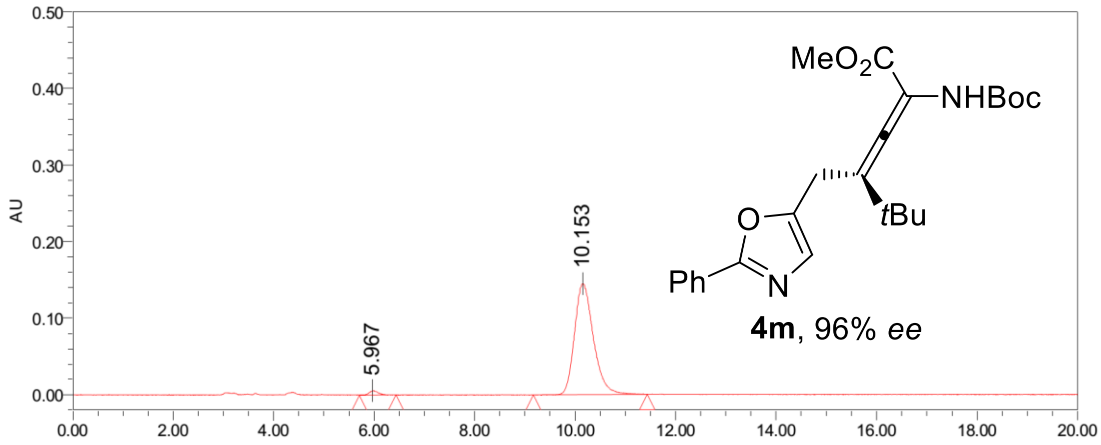

Entry	Retention Time/min	Area	Height	Area(%)
1	5.967	72788	5784	1.90
2	10.153	3755678	144942	98.10

Condition: hexane: 2-propanol = 90:10

Flow rate = 1.0 mL/min, λ = 254 nm, Chiral OD-H.

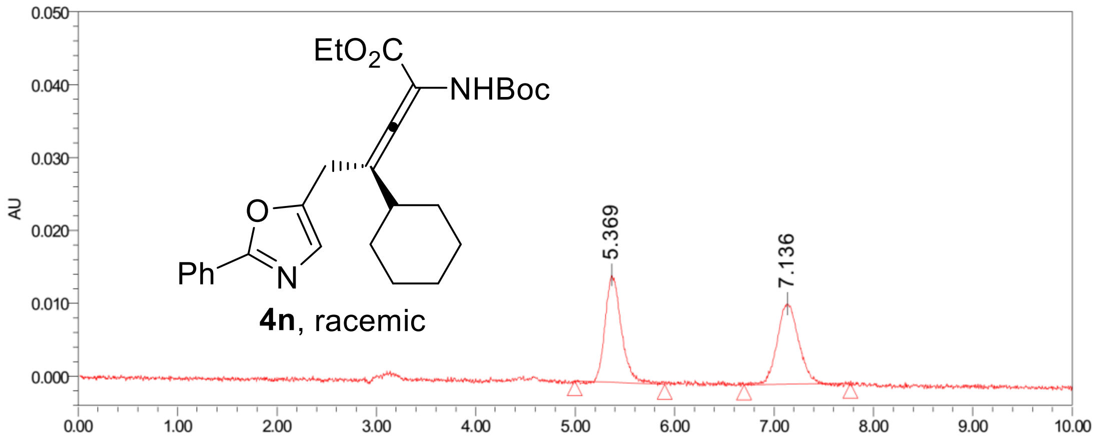

Entry	Retention Time/min	Area	Height	Area(%)
1	5.369	169742	14714	50.32
2	7.136	167555	11055	49.68

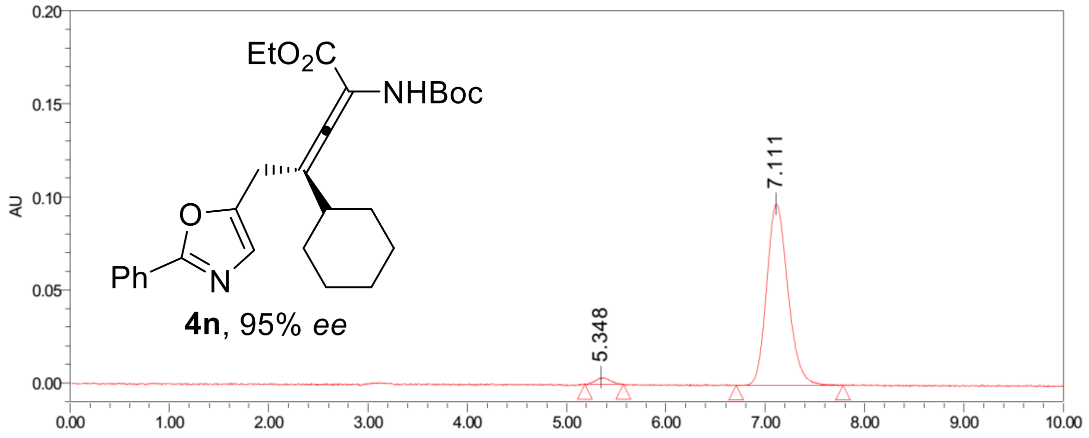

Entry	Retention Time/min	Area	Height	Area(%)
1	5.348	34167	3674	2.31
2	7.111	1444597	97259	97.69

Condition: hexane: 2-propanol = 95:05

Flow rate = 1.0 mL/min, λ = 254 nm, Chiral OD-H

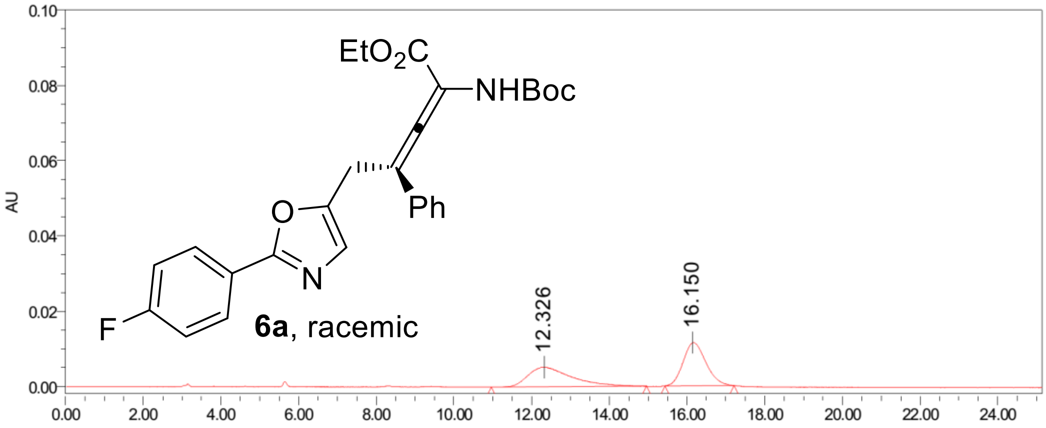

Entry	Retention Time/min	Area	Height	Area(%)
1	12.326	406338	5186	46.52
2	16.150	467114	11457	53.48

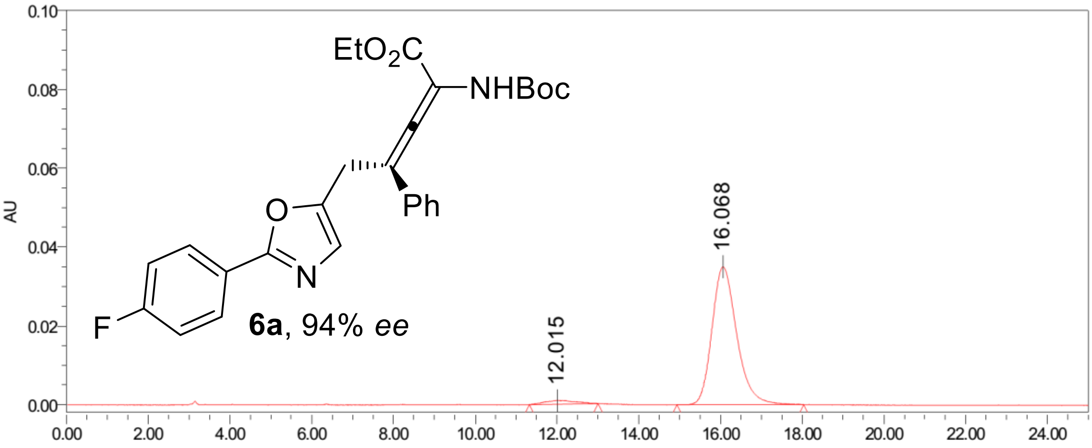

Entry	Retention Time/min	Area	Height	Area(%)
1	12.015	46541	877	3.07
2	16.068	1471453	34972	96.93

Condition: hexane: 2-propanol = 95:05

Flow rate = 1.0 mL/min, λ = 254 nm, Chiral OD-H

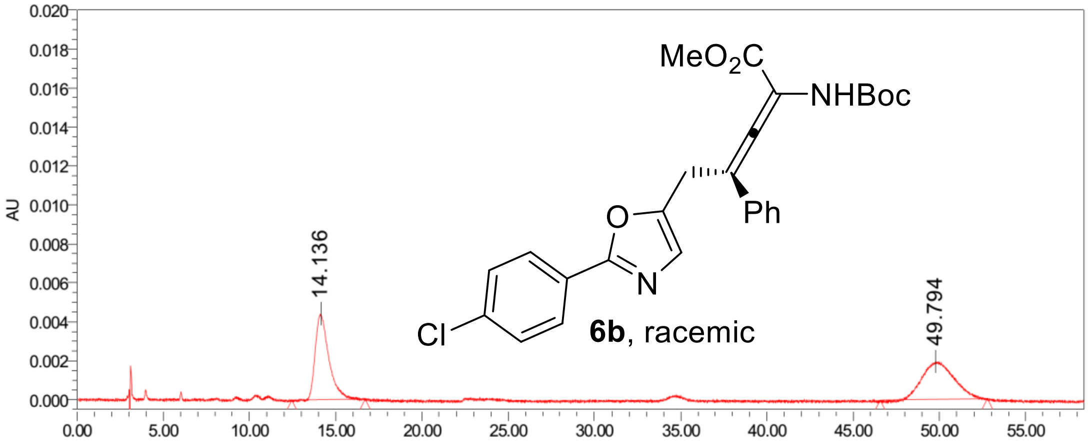

Entry	Retention Time/min	Area	Height	Area(%)
1	14.136	243925	4414	49.42
2	49.794	249694	1958	50.58

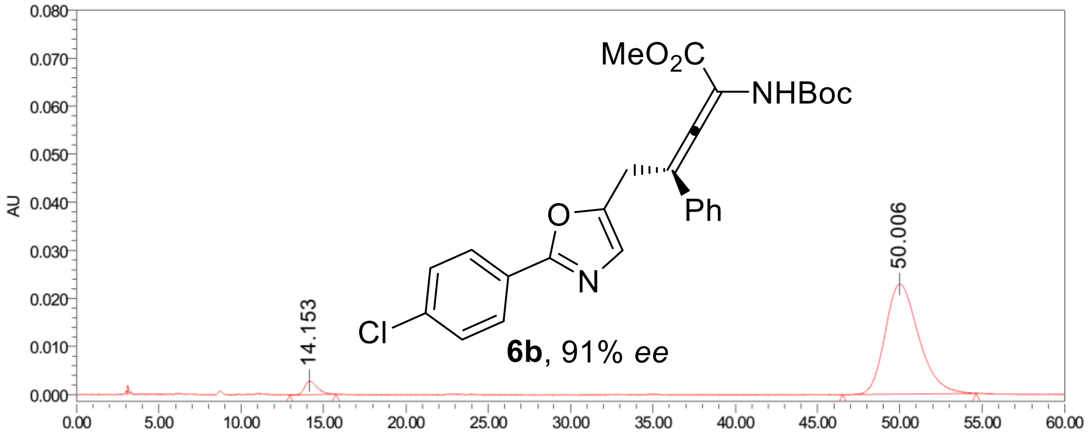

Entry	Retention Time/min	Area	Height	Area(%)
1	14.153	148544	2820	4.39
2	50.006	3232789	22921	95.61

Condition: hexane: 2-propanol = 95:05

Flow rate = 1.0 mL/min, λ = 254 nm, Chiral OD-H

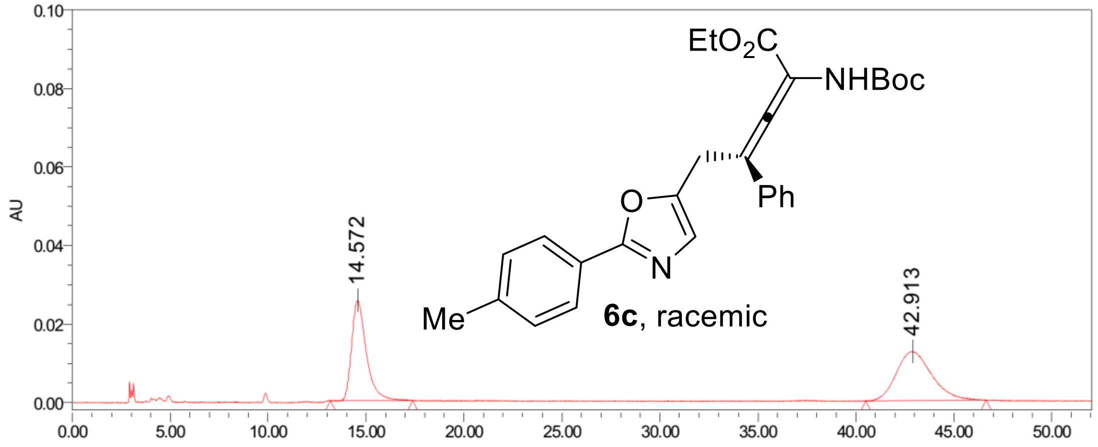

Entry	Retention Time/min	Area	Height	Area(%)
1	14.572	1317283	25462	45.57
2	42.913	1573096	12543	54.43

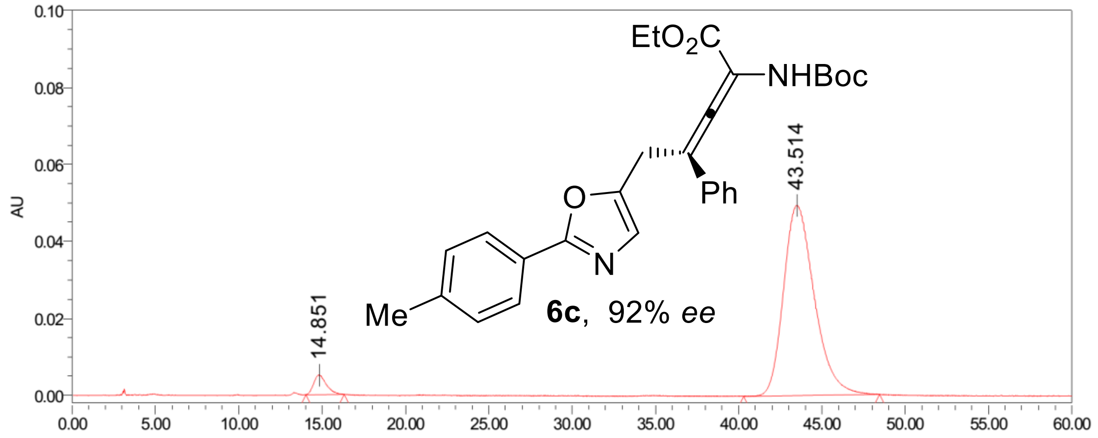

Entry	Retention Time/min	Area	Height	Area(%)
1	14.851	265600	5093	4.03
2	43.514	6321105	49428	95.97

Condition: hexane: 2-propanol = 90:10

Flow rate = 1.0 mL/min, λ = 254 nm, Chiral AD-H

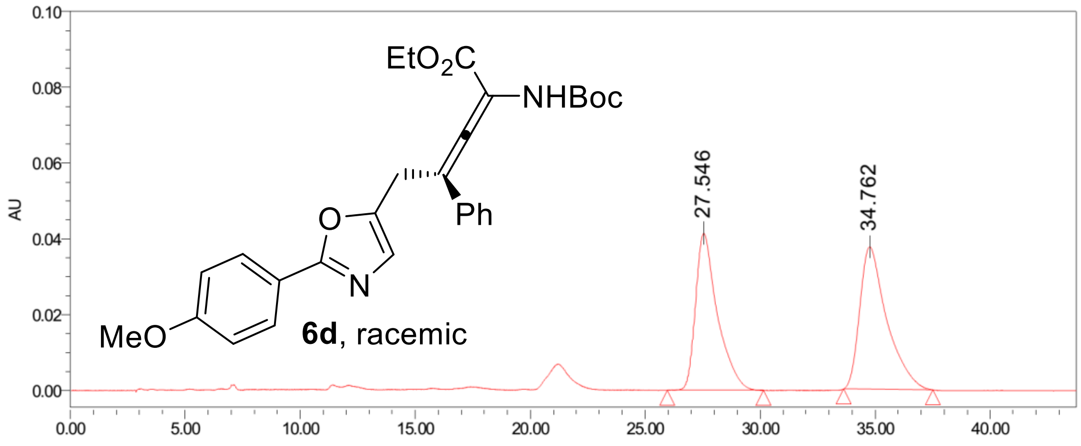

Entry	Retention Time/min	Area	Height	Area(%)
1	27.546	2727163	41390	46.94
2	34.762	3082382	37560	53.06

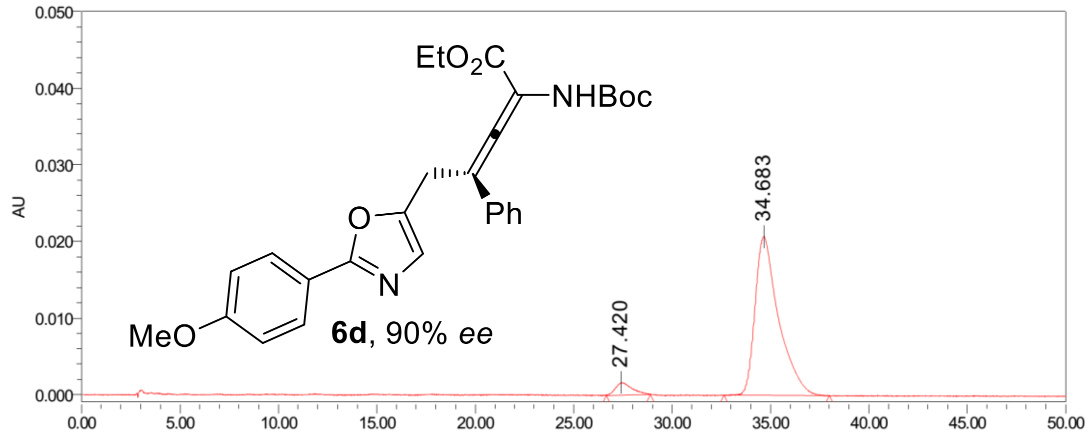

Entry	Retention Time/min	Area	Height	Area(%)
1	27.420	93049	1609	5.10
2	34.683	1730019	20732	94.90

Condition: hexane: 2-propanol = 90:10

Flow rate = 1.0 mL/min, λ = 254 nm, Chiral AD-H

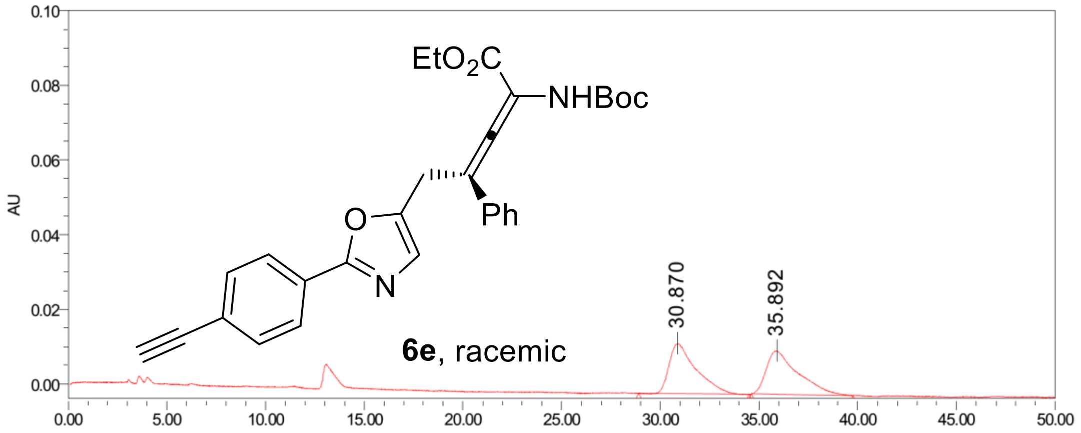

Entry	Retention Time/min	Area	Height	Area(%)
1	30.870	1279169	13540	49.72
2	35.892	1293532	11600	50.28

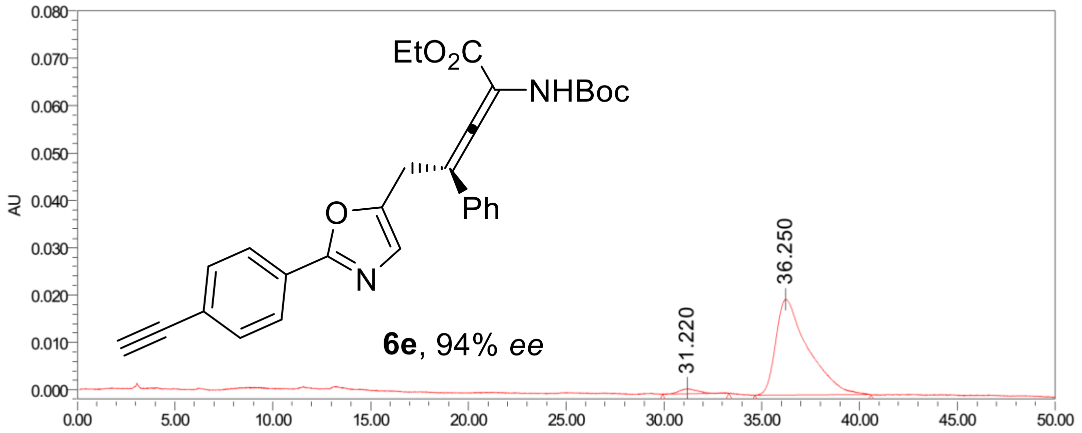

Entry	Retention Time/min	Area	Height	Area(%)
1	31.220	66796	1099	2.82
2	36.250	2301185	20208	97.18

Condition: hexane: 2-propanol = 90:10

Flow rate = 1.0 mL/min, λ = 254 nm, Chiral IC

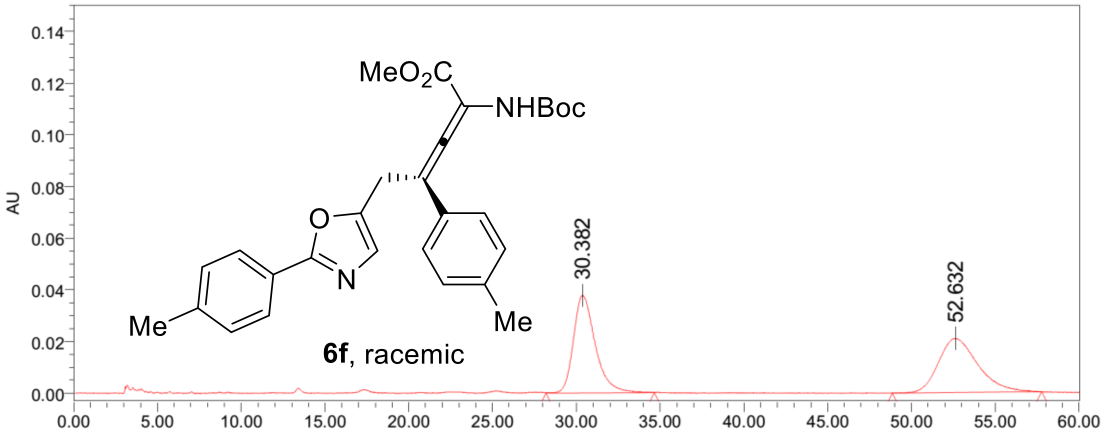

Entry	Retention Time/min	Area	Height	Area(%)
1	30.382	3429533	37718	50.00
2	52.632	3430162	20978	50.00

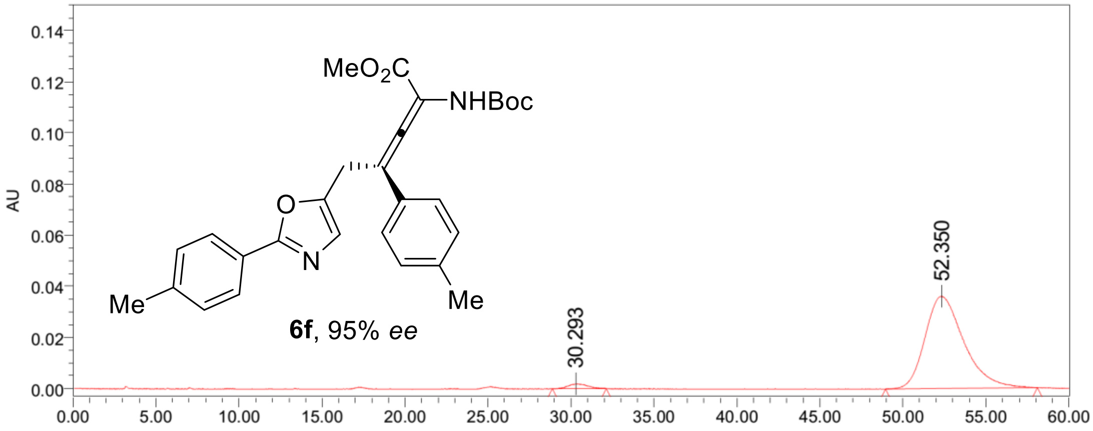

Entry	Retention Time/min	Area	Height	Area(%)
1	30.293	148326	1941	2.47
2	52.350	5860980	36003	97.53

Condition: hexane: 2-propanol = 95:05

Flow rate = 1.0 mL/min, λ = 254 nm, Chiral OD-H.

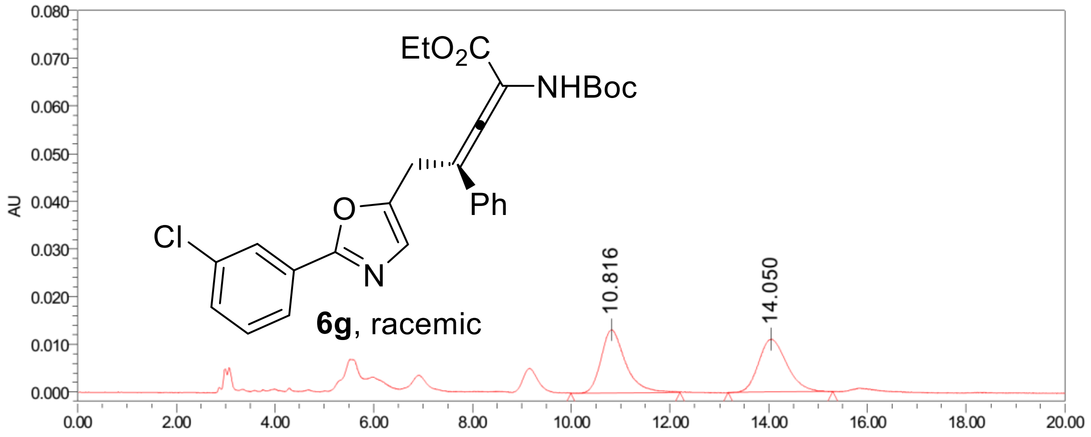

Entry	Retention Time/min	Area	Height	Area(%)
1	10.816	450831	13204	50.58
2	14.050	440468	11128	49.42

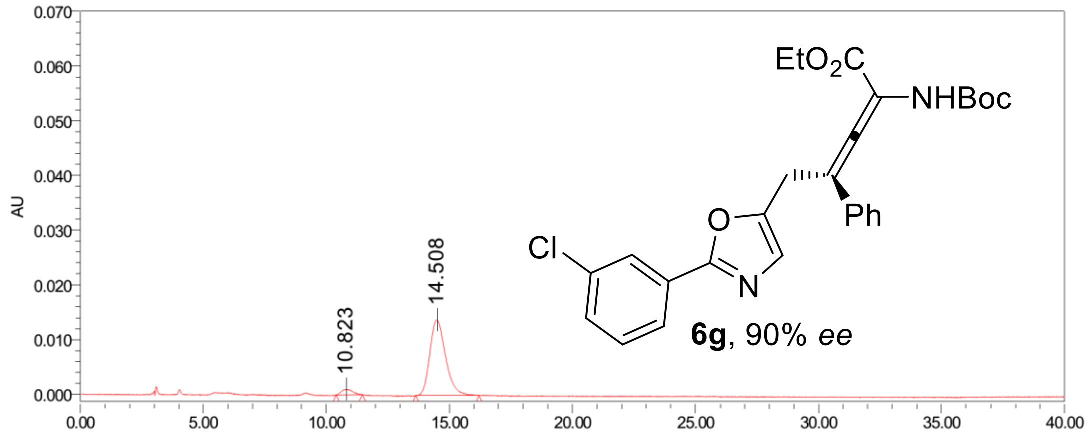

Entry	Retention Time/min	Area	Height	Area(%)
1	10.823	34376	1072	5.29
2	14.508	615994	13877	94.71

Condition: hexane: 2-propanol = 90:10

Flow rate = 1.0 mL/min, λ = 254 nm, Chiral OD-H.

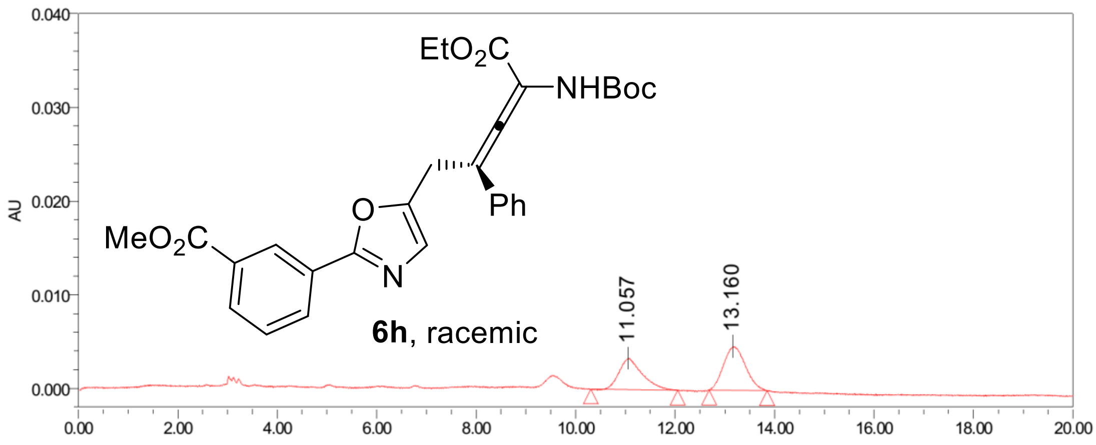

Entry	Retention Time/min	Area	Height	Area(%)
1	11.057	108457	3355	43.23
2	13.160	142411	4636	56.77

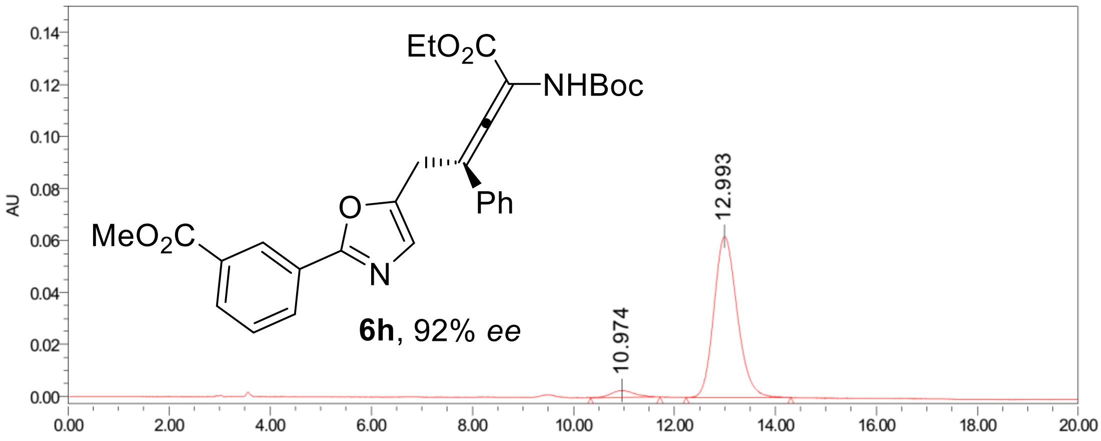

Entry	Retention Time/min	Area	Height	Area(%)
1	10.974	83292	2598	4.04
2	12.993	1980738	61920	95.96

Condition: hexane: 2-propanol = 95:05

Flow rate = 1.0 mL/min, λ = 254 nm, Chiral OD-H.

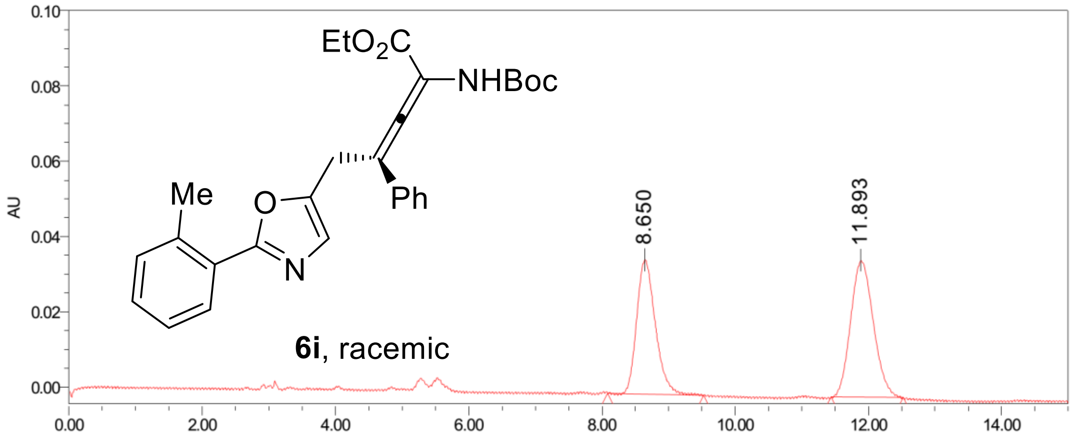

Entry	Retention Time/min	Area	Height	Area(%)
1	8.650	698586	35643	44.74
2	11.893	862768	36289	55.26

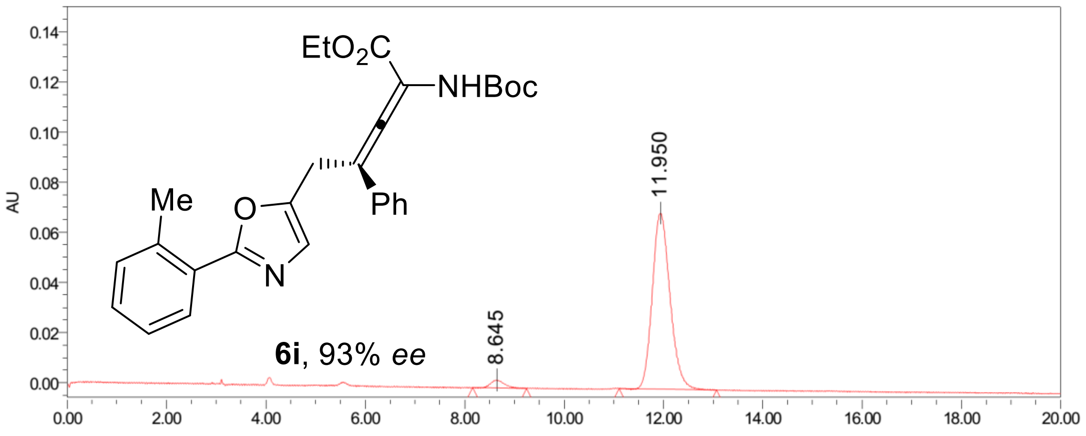

Entry	Retention Time/min	Area	Height	Area(%)
1	8.645	66295	3295	3.66
2	11.95	1743995	70233	96.34

Condition: hexane: 2-propanol = 95:05

Flow rate = 1.0 mL/min, λ = 254 nm, Chiral OD-H.

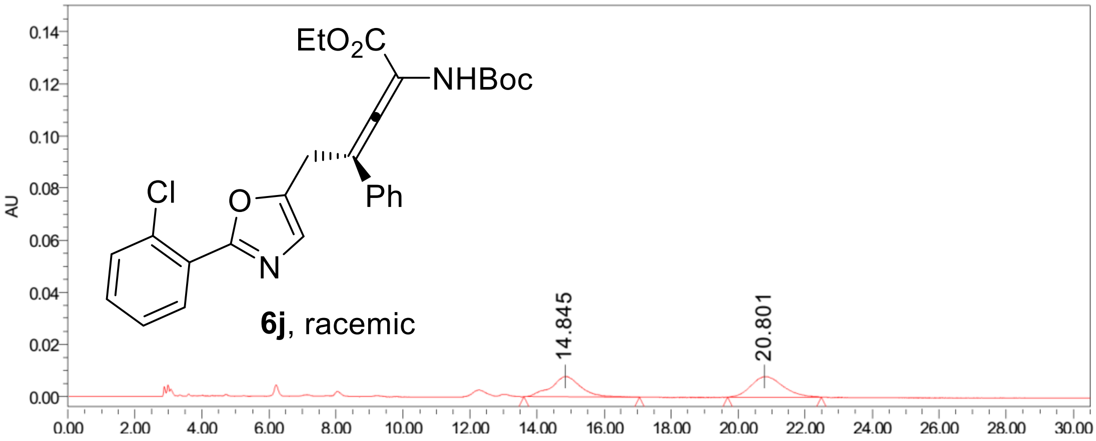

Entry	Retention Time/min	Area	Height	Area(%)
1	14.845	482250	7838	48.26
2	20.801	516945	7857	51.74

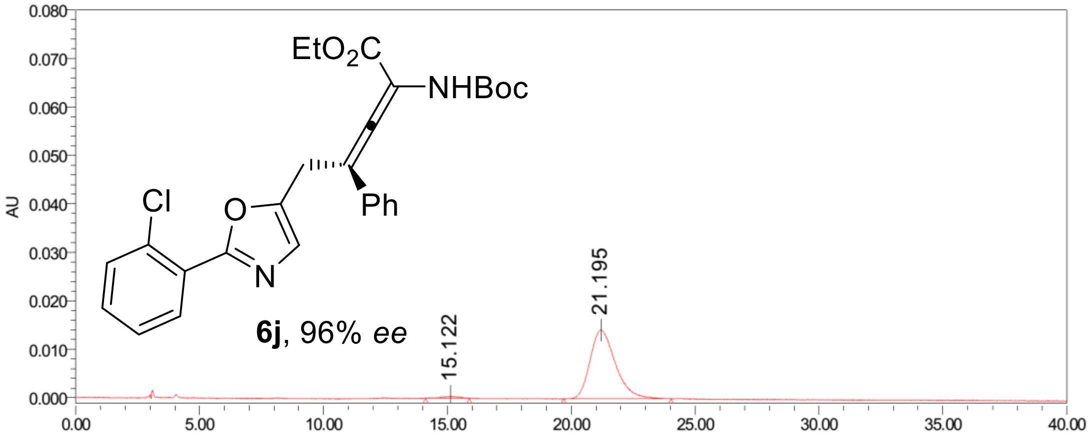

Entry	Retention Time/min	Area	Height	Area(%)
1	15.122	18555	394	1.81
2	21.195	1005172	14148	98.19

Condition: hexane: 2-propanol = 95:05

Flow rate = 1.0 mL/min, λ = 254 nm, Chiral OD-H.

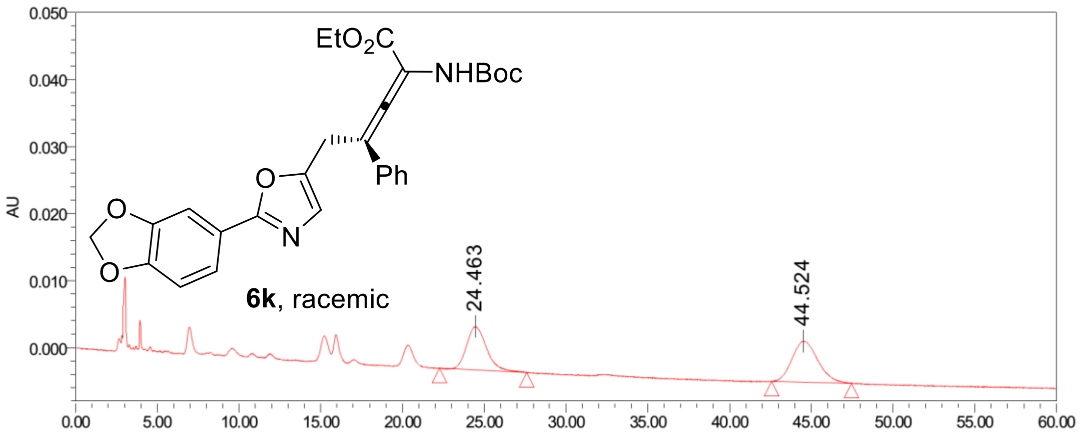

Entry	Retention Time/min	Area	Height	Area(%)
1	24.463	552121	6489	44.92
2	44.524	676878	6137	55.08

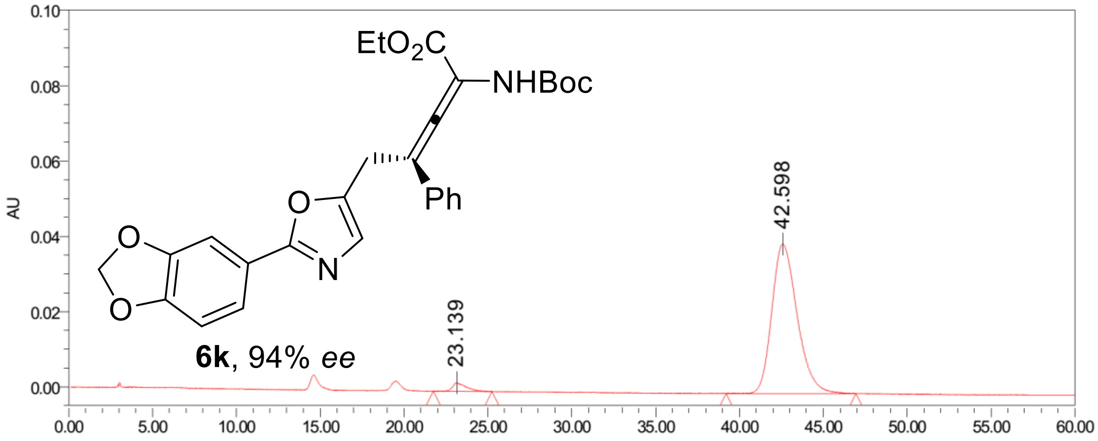

Entry	Retention Time/min	Area	Height	Area(%)
1	23.139	129720	2288	3.03
2	42.598	4154088	39665	96.97

Condition: hexane: 2-propanol = 90:10

Flow rate = 1.0 mL/min, λ = 254 nm, Chiral AD-H

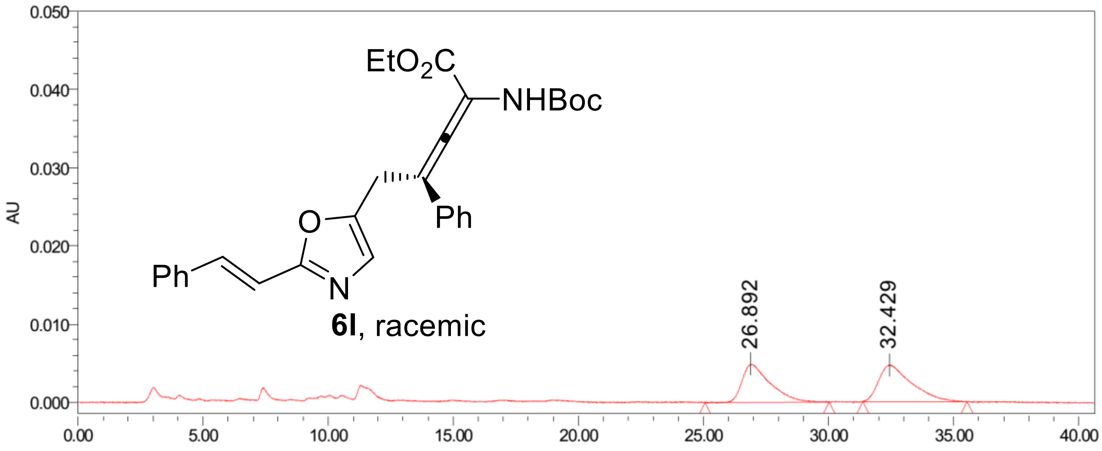

Entry	Retention Time/min	Area	Height	Area(%)
1	26.892	378795	4865	46.87
2	32.429	429437	4705	53.13

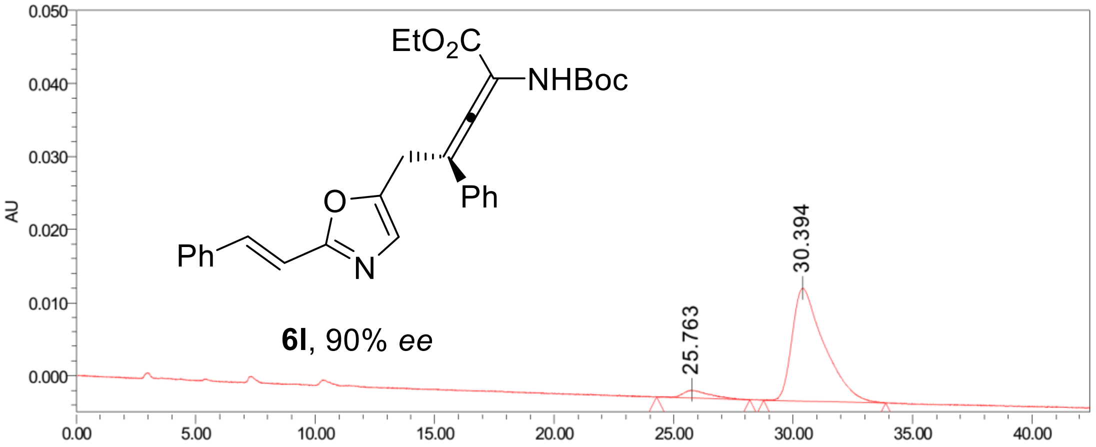

Entry	Retention Time/min	Area	Height	Area(%)
1	25.763	79557	1081	5.25
2	30.394	1436287	15464	94.75

Condition: hexane: 2-propanol = 95:05

Flow rate = 1.0 mL/min, λ = 254 nm, Chiral OD-H

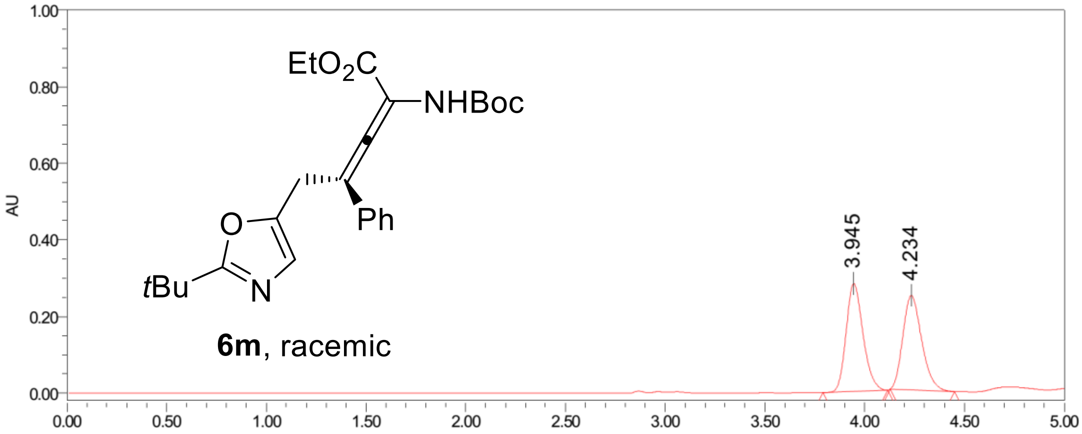

Entry	Retention Time/min	Area	Height	Area(%)
1	3.945	1656413	281896	50.53
2	4.234	1621751	246655	49.47

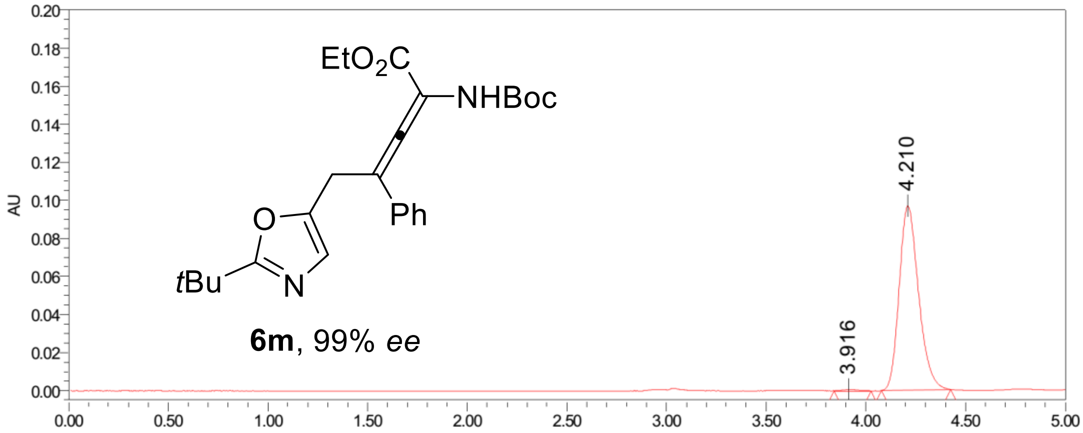

Entry	Retention Time/min	Area	Height	Area(%)
1	3.916	3111	594	0.47
2	4.210	654638	96853	99.53

Condition: hexane: 2-propanol = 70:30

Flow rate = 1.0 mL/min, λ = 254 nm, Chiral IA

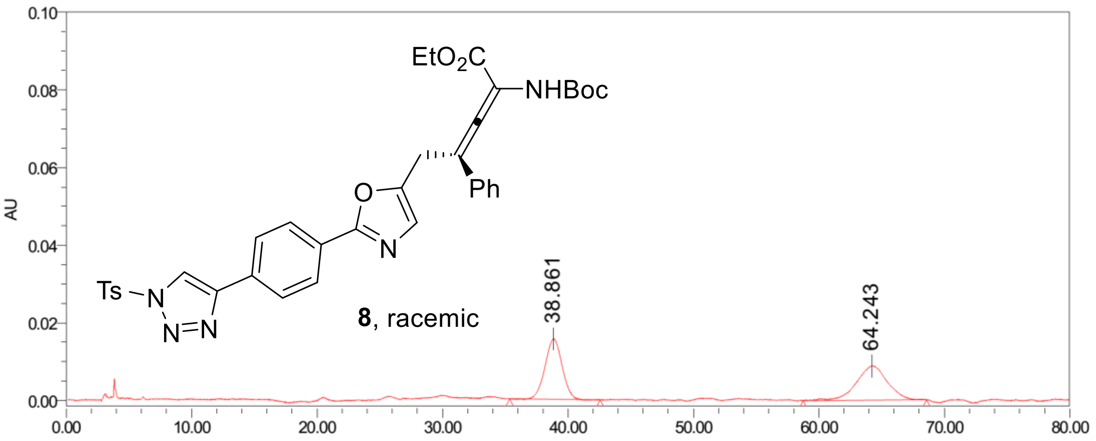

Entry	Retention Time/min	Area	Height	Area(%)
1	38.861	1488180	15571	49.86
2	64.243	1496544	8814	50.14

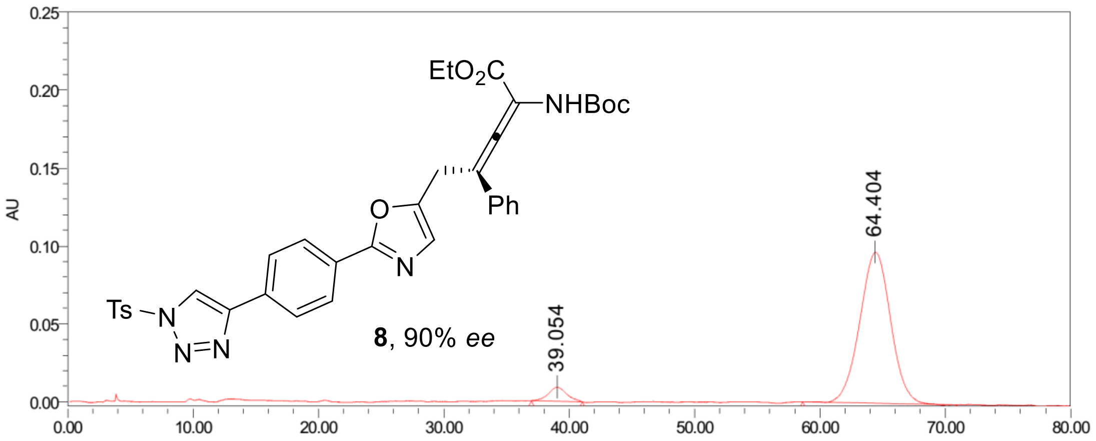

Entry	Retention Time/min	Area	Height	Area(%)
1	39.054	912050	9032	5.18
2	64.404	16681087	96854	94.82

Condition: hexane: 2-propanol = 95:05

Flow rate = 1.0 mL/min, λ = 254 nm, Chiral IC

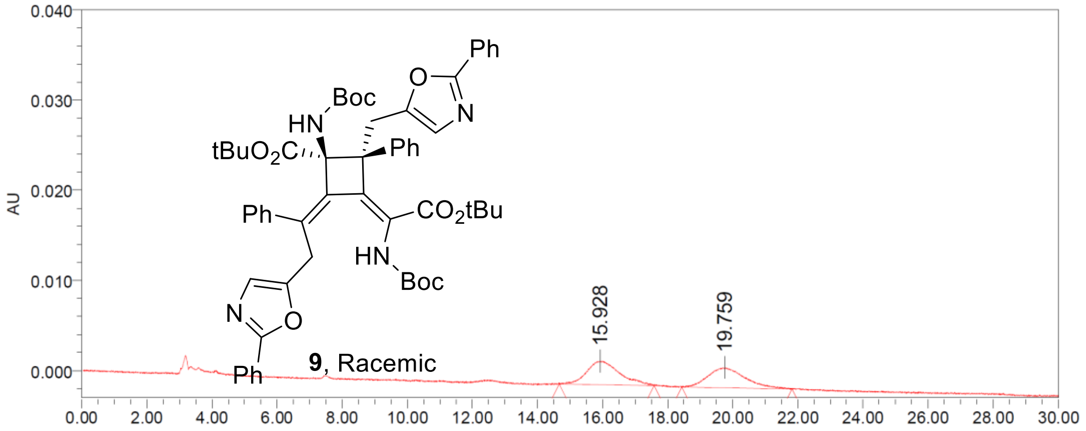

Entry	Retention Time/min	Area	Height	Area(%)
1	15.928	180853	2628	50.81
2	19.759	175059	2253	49.19

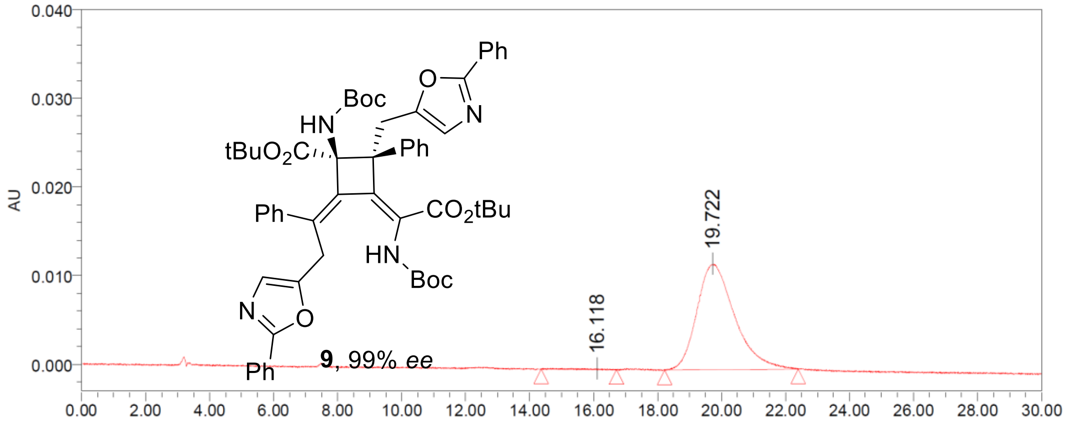

Entry	Retention Time/min	Area	Height	Area(%)
1	16.118	5388	122	0.55
2	19.722	974794	11929	99.45

Condition: hexane: 2-propanol = 90:10

Flow rate = 1.0 mL/min, λ = 254 nm, Chiral OD-H

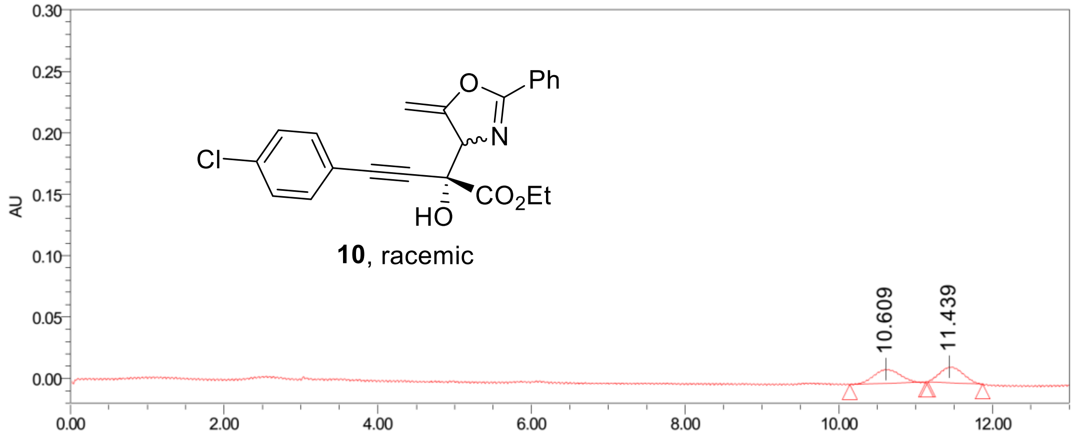

Entry	Retention Time/min	Area	Height	Area(%)
1	10.609	269755	11392	49.94
2	11.439	270367	12937	50.06

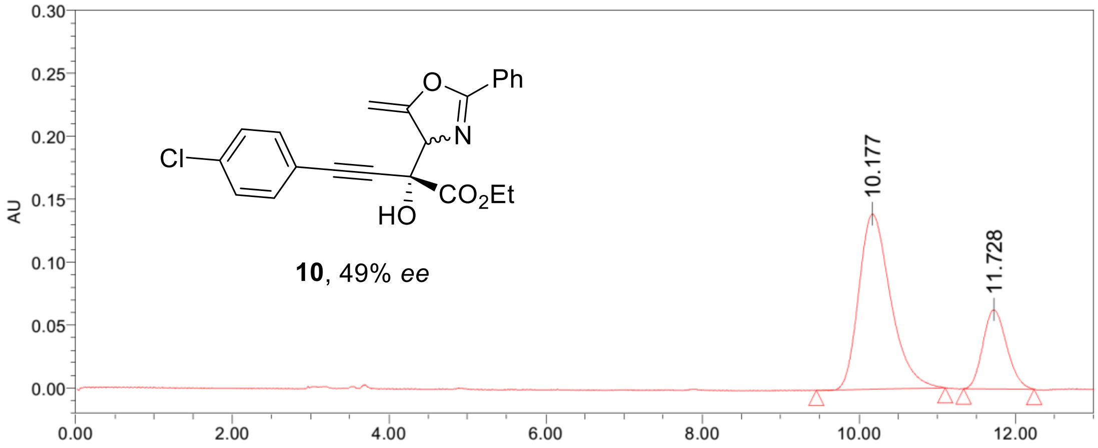

Entry	Retention Time/min	Area	Height	Area(%)
1	10.177	3925301	139367	74.64
2	11.728	1333782	63002	25.36

Condition: hexane: 2-propanol = 90:10

Flow rate = 1.0 mL/min, λ = 254 nm, Chiral IC

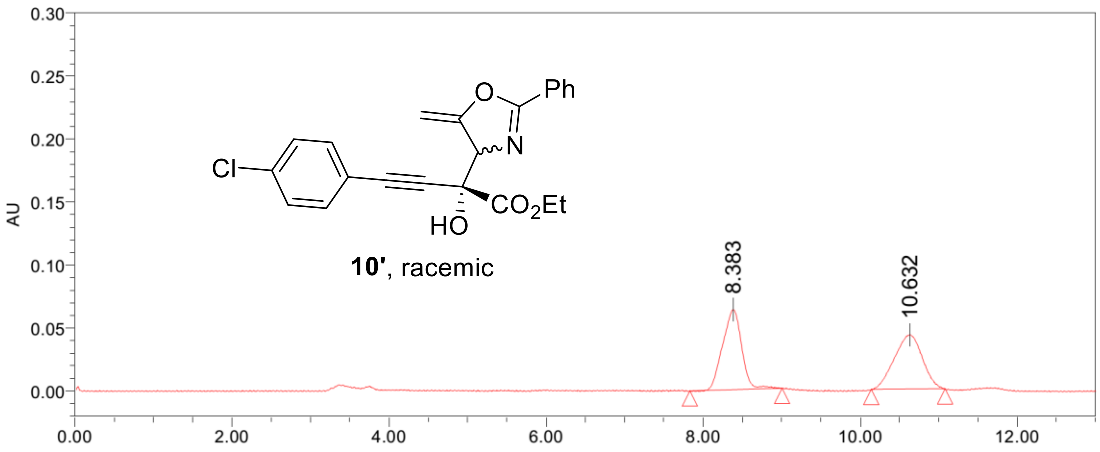

Entry	Retention Time/min	Area	Height	Area(%)
1	8.383	1062562	63461	49.81
2	10.632	1070781	43401	50.19

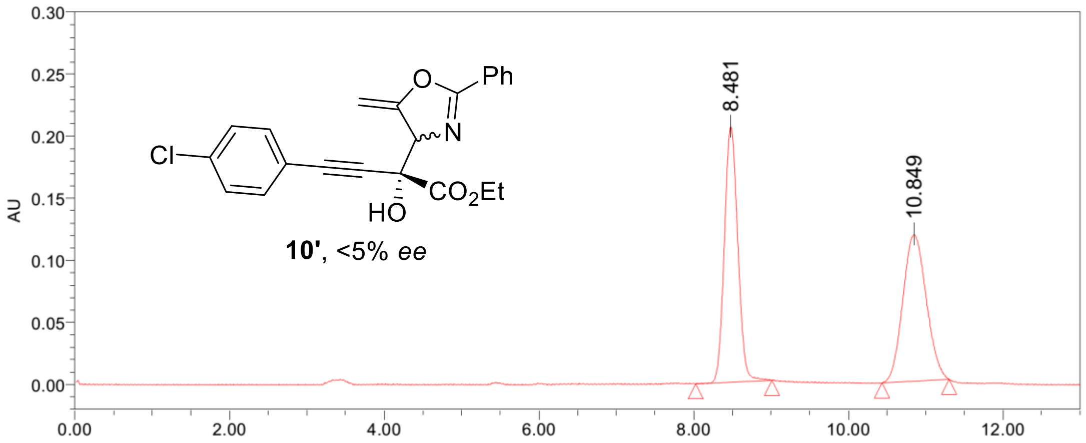

Entry	Retention Time/min	Area	Height	Area(%)
1	8.481	2478360	205966	49.89
2	10.849	2489738	118425	50.11

Crystallographic Data for Compound 6f (2235092)

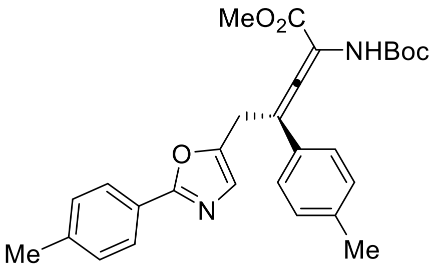

6f

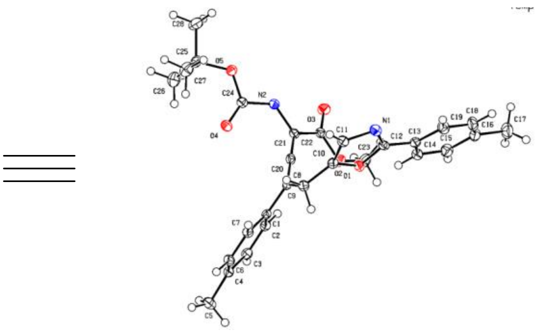

Datablock: 20230109-yhx-1_auto

```
Bond precision:   C-C = 0.0030 A          Wavelength=1.54184

Cell:           a=14.19128(15)    b=6.07041(5)       c=15.85957(16)
                alpha=90          beta=113.2156(13)  gamma=90
Temperature:    100 K
```

	Calculated	Reported
Volume	1255.62(2)	1255.63(2)
Space group	P 21	P 1 21 1
Hall group	P 2yb	P 2yb
Moiety formula	C28 H29 N2 O5	C28 H29 N2 O5
Sum formula	C28 H29 N2 O5	C28 H29 N2 O5
Mr	473.53	473.53
Dx,g cm-3	1.253	1.252
Z	2	2
Mu (mm-1)	0.701	0.701
F000	502.0	502.0
F000’	503.56	
h,k,lmax	16,7,18	16,7,18
Nref	4485[2471]	4442
Tmin,Tmax	0.777,0.869	0.157,1.000
Tmin’	0.755	

```
Correction method= # Reported T Limits: Tmin=0.157 Tmax=1.000
AbsCorr = MULTI-SCAN

Data completeness= 1.80/0.99       Theta(max)= 67.036

R(reflections)= 0.0295( 4424)                      wR2(reflections)=
                                                   0.0782( 4442)
S = 1.088                 Npar= 322
```

Crystallographic Data for Compound 9 (2235970)

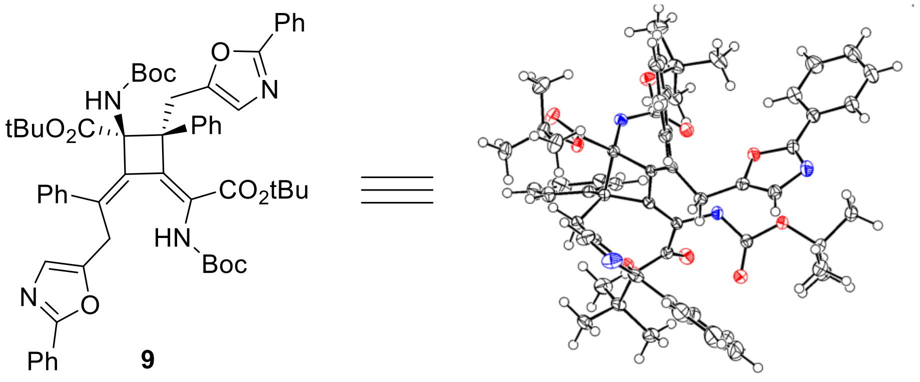

Datablock: 20230112-2_auto

Bond precision: C-C = 0.0031 A Wavelength=1.54184

Cell: a=13.8272(2) b=17.8931(2) c=21.0743(3)
alpha=90 beta=90 gamma=90

Temperature: 100 K

	Calculated	Reported
Volume	5214.02(12)	5214.02(12)
Space group	P 21 21 21	P 21 21 21
Hall group	P 2ac 2ab	P 2ac 2ab
Moiety formula	C58 H62 N4 O10	C58 H62 N4 O10
Sum formula	C58 H62 N4 O10	C58 H62 N4 O10
Mr	975.12	975.11
Dx,g cm-3	1.242	1.242
Z	4	4
Mu (mm-1)	0.689	0.689
F000	2072.0	2072.0
F000'	2078.36	
h,k,lmax	16,21,25	16,21,25
Nref	9295[5145]	9291
Tmin,Tmax	0.848,0.933	0.793,1.000
Tmin'	0.813	

Correction method= # Reported T Limits: Tmin=0.793 Tmax=1.000
AbsCorr = MULTI-SCAN

Data completeness= 1.81/1.00 Theta(max)= 67.052

R(reflections)= 0.0279(8930) wR2(reflections)= 0.0720(9291)

S = 1.043 Npar= 661

Computational Studies

All DFT calculations were performed with Gaussian 09[3] software package. Geometry optimizations of all the stationary points were carried out using the PBE0[4] functional and def2-SVP[5] with the corresponding pseudo-potential[6]. The PBE0 was chosen due to its excellent performance on 5d transition metal complexes[7]. Frequency calculations at the same level were performed to validate each structure as either a minimum or a transition state. Based on the optimized structures, single-point energy refinements were performed at the BMK-D3(BJ)/def2-TZVPP level[8] under SMD[9] model to account for solvation effects of DCE. The BMK functional was chosen because of its high accuracy in computing the kinetics of gold-catalyzed reactions.[10] Pruned integration grids with 99 radial shells and 590 angular points per shell were used. All discussed energy differences were based on Gibbs energies at 298 K (standard states are the hypothetical states at 1 mol/L) unless otherwise specified. 3D structure was prepared with CYLview.[11] NPA charge was computed using the NBO 3.1 built in Gaussian 09.[12] ESP was obtained with Multiwfn 3.8(dev)[13] and visualized with VMD 1.9.3.[14]

Conformational research was performed for the transition states related to the stereoselectivity of this reaction through a set of workflows. First, Conformer-Rotamer Ensemble Sampling Tool (CREST)[15] based on GFN0-xTB level was used to generate hundreds of low-energy structures of transition states related to different enantiomers and diastereomers. The Au-C bond length and the forming C-C bond were selected as constraints with force constant of 0.5. Then the low energy candidate structures were included in the subsequent constrained geometry optimization step at GFN2-xTB level with GBSA(DCM) model (DCE has not been supported by GBSA). Isostat tool in Molclus package[16] was used to sort and distinguish different structures with energy threshold of 0.5 kcal/mol and geometry threshold of 0.5 Å. At last, the conformers within an energy window of 6 kcal/mol (4~15 candidate structures) were optimized without constrains using Gaussian 09 software package at IEFPCM(DCE)/PBE0/def2-SVP level, then single-point energy

refinements were performed at SMD(DCE)/BMK-D3(BJ)/def2-TZVPP to find the most stable conformers of the corresponding transition states. Quasiharmonic corrections were applied with Grimme's quasi-RRHO correction[17] to obtain the thermal correction to Gibbs free energy at 298.15 K and 1 M using Shermo software package (Table S2).[18]

Table S2. Computed Energies for the Stationary Points.

Thermal corrections to Gibbs energies (TCGs), single-point energies (SPEs) in gas phase and solvent.

	Imaginary Frequencies (cm^{-1})	SPEs (in gas phase)[a] (hartree)	TCGs (in gas phase)[b] (hartree)	SPEs (under SMD model)[c] (hartree)
**A**	None	-1111.657258	0.230019	-1112.382995
**C**	None	-1111.302541	0.221498	-1111.959881
**QN-SQA**	None	-930.469777	0.21627	-932.052451
**1a**	None	-515.429275	0.128606	-516.300868
**2a**	None	-973.718483	0.248846	-975.37915
**3a**	None	-1602.894125	0.489527	-1605.59823
**3a-Proton**	None	-1603.286093	0.504081	-1606.059132
**8a**	None	-1489.279423	0.41008	-1491.79197
**8a'**	None	-1489.235693	0.408969	-1491.756717
**Int1**	None	-3015.512285	0.739154	-3019.431654
**Int2**	None	-2085.468723	0.508416	-2087.832602
**Int3**	None	-2085.464767	0.510073	-2087.84391
**Int4**	None	-2085.495043	0.514195	-2087.859536
**Int5**	None	-2085.507834	0.512057	-2087.879426
**TS1**	-358.66	-3015.512851	0.738187	-3019.40871
**TS2**	-290.01	-3015.509638	0.737349	-3019.419579
**TS3**	-516.94	-3015.494959	0.736016	-3019.404184
**TS4**	-471.82	-2085.426367	0.510258	-2087.790491
**TS5**	-193.69	-2085.457948	0.512223	-2087.827657
**TS6**	-289.29	-2085.488215	0.514176	-2087.853632

[a] Computed at PBE0/def2-SVP

[b] Computed at PBE0/def2-SVP

[c] Computed at SMD(DCE)/BMK-D3(BJ)/def2-TZVPP

Thermal corrections to Gibbs energies (TCGs), single-point energies (SPEs) in gas phase and solvent.

	Imaginary Frequencies (cm^{-1})	SPEs[a] (hartree)	TCGs[b] (hartree)	SPE[c] (hartree)
**TS1-*RR***	-308.88	-4930.499823	1.679331	-4937.604062
**TS1-*SS***	-312.39	-4930.497184	1.679439	-4937.602154
**TS1-*RS***	-324.59	-4930.497321	1.679782	-4937.603938
**TS1-*SR***	-238.55	-4930.497574	1.679431	-4937.593875

[a] Computed at IEFPCM(DCE)/PBE0/def2-SVP

[b] Computed at IEFPCM(DCE)/PBE0/def2-SVP after Grimme's quasi-RRHO correction

[c] Computed at SMD(DCE)/BMK-D3(BJ)/def2-TZVPP

Cartesian coordinates for the stationary points

1a

C	0.405513	0.828540	0.009247
C	2.779687	0.517173	-0.497886
C	3.714565	-0.453949	0.065461
O	0.592045	1.961515	0.414385
H	3.102910	0.778655	-1.522482
N	1.413515	0.054727	-0.484136
C	4.491419	-1.269760	0.508611
C	-0.960258	0.207388	0.004250
C	-1.183586	-1.174199	-0.025589
C	-2.055053	1.075994	0.067747
C	-2.482938	-1.677224	-0.014345
H	-0.344022	-1.874848	-0.023342
C	-3.352542	0.573917	0.070557
H	-1.856391	2.148610	0.118024
C	-3.568984	-0.803919	0.026647
H	-2.647487	-2.757281	-0.028340
H	-4.201862	1.260197	0.111551
H	-4.587800	-1.199210	0.033075
H	1.203123	-0.842833	-0.897337
H	2.791182	1.454359	0.081911
H	5.182445	-1.986636	0.912615

2a

N	-1.819845	1.346249	0.438037

C	-0.633912	1.758245	0.214380
C	-0.472396	3.257550	0.022671
C	0.521961	0.924963	0.137540
C	1.477942	0.170065	0.100912
C	2.593717	-0.712692	0.060516
C	3.849736	-0.254748	-0.378351
C	2.450195	-2.054165	0.461417
C	4.934171	-1.123575	-0.416516
H	3.958319	0.786753	-0.687094
C	3.541814	-2.913984	0.421560
H	1.475373	-2.405119	0.806019
C	4.783478	-2.452456	-0.017561
H	5.906455	-0.761991	-0.759270
H	3.424074	-3.953477	0.736289
H	5.638754	-3.131819	-0.048114
O	-1.371019	4.044202	0.098108
O	0.794308	3.572392	-0.242240
C	1.057098	4.951466	-0.439365
H	2.130043	5.034676	-0.646314
H	0.472310	5.341896	-1.285351
H	0.794950	5.529474	0.459122
C	-2.095230	0.000728	0.686267
O	-2.032556	-0.487914	1.784130
O	-2.500059	-0.584110	-0.433537
C	-2.962620	-1.966513	-0.452113
C	-1.831408	-2.894912	-0.025330
H	-1.567902	-2.734868	1.028113
H	-2.142671	-3.941885	-0.157382
H	-0.941096	-2.720701	-0.648797
C	-4.192453	-2.111445	0.435571
H	-4.969889	-1.395196	0.130150
H	-4.603545	-3.126933	0.333583
H	-3.943059	-1.936564	1.489828
C	-3.320054	-2.185716	-1.914610
H	-2.439093	-2.031396	-2.554658
H	-3.689507	-3.210215	-2.066626
H	-4.102249	-1.479796	-2.229366

4a'

C	2.883223	0.476951	-0.755058
C	0.733904	0.087046	-0.801738
C	1.025764	1.444891	-1.407276
O	2.391356	1.621278	-1.295224

H	0.211554	-0.559258	-1.526137
N	2.042759	-0.438231	-0.470103
C	0.256426	2.336838	-2.023958
H	0.686750	3.252996	-2.431049
H	-0.814506	2.162225	-2.114145
N	-0.470339	-1.347979	0.804151
C	-0.181032	0.025123	0.479450
C	0.609373	0.565611	1.688989
C	-1.406441	0.816257	0.321602
C	-2.443034	1.436573	0.201920
C	-3.676636	2.134744	0.024908
C	-3.867425	3.417784	0.566376
C	-4.720252	1.536675	-0.704070
C	-5.075166	4.083111	0.382001
H	-3.057466	3.883744	1.131168
C	-5.924210	2.209632	-0.883102
H	-4.567331	0.540510	-1.124598
C	-6.105854	3.482609	-0.341463
H	-5.213864	5.080291	0.806844
H	-6.728788	1.736464	-1.451256
H	-7.053060	4.008487	-0.483908
O	0.804712	-0.062543	2.694487
O	1.040631	1.795600	1.471552
C	1.809727	2.387251	2.508614
H	2.069448	3.392107	2.158030
H	1.226129	2.442684	3.438638
H	2.720337	1.799939	2.695494
C	-1.194090	-2.173464	0.005255
O	-1.631677	-1.865025	-1.084122
O	-1.337336	-3.362347	0.605732
C	-2.036327	-4.460124	-0.036977
C	-3.494527	-4.083950	-0.276191
H	-3.573509	-3.266499	-1.003818
H	-4.044864	-4.956420	-0.659401
H	-3.966849	-3.768477	0.666563
C	-1.326465	-4.847778	-1.329347
H	-0.267007	-5.069891	-1.130528
H	-1.791723	-5.750495	-1.752972
H	-1.387001	-4.038484	-2.067758
C	-1.931581	-5.578822	0.990271
H	-2.402626	-5.277802	1.937473
H	-2.434612	-6.484265	0.620963
H	-0.877442	-5.820313	1.190815
C	4.333635	0.420232	-0.550636

C	5.155870	1.490746	-0.922433
C	4.896435	-0.725384	0.028230
C	6.530878	1.413860	-0.714201
H	4.708693	2.377122	-1.375635
C	6.269351	-0.795357	0.233201
H	4.233838	-1.546713	0.308742
C	7.088551	0.273546	-0.137342
H	7.171742	2.249286	-1.005886
H	6.706669	-1.689064	0.684580
H	8.167897	0.215583	0.024108
H	-0.131438	-1.673606	1.703799

4a

C	3.548943	0.624552	-0.343597
C	1.479420	0.810671	0.091792
C	1.638054	-0.339332	-0.623263
O	2.964236	-0.460628	-0.901070
H	0.549689	1.223643	0.481555
N	2.705896	1.407460	0.257507
C	0.715627	-1.389393	-1.136676
H	1.285015	-2.320557	-1.286264
H	0.324669	-1.100953	-2.125530
N	-3.414908	0.515879	-0.651324
C	-2.820826	-0.719080	-0.900121
C	-3.733097	-1.594621	-1.700702
C	-1.628781	-1.139878	-0.514803
C	-0.452542	-1.640411	-0.200755
C	-0.232943	-2.416628	1.045822
C	1.053377	-2.813205	1.440287
C	-1.317786	-2.785211	1.858598
C	1.246964	-3.550581	2.607518
H	1.924550	-2.535418	0.844570
C	-1.124514	-3.521710	3.020332
H	-2.326793	-2.488484	1.562341
C	0.161562	-3.909082	3.402194
H	2.259397	-3.842739	2.896724
H	-1.985045	-3.797892	3.634806
H	0.314550	-4.487159	4.316677
O	-4.829548	-1.228888	-2.053256
O	-3.220017	-2.785846	-1.970473
C	-4.044739	-3.661737	-2.721314
H	-3.471575	-4.587187	-2.846522
H	-4.985507	-3.865145	-2.188964

H	-4.286426	-3.226461	-3.702117
C	-2.845412	1.555689	0.028003
O	-1.736363	1.532659	0.508731
O	-3.711560	2.573538	0.065647
C	-3.380587	3.835316	0.710268
C	-3.119224	3.610204	2.194974
H	-2.222445	2.997367	2.350216
H	-2.975680	4.579397	2.695349
H	-3.979772	3.108459	2.662972
C	-2.191610	4.481679	0.009116
H	-2.395063	4.590052	-1.067036
H	-2.017664	5.484814	0.426009
H	-1.279865	3.885406	0.140380
C	-4.641810	4.661925	0.504192
H	-5.507129	4.169240	0.971334
H	-4.521484	5.657898	0.954266
H	-4.851926	4.785730	-0.568313
C	4.991678	0.786475	-0.474206
C	5.606935	1.903071	0.111089
C	5.773078	-0.145872	-1.170832
C	6.980999	2.080679	-0.001487
H	4.982920	2.618613	0.650344
C	7.148654	0.038903	-1.279003
H	5.293918	-1.014342	-1.626941
C	7.756741	1.150471	-0.696143
H	7.453401	2.952960	0.456909
H	7.751475	-0.691669	-1.824007
H	8.836725	1.292689	-0.782965
H	-4.349788	0.624326	-1.033404

A

C	1.986608	-0.552727	-0.543148
C	0.750346	-2.631272	-0.834777
C	-0.409617	-2.549248	0.078997
O	1.019166	0.082096	-0.951933
H	0.437680	-2.274498	-1.828812
N	1.913172	-1.908353	-0.404986
C	-1.352788	-2.689300	0.863737
C	3.276022	0.090705	-0.172874
C	4.485581	-0.614335	-0.099353
C	3.268193	1.467988	0.079667
C	5.661680	0.046451	0.243379
H	4.532221	-1.678562	-0.345164

C	4.442683	2.125649	0.427648
H	2.324667	2.010303	-0.003910
C	5.640286	1.414663	0.512830
H	6.602061	-0.506893	0.289429
H	4.428734	3.198890	0.629837
H	6.564565	1.931165	0.781957
H	2.636197	-2.402364	0.101187
H	1.000700	-3.699146	-0.935828
H	-2.081335	-3.054521	1.574992
Au	-1.518770	-0.572626	0.202486
P	-2.144646	1.632387	-0.136945
C	-3.937407	1.931496	-0.163305
H	-4.140901	2.994966	-0.361379
H	-4.404449	1.320594	-0.948871
H	-4.377175	1.652876	0.804947
C	-1.490858	2.217511	-1.727667
H	-1.676398	3.295826	-1.846513
H	-0.411797	2.006169	-1.760665
H	-1.977281	1.671276	-2.548598
C	-1.467218	2.751618	1.125980
H	-0.371919	2.658352	1.142668
H	-1.741737	3.793215	0.899075
H	-1.857909	2.480508	2.117087

C

C	-2.943748	-0.858229	0.258862
C	-1.707357	-2.474746	0.841475
C	-1.041074	-1.816432	-0.160577
O	-1.844298	-0.780209	-0.528093
H	-1.384608	-3.359396	1.387411
N	-2.904028	-1.852159	1.092260
C	0.258665	-1.974060	-0.825272
H	0.135413	-1.931806	-1.920506
H	0.671004	-2.962734	-0.572908
Au	1.636728	-0.501857	-0.271411
P	3.156628	1.152514	0.361836
C	2.836841	1.889629	2.004333
H	3.577157	2.667350	2.248240
H	1.828598	2.328442	2.014569
H	2.870454	1.100581	2.769516
C	3.223446	2.599682	-0.754344
H	3.952691	3.346373	-0.403516

H	3.499750	2.267078	-1.765420
H	2.226808	3.061211	-0.808949
C	4.899395	0.607550	0.465298
H	5.219212	0.223480	-0.514232
H	5.562160	1.433158	0.767869
H	4.981025	-0.211358	1.194621
C	-3.997534	0.131295	0.086054
C	-5.135927	0.075581	0.905627
C	-3.899472	1.141068	-0.882699
C	-6.151367	1.013089	0.757116
H	-5.198811	-0.717346	1.653757
C	-4.921164	2.075922	-1.025668
H	-3.015907	1.183150	-1.522557
C	-6.049589	2.017404	-0.208152
H	-7.033681	0.960764	1.400137
H	-4.836107	2.857945	-1.784599
H	-6.850174	2.752362	-0.322855

Int1

C	-0.312629	-3.402257	1.126956
C	0.366157	-1.377893	0.706803
C	1.463838	-2.135699	1.375621
O	1.023322	-3.368232	1.561126
H	0.689029	-1.129098	-0.337622
N	-0.732531	-2.298750	0.670082
C	2.785581	-1.805353	1.700275
H	3.325113	-2.568650	2.270032
H	2.909946	-0.768929	2.039899
Au	3.578537	-1.447760	-0.281373
P	4.182168	-0.728411	-2.382457
C	4.510196	1.062981	-2.391936
H	4.739564	1.409229	-3.411416
H	3.612809	1.579926	-2.021304
H	5.349335	1.299886	-1.722711
C	2.811548	-0.951227	-3.554777
H	3.050253	-0.494112	-4.527394
H	2.608998	-2.023022	-3.692626
H	1.920409	-0.473499	-3.113137
C	-3.562664	-2.570179	-2.824003
C	-5.120713	-3.909997	-1.861206
C	-3.659079	-1.620459	-1.805331
H	-2.868196	-2.403256	-3.656807

C	-5.289386	-3.035346	-0.791359
C	-4.540867	-1.852777	-0.749965
H	-3.058031	-0.708428	-1.832163
N	-4.269123	-3.696155	-2.863735
C	-4.703678	-0.897095	0.406432
H	-5.756447	-0.573598	0.459343
N	-3.883188	0.281074	0.343488
C	-4.415818	1.500562	0.348559
C	-3.876238	2.793314	0.443044
C	-5.799540	2.008073	0.348253
C	-5.221842	3.418414	0.326206
O	-5.676693	4.537521	0.260051
O	-6.896351	1.493951	0.354739
N	-2.581914	3.149786	0.552058
C	-2.016166	4.417916	0.592312
C	-2.768520	5.598236	0.475415
C	-0.622083	4.509395	0.757836
C	-2.125023	6.831803	0.521866
H	-3.854636	5.543147	0.356012
C	0.002224	5.751840	0.804209
H	-0.038291	3.591342	0.865514
C	-0.742341	6.925398	0.684142
H	-2.726079	7.740533	0.430692
H	1.085471	5.800348	0.946147
H	-0.251954	7.900774	0.723254
H	-5.710136	-4.834071	-1.910854
H	-6.006963	-3.268752	0.000721
H	-4.499871	-1.452175	1.341676
H	-1.928100	2.350814	0.579829
H	-2.852883	0.217709	0.392124
N	-0.949835	0.661318	0.238677
C	-0.058397	0.067796	1.176956
C	-0.785508	-0.076366	2.532277
C	1.137812	0.903080	1.401871
C	2.062664	1.679422	1.550422
C	3.161449	2.585669	1.662090
C	4.086626	2.479785	2.715832
C	3.335724	3.600556	0.702328
C	5.157926	3.363468	2.802592
H	3.950782	1.701704	3.470111
C	4.408229	4.481597	0.798664
H	2.611266	3.693841	-0.109827
C	5.322813	4.365541	1.846279
H	5.867798	3.273168	3.628343

H	4.527932	5.270560	0.052067
H	6.161875	5.061377	1.920965
O	-1.817780	0.447015	2.827341
O	-0.088925	-0.876210	3.351508
C	-0.632235	-1.056254	4.651188
H	0.056519	-1.721956	5.184013
H	-0.712516	-0.091800	5.173018
H	-1.633417	-1.507370	4.594356
C	-0.519120	0.753696	-1.017191
O	0.564251	0.349994	-1.488251
O	-1.460626	1.338979	-1.792907
C	-1.209343	1.835739	-3.123273
C	-0.015440	2.787015	-3.125958
H	0.917196	2.252585	-2.903554
H	0.075204	3.268644	-4.111302
H	-0.160751	3.575822	-2.371718
C	-1.020454	0.677031	-4.098089
H	-1.905086	0.022416	-4.090030
H	-0.895032	1.060832	-5.122125
H	-0.140248	0.078987	-3.832030
C	-2.485122	2.601363	-3.453657
H	-2.634511	3.423909	-2.739575
H	-2.430101	3.020655	-4.469116
H	-3.358955	1.936074	-3.396602
C	5.647496	-1.497524	-3.146359
H	5.833129	-1.073063	-4.145020
H	6.527896	-1.328648	-2.509853
H	5.487994	-2.581483	-3.237285
C	-0.999596	-4.680137	1.260349
C	-0.393907	-5.765803	1.907626
C	-2.288783	-4.811620	0.723356
C	-1.079447	-6.971539	2.022579
H	0.611078	-5.660614	2.319802
C	-2.963833	-6.020184	0.841579
H	-2.745003	-3.962811	0.209430
C	-2.362574	-7.100119	1.491351
H	-0.608470	-7.817080	2.529030
H	-3.965071	-6.119668	0.416675
H	-2.896810	-8.049182	1.580535

Int2

C	-3.070282	-0.372770	-0.420810

C	-0.946203	-0.791115	-0.140296
C	-1.113298	0.324450	-1.140829
O	-2.413112	0.540301	-1.262255
H	-0.380472	-0.427057	0.740347
N	-2.292416	-1.136147	0.225937
C	-0.192049	1.116961	-1.812953
H	0.799929	0.672184	-1.951524
N	0.049258	-2.899515	0.599715
C	-0.124739	-2.064284	-0.554318
C	-0.954439	-2.888218	-1.561908
C	1.157980	-1.722284	-1.181759
C	2.246622	-1.526800	-1.686208
C	3.531235	-1.278707	-2.259933
C	3.680573	-1.147197	-3.651914
C	4.662242	-1.167380	-1.431137
C	4.936694	-0.908220	-4.198936
H	2.805082	-1.241171	-4.297715
C	5.913877	-0.930019	-1.988472
H	4.545309	-1.276000	-0.350892
C	6.054065	-0.799318	-3.370649
H	5.046458	-0.810946	-5.281423
H	6.789450	-0.850407	-1.340042
H	7.039626	-0.615886	-3.804874
O	-1.165855	-4.060847	-1.445111
O	-1.381784	-2.121029	-2.552333
C	-2.140668	-2.769156	-3.573831
H	-2.414943	-1.987865	-4.290446
H	-1.534487	-3.544063	-4.062799
H	-3.039205	-3.233475	-3.144554
C	0.697119	-2.484550	1.724699
O	1.070087	-1.336068	1.897282
O	0.830493	-3.496361	2.569215
C	1.449819	-3.345816	3.884929
C	2.902396	-2.917759	3.721129
H	2.972292	-1.914966	3.280712
H	3.395908	-2.908257	4.704078
H	3.441215	-3.629712	3.078082
C	0.645584	-2.359460	4.721901
H	-0.408626	-2.670615	4.773420
H	1.043239	-2.337778	5.747165
H	0.699253	-1.346967	4.301969
C	1.359073	-4.748626	4.465200
H	1.898378	-5.466227	3.830283
H	1.802534	-4.771053	5.470743

H	0.310428	-5.071137	4.539162
C	-4.523826	-0.313252	-0.419639
C	-5.215596	0.602512	-1.225223
C	-5.231458	-1.200403	0.406648
C	-6.606719	0.626722	-1.202177
H	-4.663459	1.290846	-1.867289
C	-6.619622	-1.167911	0.422639
H	-4.675109	-1.906707	1.026176
C	-7.308090	-0.255493	-0.380668
H	-7.147630	1.338355	-1.829599
H	-7.171861	-1.857825	1.064320
H	-8.400329	-0.233220	-0.365625
H	-0.240939	-3.868435	0.501941
H	-0.587566	1.729944	-2.629744
Au	0.261137	2.448982	-0.147420
P	0.950714	3.825735	1.572086
C	0.907128	2.973348	3.180046
H	1.309370	3.622752	3.972614
H	-0.129742	2.701644	3.424775
H	1.500608	2.049152	3.122140
C	2.668202	4.392095	1.365895
H	2.759635	4.973248	0.437074
H	2.970283	5.019764	2.218249
H	3.337459	3.522304	1.299958
C	-0.039142	5.337900	1.783242
H	0.346154	5.933085	2.625243
H	0.003806	5.939621	0.864132
H	-1.087953	5.071471	1.976849

Int3

C	-4.180166	-0.126336	-0.505205
C	-2.059626	0.035701	-1.009760
C	-2.580967	-1.330015	-1.400323
O	-3.888249	-1.368046	-0.973932
H	-1.607501	0.543297	-1.876920
N	-3.228778	0.728929	-0.518512
C	-2.043369	-2.322816	-2.104605
H	-1.028091	-2.237706	-2.488280
N	-0.489409	1.490120	0.230549
C	-0.953501	0.135319	0.119253
C	-1.608894	-0.190126	1.484934
C	0.148181	-0.831978	-0.108380

C	0.719287	-1.889453	-0.421952
C	1.298854	-3.137759	-0.809024
C	1.181428	-4.257243	0.035157
C	1.961758	-3.255295	-2.045465
C	1.716214	-5.477081	-0.361010
H	0.660833	-4.159615	0.989700
C	2.493521	-4.480665	-2.428076
H	2.046844	-2.382673	-2.696909
C	2.371455	-5.589597	-1.588715
H	1.620073	-6.348567	0.290114
H	3.003578	-4.574544	-3.389172
H	2.789057	-6.551494	-1.895274
O	-1.648094	0.589813	2.395283
O	-2.112715	-1.407219	1.490205
C	-2.827551	-1.796778	2.662430
H	-3.192780	-2.810753	2.469410
H	-2.163325	-1.783021	3.537778
H	-3.668893	-1.112394	2.839182
C	0.183692	2.116277	-0.776783
O	0.602871	1.533302	-1.758542
O	0.336519	3.407035	-0.493229
C	0.841536	4.361972	-1.480613
C	2.300032	4.056098	-1.799134
H	2.400682	3.072973	-2.277498
H	2.695842	4.820893	-2.483305
H	2.908413	4.085777	-0.881306
C	-0.034153	4.328422	-2.726155
H	-1.088875	4.487425	-2.456368
H	0.266704	5.137498	-3.407444
H	0.059795	3.372991	-3.257778
C	0.709979	5.695798	-0.762231
H	1.297692	5.699367	0.167652
H	1.073160	6.509604	-1.405747
H	-0.340394	5.896392	-0.507107
C	-5.546138	0.091148	-0.032125
C	-6.502197	-0.930852	-0.104281
C	-5.894573	1.342309	0.497002
C	-7.796411	-0.700151	0.353170
H	-6.225917	-1.899494	-0.524201
C	-7.188615	1.564551	0.951395
H	-5.135309	2.125592	0.540231
C	-8.140175	0.544452	0.880278
H	-8.543089	-1.495188	0.294810
H	-7.461317	2.539127	1.362358

H	-9.157646	0.722882	1.236430
H	-0.902978	2.034873	0.981690
H	-2.618931	-3.222139	-2.328258
Au	2.198233	-0.288930	0.466638
P	4.070727	0.822799	1.219783
C	5.100975	-0.126445	2.379514
H	5.469830	-1.038082	1.888186
H	5.957832	0.479080	2.712582
H	4.501135	-0.418607	3.253193
C	5.179601	1.345598	-0.123418
H	6.034173	1.909570	0.280740
H	5.550156	0.462123	-0.662662
H	4.624092	1.978460	-0.830399
C	3.597239	2.343318	2.100368
H	4.492298	2.911529	2.396046
H	2.963474	2.962952	1.449344
H	3.018240	2.082737	2.997995

Int4

C	4.243694	-0.164490	0.267617
C	2.143684	0.246654	0.619784
C	2.436478	-1.094835	1.052035
O	3.683358	-1.347482	0.874171
H	1.735021	0.874709	1.477847
N	3.376448	0.746103	0.104014
C	1.462357	-2.037934	1.571652
H	1.796598	-3.074074	1.399767
H	1.417152	-1.911865	2.671773
N	0.428374	1.650390	-0.408555
C	0.873622	0.294550	-0.303943
C	1.269232	-0.143342	-1.720637
C	-0.200096	-0.682008	0.181232
C	0.083657	-1.743748	0.967032
C	-0.923409	-2.775734	1.327798
C	-1.139674	-3.134229	2.666666
C	-1.659441	-3.436789	0.332091
C	-2.086172	-4.098968	3.003653
H	-0.584950	-2.634172	3.465767
C	-2.597217	-4.410785	0.668429
H	-1.473321	-3.190841	-0.716145
C	-2.817352	-4.741386	2.005150
H	-2.253346	-4.350719	4.053544
H	-3.151486	-4.925089	-0.120667

H	-3.551151	-5.506561	2.268924
O	0.939139	0.437259	-2.716541
O	1.995504	-1.253836	-1.686363
C	2.379175	-1.802739	-2.947271
H	2.991601	-2.682725	-2.723706
H	1.487986	-2.091614	-3.521177
H	2.955396	-1.066718	-3.524568
C	0.171259	2.420466	0.679913
O	0.445695	2.059404	1.818412
O	-0.374222	3.570769	0.333878
C	-0.653938	4.635560	1.304772
C	-1.679411	4.145093	2.317522
H	-1.272436	3.337778	2.939324
H	-1.974703	4.978111	2.972087
H	-2.582213	3.780923	1.803900
C	0.643126	5.084308	1.963038
H	1.380211	5.379158	1.201096
H	0.445752	5.959619	2.598846
H	1.070158	4.290587	2.588945
C	-1.231264	5.739146	0.432623
H	-2.142726	5.395034	-0.077736
H	-1.487493	6.612029	1.049436
H	-0.503967	6.053342	-0.329795
C	5.659353	-0.217862	-0.038976
C	6.425179	-1.362481	0.229082
C	6.257076	0.914389	-0.616604
C	7.780618	-1.370206	-0.082567
H	5.961944	-2.241147	0.681098
C	7.610847	0.894454	-0.923783
H	5.646876	1.797946	-0.814315
C	8.372818	-0.245770	-0.657573
H	8.379713	-2.258978	0.126063
H	8.078737	1.773485	-1.371955
H	9.438209	-0.256355	-0.899270
H	0.271244	2.012009	-1.344620
Au	-2.129456	-0.264479	-0.457660
P	-4.306386	0.243253	-1.108410
C	-5.446340	0.503258	0.293134
H	-6.462334	0.735510	-0.061176
H	-5.475942	-0.403855	0.913918
H	-5.079747	1.332313	0.915446
C	-5.112586	-1.048967	-2.113802
H	-5.134762	-1.990684	-1.546492
H	-6.141056	-0.759538	-2.378680

H	-4.533532	-1.213320	-3.033932
C	-4.456633	1.761128	-2.109870
H	-3.861523	1.654634	-3.028327
H	-5.506512	1.955023	-2.377410
H	-4.062005	2.616322	-1.542376

Int5

C	-4.158542	0.940586	-0.406077
C	-2.617770	2.350677	-0.755329
C	-2.311007	1.235230	-1.487318
O	-3.302569	0.338041	-1.259921
H	-2.073502	3.292632	-0.703538
N	-3.788517	2.143222	-0.075223
C	-1.192877	0.820659	-2.359582
H	-1.596277	0.368846	-3.278133
H	-0.613562	1.711646	-2.650538
N	0.520438	2.220952	0.689455
C	-0.108991	1.052329	0.529055
C	-1.083715	0.822176	1.684028
C	0.156423	0.065657	-0.429802
C	-0.249766	-0.175371	-1.681874
C	0.201196	-1.346362	-2.464369
C	0.633751	-1.190528	-3.789963
C	0.189408	-2.635556	-1.908314
C	1.078835	-2.286923	-4.524001
H	0.648135	-0.197203	-4.245409
C	0.624522	-3.733277	-2.649026
H	-0.215469	-2.777534	-0.902294
C	1.077417	-3.561092	-3.956426
H	1.427879	-2.145183	-5.549445
H	0.586908	-4.733683	-2.210446
H	1.413618	-4.421467	-4.539657
O	-1.123210	1.587702	2.612228
O	-1.827726	-0.243403	1.519793
C	-2.797099	-0.500365	2.540286
H	-3.350571	-1.385761	2.212224
H	-2.293922	-0.684306	3.499895
H	-3.473536	0.358953	2.640655
C	1.429274	2.827631	-0.205716
O	1.696154	2.364829	-1.280139
O	1.870389	3.926341	0.363165
C	2.808496	4.843496	-0.317334
C	4.114153	4.110398	-0.586306
H	3.977603	3.294498	-1.307946

H	4.846790	4.818532	-0.999693
H	4.528858	3.703582	0.348209
C	2.160594	5.369234	-1.589439
H	1.191564	5.839849	-1.365395
H	2.811116	6.136132	-2.034071
H	2.014228	4.572324	-2.329990
C	2.995172	5.950325	0.705827
H	3.413039	5.551463	1.641368
H	3.687306	6.707958	0.312492
H	2.037218	6.440275	0.931632
C	-5.337689	0.209954	0.036398
C	-5.571211	-1.110004	-0.376416
C	-6.248780	0.843605	0.895283
C	-6.702045	-1.786625	0.071133
H	-4.866078	-1.598170	-1.052098
C	-7.375487	0.159682	1.337437
H	-6.058024	1.875343	1.198187
C	-7.604095	-1.155596	0.928351
H	-6.884578	-2.812982	-0.255432
H	-8.085424	0.656935	2.002262
H	-8.492440	-1.688856	1.274911
H	0.305738	2.707321	1.564371
Au	1.575880	-1.107698	0.601737
P	3.226047	-2.451217	1.544532
C	4.912237	-1.827750	1.251816
H	5.658024	-2.510258	1.687351
H	5.089528	-1.737538	0.170496
H	5.022215	-0.832786	1.706248
C	3.216574	-4.141421	0.866397
H	3.333092	-4.100035	-0.226325
H	4.034053	-4.737484	1.299924
H	2.255606	-4.624460	1.094554
C	3.096638	-2.655495	3.350127
H	2.121876	-3.095942	3.604034
H	3.898997	-3.310812	3.722460
H	3.173301	-1.674400	3.840189

QN-SQA

C	4.512372	-1.506757	-1.472690
C	5.923814	-1.046062	0.247344
C	3.470970	-0.788100	-0.887386
H	4.359086	-1.999467	-2.440128
C	4.951521	-0.307027	0.920358

C	3.687361	-0.165378	0.344281
H	2.502982	-0.712948	-1.388506
N	5.717195	-1.639923	-0.924419
C	2.616215	0.653370	1.019819
H	2.854232	0.752359	2.093399
N	1.285699	0.109913	0.839489
C	0.254231	0.846855	0.407861
C	-1.107090	0.615508	0.180819
C	0.174549	2.241482	-0.073765
C	-1.325639	2.025098	-0.270082
O	-2.253193	2.698202	-0.635102
O	0.971292	3.133480	-0.223133
N	-1.837284	-0.504291	0.374828
C	-3.201477	-0.750995	0.168264
C	-4.089740	0.224824	-0.300003
C	-3.674568	-2.040941	0.450372
C	-5.430965	-0.105229	-0.478498
H	-3.733244	1.233833	-0.521648
C	-5.017111	-2.353229	0.266433
H	-2.983230	-2.806330	0.816951
C	-5.906167	-1.386280	-0.200245
H	-6.115870	0.663554	-0.844623
H	-5.368311	-3.363313	0.490744
H	-6.960756	-1.629530	-0.345590
H	6.920682	-1.166383	0.687704
H	5.181488	0.150661	1.886435
H	2.604661	1.675123	0.604377
H	-1.325404	-1.312112	0.708768
H	1.129971	-0.841636	1.151408

TS2

C	4.581486	1.340825	0.779749
C	2.503939	0.945794	1.024819
C	2.891189	0.412451	-0.237648
O	4.182742	0.744097	-0.392699
H	1.663015	0.585083	1.609586
N	3.641364	1.427690	1.655629
C	2.195670	-0.279443	-1.272453
H	2.658387	-0.103726	-2.254419
H	1.121173	-0.028472	-1.275693
Au	2.278227	-2.371590	-0.946636

P	2.286861	-4.686296	-0.736113
C	0.736795	-5.454299	-1.308288
H	0.791371	-6.551672	-1.240272
H	-0.070564	-5.083131	-0.660607
H	0.530229	-5.158483	-2.346533
C	2.497672	-5.336633	0.952008
H	2.590008	-6.433719	0.936305
H	3.391020	-4.899601	1.419901
H	1.596982	-5.050185	1.519400
C	-0.106739	-2.822031	2.391917
C	-1.810703	-4.154479	1.673187
C	-0.987726	-1.947968	3.023710
H	0.973570	-2.635185	2.427762
C	-2.775082	-3.340466	2.261748
C	-2.359470	-2.202281	2.959232
H	-0.605967	-1.074673	3.559881
N	-0.500574	-3.907312	1.726474
C	-3.372894	-1.258627	3.566808
H	-4.240447	-1.834358	3.918514
N	-3.830021	-0.264939	2.621250
C	-4.912905	-0.442527	1.868587
C	-5.515033	0.302200	0.840519
C	-5.923665	-1.507099	1.779825
C	-6.665999	-0.642759	0.768212
O	-7.717722	-0.713863	0.177941
O	-6.062045	-2.599985	2.290283
N	-5.079058	1.437437	0.267740
C	-5.680146	2.211448	-0.722518
C	-6.977141	1.974123	-1.202320
C	-4.939041	3.282993	-1.248993
C	-7.507510	2.804261	-2.186989
H	-7.558668	1.132820	-0.815358
C	-5.484817	4.101448	-2.232001
H	-3.927769	3.465512	-0.876746
C	-6.775023	3.870126	-2.709584
H	-8.518839	2.606416	-2.551889
H	-4.892093	4.930440	-2.627588
H	-7.204378	4.513447	-3.480903
H	-2.116635	-5.056465	1.130146
H	-3.841945	-3.575155	2.184902
H	-2.929960	-0.739106	4.430122
H	-4.077006	1.628765	0.421487
H	-3.260134	0.576575	2.498698
N	-2.061022	1.526186	0.265945

C	-1.072820	2.164685	0.881191
C	-1.439626	2.669203	2.257827
C	0.213602	2.406763	0.477685
C	1.448577	2.568313	0.272593
C	2.395752	3.388525	-0.454048
C	3.382080	4.145375	0.197183
C	2.307120	3.430551	-1.857882
C	4.262078	4.931117	-0.543875
H	3.443938	4.116388	1.286211
C	3.187770	4.220205	-2.588912
H	1.525315	2.846154	-2.348857
C	4.169107	4.967868	-1.934451
H	5.025345	5.520175	-0.029934
H	3.106431	4.257074	-3.678082
H	4.860737	5.586403	-2.511903
O	-2.301807	2.169948	2.943836
O	-0.709787	3.700894	2.648196
C	-0.974184	4.204660	3.947890
H	-0.286179	5.044584	4.094734
H	-2.016777	4.544978	4.027976
H	-0.800209	3.430043	4.709191
C	-1.798878	0.955564	-0.957762
O	-0.750855	1.012212	-1.581598
O	-2.889873	0.310099	-1.381489
C	-2.954960	-0.360128	-2.664763
C	-2.749474	0.645216	-3.792916
H	-1.733497	1.059656	-3.774063
H	-2.912753	0.151078	-4.762517
H	-3.474510	1.467984	-3.700941
C	-1.939431	-1.498127	-2.714878
H	-2.076662	-2.166621	-1.850879
H	-2.092467	-2.087094	-3.631864
H	-0.910764	-1.115625	-2.708287
C	-4.373893	-0.912468	-2.694025
H	-5.109532	-0.098236	-2.622426
H	-4.549357	-1.461072	-3.630979
H	-4.539388	-1.598940	-1.850645
C	3.598808	-5.491506	-1.721492
H	3.548253	-6.586839	-1.621353
H	3.485741	-5.217636	-2.780398
H	4.583238	-5.140921	-1.379222
C	5.970074	1.759053	0.888564
C	6.856630	1.622808	-0.188898
C	6.424761	2.308580	2.097247

C	8.180831	2.030086	-0.054526
H	6.498626	1.201553	-1.129823
C	7.748375	2.712317	2.222665
H	5.721292	2.406220	2.926676
C	8.630238	2.574367	1.148231
H	8.867721	1.923566	-0.897477
H	8.098391	3.137786	3.166238
H	9.670476	2.893105	1.249806

TS1

C	1.057189	2.851911	0.974413
C	0.604551	0.764568	0.931187
C	1.333505	1.083335	-0.262679
O	1.515430	2.402298	-0.257063
H	0.699606	-0.211693	1.408353
N	0.602684	1.919041	1.723506
C	1.883516	0.253690	-1.273095
H	1.987079	0.745016	-2.247779
H	1.373385	-0.717746	-1.325390
Au	3.831107	-0.212425	-0.536394
P	5.951894	-0.792065	0.207536
C	6.637863	-2.241871	-0.661211
H	7.640156	-2.489124	-0.278987
H	5.965619	-3.096612	-0.501344
H	6.695929	-2.038743	-1.740019
C	6.041964	-1.237331	1.968459
H	7.073657	-1.499467	2.249925
H	5.698138	-0.392171	2.582096
H	5.368275	-2.098862	2.127967
C	2.443780	-2.844601	2.071342
C	3.439491	-4.381059	0.722032
C	1.177964	-3.121752	1.555752
H	2.555338	-2.086248	2.855498
C	2.229021	-4.721818	0.128817
C	1.058380	-4.077006	0.543811
H	0.298366	-2.589056	1.927918
N	3.561073	-3.448382	1.667455
C	-0.264783	-4.428640	-0.084617
H	-0.105638	-4.567184	-1.171247
N	-1.307973	-3.469828	0.157731
C	-2.519657	-3.849020	0.557494
C	-3.753371	-3.191268	0.666450

C	-3.094391	-5.138338	0.982948
C	-4.415921	-4.409450	1.210219
O	-5.507471	-4.710424	1.631642
O	-2.665045	-6.263969	1.097315
N	-4.041319	-1.904537	0.375448
C	-5.251371	-1.217220	0.480565
C	-6.415749	-1.804350	0.997762
C	-5.286502	0.120390	0.052055
C	-7.585182	-1.052328	1.078537
H	-6.402704	-2.846190	1.330726
C	-6.463532	0.855487	0.141757
H	-4.381507	0.587837	-0.344589
C	-7.623701	0.276308	0.655768
H	-8.485407	-1.523578	1.481774
H	-6.468295	1.895116	-0.195763
H	-8.548236	0.854045	0.724213
H	4.361334	-4.887928	0.411435
H	2.199267	-5.484311	-0.654378
H	-0.605537	-5.405080	0.301876
H	-3.255334	-1.382653	-0.028778
H	-1.183506	-2.478096	-0.109722
N	-1.376967	-0.571139	-0.440215
C	-1.241578	0.491938	0.407556
C	-1.714759	0.056548	1.809820
C	-1.659783	1.817694	0.017320
C	-1.977372	2.954639	-0.276868
C	-2.359352	4.257596	-0.710534
C	-2.518262	5.313043	0.204903
C	-2.574641	4.498070	-2.080657
C	-2.887321	6.577207	-0.242909
H	-2.345765	5.127302	1.266703
C	-2.947170	5.764389	-2.517679
H	-2.440793	3.674874	-2.785549
C	-3.104876	6.805971	-1.601996
H	-3.007684	7.391790	0.475370
H	-3.115659	5.941838	-3.582705
H	-3.398210	7.799785	-1.949257
O	-1.234964	-0.863394	2.420068
O	-2.747051	0.767425	2.219231
C	-3.345280	0.366275	3.445283
H	-4.158642	1.075953	3.630799
H	-3.746169	-0.653540	3.357668
H	-2.607365	0.394501	4.259585
C	-1.312407	-0.344835	-1.786803

O	-0.994907	0.683622	-2.357357
O	-1.646639	-1.487075	-2.426611
C	-1.731930	-1.563264	-3.867979
C	-2.791873	-0.595892	-4.386020
H	-2.492944	0.444675	-4.207345
H	-2.936553	-0.743689	-5.466852
H	-3.753369	-0.781259	-3.883474
C	-0.366950	-1.299012	-4.496675
H	0.384961	-1.986109	-4.077891
H	-0.416593	-1.470307	-5.582674
H	-0.048220	-0.265398	-4.312308
C	-2.162992	-3.003756	-4.111638
H	-3.122582	-3.207112	-3.614476
H	-2.277783	-3.194098	-5.188734
H	-1.414834	-3.704205	-3.711197
C	7.206220	0.514634	-0.027126
H	8.192858	0.177182	0.326218
H	7.271349	0.780491	-1.092016
H	6.908432	1.411952	0.534285
C	1.169142	4.276354	1.250005
C	1.664855	5.168694	0.290108
C	0.762521	4.756529	2.504323
C	1.754500	6.526007	0.586155
H	1.969717	4.793619	-0.688283
C	0.858721	6.112451	2.792364
H	0.374045	4.046023	3.236825
C	1.354537	7.000414	1.834744
H	2.136210	7.219714	-0.166515
H	0.543881	6.482732	3.770957
H	1.426481	8.066606	2.063088

TS3

C	-2.765063	-0.784554	2.385323
C	-1.290443	0.075422	1.125019
C	-2.109912	-0.702584	0.320750
O	-3.043722	-1.254218	1.145757
H	-0.449823	0.692339	0.816363
N	-1.735807	0.008622	2.416768
C	-2.014627	-1.158758	-1.042799
H	-2.836642	-1.748645	-1.455945
H	-1.219164	-0.741495	-1.679290
Au	-3.078711	0.836097	-1.477266
P	-4.134785	2.792168	-2.022889

C	-3.040347	4.055872	-2.729681
H	-3.619191	4.949123	-3.011267
H	-2.304512	4.310665	-1.945617
H	-2.521465	3.658485	-3.613216
C	-4.888882	3.601348	-0.575778
H	-5.402023	4.529156	-0.872373
H	-5.604773	2.921362	-0.093069
H	-4.086348	3.838665	0.137153
C	0.023291	3.366964	-0.472681
C	-1.137763	4.243545	1.277160
C	1.068651	2.962738	0.353091
H	0.072807	3.160389	-1.547836
C	-0.152311	3.872684	2.186892
C	0.989105	3.210476	1.725363
H	1.931859	2.439502	-0.062041
N	-1.067703	3.994207	-0.031260
C	2.089832	2.787896	2.670295
H	2.772982	3.640742	2.828682
N	2.868126	1.683088	2.177672
C	4.164252	1.800492	1.881644
C	5.104177	0.950252	1.277734
C	5.129441	2.903214	2.003634
C	6.196265	1.949635	1.474593
O	7.390241	2.000935	1.300348
O	5.064955	4.054217	2.376389
N	4.921281	-0.286253	0.782362
C	5.847823	-1.151585	0.203996
C	7.225657	-0.890506	0.167798
C	5.359342	-2.341593	-0.362066
C	8.084033	-1.813017	-0.425567
H	7.618205	0.038828	0.589339
C	6.231506	-3.251414	-0.949463
H	4.284801	-2.538441	-0.340476
C	7.602629	-2.995999	-0.986268
H	9.155085	-1.594988	-0.447675
H	5.831601	-4.171647	-1.383669
H	8.287856	-3.711079	-1.447193
H	-2.036383	4.763015	1.630556
H	-0.281783	4.088919	3.250505
H	1.644760	2.544688	3.650475
H	3.932686	-0.557671	0.658294
H	2.437918	0.752853	2.150451
N	2.041792	-0.789179	0.049610
C	1.116293	-1.602192	0.549199

C	1.115487	-1.694693	2.053694
C	0.237394	-2.393441	-0.151793
C	-0.732601	-2.761878	-0.868378
C	-1.303496	-3.873608	-1.602769
C	-1.469388	-5.111678	-0.959529
C	-1.682586	-3.752039	-2.949768
C	-1.994659	-6.199364	-1.651151
H	-1.174737	-5.203797	0.087908
C	-2.207866	-4.843263	-3.635022
H	-1.543942	-2.797180	-3.462438
C	-2.367404	-6.069708	-2.988897
H	-2.114592	-7.157668	-1.139676
H	-2.488948	-4.737940	-4.685822
H	-2.780310	-6.924853	-3.529353
O	1.740580	-0.942728	2.764185
O	0.341309	-2.665018	2.517541
C	0.222678	-2.744164	3.929966
H	-0.451223	-3.584115	4.132839
H	1.204694	-2.919204	4.392817
H	-0.199253	-1.810805	4.329790
C	1.913274	-0.326099	-1.232044
O	0.874869	-0.184624	-1.863451
O	3.116683	0.044558	-1.690090
C	3.338185	0.515037	-3.042051
C	2.949647	-0.573709	-4.036810
H	1.872635	-0.781753	-3.994312
H	3.208825	-0.255844	-5.057852
H	3.501158	-1.500056	-3.816510
C	2.577220	1.813423	-3.293130
H	2.843788	2.565065	-2.534193
H	2.855200	2.218006	-4.278010
H	1.493143	1.644584	-3.270298
C	4.840458	0.762184	-3.077540
H	5.388939	-0.161585	-2.843333
H	5.145913	1.113911	-4.073914
H	5.125361	1.522519	-2.335651
C	-5.497850	2.572250	-3.214431
H	-5.986285	3.535837	-3.426762
H	-5.101923	2.152462	-4.150221
H	-6.238506	1.869937	-2.805886
C	-3.616304	-1.191379	3.491864
C	-4.666210	-2.103417	3.306739
C	-3.378022	-0.662690	4.770735
C	-5.461558	-2.478541	4.385528

H	-4.848941	-2.517120	2.313172
C	-4.176304	-1.043991	5.842646
H	-2.561117	0.050761	4.898030
C	-5.220318	-1.952398	5.654681
H	-6.276514	-3.190674	4.234752
H	-3.985089	-0.628567	6.835055
H	-5.846296	-2.250269	6.499287

TS4

C	2.419113	-0.233913	-0.889031
C	0.276684	-0.397636	-1.128322
C	0.568462	0.002265	0.276835
O	1.966792	-0.083483	0.374895
H	-0.549930	0.093626	-1.651384
N	1.516005	-0.358000	-1.797464
C	-0.194410	-0.421146	1.393031
H	0.255850	-0.346036	2.387747
H	-1.277563	-0.299888	1.308902
N	-1.685041	-1.997672	-1.872184
C	-0.428929	-2.078654	-1.216675
C	0.526748	-2.839949	-2.143069
C	-0.422633	-2.544695	0.120140
C	-0.303656	-2.447638	1.357896
C	-0.286110	-2.978718	2.703134
C	-1.422666	-3.627803	3.210058
C	0.859030	-2.853156	3.505659
C	-1.405206	-4.149250	4.500279
H	-2.312653	-3.721771	2.584705
C	0.867012	-3.380743	4.792747
H	1.746979	-2.355802	3.107625
C	-0.263967	-4.027169	5.292548
H	-2.290450	-4.657364	4.889273
H	1.763994	-3.290837	5.409573
H	-0.254760	-4.439519	6.304072
O	0.247366	-3.074453	-3.286585
O	1.627611	-3.206492	-1.522386
C	2.597439	-3.891328	-2.312212
H	3.412667	-4.153428	-1.629833
H	2.159162	-4.795115	-2.757262
H	2.964007	-3.235491	-3.114001
C	-2.748262	-1.302765	-1.374115
O	-2.700135	-0.665273	-0.337388

O	-3.788687	-1.426730	-2.183875
C	-5.083362	-0.804350	-1.906057
C	-5.652265	-1.363918	-0.609198
H	-5.035943	-1.078137	0.252736
H	-6.669895	-0.975520	-0.456861
H	-5.712891	-2.461362	-0.659133
C	-4.929020	0.710591	-1.864342
H	-4.458311	1.073064	-2.790864
H	-5.921728	1.177439	-1.784445
H	-4.323379	1.023847	-1.004244
C	-5.925248	-1.230069	-3.098752
H	-5.990274	-2.326121	-3.155170
H	-6.943076	-0.825353	-3.006884
H	-5.486007	-0.860614	-4.036729
C	3.863523	-0.213850	-1.097040
C	4.748464	-0.103081	-0.015774
C	4.363078	-0.309392	-2.404682
C	6.121304	-0.093844	-0.243511
H	4.355214	-0.027616	0.999538
C	5.735324	-0.297444	-2.623815
H	3.657383	-0.385065	-3.234661
C	6.615321	-0.191367	-1.544441
H	6.811632	-0.010645	0.598819
H	6.124387	-0.368872	-3.641950
H	7.693796	-0.183165	-1.719494
H	-1.731507	-2.418366	-2.797000
Au	0.005897	2.107775	0.559538
P	-0.452405	4.374041	0.701896
C	-2.114533	4.731604	1.352857
H	-2.288612	5.817797	1.393141
H	-2.871836	4.265015	0.706713
H	-2.213822	4.310452	2.363515
C	-0.367369	5.217250	-0.909395
H	-0.587090	6.289806	-0.795396
H	0.638120	5.096844	-1.337526
H	-1.095119	4.769319	-1.601046
C	0.700667	5.277181	1.782813
H	0.444855	6.347497	1.809535
H	0.651621	4.866969	2.801619
H	1.727863	5.158617	1.409270

TS5

C	3.942840	0.661356	0.145344

C	1.945465	1.511854	0.387887
C	2.322685	0.736524	1.609634
O	3.541913	0.193420	1.373937
H	1.725373	2.559212	0.641507
N	3.111328	1.420087	-0.453852
C	1.626010	0.550919	2.745173
H	2.024844	-0.063508	3.553649
H	0.726066	1.149647	2.892523
N	-0.302824	2.098569	-0.581362
C	0.610919	1.016040	-0.371873
C	1.032038	0.553668	-1.786059
C	-0.049252	-0.137355	0.325332
C	0.070758	-0.787752	1.409727
C	-0.137648	-1.790023	2.403479
C	-1.016803	-1.548443	3.477941
C	0.535603	-3.023957	2.313083
C	-1.221752	-2.531794	4.436548
H	-1.527149	-0.585099	3.545090
C	0.326322	-3.997695	3.281827
H	1.220129	-3.200280	1.480871
C	-0.550248	-3.753454	4.341085
H	-1.904997	-2.346701	5.268265
H	0.849272	-4.954100	3.213669
H	-0.710276	-4.521510	5.101582
O	0.776525	1.177322	-2.779661
O	1.663078	-0.604651	-1.749018
C	2.206334	-1.065660	-2.982308
H	2.637246	-2.051212	-2.776323
H	1.422008	-1.136283	-3.748633
H	2.986874	-0.374612	-3.330359
C	-0.871106	2.781327	0.454969
O	-0.653458	2.513722	1.621045
O	-1.666203	3.736316	-0.009228
C	-2.363370	4.669626	0.877157
C	-3.333051	3.903713	1.767891
H	-2.796712	3.246485	2.463915
H	-3.937713	4.614518	2.349879
H	-4.018751	3.297662	1.156110
C	-1.348880	5.470278	1.682391
H	-0.638048	5.975254	1.011173
H	-1.870364	6.243446	2.265319
H	-0.793081	4.826976	2.376211
C	-3.111876	5.565514	-0.097450
H	-3.809138	4.976117	-0.710742

H	-3.687843	6.323962	0.451400
H	-2.410034	6.080432	-0.769200
C	5.262129	0.241632	-0.315637
C	6.047170	-0.633977	0.446219
C	5.742761	0.731845	-1.539214
C	7.300533	-1.021102	-0.019106
H	5.672632	-1.004715	1.401794
C	6.995265	0.340864	-1.995986
H	5.123099	1.426154	-2.110783
C	7.774376	-0.536360	-1.237798
H	7.913784	-1.702240	0.574925
H	7.372569	0.725818	-2.945997
H	8.760330	-0.839326	-1.598270
H	-0.414847	2.415938	-1.540608
Au	-1.760977	-0.993710	-0.595636
P	-3.609567	-1.764766	-1.750367
C	-5.164953	-1.650252	-0.810032
H	-6.009281	-2.011914	-1.416724
H	-5.089660	-2.255597	0.104566
H	-5.346198	-0.605031	-0.521525
C	-3.486987	-3.508184	-2.262736
H	-3.377954	-4.147800	-1.375233
H	-4.386980	-3.810201	-2.820010
H	-2.602278	-3.645100	-2.900758
C	-3.904839	-0.830853	-3.285284
H	-3.026652	-0.910300	-3.941873
H	-4.789458	-1.221680	-3.810897
H	-4.063128	0.230725	-3.047056

TS6

C	4.175959	0.268951	0.486875
C	2.262362	0.985379	1.120308
C	2.441480	-0.310160	1.641580
O	3.601312	-0.767085	1.223150
H	1.697701	1.741216	1.673905
N	3.433764	1.316060	0.431830
C	1.457669	-1.128752	2.327377
H	1.879927	-2.104680	2.600836
H	1.107254	-0.622611	3.241877
N	0.211717	1.937750	-0.263775
C	0.762142	0.680225	-0.092708
C	1.509718	0.322557	-1.384884
C	-0.074688	-0.414336	0.428312

C	0.248032	-1.312078	1.375059
C	-0.524865	-2.549411	1.640404
C	-0.884282	-2.905309	2.949081
C	-0.881956	-3.408624	0.588873
C	-1.614007	-4.065772	3.195346
H	-0.614782	-2.257362	3.787130
C	-1.600222	-4.575627	0.837982
H	-0.564065	-3.162801	-0.427308
C	-1.974267	-4.904318	2.141080
H	-1.901199	-4.318040	4.218741
H	-1.857185	-5.241711	0.010545
H	-2.535936	-5.820689	2.336916
O	1.516558	1.049248	-2.341108
O	2.111152	-0.846829	-1.305678
C	2.825708	-1.269189	-2.468132
H	3.259788	-2.242956	-2.218696
H	2.139558	-1.358932	-3.321514
H	3.615549	-0.546254	-2.715301
C	-0.460566	2.608614	0.740018
O	-0.506056	2.190369	1.875323
O	-0.977708	3.723395	0.261332
C	-1.683582	4.688176	1.116552
C	-2.928730	4.030701	1.695529
H	-2.668621	3.214334	2.381587
H	-3.513050	4.779588	2.249715
H	-3.564266	3.635772	0.888095
C	-0.742475	5.200341	2.197781
H	0.174809	5.609874	1.748640
H	-1.235790	6.010660	2.753999
H	-0.474648	4.407583	2.907732
C	-2.049622	5.795326	0.141765
H	-2.684304	5.407046	-0.667999
H	-2.600477	6.589849	0.664552
H	-1.146412	6.235178	-0.305163
C	5.486065	0.028417	-0.091057
C	6.158729	-1.186764	0.107066
C	6.078441	1.044667	-0.857917
C	7.413891	-1.379580	-0.460974
H	5.700398	-1.974245	0.708132
C	7.331640	0.840913	-1.421441
H	5.542585	1.985756	-0.997444
C	8.000158	-0.369518	-1.224060
H	7.940476	-2.323527	-0.304915
H	7.794244	1.631245	-2.016475

H	8.986813	-0.525138	-1.666693
H	0.427786	2.410344	-1.140302
Au	-1.930649	-0.509929	-0.536625
P	-4.025434	-0.610357	-1.532481
C	-5.368211	0.012902	-0.467483
H	-6.339985	-0.059178	-0.979481
H	-5.402641	-0.573695	0.461830
H	-5.172714	1.062570	-0.204840
C	-4.535575	-2.303223	-1.979239
H	-4.525243	-2.937532	-1.081087
H	-5.544974	-2.306465	-2.418233
H	-3.823374	-2.721139	-2.705229
C	-4.175728	0.344511	-3.078611
H	-3.441052	-0.020863	-3.810380
H	-5.187899	0.247878	-3.500330
H	-3.965241	1.405096	-2.879643

3a-Proton

C	-2.334664	3.332613	-1.144032
C	-2.306981	2.408907	-3.240088
C	-1.763834	2.166251	-0.532530
C	-1.758987	1.202594	-2.751981
C	-1.486420	1.064341	-1.398772
N	-2.591141	3.426889	-2.465459
C	-0.896780	-0.219860	-0.853443
N	0.531254	-0.333856	-1.108049
C	1.394535	-0.401934	-0.067689
C	2.789927	-0.390738	0.090663
C	1.146859	-0.616594	1.353809
C	2.647228	-0.538436	1.586850
O	3.374258	-0.576079	2.537466
O	0.141981	-0.761031	2.028186
N	3.771917	-0.252557	-0.803765
C	5.173738	-0.210705	-0.639950
C	5.797550	-0.302431	0.607574
C	5.942686	-0.068231	-1.801651
C	7.187816	-0.249330	0.673587
H	5.208351	-0.412736	1.519908
C	7.329942	-0.016763	-1.718146
H	5.453558	0.004477	-2.778190
C	7.960201	-0.107234	-0.478296
H	9.049162	-0.066696	-0.410317
H	-1.544264	0.398613	-3.463656

H	-0.995006	-0.226243	0.238946
H	3.483089	-0.173465	-1.772781
H	0.860586	-0.006811	-2.009982
C	-1.511150	2.162992	0.865776
C	-2.646454	4.452044	-0.321675
H	-1.046918	1.300616	1.344901
C	-2.402045	4.426896	1.021525
C	-1.822503	3.275989	1.630942
H	-2.631754	5.279150	1.664125
H	-3.081630	5.325682	-0.809973
C	-1.616335	-1.482300	-1.360026
C	-1.197848	-2.776619	-0.643906
H	-1.470661	-1.572999	-2.447991
C	-3.518528	-1.431835	0.250373
C	-2.448024	-3.600590	-0.322861
H	-0.653058	-2.538690	0.281459
H	-0.510632	-3.347855	-1.283173
C	-3.300132	-2.877152	0.744991
H	-2.920374	-0.705585	0.817085
H	-4.568519	-1.115508	0.313445
H	-2.158763	-4.589635	0.058067
N	-3.109358	-1.328622	-1.189936
C	-2.707445	-2.893609	2.126077
H	-1.764257	-2.348536	2.267513
C	-3.266019	-3.517820	3.163435
H	-4.212859	-4.060560	3.071680
H	-2.792968	-3.511022	4.148452
C	-3.288880	-3.749285	-1.595275
H	-2.679668	-4.179897	-2.404068
H	-4.129215	-4.438118	-1.429257
C	-3.823635	-2.366709	-1.998773
H	-3.658417	-2.130261	-3.058968
H	-4.895643	-2.245578	-1.789423
H	-4.275123	-3.389443	0.787863
O	-1.618123	3.376652	2.943459
C	-1.033311	2.302810	3.643118
H	-1.647941	1.389918	3.572498
H	-0.021045	2.078166	3.269100
H	-0.968854	2.614671	4.691918
H	-2.515789	2.520251	-4.310564
H	7.919866	0.095352	-2.630483
H	7.671651	-0.320724	1.650375
H	-3.349581	-0.389407	-1.538678

3a

C	-1.771790	3.633313	-1.134361
C	-2.480096	2.590128	-3.036558
C	-1.425033	2.398638	-0.499653
C	-2.178632	1.314729	-2.511784
C	-1.642470	1.198080	-1.245812
N	-2.284464	3.708996	-2.383770
C	-1.219013	-0.142501	-0.678425
N	0.218954	-0.313673	-0.897660
C	1.108982	-0.494071	0.079677
C	2.505908	-0.628898	0.137838
C	0.967075	-0.678668	1.533823
C	2.484175	-0.762116	1.624403
O	3.288093	-0.877049	2.513731
O	0.030582	-0.705930	2.301937
N	3.415637	-0.582399	-0.855368
C	4.813263	-0.694392	-0.818107
C	5.530102	-0.874370	0.370825
C	5.499624	-0.618944	-2.038597
C	6.918027	-0.976569	0.319029
H	5.005672	-0.930508	1.328043
C	6.885943	-0.722907	-2.071920
H	4.941962	-0.476636	-2.969528
C	7.605678	-0.903102	-0.891811
H	8.694351	-0.984747	-0.916029
H	-2.396964	0.430895	-3.112484
H	-1.352681	-0.130398	0.411913
H	3.042772	-0.453905	-1.788761
H	0.541649	-0.148346	-1.844766
C	-0.897163	2.421734	0.820181
C	-1.573425	4.848056	-0.420773
H	-0.644293	1.489054	1.322026
C	-1.065288	4.848102	0.848281
C	-0.724129	3.622690	1.484619
H	-0.911605	5.772783	1.408170
H	-1.847498	5.774280	-0.929248
C	-1.963953	-1.369549	-1.228336
C	-1.526568	-2.679411	-0.516748
H	-1.713314	-1.447174	-2.301777
C	-3.896656	-1.302187	0.205429
C	-2.789660	-3.487641	-0.199919
H	-0.977348	-2.464475	0.413293
H	-0.848122	-3.270148	-1.152035

C	-3.669472	-2.720363	0.814138
H	-3.396818	-0.534154	0.815881
H	-4.966320	-1.042147	0.213288
H	-2.515622	-4.466934	0.220952
N	-3.411590	-1.212024	-1.165494
C	-3.100307	-2.666743	2.199028
H	-2.159099	-2.114137	2.326811
C	-3.665636	-3.221237	3.273524
H	-4.609948	-3.772440	3.203965
H	-3.206813	-3.144193	4.262822
C	-3.598819	-3.664307	-1.489407
H	-2.982096	-4.171883	-2.249493
H	-4.470663	-4.312180	-1.306331
C	-4.036095	-2.255659	-1.968111
H	-3.773196	-2.092165	-3.026356
H	-5.127583	-2.128458	-1.894003
H	-4.635484	-3.251213	0.870455
O	-0.245263	3.745010	2.732168
C	0.142490	2.589259	3.431348
H	-0.688521	1.872782	3.541025
H	0.981064	2.074105	2.932865
H	0.468326	2.919892	4.425168
H	-2.905683	2.671052	-4.044490
H	7.405386	-0.661507	-3.031209
H	7.469070	-1.116189	1.252263

TS1-*RR*

C	0.309488	3.162641	1.198264
C	-0.235338	1.944822	-0.481471
C	0.588410	1.149778	0.399276
O	0.819158	1.911309	1.484992
H	-0.212453	1.825309	-1.565593
N	-0.248972	3.241120	0.049123
C	1.091264	-0.156785	0.323495
H	1.300401	-0.637638	1.286800
H	0.587929	-0.785994	-0.418015
Au	3.062716	0.188546	-0.476231
P	5.115773	0.541417	-1.575309
C	-5.789914	0.613940	3.673968
C	-5.837373	2.292521	2.126447
C	-5.848031	-0.406954	2.672377
C	-5.915501	1.373401	1.058484
C	-5.917421	0.017144	1.306587

N	-5.779997	1.939226	3.388231
C	-5.943809	-0.997260	0.176590
N	-4.589719	-1.428188	-0.146180
C	-4.203727	-2.695040	-0.142969
C	-3.026918	-3.341459	-0.582366
C	-4.843691	-3.971859	0.189934
C	-3.554560	-4.668040	-0.180737
O	-3.168711	-5.820936	-0.169074
O	-5.944423	-4.306957	0.597173
N	-1.932307	-2.790933	-1.125422
C	-0.886271	-3.422724	-1.798440
C	-0.682766	-4.810614	-1.762150
C	-0.022787	-2.624523	-2.567460
C	0.360614	-5.375602	-2.492605
H	-1.352488	-5.443167	-1.174025
C	1.013183	-3.204734	-3.292764
H	-0.187098	-1.545952	-2.614762
C	1.214149	-4.585100	-3.263076
H	2.025243	-5.039560	-3.836431
H	-5.975351	1.758957	0.041081
H	-6.472887	-1.895367	0.525096
H	-1.945544	-1.752754	-1.152455
H	-3.912494	-0.744495	-0.513466
C	5.315420	2.455620	-1.851781
C	0.457344	4.196641	2.212240
C	0.008168	5.496473	1.929099
C	1.024088	3.914586	3.463621
C	0.130783	6.497813	2.885099
H	-0.436037	5.701455	0.953022
C	1.139818	4.922954	4.417574
H	1.366743	2.902883	3.686800
C	0.696522	6.214114	4.131106
H	-0.218621	7.508110	2.659949
H	1.578721	4.698030	5.392324
H	0.789424	7.002964	4.881404
C	-5.840877	-1.771669	3.073341
C	-5.734524	0.228599	5.041801
H	-5.886435	-2.559497	2.321691
C	-5.731710	-1.090067	5.406546
C	-5.784468	-2.107539	4.414758
H	-5.689486	-1.395075	6.454381
H	-5.694083	1.024479	5.788336
C	-6.630989	-0.520157	-1.113896
C	-6.653747	-1.629865	-2.198047

H	-6.043991	0.330756	-1.499466
C	-8.917345	-1.085999	-0.603056
C	-8.032943	-1.609381	-2.863179
H	-6.466087	-2.621665	-1.756998
H	-5.857545	-1.465429	-2.939796
C	-9.115510	-2.019330	-1.837827
H	-8.557684	-1.663944	0.262773
H	-9.878690	-0.645560	-0.296029
H	-8.052971	-2.302299	-3.718240
N	-7.978363	-0.006566	-0.877443
C	-9.087768	-3.469309	-1.463871
H	-8.190826	-3.823771	-0.936586
C	-10.073058	-4.339088	-1.703023
H	-10.993545	-4.033998	-2.213320
H	-9.997711	-5.386639	-1.397631
C	-8.338630	-0.180661	-3.325603
H	-7.546369	0.162115	-4.010966
H	-9.283828	-0.155582	-3.890737
C	-8.416519	0.718681	-2.065886
H	-7.793664	1.621105	-2.179093
H	-9.446691	1.065548	-1.887642
C	5.748564	2.907594	-3.252368
H	5.723131	4.009101	-3.263723
H	6.771416	2.620888	-3.522850
H	5.067510	2.566957	-4.042916
C	6.296861	3.008163	-0.817084
H	6.303209	4.107701	-0.892742
H	5.999375	2.752184	0.207308
H	7.324772	2.657969	-0.975734
C	3.934172	3.080958	-1.596847
H	4.006258	4.169851	-1.751807
H	3.166214	2.693756	-2.282513
H	3.587615	2.910691	-0.568060
C	6.549988	-0.174225	-0.602517
C	6.338093	-0.806453	0.667906
C	7.866326	-0.102244	-1.115624
C	7.444631	-1.338328	1.377868
C	8.923352	-0.694074	-0.408009
C	8.743664	-1.291203	0.837271
C	8.281818	0.642726	-2.347020
H	8.783762	-0.012088	-3.073381
H	7.439381	1.112459	-2.849918
H	9.011570	1.423446	-2.082700
C	5.031986	-0.936025	1.419785

C	4.311081	-2.159826	1.402091
C	4.682185	0.047822	2.380495
C	3.286028	-2.366471	2.330849
C	3.638753	-0.207947	3.276393
C	2.933938	-1.411957	3.286919
H	2.754014	-3.320575	2.312671
H	3.382630	0.553022	4.019965
C	4.643923	-3.291480	0.441345
H	5.296386	-2.872713	-0.338467
C	1.889141	-1.684206	4.353093
H	1.616817	-0.704953	4.784173
C	5.440301	1.358260	2.521246
H	6.136961	1.423181	1.672398
C	3.397195	-3.862125	-0.237088
H	2.784243	-4.447573	0.466041
H	2.753190	-3.075978	-0.657589
H	3.682336	-4.539597	-1.056340
C	5.417762	-4.424175	1.122692
H	4.835239	-4.853910	1.953696
H	5.619598	-5.233160	0.402853
H	6.380470	-4.084877	1.525275
C	0.610269	-2.324747	3.817001
H	0.134707	-1.713399	3.035089
H	0.800495	-3.324631	3.395587
H	-0.120488	-2.451555	4.630816
C	2.493486	-2.528094	5.479885
H	1.764432	-2.676589	6.292242
H	2.790689	-3.522023	5.107629
H	3.387648	-2.048043	5.905988
C	4.512170	2.572038	2.467347
H	5.095942	3.505042	2.428433
H	3.851150	2.541746	1.588836
H	3.871525	2.628493	3.360534
C	6.282166	1.401113	3.799415
H	7.006085	0.576482	3.841204
H	6.843145	2.347244	3.857378
H	5.645181	1.335029	4.696125
C	7.322659	-1.954698	2.748582
H	6.290401	-2.034553	3.101840
H	7.769174	-2.960571	2.767796
H	7.886305	-1.362652	3.487853
C	9.924598	-1.824371	1.591138
H	9.952119	-1.427517	2.617039
H	9.886016	-2.922409	1.680632

H	10.862189	-1.549148	1.094401
O	10.179603	-0.597841	-0.941253
C	10.601979	-1.718644	-1.691760
H	11.594095	-1.482525	-2.098641
H	10.678759	-2.623196	-1.064614
H	9.913142	-1.931472	-2.527493
H	-10.096828	-1.811682	-2.297984
O	-5.775646	-3.363825	4.891314
C	-5.778340	-4.437301	3.980292
H	-4.881465	-4.423974	3.339285
H	-5.780041	-5.355798	4.579831
H	-6.668806	-4.423763	3.331149
N	-2.251726	0.150614	-1.264561
C	-2.093583	1.194420	-0.402209
C	-2.073892	0.717794	1.045346
C	-2.851329	2.404454	-0.623615
C	-3.558016	3.377163	-0.811861
C	-4.384929	4.523850	-1.002641
C	-4.248304	5.645084	-0.161479
C	-5.350439	4.552691	-2.027233
C	-5.054508	6.763743	-0.347901
H	-3.503133	5.625703	0.636527
C	-6.153200	5.675558	-2.204354
H	-5.465187	3.684376	-2.679883
C	-6.007624	6.783427	-1.367874
H	-4.939215	7.628405	0.310081
H	-6.899575	5.685974	-3.002237
H	-6.639473	7.663426	-1.509883
O	-1.930267	-0.435756	1.362194
O	-2.199744	1.721385	1.905821
C	-2.156541	1.382791	3.287938
H	-2.422042	2.294836	3.832916
H	-2.875373	0.583438	3.511967
H	-1.146252	1.051187	3.569442
C	-2.365844	0.324997	-2.611245
O	-2.901855	-0.495707	-3.338252
O	-1.769488	1.444978	-3.072096
C	-1.745797	1.803433	-4.481218
C	-1.005540	3.134260	-4.488949
H	-0.928632	3.518804	-5.516310
H	-1.536908	3.877261	-3.876210
H	0.012027	3.016578	-4.086545
C	-0.970737	0.755801	-5.272445
H	0.037515	0.626491	-4.848576

H	-1.488756	-0.211091	-5.257007
H	-0.861526	1.085053	-6.316587
C	-3.166799	1.981646	-5.002588
H	-3.713828	1.031197	-4.980598
H	-3.705848	2.720296	-4.390268
H	-3.136862	2.352501	-6.037927
H	-5.823985	3.367099	1.906401
H	1.664335	-2.567155	-3.895449
H	0.503288	-6.458954	-2.457920
C	4.886233	-0.399579	-3.236225
C	3.579539	0.116694	-3.856624
H	3.353236	-0.487529	-4.750512
H	2.729438	0.028218	-3.162289
H	3.645119	1.163925	-4.183202
C	4.710256	-1.870417	-2.857359
H	4.481944	-2.448537	-3.767640
H	5.628791	-2.288529	-2.419673
H	3.883182	-2.018732	-2.150208
C	5.994275	-0.326713	-4.288288
H	5.639236	-0.870700	-5.178883
H	6.234651	0.693509	-4.609826
H	6.913553	-0.824055	-3.956869

TS1-*RS*

C	-0.534146	2.926654	-2.202435
C	0.400067	1.143106	-1.474823
C	-0.644996	1.503450	-0.551909
H	0.647086	0.098021	-1.656733
C	-1.135838	0.876318	0.602152
H	-0.442151	0.141379	1.028584
H	-1.638511	1.533126	1.321407
Au	-2.696709	-0.414083	-0.135445
P	-4.169982	-2.085714	-0.897701
C	7.514338	1.235444	-1.508674
C	6.338847	2.556947	-0.063608
C	7.092072	0.031987	-0.859518
C	5.871396	1.439383	0.659763
C	6.233655	0.164148	0.277661
N	7.130057	2.471781	-1.107289
C	5.678452	-1.064603	0.976119
N	4.463430	-1.475890	0.275707
C	4.341395	-2.677638	-0.264141
C	3.440145	-3.276059	-1.178809

C	5.149873	-3.906806	-0.210973
C	4.088824	-4.596744	-1.032291
O	3.895529	-5.738625	-1.403302
O	6.231903	-4.212539	0.261518
N	2.445552	-2.709005	-1.881005
C	1.577141	-3.293446	-2.805133
C	0.865365	-2.438744	-3.663986
C	1.386425	-4.679546	-2.903986
C	-0.025748	-2.961500	-4.596220
H	1.031738	-1.359886	-3.608464
C	0.492145	-5.186767	-3.844275
H	1.963564	-5.355579	-2.269340
C	-0.223648	-4.339870	-4.691771
H	-0.920828	-4.749904	-5.425912
H	5.222161	1.603808	1.520692
H	6.391873	-1.889992	0.855576
H	2.468842	-1.682772	-1.886029
H	3.679262	-0.791105	0.219032
C	-3.280792	-3.707682	-0.374187
C	-0.934584	4.133701	-2.910497
C	-0.316602	4.441004	-4.132871
C	-1.921146	4.988102	-2.396308
C	-0.685618	5.584709	-4.830719
H	0.454375	3.771593	-4.519079
C	-2.283975	6.133105	-3.101483
H	-2.400700	4.752852	-1.444522
C	-1.669758	6.433529	-4.317393
H	-0.201289	5.819749	-5.781310
H	-3.052057	6.796486	-2.697257
H	-1.956531	7.333292	-4.867013
C	7.539385	-1.220297	-1.364814
C	8.376565	1.137520	-2.636082
H	7.223391	-2.143654	-0.881063
C	8.797734	-0.077614	-3.103556
C	8.376406	-1.275860	-2.464364
H	9.459908	-0.160807	-3.968056
H	8.689377	2.068374	-3.113718
C	5.434091	-0.896974	2.481649
C	4.903963	-2.193189	3.141748
H	4.667926	-0.118258	2.612374
C	7.626147	-1.486566	3.307940
C	5.627048	-2.367986	4.480937
H	5.080987	-3.071916	2.501468
H	3.817248	-2.114283	3.288885

C	7.130266	-2.635744	4.238999
H	7.871852	-1.880911	2.309545
H	8.558530	-1.050407	3.699842
H	5.189354	-3.206935	5.043840
N	6.637392	-0.424906	3.171306
C	7.427320	-3.990941	3.676121
H	7.030837	-4.200338	2.672685
C	8.147652	-4.932776	4.292062
H	8.575266	-4.766641	5.287408
H	8.337971	-5.903753	3.825596
C	5.506961	-1.067290	5.282696
H	4.442508	-0.811372	5.411824
H	5.929871	-1.197824	6.291683
C	6.256522	0.041124	4.500744
H	5.634815	0.944423	4.395254
H	7.174760	0.346482	5.027517
C	-1.850925	-3.624320	-0.925989
H	-1.277789	-4.483526	-0.541902
H	-1.803836	-3.670443	-2.023023
H	-1.338369	-2.706111	-0.600472
C	-3.892674	-5.050323	-0.779148
H	-3.203244	-5.840292	-0.439139
H	-4.854575	-5.232541	-0.285109
H	-4.015569	-5.177359	-1.861153
C	-3.221986	-3.679163	1.153640
H	-2.744809	-2.764870	1.530376
H	-4.223142	-3.766570	1.601469
H	-2.624135	-4.537286	1.501409
C	-5.844880	-1.954333	-0.065169
C	-6.120119	-0.908021	0.876332
C	-6.861255	-2.894908	-0.353917
C	-7.397142	-0.835641	1.488571
C	-8.090008	-2.821533	0.319365
C	-8.386400	-1.800169	1.218772
C	-6.788768	-3.959616	-1.405580
H	-6.947806	-4.962878	-0.985302
H	-7.587958	-3.807705	-2.147408
H	-5.832287	-3.965217	-1.924076
C	-5.203530	0.224008	1.284479
C	-5.281692	1.464197	0.599955
C	-4.492030	0.166665	2.512431
C	-4.664525	2.595190	1.145354
C	-3.903879	1.330836	3.015242
C	-3.983173	2.561639	2.360813

H	-4.743730	3.541297	0.605291
H	-3.369437	1.281333	3.968439
C	-6.057915	1.639013	-0.695112
H	-6.368666	0.637498	-1.027045
C	-3.393262	3.804477	3.000280
H	-2.546429	3.465758	3.622783
C	-4.375103	-1.107522	3.335621
H	-4.674130	-1.941723	2.685152
C	-5.199102	2.261758	-1.797900
H	-5.729996	2.238821	-2.762470
H	-4.965781	3.316200	-1.582024
H	-4.242298	1.731625	-1.916988
C	-7.334073	2.461179	-0.495756
H	-7.884109	2.552752	-1.445669
H	-8.007249	2.000521	0.239909
H	-7.099208	3.479353	-0.146007
C	-2.850826	4.813966	1.992331
H	-2.318172	5.625421	2.511730
H	-2.152549	4.342911	1.283328
H	-3.659370	5.281386	1.407907
C	-4.413147	4.458946	3.937541
H	-4.767733	3.751144	4.701936
H	-3.971415	5.326044	4.453882
H	-5.291336	4.811586	3.372585
C	-2.940947	-1.366025	3.805234
H	-2.640895	-0.663397	4.598531
H	-2.853062	-2.380934	4.222788
H	-2.212663	-1.268222	2.986096
C	-5.316851	-1.110413	4.543622
H	-5.104558	-0.258917	5.210380
H	-6.371980	-1.052463	4.247150
H	-5.182459	-2.034458	5.127889
C	-7.794868	0.270947	2.432622
H	-6.979430	0.962082	2.665337
H	-8.623440	0.859590	2.006271
H	-8.175039	-0.139307	3.380802
C	-9.747544	-1.719928	1.841728
H	-10.429235	-2.448099	1.387044
H	-9.707665	-1.909646	2.926968
H	-10.183440	-0.717594	1.715089
O	-9.044838	-3.749996	0.006173
C	-9.104591	-4.867231	0.869825
H	-9.382768	-4.573867	1.896471
H	-9.872354	-5.544432	0.472855

H	-8.140329	-5.402956	0.909366
H	7.638500	-2.559299	5.215813
O	8.850192	-2.409553	-3.009174
C	8.466138	-3.643696	-2.451674
H	7.375098	-3.792641	-2.509838
H	8.967183	-4.422186	-3.039750
H	8.775426	-3.730536	-1.396877
N	2.313481	0.576158	0.391560
C	2.111591	1.521269	-0.561784
C	2.935120	1.175085	-1.811766
C	2.025942	2.946523	-0.328765
C	1.946991	4.161053	-0.284407
C	1.909854	5.587132	-0.222989
C	2.943340	6.344210	-0.806908
C	0.848402	6.257711	0.412657
C	2.914599	7.734000	-0.748202
H	3.767554	5.827748	-1.303403
C	0.825842	7.648426	0.461941
H	0.042847	5.677325	0.865744
C	1.857348	8.390284	-0.115392
H	3.724119	8.310714	-1.202084
H	-0.004444	8.157892	0.957071
H	1.837342	9.481936	-0.072811
O	3.026730	0.049557	-2.250158
O	3.537237	2.210560	-2.348197
C	4.308722	1.970697	-3.522834
H	3.656770	1.628184	-4.338644
H	4.773143	2.928346	-3.779491
H	5.079670	1.213033	-3.328613
C	2.058201	0.727109	1.717892
O	2.259022	-0.178726	2.517791
O	1.596887	1.935344	2.096985
C	1.388689	2.289527	3.488593
C	0.912624	3.734098	3.413902
H	0.710788	4.120430	4.423565
H	1.674049	4.370187	2.939830
H	-0.011497	3.803956	2.821820
C	0.312603	1.410274	4.117570
H	0.636415	0.363535	4.162439
H	0.099698	1.762022	5.138407
H	-0.619955	1.467877	3.537069
C	2.703704	2.208373	4.256357
H	3.049554	1.169994	4.330646
H	3.476881	2.809490	3.753498

H	2.566171	2.609827	5.271594
H	6.032751	3.562725	0.249354
H	0.356581	-6.269288	-3.913774
H	-0.565129	-2.280643	-5.259396
C	-4.310189	-1.927863	-2.828737
C	-4.170062	-3.224108	-3.636394
H	-4.991471	-3.936722	-3.495320
H	-4.180890	-2.948037	-4.703331
H	-3.218442	-3.740434	-3.453961
C	-3.159420	-1.010818	-3.271253
H	-2.174313	-1.434479	-3.028418
H	-3.210233	-0.884078	-4.365205
H	-3.226196	-0.015030	-2.811827
C	-5.635302	-1.249462	-3.177878
H	-5.746863	-0.285606	-2.665602
H	-5.651909	-1.050274	-4.261773
H	-6.508281	-1.870062	-2.939242
N	0.307111	2.025713	-2.556648
O	-1.143735	2.669370	-0.987476

TS1-*SR*

C	1.668922	3.458431	-1.955791
C	-0.042075	2.292122	-1.461271
C	0.967251	1.995886	-0.496539
H	-0.869347	1.628626	-1.705101
C	1.005208	1.120393	0.609011
H	-0.002245	0.851833	0.956671
H	1.706662	1.413352	1.400915
Au	1.828364	-0.684847	-0.226629
P	2.464121	-2.728681	-1.239768
C	2.507420	-2.422175	-3.161973
C	1.045274	-3.925225	-0.744842
C	2.634121	4.396045	-2.507982
C	3.844711	4.666448	-1.852873
C	2.335484	5.054038	-3.712353
C	4.745658	5.575780	-2.401722
H	4.071857	4.168988	-0.908866
C	3.239734	5.960693	-4.253014
H	1.387276	4.841687	-4.209927
C	4.447803	6.223274	-3.600659
H	5.685998	5.783054	-1.885767
H	3.002658	6.468424	-5.190893
H	5.156443	6.936575	-4.028367

C	1.778153	-1.092868	-3.415058
H	2.265308	-0.248526	-2.908051
H	0.728114	-1.125024	-3.089522
H	1.787864	-0.887488	-4.498081
C	1.815189	-3.466552	-4.047451
H	1.859334	-3.097293	-5.084953
H	0.752461	-3.597546	-3.804047
H	2.297627	-4.450796	-4.051744
C	3.964691	-2.265565	-3.598894
H	4.482015	-1.490887	-3.019017
H	3.984534	-1.955468	-4.656529
H	4.537447	-3.197761	-3.513650
C	1.040702	-5.348707	-1.304138
H	1.024453	-5.397149	-2.399668
H	0.119994	-5.836943	-0.945141
H	1.883431	-5.942570	-0.930320
C	1.102151	-4.033447	0.778277
H	1.074129	-3.047262	1.259424
H	2.004799	-4.565985	1.113208
H	0.223180	-4.593839	1.134516
C	-0.264343	-3.231292	-1.140004
H	-0.336654	-2.220259	-0.709567
H	-1.108504	-3.822901	-0.750894
H	-0.397339	-3.150133	-2.227590
C	4.114303	-3.369441	-0.615585
C	4.867788	-2.633766	0.356648
C	4.636322	-4.595097	-1.092462
C	6.108740	-3.142204	0.816681
C	5.841176	-5.089550	-0.570298
C	6.597974	-4.380106	0.359121
C	4.057247	-5.415132	-2.204501
H	3.843549	-6.445526	-1.887051
H	4.782198	-5.492102	-3.029655
H	3.134654	-4.990613	-2.592999
C	4.516388	-1.290347	0.951330
C	5.040722	-0.109179	0.369458
C	3.920794	-1.213919	2.237717
C	4.956710	1.100383	1.069488
C	3.864333	0.020164	2.890272
C	4.384131	1.191974	2.337274
H	5.375747	1.996909	0.606835
H	3.405310	0.072530	3.881794
C	5.754044	-0.105341	-0.974062
H	5.645597	-1.112162	-1.405079

C	4.367541	2.477190	3.143865
H	3.472697	2.428504	3.789115
C	3.379901	-2.434928	2.965372
H	3.279181	-3.237473	2.221316
C	5.137910	0.896979	-1.951276
H	5.313591	1.934793	-1.629036
H	4.050363	0.760830	-2.043346
H	5.585110	0.787560	-2.951779
C	7.254486	0.162593	-0.828621
H	7.440435	1.162481	-0.404316
H	7.748851	0.121144	-1.812134
H	7.741151	-0.573797	-0.175214
C	4.256557	3.741128	2.297692
H	5.163392	3.909126	1.694492
H	4.132467	4.623918	2.943105
H	3.395498	3.700244	1.613606
C	5.590148	2.541176	4.065237
H	5.646017	1.659044	4.720942
H	5.554684	3.438876	4.702943
H	6.520468	2.580792	3.475361
C	2.003630	-2.178956	3.581270
H	1.315204	-1.689700	2.876360
H	2.069891	-1.533330	4.470395
H	1.541693	-3.125033	3.902694
C	4.337868	-2.941003	4.048154
H	5.304393	-3.255770	3.634297
H	3.897811	-3.806344	4.568864
H	4.530087	-2.159624	4.801364
C	6.990350	-2.398706	1.787924
H	7.948644	-2.132767	1.312604
H	7.249358	-3.033309	2.649350
H	6.538544	-1.477432	2.167536
C	7.920822	-4.914969	0.818698
H	8.210491	-5.798068	0.237412
H	7.896762	-5.191855	1.885579
H	8.713219	-4.159158	0.709940
O	6.310732	-6.275762	-1.065615
C	5.986884	-7.418218	-0.299328
H	4.895845	-7.528555	-0.174242
H	6.450069	-7.382063	0.701452
H	6.374072	-8.292537	-0.839128
N	-1.858337	2.735302	0.654439
C	-1.335190	3.541975	-0.280969
C	-2.179426	3.828764	-1.523857

C	-0.397979	4.550966	0.103514
C	0.395935	5.410776	0.448993
C	1.299505	6.432557	0.861207
C	1.925750	7.258948	-0.091805
C	1.573943	6.631229	2.228283
C	2.804145	8.257020	0.318112
H	1.717569	7.105454	-1.152516
C	2.450985	7.634064	2.627372
H	1.090268	5.991216	2.968952
C	3.068240	8.448324	1.675332
H	3.286788	8.891745	-0.428782
H	2.655485	7.782484	3.690347
H	3.757219	9.234723	1.992879
O	-2.943559	3.023096	-1.996717
O	-1.963011	5.035042	-2.015190
C	-2.654997	5.366673	-3.212392
H	-2.359997	4.687283	-4.024915
H	-2.368356	6.395419	-3.455891
H	-3.741933	5.299490	-3.063744
C	-3.138086	2.786133	1.003184
O	-3.744892	1.883537	1.611036
O	-3.731635	3.965461	0.725438
C	-5.061083	4.339503	1.175625
C	-6.113866	3.434291	0.551353
H	-7.118274	3.825511	0.772518
H	-6.044628	2.412354	0.943097
H	-5.989300	3.406813	-0.541619
C	-5.205544	5.762809	0.651974
H	-6.181755	6.177076	0.942251
H	-5.130969	5.781108	-0.445613
H	-4.414420	6.406801	1.063386
C	-5.119637	4.324300	2.698637
H	-4.329136	4.965331	3.117797
H	-4.995216	3.305712	3.086589
H	-6.091479	4.714958	3.035123
C	-8.771540	-1.405650	0.599641
C	-8.610555	0.571917	-0.529254
C	-7.410585	-1.757698	0.315393
C	-7.258947	0.328387	-0.867005
C	-6.633798	-0.824290	-0.447628
N	-9.345612	-0.250053	0.177045
C	-5.162304	-1.081868	-0.724984
N	-4.367098	-0.677142	0.429932
C	-3.596173	-1.501814	1.126286

C	-2.721346	-1.320101	2.225518
C	-3.263603	-2.927981	1.034870
C	-2.403184	-2.766604	2.265397
O	-1.733433	-3.514568	2.952290
O	-3.553795	-3.837170	0.273859
N	-2.408307	-0.188375	2.867900
C	-1.641328	0.000023	4.019080
C	-1.310950	1.321102	4.366305
C	-1.216783	-1.053820	4.840885
C	-0.579700	1.581126	5.520773
H	-1.625735	2.139502	3.714044
C	-0.483069	-0.776446	5.992544
H	-1.441188	-2.085803	4.562995
C	-0.162122	0.534857	6.345572
H	0.409419	0.739945	7.253646
H	-6.727615	1.067126	-1.466568
H	-5.009177	-2.164578	-0.836246
H	-2.819885	0.669042	2.449326
H	-4.363747	0.312757	0.704947
C	-6.915229	-2.993521	0.789859
C	-9.563164	-2.304467	1.355310
H	-5.893073	-3.318353	0.588090
C	-9.060448	-3.499541	1.813300
C	-7.717368	-3.853259	1.524405
H	-9.700636	-4.166532	2.391492
H	-10.595654	-2.014683	1.561577
C	-4.606447	-0.387615	-1.978382
C	-3.119824	-0.744448	-2.227854
H	-4.669900	0.700282	-1.807584
C	-5.167434	-2.003249	-3.677673
C	-2.930772	-0.935647	-3.735683
H	-2.830141	-1.660043	-1.687424
H	-2.467114	0.059066	-1.856026
C	-3.712468	-2.183300	-4.208538
H	-5.375155	-2.726907	-2.873580
H	-5.897109	-2.215340	-4.474684
H	-1.862464	-1.056375	-3.973739
N	-5.402528	-0.656440	-3.174194
C	-3.109396	-3.483952	-3.778561
H	-3.105120	-3.686774	-2.698718
C	-2.603972	-4.406686	-4.602641
H	-2.596538	-4.260928	-5.688587
H	-2.184145	-5.342736	-4.223187
C	-3.505843	0.284466	-4.464431

H	-3.026914	1.200616	-4.083913
H	-3.287898	0.223542	-5.542550
C	-5.032907	0.312086	-4.202651
H	-5.361362	1.313654	-3.880954
H	-5.599629	0.067637	-5.115115
H	-3.720003	-2.165029	-5.311847
O	-7.152675	-5.006768	1.928945
C	-7.907633	-5.922670	2.682995
H	-7.245549	-6.771341	2.894404
H	-8.243856	-5.487925	3.639494
H	-8.786277	-6.288100	2.125006
H	-9.086193	1.499871	-0.870419
H	-0.157950	-1.606747	6.624969
H	-0.332699	2.614859	5.775934
N	0.509525	3.134474	-2.413575
O	2.004396	2.796799	-0.796159

TS1-*SS*

C	-0.488539	3.348515	1.203542
C	0.242492	2.082870	-0.370096
C	-0.605708	1.285512	0.494100
O	-0.946302	2.084678	1.524134
H	0.279251	1.923991	-1.448825
N	0.143375	3.403479	0.093079
C	-1.037175	-0.047200	0.454970
H	-1.255706	-0.508924	1.425835
H	-0.495496	-0.680198	-0.254433
Au	-3.021356	0.254274	-0.344628
P	-5.107576	0.705867	-1.339421
C	-4.866088	0.160473	-3.165338
C	-5.414921	2.621389	-1.192475
C	-0.782360	4.425863	2.138136
C	-1.476121	4.186086	3.332848
C	-0.360283	5.728704	1.827912
C	-1.744891	5.239242	4.204012
H	-1.803765	3.174204	3.575542
C	-0.634509	6.774367	2.701551
H	0.181026	5.901149	0.895533
C	-1.327213	6.532802	3.891054
H	-2.285422	5.047792	5.133840
H	-0.305919	7.786771	2.455462
H	-1.540729	7.356951	4.575930
C	-4.603011	-1.344855	-3.117927

H	-5.490860	-1.901640	-2.783345
H	-3.759769	-1.595130	-2.460555
H	-4.352986	-1.695020	-4.132697
C	-3.600795	0.868394	-3.672110
H	-3.717147	1.958903	-3.740204
H	-3.377831	0.501188	-4.687232
H	-2.728163	0.656842	-3.034742
C	-6.000891	0.393896	-4.164767
H	-6.883664	-0.213768	-3.933673
H	-5.639283	0.070164	-5.154449
H	-6.302763	1.443449	-4.260664
C	-6.404062	2.881039	-0.055011
H	-6.470481	3.968792	0.110008
H	-6.072438	2.426054	0.886994
H	-7.414854	2.515448	-0.275961
C	-4.066730	3.257999	-0.818231
H	-3.285646	3.058924	-1.566354
H	-3.702881	2.907576	0.157466
H	-4.199509	4.350172	-0.752507
C	-5.895350	3.335968	-2.461887
H	-5.210238	3.205068	-3.309421
H	-5.925221	4.414868	-2.240290
H	-6.906756	3.060306	-2.783458
C	-6.482955	-0.277739	-0.530885
C	-6.213085	-1.159505	0.567895
C	-7.812564	-0.163689	-1.001369
C	-7.276918	-1.888311	1.158027
C	-8.824576	-0.946805	-0.426127
C	-8.588795	-1.789990	0.656913
C	-8.289998	0.806195	-2.038584
H	-8.775755	0.297646	-2.883432
H	-7.481935	1.414857	-2.437920
H	-9.049892	1.474187	-1.604673
C	-4.886503	-1.390572	1.256540
C	-4.113704	-2.541007	0.950756
C	-4.566092	-0.643373	2.420469
C	-3.068183	-2.909627	1.804696
C	-3.509885	-1.057500	3.237265
C	-2.754062	-2.199111	2.963076
H	-2.484935	-3.804629	1.570908
H	-3.290565	-0.478884	4.139129
C	-4.410821	-3.435233	-0.242684
H	-5.076774	-2.871572	-0.911287
C	-1.679672	-2.694070	3.912703

H	-1.168363	-3.528995	3.403998
C	-5.362836	0.577033	2.852783
H	-6.079342	0.802016	2.048616
C	-3.148087	-3.797799	-1.025490
H	-2.516415	-4.508874	-0.470655
H	-2.529505	-2.917077	-1.253497
H	-3.413367	-4.280112	-1.978543
C	-5.149194	-4.716280	0.158005
H	-6.128779	-4.508631	0.607084
H	-4.560026	-5.300800	0.883109
H	-5.313743	-5.350833	-0.727221
C	-2.308149	-3.250963	5.193091
H	-3.038889	-4.042726	4.968469
H	-2.831573	-2.459509	5.753920
H	-1.535874	-3.675635	5.853780
C	-0.623903	-1.636732	4.234211
H	0.142740	-2.054734	4.905492
H	-1.065199	-0.765464	4.745316
H	-0.111671	-1.278491	3.328479
C	-4.462964	1.799046	3.042193
H	-5.064650	2.707889	3.199780
H	-3.811243	1.960157	2.170426
H	-3.813188	1.682491	3.924076
C	-6.177857	0.321633	4.123215
H	-6.883714	-0.510530	3.998209
H	-6.757532	1.218511	4.393063
H	-5.521230	0.080457	4.974643
C	-7.094317	-2.780637	2.359900
H	-7.662799	-2.388944	3.219076
H	-6.050952	-2.886928	2.670794
H	-7.497471	-3.786639	2.167362
C	-9.725179	-2.533348	1.291735
H	-9.626945	-3.621916	1.148258
H	-10.685792	-2.212345	0.872530
H	-9.752284	-2.362170	2.378429
O	-10.095770	-0.800619	-0.910211
C	-10.483220	-1.752257	-1.880918
H	-9.805034	-1.744406	-2.751686
H	-11.494414	-1.483784	-2.214064
H	-10.503288	-2.773654	-1.464136
N	2.384026	0.422724	-1.000556
C	2.104903	1.453480	-0.148410
C	2.010402	0.976889	1.295635
C	2.826486	2.700585	-0.289520

C	3.488333	3.717403	-0.384194
C	4.235607	4.929682	-0.478233
C	5.242100	5.085233	-1.450333
C	3.970360	5.993907	0.405063
C	5.959693	6.274841	-1.533835
H	5.459022	4.261930	-2.133933
C	4.691698	7.180068	0.312377
H	3.191527	5.877609	1.161728
C	5.687446	7.324868	-0.655395
H	6.739483	6.382485	-2.291575
H	4.475845	7.999193	1.002634
H	6.252983	8.257226	-0.724451
O	1.935834	-0.182011	1.617367
O	1.985972	1.993151	2.152864
C	1.834848	1.668795	3.529153
H	0.882060	1.148601	3.702128
H	1.848639	2.621853	4.068940
H	2.660824	1.028665	3.869902
C	2.610726	0.628431	-2.328895
O	3.206216	-0.175992	-3.025597
O	2.047448	1.756375	-2.815735
C	2.183220	2.164232	-4.204589
C	3.653979	2.361567	-4.553057
H	4.112726	3.088837	-3.866799
H	3.741896	2.754584	-5.576807
H	4.204479	1.415287	-4.486439
C	1.443064	3.494305	-4.253134
H	1.901930	4.220080	-3.565771
H	0.388494	3.364085	-3.966849
H	1.476508	3.908273	-5.271524
C	1.510445	1.146719	-5.119415
H	1.539676	1.507733	-6.158429
H	0.454790	1.014324	-4.835452
H	2.016818	0.175106	-5.066137
C	7.927966	0.376779	-1.980243
C	7.284795	2.038152	-0.551946
C	7.230691	-0.626132	-1.235558
C	6.566730	1.134268	0.260182
C	6.526011	-0.205536	-0.063113
N	7.942429	1.686104	-1.631221
C	5.729329	-1.207457	0.752738
N	4.466577	-1.479142	0.076855
C	4.042249	-2.699701	-0.206927
C	2.901278	-3.193312	-0.879905

C	4.601052	-4.050835	-0.065003
C	3.344332	-4.595394	-0.702119
O	2.930574	-5.708227	-0.966508
O	5.638061	-4.514864	0.379893
N	1.870281	-2.499655	-1.385571
C	0.890998	-2.929769	-2.281556
C	0.698218	-4.280683	-2.608374
C	0.089489	-1.955499	-2.899883
C	-0.268276	-4.633406	-3.547843
H	1.313570	-5.048686	-2.132774
C	-0.869050	-2.324298	-3.838693
H	0.239513	-0.900942	-2.658869
C	-1.055459	-3.665775	-4.173241
H	-1.804696	-3.953461	-4.914179
H	6.061522	1.514500	1.147876
H	6.278879	-2.160113	0.762246
H	1.928927	-1.479898	-1.218710
H	3.854592	-0.695836	-0.205040
C	7.275054	-1.975065	-1.681630
C	8.648226	-0.012509	-3.143573
H	6.751813	-2.747440	-1.120612
C	8.679424	-1.317181	-3.554381
C	7.984162	-2.315953	-2.818973
H	9.228989	-1.624462	-4.446722
H	9.174425	0.769209	-3.695388
C	5.474867	-0.811856	2.217856
C	4.716547	-1.919805	2.994448
H	4.853182	0.099357	2.207477
C	7.517022	-1.629301	3.208351
C	5.369826	-2.066133	4.372283
H	4.760631	-2.881254	2.459522
H	3.651330	-1.663484	3.091839
C	6.816087	-2.590351	4.218059
H	7.736576	-2.154857	2.265975
H	8.488883	-1.297054	3.605315
H	4.789207	-2.764454	4.994481
N	6.707393	-0.453048	2.918507
C	6.904469	-4.023228	3.791889
H	6.500458	-4.264099	2.798283
C	7.452818	-5.003059	4.515815
H	7.880854	-4.812170	5.506476
H	7.495777	-6.031532	4.145890
C	5.439943	-0.688391	5.038838
H	4.425449	-0.264119	5.118378

H	5.827751	-0.779619	6.065872
C	6.358266	0.209401	4.170990
H	5.872637	1.171276	3.938786
H	7.295299	0.446901	4.699120
H	7.303806	-2.501613	5.203951
O	8.081934	-3.561239	-3.315003
C	7.367152	-4.599825	-2.688728
H	7.669955	-4.734237	-1.637316
H	6.280260	-4.413895	-2.719653
H	7.590726	-5.515813	-3.249080
H	7.306071	3.100282	-0.279383
H	-1.470791	-1.548940	-4.319010
H	-0.402350	-5.689646	-3.795978

References:

[1] a) G. Dong, M. Bao, X. Xie, S. Jia, W. Hu, X. Xu, *Angew. Chem. Int. Ed.* **2021**, *60*, 1992; b) Y. Liu, Y. Shi, L. Wei, K. Zhao, J. Zhao, P. Zhang, X. Xu, P. Li, *Chem. Asian J.* **2021**, *16*, 2417.

[2] J. Yang, Z. Wang, Z. He, G. Li, L. Hong, W. Sun, R. Wang, *Angew. Chem. Int. Ed.* **2020**, *59*, 642.

[3] M. J. Frisch, G. W. Trucks, H. B. Schlegel, G. E. Scuseria, M. A. Robb, J. R. Cheeseman, G. Scalmani, V. Barone, B. Mennucci, G. A. Petersson, H. Nakatsuji, M. Caricato, X. Li, H. P. Hratchian, A. F. Izmaylov, J. Bloino, G. Zheng, J. L. Sonnenberg, M. Hada, M. Ehara, K. Toyota, R. Fukuda, J. Hasegawa, M. Ishida, T. Nakajima, Y. Honda, O. Kitao, H. Nakai, T. Vreven, J. A. Montgomery, J. E. Peralta, F. Ogliaro, M. Bearpark, J. J. Heyd, E. Brothers, K. N. Kudin, V. N. Staroverov, T. Keith, R. Kobayashi, J. Normand, K. Raghavachari, A. Rendell, J. C. Burant, S. S. Iyengar, J. Tomasi, M. Cossi, N. Rega, J. M. Millam, M. Klene, J. E. Knox, J. B. Cross, V. Bakken, C. Adamo, J. Jaramillo, R. Gomperts, R. E. Stratmann, O. Yazyev, A. J. Austin, R. Cammi, C. Pomelli, J. W. Ochterski, R. L. Martin, K. Morokuma, V. G. Zakrzewski, G. A. Voth, P. Salvador, J. J. Dannenberg, S. Dapprich, A. D. Daniels,

Ö. Farkas, J. B. Foresman, J. V. Ortiz, J. Cioslowski, D. J Fox, Gaussian 09, Revision E.01; Gaussian, Inc.: Wallingford, CT, **2013**.

[4] C. Adamo, V. Barone, *J. Chem. Phys.* **1999**, *110*, 6158.

[5] F. Weigend, R. Ahlrichs, *Phys. Chem. Chem. Phys.* **2005**, *7*, 3297.

[6] a) D. Andrae, U. Häußermann, M. Dolg, H. Stoll, H. Preuβ, *Theor. Chim. Acta.* **1990**, *77*, 123; b) K. A. Peterson, D. Figgen, E. Goll, H. Stoll, M. Dolg, *J. Chem. Phys.* **2003**, *119*, 11113.

[7] a) M. Bühl, C. Reimann, D. A. Pantazis, T. Bredow, F. Neese, *J. Chem. Theory Comput.* **2008**, *4*, 1449; b) R. Kang, H. Chen, S. Shaik, J. Yao, *J. Chem. Theory Comput.* **2011**, *7*, 4002.

[8] a) A. D. Boese, J. M. L Martin, *J. Chem. Phys.* **2004**, *121*, 3405; b) S. Grimme, S. Ehrlich, L. Goerigk, *J. Comput. Chem.* **2011**, *32*, 1456; c) L. Goerigk, S. Grimme, *Phys. Chem. Chem. Phys.* **2011**, *13*, 6670.

[9] A. V. Marenich, C. J. Cramer, D. G. Truhlar, *J. Phys. Chem. B* **2009**, *113*, 6378.

[10] R. Kang, W. Lai, J. Yao, S. Shaik, H. Chen, *J. Chem. Theory Comput.* **2012**, *8*, 3119.

[11] CYLview20; C. Y. Legault, Université de Sherbrooke, **2020** (http://www.cylview.org).

[12] NBO Version 3.1, E. D. Glendening, A. E. Reed, J. E. Carpenter, F. Weinhold.

[13] T. Lu, F. Chen, *J. Comput. Chem.* **2012**, *33*, 580.

[14] Visual Molecular Dynamics. Theoretical and Computational Biophysics Group. https://www.ks.uiuc.edu/Research/vmd/.

[15] a) P. Pracht, F. Bohle, S. Grimme, *Phys. Chem. Chem. Phys.* **2020**, *22*, 7169; b) S. Grimme, *J. Chem. Theory Comput.* **2019**, *15*, 2847.

[16] T. Lu, Molclus program, Version 1.9.9.9, http://www.keinsci.com/research/molclus.html. (accessed 06 30, 2023).

[17] S. Grimme, *Chem. Eur. J.* **2012**, *18*, 9955.

[18] T. Lu, Q. Chen, *Comput. Theor. Chem.* **2021**, *1200*, 113249.
